# A novel direct activator of AMPK inhibits prostate cancer growth by blocking lipogenesis

**DOI:** 10.1002/emmm.201302734

**Published:** 2014-02-04

**Authors:** Giorgia Zadra, Cornelia Photopoulos, Svitlana Tyekucheva, Pedram Heidari, Qing Ping Weng, Giuseppe Fedele, Hong Liu, Natalia Scaglia, Carmen Priolo, Ewa Sicinska, Umar Mahmood, Sabina Signoretti, Neal Birnberg, Massimo Loda

**Affiliations:** 1 Department of Medical Oncology, Dana-Farber Cancer Institute, Harvard Medical SchoolBoston, MA, USA; 2 Department of Pathology, Brigham and Women's Hospital, Harvard Medical SchoolBoston, MA, USA; 3 Department of Biostatistics and Computational Biology, Dana-Farber Cancer Institute, Harvard Medical SchoolBoston, MA, USA; 4 Department of Biostatistics, Harvard School of Public HealthBoston, MA, USA; 5 Division of Nuclear Medicine and Molecular Imaging, Department of Radiology, Massachusetts General HospitalBoston, MA, USA; 6 Mercury Pharmaceuticals, Inc.Woburn, MA, USA; 7 Center for Molecular Oncologic Pathology, Dana-Farber Cancer Institute, Harvard Medical SchoolBoston, MA, USA; 8 The Broad InstituteCambridge, MA, USA; 9 Division of Cancer Studies, King's College LondonLondon, UK

**Keywords:** AMPK direct activation, androgen signaling inhibitors, *de novo* lipogenesis, MT 63–78, prostate cancer

## Abstract

5′AMP-activated kinase (AMPK) constitutes a hub for cellular metabolic and growth control, thus representing an ideal therapeutic target for prostate cancers (PCas) characterized by increased lipogenesis and activation of mTORC1 pathway. However, whether AMPK activation itself is sufficient to block cancer cell growth remains to be determined. A small molecule screening was performed and identified MT 63–78, a specific and potent direct AMPK activator. Here, we show that direct activation of AMPK inhibits PCa cell growth in androgen sensitive and castration resistant PCa (CRPC) models, induces mitotic arrest, and apoptosis. *In vivo*, AMPK activation is sufficient to reduce PCa growth, whereas the allelic loss of its catalytic subunits fosters PCa development. Importantly, despite mTORC1 blockade, the suppression of *de novo* lipogenesis is the underpinning mechanism responsible for AMPK-mediated PCa growth inhibition, suggesting AMPK as a therapeutic target especially for lipogenesis-driven PCas. Finally, we demonstrate that MT 63–78 enhances the growth inhibitory effect of AR signaling inhibitors MDV3100 and abiraterone. This study thus provides a rationale for their combined use in CRPC treatment.

## Introduction

Metabolism in cancer cells is reprogrammed to facilitate the incorporation of nucleotides, amino acids, and lipids into the biomass needed to produce a new cell (Vander Heiden *et al*, 2009). Increased *de novo* fatty acid (FA) and cholesterol synthesis is a hallmark of prostate cancer (PCa) and correlates with tumor progression and poorer prognosis (reviewed in Pelton *et al*, 2012; Zadra *et al*, 2013). Increased protein synthesis due to alterations in phosphatase and tensin homologue/phosphatidylinositol 3-kinase/Akt/mammalian target of rapamycin (PTEN/PI3K/Akt/mTOR) pathway is also a common feature of both primary and metastatic PCas (Majumder & Sellers, 2005), and is associated with increased lipogenesis (Van de Sande *et al*, 2002). However, despite promising preclinical results, targeting mTOR complex 1 (mTORC1) with rapamycin and its analogues failed to show clinical efficacy in PCa (Amato *et al*, 2008) due to the emergence of survival feedback loops (O'Reilly *et al*, 2006; Carracedo *et al*, 2008; Rodrik-Outmezguine *et al*, 2011) or concomitant development of a tumor-driven lipogenic phenotype (Menendez & Lupu, 2007). The side effects of existing FA synthesis inhibitors have thus far precluded their clinical use in cancer (Flavin *et al*, 2011). Thus, alternative strategies to target these pathways in PCa patients are in great need.

5′ AMP-activated kinase (AMPK) is an energy sensor serine/threonine kinase that stands at the crossroads linking cell metabolism and oncogenesis. It is a heterotrimeric complex (composed of a catalytic α subunit and regulatory β and γ subunits) that responds to an increased AMP/ATP ratio by turning on ATP-generating pathways, while switching off ATP-consuming ones (Hardie & Carling, 1997). AMPK directs the switch from an anabolic to a catabolic state both by phosphorylating key metabolic targets, and by regulating their gene expression (Hardie, 2007). AMPK activation requires phosphorylation of Thr172 within the α subunit by an upstream Ser/Thr protein kinase, mainly the tumor suppressor liver kinase beta 1 (LKB1) and the calcium/calmodulin-dependent protein kinase kinase-β (CaMKKβ) (Woods *et al*, 2003; Hawley *et al*, 2005). Germline inactivating mutations of *LKB1* are responsible for the Peutz-Jeghers hereditary cancer syndrome. Somatic mutations of *LKB1* are also found in a significant fraction of non-small cell lung carcinomas (NSCLCs) and cervical tumors, thus suggesting that LKB1/AMPK axis may act as a tumor suppressor (Shackelford & Shaw, 2009). When pharmacologically activated, AMPK exerts pleitropic effects resulting in the suppression of tumorigenesis and tumor progression, including inhibition of mTORC1 signaling members tuberous sclerosis complex 2 (TSC2) and Raptor, FA and cholesterol biosynthesis, cell cycle progression, as well as induction of autophagy and apoptosis (Shaw, 2009; Fogarty & Hardie, 2010). In contrast, AMPK loss fosters tumor progression (Faubert *et al*, 2013). Epidemiologic and experimental evidence using metformin and AICAR suggest that AMPK activation is a valid anti-cancer strategy (Xiang *et al*, 2004; Evans *et al*, 2005; Bowker *et al*, 2006; Zakikhani *et al*, 2008). However, these activators are known to have additional molecular targets and AMPK-independent effects, leaving unanswered the question of whether AMPK activation *per se* is necessary and sufficient to affect tumor growth (Ben Sahra *et al*, 2008, 2011; Kalender *et al*, 2010; Santidrian *et al*, 2010). Direct targeting of AMPK with A-769662 (Abbott Laboratories) has initially shown promising results in the treatment of metabolic disorders and cancer (Cool *et al*, 2006; Huang *et al*, 2008). However, AMPK-independent toxic effects and reduced efficacy in cancer cells have been recently reported (Moreno *et al*, 2008; Garcia-Garcia *et al*, 2009). Thus, development of novel AMPK direct activators is the focus of intense efforts (Pang *et al*, 2008; Lee *et al*, 2011).

Androgens are the major drivers of PCa carcinogenesis/progression and androgen deprivation therapy (ADT) is an effective first-line therapy for advanced PCa. Despite good initial responses, most patients relapse within several years with a more aggressive castration-resistant PCa (CRPC), where androgen receptor (AR) reactivation occurs through a variety of mechanisms (Yuan *et al*, 2013). Hence targeting the AR remains a critical component of the current CRPC therapies, including the AR antagonist MDV3100, and the inhibitor of androgen synthesis abiraterone (Higano & Crawford, 2011). However, emergence of treatment resistance to these new drugs represents a major limitation (Yuan *et al*, 2013). Previous groups have shown AR-induced *de novo* lipogenesis, which is enhanced during the emergence of androgen independence to contribute to the survival/growth of CRPC cells (Swinnen *et al*, 1997; Ettinger *et al*, 2004). Since AMPK activation negatively regulates the expression/activity of lipogenic transcriptional factors (SREBPs, Sterol Regulatory Element-Binding Proteins) and the major lipogenesis and cholesterol synthesis enzymes, we reasoned that combinatorial regimens of AMPK activators and AR pathway inhibitors might result in a therapeutic benefit and overcome resistance to ADT.

In this study, we use a novel small molecule AMPK activator to investigate whether AMPK *per se* plays an anti-cancer role in PCa, and whether this can be monitored *in vivo* by PET imaging. We dissect the molecular underpinning mechanisms of AMPK mediated-growth inhibition and we seek to determine the effect of AMPK activation in CRPC and its combination with AR signaling inhibitors.

## Results

### MT 63–78 selectively activates AMPK in prostate cancer cells

**Figure 1:**
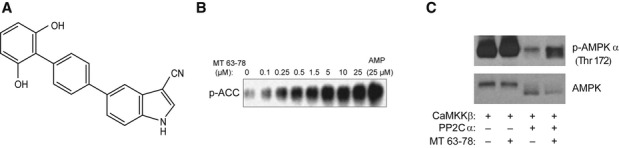
The novel small molecule MT 63–78 induces a direct activation of AMPK and prevents its dephosphorylation.
Molecular structure of MT 63–78 (MW = 326 Da).Dose-dependent phosphorylation of GST-ACC peptide (1–150) by human recombinant AMPK α1β1γ1, after 30-min incubation with MT 63–78. AMP (25 μM) was used as positive control.AMPK dephosphorylation assay, as described in Supplementary materials and methods. Recombinant AMPK α1β1γ1 (100 ng) was incubated with 100 ng of upstream kinase calcium/calmodulin-dependent protein kinase kinase-β (CaMKKβ). Phosphorylation of AMPK was then detected in the presence or absence of protein phosphatase 2C alpha (PP2Cα, 26 ng) and in the presence or absence of MT 63–78 (5 μM) using an antibody against the residue Thr172 on the α subunit. Source data are available for this figure.

**Figure 2:**
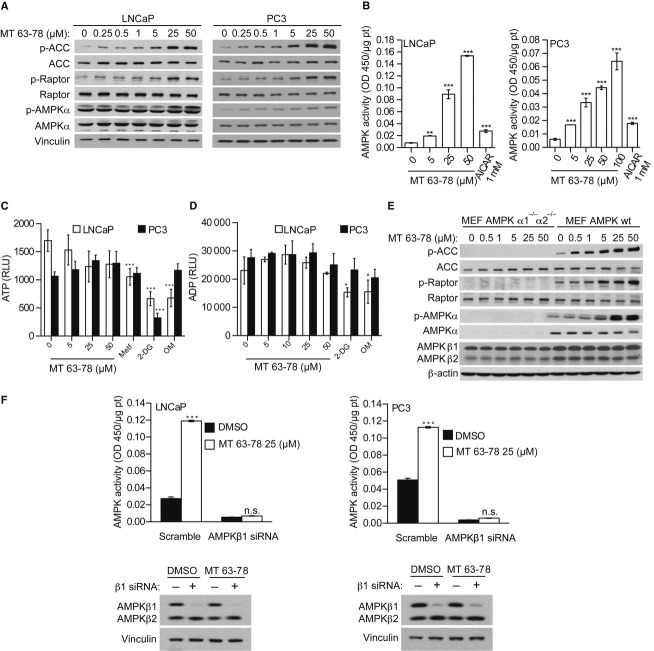
MT63–78 activates AMPK in PCa cells without altering the energy levels.
Dose-response activation of AMPK and phosphorylation of its direct targets Acetyl CoA Carboxylase (ACC) and Raptor in PCa cells.AMPK activity assay as described in Supplementary materials and methods. Results are expressed as normalized average ± s.d. of three independent samples. One-way ANOVA test, followed by Dunnett's post hoc test for multiple comparisons was performed and adjusted *P* values were calculated (LNCaP: **P = 0.0074 MT 5 μM versus DMSO; ***P < 0.0001 MT 25 μM versus DMSO; ***P < 0.0001 MT 50 μM versus DMSO; ***P = 0.0002 AICAR versus DMSO. PC3: ***P = 0.00022 MT 5 μM versus DMSO; ***P < 0.0001 MT 25 μM versus DMSO; ***P < 0.0001 MT 50 μM versus DMSO; ***P < 0.0001 MT 100 μM versus DMSO; ***P = 0.000173 AICAR versus DMSO). RLU = relative luminescence units..Measurment of ATP. 24-h incubation with metformin (Metf, 2.5 mM), 2-deoxyglucose (2-DG, 10 mM), and olygomycin (OM, 1 μM) was used as control. Results are expressed as means ± s.d. of three independent samples. One-way ANOVA test, followed by Dunnett's post hoc test for multiple comparisons was performed and adjusted *P* values were calculated (LNCaP: **P = 0.0093 Metf versus DMSO; ***P = 0.0002 2-DG versus DMSO; ***P = 0.0002 OM versus DMSO. PC3: ***P = 0.0002 2-DG versus DMSO).Measurment of ADP. Twelve hour incubation 2-deoxyglucose (2-DG, 10 mM), and olygomycin (OM, 1 μM) was used as control. Results are expressed as means ± s.d. of three independent samples. One-way ANOVA test, followed by Dunnett's post hoc test for multiple comparisons was performed and adjusted *P* values were calculated (LNCaP: *P = 0.0172 2-DG versus DMSO; *P = 0.0208 OM versus DMSO).Expression levels of phosphorylated ACC, Raptor, AMPK and their correspondent total forms in wild-type (wt) and AMPK α1^−/−^ and α2^−/−^ MEFs.AMPK activity in LNCaP and PC3 cells transfected with AMPK β1 subunit siRNA, following treatment with MT 63–78 (MT) or DMSO. Results are expressed as normalized average ± s.d. of three independent samples. One-way ANOVA test, followed by Bonferroni post hoc test for multiple comparisons was performed and adjusted *P* values were calculated. (LNCaP scramble: ***P = 1.92E-13 MT versus DMSO; LNCaP β1 siRNA *P* = 0.1179 MT versus DMSO. PC3 scramble: ***P < 0.0001 MT versus DMSO; PC3 β1 siRNA: *P* = 0.2455 MT versus DMSO. n.s. = non significant. Western blot analysis shows the rate of AMPK β1 silencing. Data information: Analyses in (A–F) were performed after 30-min incubation with MT 63–78, at indicated concentrations. Source data are available for this figure.

The small molecule MT 63–78 (now Debio 0930) was identified in a targeted screening using purified human recombinant AMPK α1β1γ1 (Fig 1A). Oral bioavailability and pharmacokinetic characterization of the compound is provided in Supplementary Fig 1A. Using a cell-free assay, we demonstrated that MT 63–78 allosterically activates recombinant AMPK in a dose-dependent manner (Fig 1B). In addition, MT 63–78, like AMP, inhibits AMPK dephosphorylation on Thr172 by protein phosphatase 2C alpha (Fig 1C). We tested whether MT 63–78 was able to activate AMPK in PCa cells, using androgen-dependent LNCaP (PTEN null) and androgen-independent PC3 (PTEN and p53 null) cell lines as models. We observed a dose-dependent phosphorylation of the two major AMPK targets Acetyl-CoA Carboxylase (ACC) on Ser79 and of Raptor on Ser792, after 30 min of treatment. A corresponding increase in Thr172 phosphorylation on the AMPK α subunit was also observed (Fig 2A). AMPK activity induced by MT 63–78 was significantly increased in both cell lines in a dose-dependent manner (EC_50_ = 25 μM) (Fig 2B). This increase was significantly stronger compared to treatment with the current available AMPK activator A-769662 (Abbott Laboratories) and AICAR (Supplementary Fig 1B). In contrast to metformin, 2-deoxyglucose, and oligomycin, addition of the compound did not cause any changes in intracellular ATP, ADP levels in LNCaP and PC3 cells, demonstrating that AMPK activation by MT 63–78 is not an indirect effect of increased energy stress (Fig 2C and D). We also confirmed these data in HepG2 cells by measuring ATP, ADP, and AMP levels using high-performance liquid chromatography (HPLC). Reduction in ATP levels and increased ADP, AMP levels were only observed at 200 μM of the compound, which is far beyond the concentrations used in this study (Supplementary Fig 2).

To evaluate the specificity of MT 63–78, AMPK α1^−/−^ and α2^−/−^ and wild-type (wt) mouse embryonic fibroblasts (MEFs) were incubated with MT 63–78 for 30 min. In contrast to wt MEFs, no phosphorylation of ACC and Raptor was observed in AMPK α1^−/−^ and α2^−/−^ MEFs (Fig 2E). We also screened MT 63–78 at 5 and 25 μM in cell-free assays against a panel of 93 protein kinases other than AMPK (Kinase Profiler, Millipore). Only 2 kinases (IGF-1R, Flt3) were slighltly activated at the highest concentration, whereas the majority of the kinases were not stimulated by either of the two concentrations, including the rat homolog of AMPK and the human members of AMPK-related kinase family (SIK, ARK5, MARK1). At 25 μM, six kinases were marginally inhibited, but only in one case (MST1) the inhibition was more than 50% (Supplementary Fig 3).

The compound exerts its effect by binding to the regulatory β subunit. To discriminate between the β1 and β2 subunits, we tested the *in vitro* activity of four different AMPK heterotrimeric complexes (α1β1γ1, α2β1γ1, α1β2γ1, α2β2γ1), following incubation with MT 63–78, A-769662, and AMP. We observed that both MT 63–78 and A-769662 show maximal AMPK activation in β1 subunit-containing heterotrimers (α1β1γ1, α2β1γ1) and a stronger effect for A-769662. At high doses MT 63–78 was also able to activate β2 subunit-containing heterotrimers, whereas A-769662 showed no activation (α1β2γ1) or low activation (α2β2γ1), suggesting a low-affinity binding of MT 63–78 to the β2 subunit, especially when β2 is complexed with the α2 subunit (Supplementary Fig 4A). However, using PCa cell models, we observed that β1 subunit knock down significantly reduces the basal activity of AMPK (80 and 92% for LNCaP and PC3, respectively) and totally abolishes its activation by MT 63–78 (Fig 2F). In contrast, in β2 subunit-silenced LNCaP and PC3 cells AMPK activity and ACC phosphorylation were still increased (although at lower extent) when cells were treated with MT 63–78 (Supplementary Fig 4B and C). This suggests that MT 63–78 activates AMPK mainly by binding the β1 subunit in a cell-based context.

Taken together, these results show that the small molecule MT 63–78 is a specific and potent tool for studying the effect of a direct activation of AMPK in PCa.

### AMPK activation by MT 63–78 is independent of the status of the tumor suppressor LKB1

**Figure 3:**
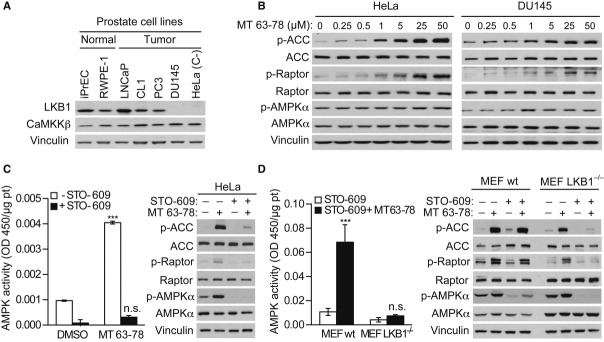
LKB1 is not essential for MT 63–78-mediated activation of AMPK.
LKB1 and CaMKKβ expression in PCa cells. HeLa cells were used as negative control.Dose-response activation of AMPK, phosphorylation of ACC and Raptor in LKB1 null HeLa (left) and DU145 (right) cells, after 30-min incubation with MT 63–78, at indicated concentrations.AMPK activity in HeLa cells treated with CaMKKβ inhibitor STO-609. HeLa cells were incubated with or w/o STO-609 (10 μg/ml) for 2.5 h prior to incubation with MT 63–78 (25 μM) for 30 min. Results are expressed as normalized average ± s.d. of three independent samples. One-way ANOVA test, followed by Bonferroni post hoc test for multiple comparisons was performed and adjusted *P* values were calculated (HeLa: ***P < 0.0001 MT versus DMSO; HeLa + STO-609: *P* = 0.0605 MT versus DMSO), n.s. = non significant. One sample for each condition was used for western blot analysis.AMPK activity and western blot analysis in wt and LKB1^−/−^ MEFs treated as in C. Results are expressed as normalized average ± s.d. of three independent samples. One-way ANOVA test, followed by Bonferroni post hoc test for multiple comparisons was performed and adjusted *P* values were calculated (MEF LKB1wt + STO-609: ***P < 0.0001 MT versus DMSO; MEF LKB1^−/−^ + STO-609: *P* > 0.09999 MT versus DMSO). n.s. = non significant. Source data are available for this figure.

Activation of AMPK requires phosphorylation of Thr172 mainly by LKB1 and CaMKKβ (Woods *et al*, 2003; Hawley *et al*, 2005). Both upstream kinases are expressed in normal prostate cells and in PCa cells, except for DU145 cells, which are LKB1 null (Fig 3A). We treated DU145 and HeLa cells (the model commonly used for LKB1 deficiency) with MT 63–78 and we observed a dose-dependent activation of AMPK in both cell lines (Fig 3B). HeLa as well as wt and LKB1^−/−^ MEFs were then exposed to the CaMKKβ inhibitor STO-609, prior to incubation with the compound. STO-609 almost completely abolished AMPK activation by MT 63–78 as well as the phosphorylation of AMPK downstream targets ACC and raptor in HeLa and LKB1^−/−^ MEFs, whereas STO-609 did not have any significant effect on wt MEFs (Fig 3C and D).

These results suggest that activation of AMPK in response to MT 63–78 is independent of LKB1 when CaMKKβ is functional, opening new perspectives for the treatment of LKB1-null tumors.

### Direct activation of AMPK inhibits androgen dependent and CRPC cell growth

**Figure 4:**
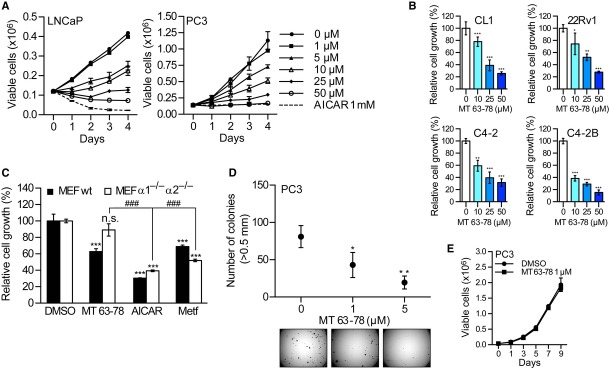
MT63–78 inhibits PCa cell growth and soft agar colony formation.
Growth curves of LNCaP and PC3 cells treated with MT 63–78 and AICAR for 4 days, at indicated concentrations.Relative growth of CL1, 22Rv1, C4-2, C4-2B cells, following 48-h treatment with MT 63–78. Results are expressed as percentage of cells compared to control (DMSO) ± s.d. of three independent samples. For CL1 experiments, six independent samples were used. One-way ANOVA test, followed by Dunnett's post hoc test for multiple comparisons was performed and adjusted *P* values were calculated (CL1: ***P = 0.00047 MT 10 μM versus DMSO; ***P < 1E-04 MT 25 μM versus DMSO; ***P < 1E-04 MT 50 μM versus DMSO. 22Rv1: *P = 0.0471 MT 10 μM versus DMSO; **P = 0.0021 MT 25 μM versus DMSO; ***P = 0.0004 MT 50 μM versus DMSO. C4-2: **P = 0.0011 MT 10 μM versus DMSO; ***P < 0.0001 MT 25 μM versus DMSO; ***P < 0.0001 MT 50 μM versus DMSO. C4-2B: ***P < 1E-08 MT 10 μM versus DMSO; ***P < 1E-08 MT 25 μM versus DMSO; ***P < 1E-08 MT 50 μM versus DMSO).Relative growth of wt and AMPK α1^−/−^ and α2^−/−^ MEFs, following 3-day treatment with MT 63–78 (25 μM), AICAR (1 mM), and metformin (Metf, 2.5 mM). Results are expressed as percentage of cells compared to control (DMSO) ± s.d. of three independent samples. One-way ANOVA test, followed by Tukey's post hoc test for multiple comparisons was performed and adjusted *P* values were calculated. (MEFs wt: ***P = 5.91E-07 MT versus DMSO; ***P = 3.75E-06 AICAR versus DMSO; ***P = 6.14E-06 Metf versus DMSO. AMPK α1^−/−^ and α2^−/−^ MEFs: *P* = 0.1373 MT versus DMSO non-significant (n.s.); ***P = 8.79E-09 AICAR versus DMSO; ***P = 4.27E-08 Metf versus DMSO; ^###^*P* = 1.41E-07 AICAR versus MT; ^###^*P* = 1.37E-06 Metf versus MT.Number of PC3 colonies in soft agar after 3-week treatment with 1 and 5 μM (positive control) MT 63–78. Results are expressed as number of colonies ± s.d. of three independent experiments. One-way ANOVA test, followed by Dunnett's post hoc test for multiple comparisons was performed and adjusted *P* values were calculated (*P = 0.027 MT 1 μM versus DMSO; **P = 0.0029 MT 5 μM versus DMSO). Photographs of colonies (10 ×  magnification) are shown.Growth curve of PC3 cells treated with 1 μM MT-63–78 or vehicle for 9 days. Results are expressed as number of viable cells ± s.d. of three independent samples. Two way ANOVA test, followed by SIDAK's multiple comparison test was performed. No significant difference was observed.

Growth of AR + and AR− LNCaP and PC3 cells was analyzed over 4 days, following the addition of MT 63–78. A dose-dependent decrease in cell number, concomitant to the activation of AMPK signaling was observed (Fig 4A). The growth inhibitory effect of MT 63–78 was also evaluated in several AR+ CRPC cell models (CL1, C4-2, and 22Rv1), as well as in the C4-2B subline derived from bone metastasis. Forty-eight hours incubation with MT 63–78 significantly reduced cell growth in all CRPC cell models (Fig 4B). High concentrations of the compound (50 μM) exerted a more moderate effect in immortalized non-transformed iPrEC and RWPE-1 cells, and low concentrations (5 μM) do not affect RWPE-1 cells, while still inhibiting PCa cell growth. These data suggest that the compound mostly affects cancer cell growth (Supplementary Fig 5A and B). We compared the efficacy of MT 63–78 and A-769662 in inhibiting AR sensitive and CRPC PCa cell growth and showed that MT 63–78 has 16–40 times higher potency than A-769662 (Supplementary Fig 5C). In addition, the growth inhibitory effect of MT 63–78 was not limited to PCa cells, and included LKB1-null A549 and BRAF mutated (V600E) KTC-1 cells (Supplementary Fig 6).

Using AMPK α1^−/−^ and α2^−/−^ MEFs, we confirmed that MT 63–78-mediated inhibition of cell growth is strictly dependent on the functional activity of AMPK (Fig 4C) in contrast to AICAR and metformin, as previously shown by other groups (Ben Sahra *et al*, 2008; Park *et al*, 2009; Gonzalez-Girones *et al*, 2013). At concentrations that do not affect cell number (1 μM), MT 63–78 still inhibits anchorage-independent growth (Fig 4D and E).

### MT 63–78 induces mitotic arrest and apoptosis

**Figure 5:**
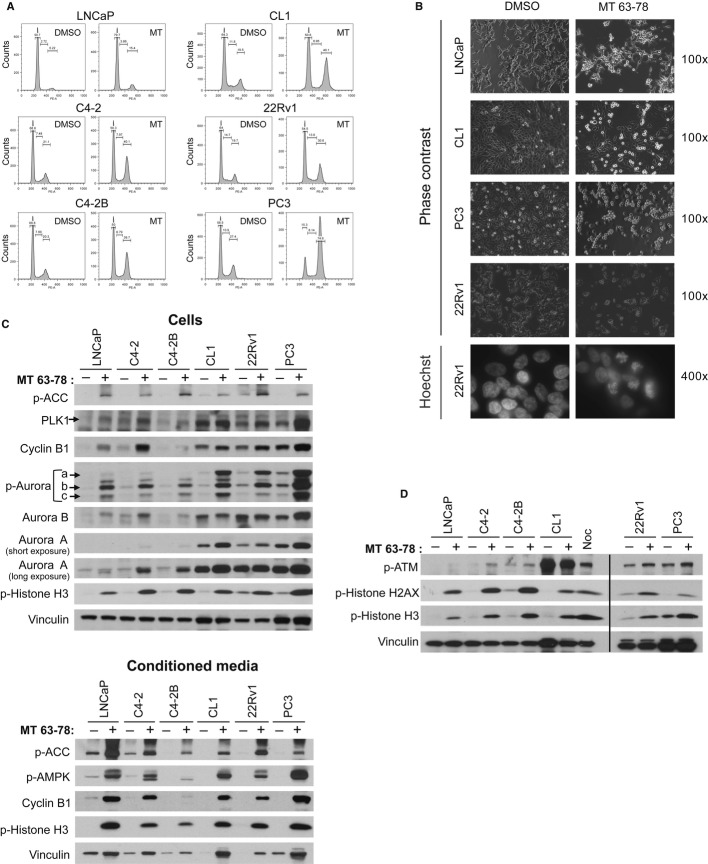
MT63–78 induces mitotic arrest in PCa cells.
Cell cycle analysis in LNCaP and CRPC cells treated for 24 h with 25 μM MT 63–78 (MT). Percentage of cells in G1, S, and G2/M phases is indicated.Phase contrast and immunofluorescence (Hoechst staining) images of LNCaP and CRPC cells, following 24-h treatment with MT 63–78 (25 μM). Magnification is indicated.Western blot analysis of mitotic proteins in PCa cell lysates and conditioned media, following 24-h treatment with 25 μM MT 63–78.Western blot analysis of DNA damage signaling in PCa cell lysates, following 24-h treatment with 25 μM MT 63–78. Treatment with 100 ng/ml Nocodazole (Noc) for 14 h was used as positive control. Source data are available for this figure.

**Figure 6:**
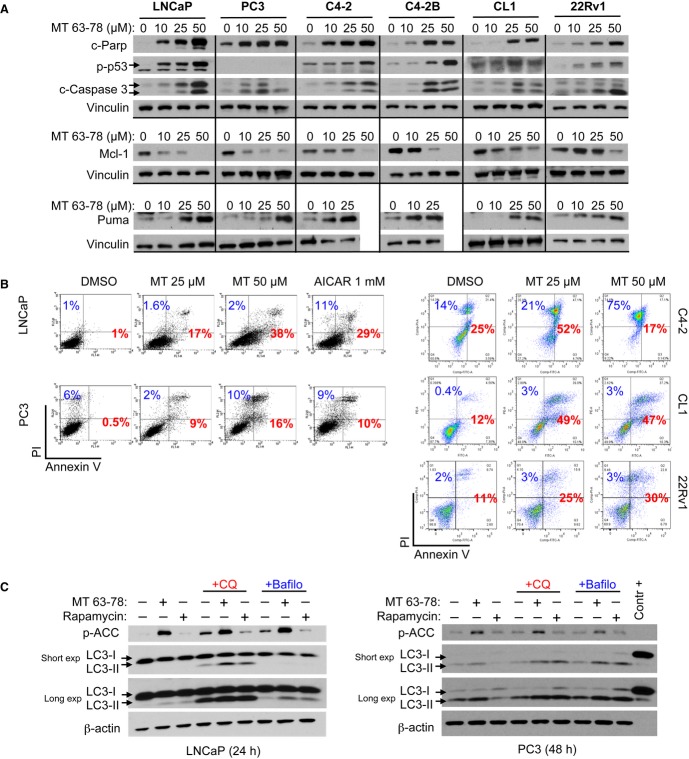
MT63–78 induces apoptosis in PCa cells.
Western blot analysis of apoptosis markers, pro-apoptotic, and anti-apoptotic proteins in PCa cells lysates, after 24-h treatment with MT 63–78.Flow cytometry plots of Annexin V/PI assay in PCa cells, following 48-h treatment with MT 63–78. LNCaP treatment with AICAR was used as positive control. Percentage of apoptotic cells (early and late apoptosis) is indicated in red color. Percentage of necrotic cells is depicted in blue.Western blot analysis of autophagic flux in LNCaP and PC3 cells, following treatement with MT 63–78 (25 μM). Lysosomal inhibitor Cloroquine (CQ, 10 μM) was added together with MT 63–78, whereas Bafilomycin A1 (Bafilo, 400 nM) was added 6 h before harvesting. Treatment with rapamycin (10 nM) was used as positive control. Contr+= 10  l of positive control for anti-LC3 antibody (PM036-PN). Source data are available for this figure.

Cell cycle analysis using propidium iodide (PI) showed that 24-h treatment with MT 63–78 induces a significant enrichement in the G_2_/M population in both androgen sensitive and CRPC cell models (Fig 5A). G2-M arrest was particularly dramatic in the p53 null PC3 cells, suggesting a p53-independent effect. In contrast, in LNCaP cells, G2/M arrest was more evident after 48-h treatment (Supplementary Fig 7A). These finding were also confirmed in other tumor models (Supplementary Fig 7A). In depth morphological and molecular characterization indicated that MT 63–78 induces arrest in M phase. We observed accumulation of rounded and detached cells, and chromosome condensation in pro-metaphase (Fig 5B). Western blot analysis showed increased phosphorylation of histone H3 (marker of mitosis) in treated cells and accumulation of the mitotic regulator cyclin B1 (Fig 5C), suggesting that arrest takes place before anaphase when most of cyclin B1 is degradated (Clute & Pines, 1999; Chang *et al*, 2003), most likely during pro-metaphase. In contrast to nocodazole treatment, no significant changes in cdc2 expression were observed (Supplementary Fig 7B). Moreover, the viable cells recovered from the conditioned media of MT 63–78 treated dishes showed high levels of cyclin B1 and positivity for phospho-histone H3, in keeping with the known looser attachment of cells to the substrate during mitotic rounding (Fig 5C). Consistent with the accumulation of cyclin B1, we observed accumulation and/or increased activation of key mitotic kinases such as Aurora kinases A, B, and Polo-like kinase 1 (PLK1) (Fig 5C). Evidence is now accumulating that a fine-tuned spatial and temporal activation of AMPK at the mitotic apparatus is required for correct spinde assembly/orientation, chromosal segregation, and proper cell division (Lee *et al*, 2007; Vazquez-Martin *et al*, 2009a,2009b; Thaiparambil *et al*, 2012). Thus, our results suggest that MT 63–78-induced sustained activation of AMPK alters these dynamics and does not allow cells to undergo a correct cytockinesis, as previously shown with metformin (Vazquez-Martin *et al*, 2009b). Recently, it has been demonstrated that drug-induced prolonged M phase arrest leads to telomere deprotection and activation of DNA damage response with ATM activation and increased phosphorylation of histone H2AX (γ-H2AX), which can also be found in mitotic arrested cells undergoing apoptosis (Rogakou *et al*, 2000; Hayashi *et al*, 2012). Markedly increased phosphorylation of histone H2AX was indeed observed upon mitotic block induced by MT 63–78 (Fig 5D). Mitotic arrested PCa cells undergo cell death via activation of the intrinsic apoptotic pathways. We observed p53 activation, caspase-3, parp cleavage, and outer membrane permeabilization in all the models assessed, including the p53 null PC3 cells. The extent of apoptosis in PC3 cells was however reduced with respect to p53 wt cells (Fig 6B). To identify the underlying molecular mechanisms, we evaluated the expression of the most critical Bcl-2 family members. MT 63–78 treatment induces reduction of anti-apoptotic Mcl-1 in concert with accumulation of the pro-apoptotic BH3-only protein Puma (Fig 6A) in all PCa cells. In contrast, Noxa accumulation was only observed in 22Rv1 cells. Bcl-2 levels were moderately reduced in C4-2, C4-2B, CL1, and 22Rv1 cells. Pro-survival XIAP protein was also found reduced in C4-2 and C4-2B cells (Supplementary Fig 8).

The induction of autophagic response upon AMPK activation has been previously reported (Hoyer-Hansen & Jaattela, 2007; Egan *et al*, 2011). However, only a modest effect of MT 63–78 on authophagy flux was observed in LNCaP and PC3 cells following 24-and 48-h treatment, respectively (Fig 6C).

### Repression of the mTORC1 pathway does not account for the growth inhibitory effect of AMPK direct activation

**Figure 7:**
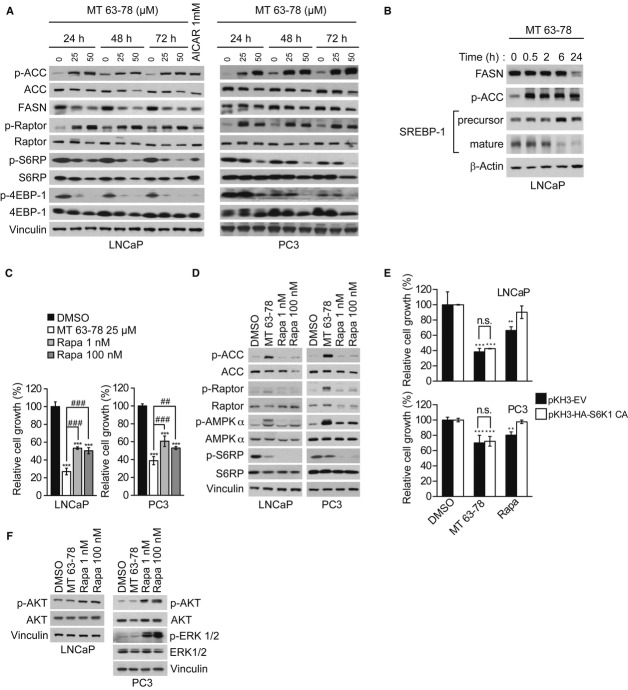
Inhibition of mTORC1 is only partially responsible for the anti-growth effects of MT 63–78.
Western blot analysis of lipogenic and mTORC1 pathways, after incubation with MT 63–78 for the indicated time points. ACC = Acetyl-CoA carboxylase, FASN = fatty acid synthase, S6RP = S6 ribosomal protein, 4EBP-1 = 4 element binding protein 1. AICAR (1 mM) was used as positive control.Expression levels of precursor and mature forms of SREBP-1, following MT 63–78 (25 μM) treatment for the indicated time points.Relative growth of LNCaP and PC3 cells after 3-day treatment with MT 63–78 (MT) or rapamycin (Rapa). Results are expressed as mean ± s.d. of three independent samples. One-way ANOVA test, followed by Tukey's post hoc test for multiple comparisons was performed and adjusted *P* values were calculated (LNCaP: ***P = 5.11E-08 MT versus DMSO; ***P = 1.45E-06 Rapa 1 nM versus DMSO; ***P = 2.47E-06 Rapa 100 nM versus DMSO; ^###^*P* = 0.000152 MT versus Rapa 1 nM; ^###^*P* = 0.000393 MT versus Rapa 100 nM. PC3: ***P = 1.63E-07 MT versus DMSO; ***P = 6.09E-06 Rapa 1 nM versus DMSO; ***P = 1.53E-06 Rapa 100 nM versus DMSO; ^###^*P* = 0.000438 MT versus Rapa 1 nM; ^##^*P* = 0.00763 MT versus Rapa 100 nM).Expression levels of phosphorylated ACC, Raptor, AMPK, S6RP, and their total forms after 3-day treatment with MT 63–78 (25 μM) and rapamycin (Rapa).Relative growth of LNCaP and PC3 cells transfected with empy vector (pKH3-EV) or with a construct containing constitutively active, rapamycin insensitive, S6 kinase 1 (pKH3-HA-S6K1 CA). Count was performed 3 days after incubation with MT 63–78 (25 μM) or rapamycin (1 nM). Drugs were added 12 h post-transfection. Results are expressed as a percentage of control (DMSO-treated cells) ± s.d. of three independent samples. One-way ANOVA test, followed by Tukey's post hoc test for multiple comparisons was performed and adjusted *P* values were calculated (LNCaP-pKH3-EV: ***P < 0.0001 MT versus DMSO; **P = 0.0089 Rapa versus DMSO. LNCaP-pKH3-HA-S6K1 CA: ***P = 0.0008 MT versus DMSO. PC3-pKH3-EV: ***P = 0.0003 MT versus DMSO; **P = 0.0088 Rapa versus DMSO. PC3-pKH3-HA-S6K1 CA: ***P = 0.0005 MT versus DMSO). n.s. = non significant.Feedback activation of Akt and p42/p44 MAPK after 72-h incubation with MT 63–78 (25 μM) or rapamycin (Rapa). Source data are available for this figure.

We next turned our attention on the two AMPK downstream pathways known to play a dominant role in the control of PCa cell growth/proliferation and survival. We, therefore, assessed the relative contribution of mTORC1 and lipogenesis in mediating PCa growth inhibition, following AMPK direct activation. AMPK inhibits the mTORC1 pathway through phosphorylation of the TSC2 complex and Raptor, whereas it affects *de novo* FA and cholesterol synthesis through phosphorylation of the rate limiting enzymes ACC and 3-hydroxy-3-methyl-glutaryl-CoA reductase (HMGCR) and of the transcriptional regulators SREBPs, as well as via transcriptional repression of lipogenic genes (Clarke & Hardie, 1990; Zhou *et al*, 2001). Both the mTORC1 (assessed by phosphorylation of Raptor, S6RP, and 4EBP-1) and the lipogenic pathways were inhibited by MT 63–78. Increased phosphorylation of ACC was observed in LNCaP and PC3 cells while expression of ACC and FA synthase (FASN) was also significantly downregulated (Fig 7A). Levels of the mature form of SREBP-1, responsible for the transcription of ACC and FASN, were significantly reduced (Fig 7B). FASN activity was repressed as well (Supplementary Fig 9A). However, when we compared MT 63–78 to the mTORC1 inhibitor rapamycin, we observed that the former caused a greater inhibition of PCa cell growth despite a more modest reduction of S6RP phosphorylation (Fig 7C and D). Moreover, re-activation of the mTORC1 pathway, by transient transfection of constitutively active and rapamycin insensitive S6 Kinase 1, was not sufficient to rescue MT 63–78 mediated-cell growth inhibition (LNCaP, *P* = 0.9950; PC3, *P* = 0.9740, one-way anova followed by Tukey's post hoc test) (Fig 7E).

Taken together, these results suggest that the blockade of the mTORC1 pathway is not the principal mechanism responsible for growth inhibition mediated by direct AMPK activation. In contrast to rapamycin treatment, no increase in Akt and MAPK phosphorylation was detected in LNCaP and PC3 cells treated with MT 63–78, suggesting that direct AMPK activation does not induce undesired mitogenic feedback loops (Fig 7F).

### Blockade of de novo lipogenesis is the key mechanism of AMPK-mediated growth inhibition

**Figure 8:**
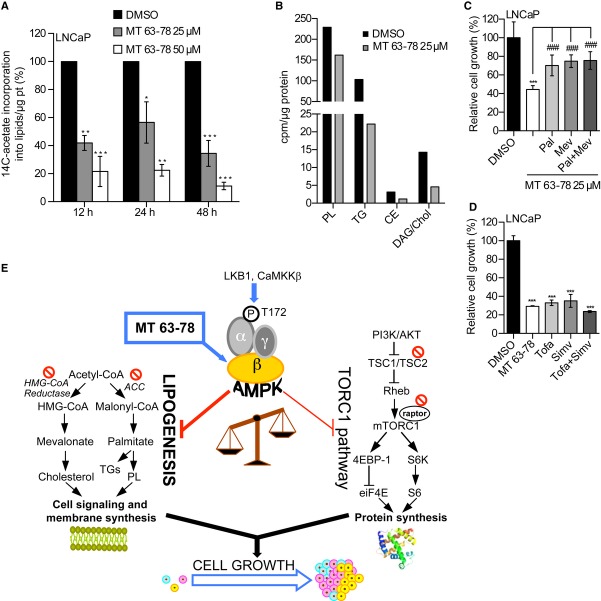
The anti-growth effects of MT 63–78 are mediated by inhibition of lipogenesis.
Incorporation of ^14^C-acetate into lipids, following treatment with MT 63–78 (MT). Results are expressed as a percentage of ^14^C-acetate incorporation into lipids compared to control, normalized to protein content. Mean values ± s.d. of three independent experiments are shown. One-way ANOVA test, followed by Dunnett's post hoc test for multiple comparisons was performed and adjusted *P* values were calculated (LNCaP-12 h: **P = 0.0018 MT 25 μM versus DMSO; ***P = 0.0004 MT 50 μM versus DMSO. LNCaP-24 h: *P = 0.0230 MT 25 μM versus DMSO; **P = 0.0014 MT 50 μM versus DMSO; LNCaP-48 h: ***P = 0.0003 MT 25 μM versus DMSO; ***P = 5.04E-05 MT 50 μM versus DMSO).^14^C-acetate incorporation in different lipid species. Data are expressed in cpm normalized to protein content. PL = Phospholipids, TG = Triacylglycerols, CE = Cholesterol esters, Chol = Cholesterol, DAG = Diacylglycerols.Relative cell growth after 3-day treatment with MT 63–78 (MT) alone or in combination with 75 μM palmitate (Pal) and/or 100 μM mevalonate (Mev). Results are expressed as mean ± s.d. of five independent samples. One-way ANOVA test, followed by Tukey's post hoc test for multiple comparisons was performed and adjusted *P* values were calculated (***P = 2.4E-09 MT versus DMSO; ^###^*P* = 0.000573 MT+Pal versus MT; ^###^*P* = 6.96E-05 MT+Mev versus MT; ^###^*P* = 4.97E-05 MT+Pal+Mev versus MT).Relative cell growth after 3-day treatment with MT 63–78 (25 μM), tofa (10 μg/ml), simvastatin (Simv, 10 μM), and their combination. Results are expressed as mean ± s.d. of three independent samples. One-way ANOVA test, followed by Tukey's post hoc test for multiple comparisons was performed and adjusted *P* values were calculated (***P = 1.13E-08 MT versus DMSO; ***P = 2.26E-08 Tofa versus DMSO; ***P = 3.17E-08 Simv versus DMSO; ***P = 4.82E-09 Tofa+Simv versus DMSO).Working model for MT 63–78. LKB1 = liver kinase B1, CaMKKβ = calcium/calmodulin-dependent protein kinase kinase-β, ACC = Acetyl-CoA carboxylase, HMG-CoA = 3-Hydroxy-3-methyl-glutaryl-CoA, PI3K = phosphatidylinositol 3-kinase, TSC1/TSC2 = tuberous sclerosis complex 1/2, Rheb = Ras homolog enriched in brain, mTORC1 =  mammalian target of rapamycin complex 1, 4EBP-1 = 4E-binding protein 1, S6K = S6 kinase, eiF4E = Eukaryotic translation initiation factor 4E, PL = Phospholipids, TG = Triacylglycerols. Color code: blue = activation; red = inhibition.

To corroborate the inhibitory effect of MT 63–78 on lipogenesis, we measured ^14^C-acetate incorporation into lipids extracted from LNCaP cells and observed a dose-dependent reduction in ^14^C-acetate incorporation at all the time points analyzed (Fig 8A). Comparable results were observed in PC3 cells (Supplementary Fig 9B). As expected, most of the radioactivity was incorporated into the phospholipid fraction, with lower amounts in triacylglycerols and other neutral lipids. AMPK activation affects primarily *de novo* production of phospholipids but also significantly decreases the production of neutral lipids (triacylglycerides, cholesterol, cholestererol esters, and diacylglyclerols) (Fig 8B). We then determined whether the repression of lipogenesis accounts for MT 63–78-mediated cell growth inhibition. Addition of exogenous mevalonate and palmitate, the final products of HMGCR and FASN, rescued partially but significantly LNCaP cell growth. Surprisingly, no additive effect was observed with palmitate and mevalonate together (Fig 8C). Of note, neither palmitate nor mevalonate showed an effect on growth of untreated LNCaP cells (Supplementary Fig 9C). Moreover, the addition of the ACC inhibitor TOFA and HMGCR inhibitor simvastatin, alone or combined, inhibited cell growth similarly to MT 63–78 (Fig 8D). These results suggest that the growth inhibitory effect of direct AMPK activation in PCa cells is mediated in large part through inhibition of FA and cholesterol synthesis (Fig 8E).

### MT 63–78 enhances the growth inhibitory effect of AR signaling inhibitors

**Figure 9:**
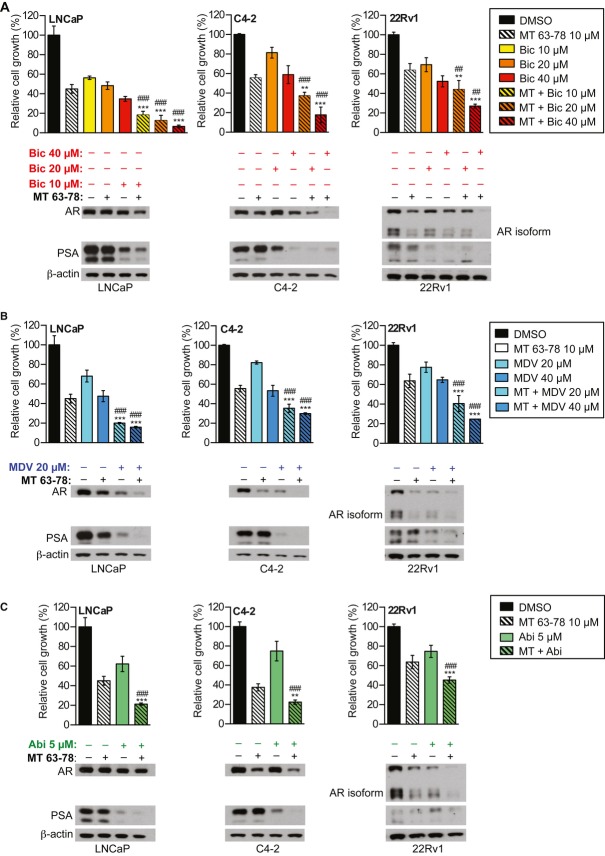
MT63–78 enhances the growth inhibitory effect of ADT.
Relative growth of LNCaP and CRPC cells C4-2 and 22Rv1, following 3-day treatment with MT 63–78 (MT), AR antagonist bicalutamide (Bic), and combined treatment (MT+Bic). Results are expressed as mean ± s.d. of three independent samples. One-way ANOVA test, followed by Tukey's post hoc test for multiple comparisons was performed and adjusted *P* values were calculated (**P < 0.01, ***P < 0.001 MT versus MT+Bic; ^##^*P* < 0.01, ^###^*P* < 0.001 Bic versus MT+Bic). Exact *P* values for each comparison are reported in Supplementary materials and methods. Expression levels of AR and PSA following single or combined treatments are shown under the bar graphs.Relative growth of LNCaP and CRPC cells C4-2 and 22Rv1, following 3-day treatment with MT 63–78 (MT), AR antagonist MDV3100 (MDV), and combined treatment (MT+MDV). Results are expressed as mean ± s.d. of three independent samples. One-way ANOVA test, followed by Tukey's post hoc test for multiple comparisons was performed and adjusted *P* values were calculated (***P < 0.001 MT versus MT+MDV; ^###^*P* < 0.001 MDV versus MT+MDV). Exact *P* values for each comparison are reported in Supplementary materials and methods. Expression levels of AR and PSA following single or combined treatments are shown under the bar graphs.Relative growth of LNCaP and CRPC cells C4-2 and 22Rv1, following 3-day treatment with MT 63–78 (MT), CYP17A1 inhibitor abiraterone (Abi) and combined treatment (MT+Abi). Results are expressed as mean ± s.d. of three independent samples. One-way ANOVA test, followed by Tukey's post hoc test for multiple comparisons was performed and adjusted *P* values were calculated (**P < 0.01, ***P < 0.001 MT versus MT+Abi; ^###^*P* < 0.001 Abi versus MT+Abi). Exact *P* values for each comparison are reported in Supplementary materials and methods. Expression levels of AR and PSA following single or combined treatments are shown under the bar graphs.

Enhanced lipogenesis is associated with the emergence of CRPC phenotype. The current standard of care for advanced CRPC is based on the administration of AR antagonists/inhibitors of androgen synthesis. Recently, MDV3100 and abiraterone have been FDA approved for the treatment of naïve or post-docetaxel advanced CRPC. However, emergence of treatment resistance to these new drugs represents a major limitation. Thus, we tested whether a combination treatment of MT 63–78 with AR antagonists/androgen synthesis inhibitors could result in increased therapeutic response. MT 63–78 significantly enhances the growth inhibitory effect of AR antagonists (bicalutamide and MDV3100) and abiraterone in both LNCaP and CRPC cells (Fig 9A–C, Supplementary Fig 10). MT 63–78 reduces AR levels and combinatorial treatments result in further reduction of AR and PSA expression levels when compared to single treatments (Fig 9A–C).

### Direct activation of AMPK inhibits tumor growth in xenograft models

**Figure 10:**
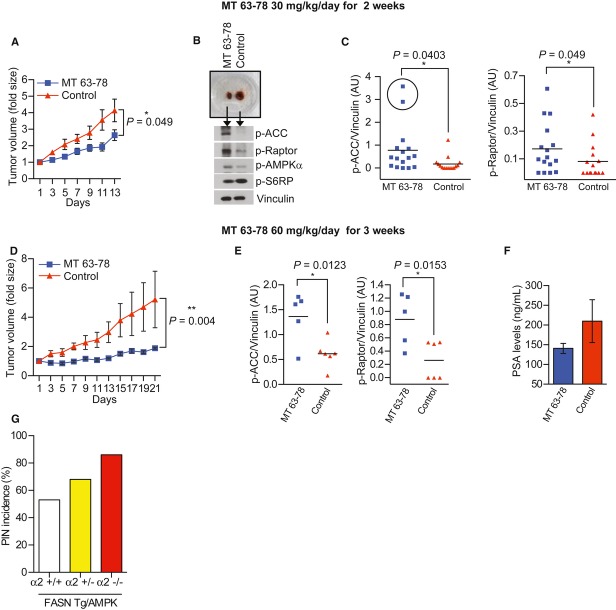
In *vivo* effects of AMPK activation.
Average tumor volume of LNCaP xenografts during 14-day treatment with MT 63–78 (30 mg/kg, i.p.). Experiments were performed twice for a total number of 17 mice under treatment and 15 controls. Results are expressed as n fold the mean initial volume (equal to 1) ± s.e.m. (*P = 0.049, end of treatment, Unpaired *t*-test).Representative image of treated and control mice (with similar pre-treatment tumor volume) and their excised tumors at sacrifice. Correspondent western blot analysis of key proteins belonging to lipogenic and mTORC1 pathways is shown.Densitometric analysis of phosphorylated ACC and Raptor in tumor homogenates of all treated mice (*n* = 16) and controls (*n* = 15). One treated mouse was not included in the analysis because frozen material was not available. Highlighted (black circle) are the two tumors with the highest ACC phosphorylation and volume reduction. Results are expressed in arbitrary units, after normalization to Vinculin. Unpaired *t*-test and Wilcoxon Mann–Whitney *t*-test were performed. Significant P values are reported on the graphs.Average tumor volume of LNCaP xenografts during 21-day treatment with MT 63–78 (60 mg/kg, i.p., five mice) and vehicle (six mice). Results are expressed as n fold the mean initial volume (equal to 1) ± s.e.m. (**P = 0.004, end of treatment, Wilcoxon Mann–Whitney test). One mouse from treated group was removed from the study since biochemical analysis showed that the drug for unknown reasons did not penentrate in the tumor.Densitometric analysis of phosphorylated ACC and Raptor performed as in C. Unpaired *t*-tests were performed. Significant P values are reported on the graphs.Serum PSA levels measured at the end of the 21-day treatment with MT 63–78 (60 mg/kg, i.p.) in five treated and five control mice. Results are expressed as mean ± s.e.m. ELISA was performed twice (each sample in duplicate) with similar results. A representative experiment is shown. Serum from one control mouse was not available. Unpaired *t*-test was performed (*P* = 0.27).PIN incidence in FASN-Tg α2^+/+^ (*n* = 34), FASN-Tg/AMPK α2^+/−^ (*n* = 28), FASN-Tg/AMPK α2^−/−^ (*n* = 7) age-matched mice. Logistic regression *P* = 0.077, with post hoc calculated power of 0.44. Source data are available for this figure.

We next sought to determine if the direct activation of AMPK could inhibit tumor growth in an *in vivo* model. Thirty-two nude mice bearing LNCaP tumors measuring 50–100 mm^3^ were treated intraperitoneally (i.p.) with MT 63–78 at 30 mg/kg daily (*n* = 17) or with 5% hydroxypropyl beta-cyclodextrine as control (*n* = 15) for 14 days. MT 63–78 led to a 33% inhibition of tumor growth (*P* = 0.049, Fig 10A). Consistent with our *in vitro* findings, MT 63–78 promotes AMPK activation in xenograft tumors and inhibits the lipogenesis and mTORC1 pathways, as assessed by increased ACC and Raptor phosphorylation, respectively (Fig 10B and C). Importantly, the highest levels of ACC phosphorylation were found in the two mice showing the most significant tumor volume reduction (Fig. 10C, black circles). Despite the fact that MT 63–78 reduced the glycemia and improved glucose tolerance in animal models of obesity and insulin resistance (Supplementary Fig 11A and B), no effect on weight, serum glucose and triglycerides levels were observed in non-obese nude mice maintained on normal chow diet, following MT 63–78 treatment (Supplementary Fig 11C and D). Prolonged treatment (21 days) with higher dose of MT 63–78 (60 mg/kg) resulted in a more robust inhibition of LNCaP xenograft growth (*P* = 0.004, end of treatment) (Fig 10D) as well as increased pharmacodinamic markers p-ACC and p-Raptor in treated tumors compared to controls (Fig 10E). Slight increase of apoptotic marker cleaved-parp was observed in the treated tumors (Supplementary Fig 11E). Serum PSA levels were measured at the end of the treatment. Mean PSA level in treated group was lower than in control, although the difference did not reach statistical significance (Fig 10F).

### Loss of AMPK catalytic activity fosters PCa development

Since our results demonstrated that AMPK direct activation inhibits PCa tumor growth, we hypothesized that the loss of AMPK catalytic alleles and, as a result, activity, would favor PCa development. Thus, we crossed prostate specific FASN transgenic (FASN-Tg) mice, which develop age-dependent prostate epithelial neoplasia (PIN) (Migita *et al*, 2009) with AMPK α2^−/−^ mice. At the age of 13–16 months, 34 FASN-Tg/AMPK α2^+/+^, 28 FASN-Tg/AMPK α2^+/−^, 7 FASN-Tg/AMPK α2^−/−^ mice were sacrificed and the three prostate lobes (anterior, ventral, dorso-lateral) were collected for histological examination. Despite the expression of AMPK α1 catalytic isoform in the murine prostate, we observed increased incidence of PIN in FASN-Tg/AMPK α2^−/−^, suggesting that loss of α2 subunit is sufficient to enhance FASN-induced tumorigenesis (Fig 10G). The odds of PIN occurrence increase with the number of knockout alleles (logistic regression *P* = 0.077, with post hoc calculated power of 0.44).

### *In vivo* assessment of AMPK activation using ^11^C-acetate positron emission tomography

**Figure 11:**
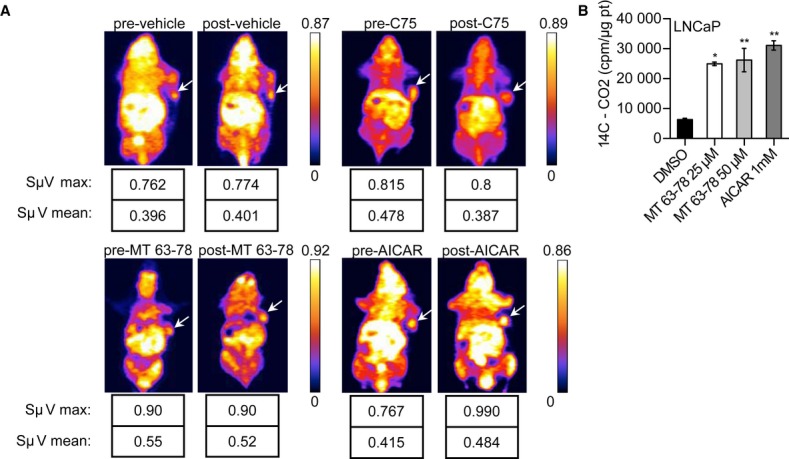
^11^C-acetate PET in LNCaP xenograft treated with AMPK activators.
^11^C-acetate micro-PET imaging before and after i.p. treatment with vehicle (5% hydroxypropyl beta-cyclodextrine) or AMPK activators MT 63–78 and AICAR. FASN inhibitor C75 was used as positive control. Fire LUT images of tumor-bearing mice, after 15 min of i.v. injection of ^11^C-acetate, are shown. Arrows mark tumor location. Maximal and mean standardized uptake volumes (SUVs) are indicated.^14^C-acetate oxidation in LNCaP cells, following 6-h treatment with MT 63–78 (MT) or AICAR. ^14^C-CO_2_ was measured in cpm and normalized to protein content, after background subtraction. Results are expressed as mean ± s.d. of three independent samples. One-way ANOVA test, followed by Dunnett's post hoc test for multiple comparisons was performed and adjusted *P* values were calculated (*P = 0.0109 MT 25 μM versus DMSO; **P = 0.0051 MT 50 μM versus DMSO; **P = 0.0016 AICAR versus DMSO).

Since acetate is a precursor for FA and cholesterol synthesis, we performed Positron Emission Tomography (PET) to investigate whether inhibition of lipogenesis by MT 63–78 was reflected by a reduction of ^11^C-acetate uptake by tumor xenografts. Three mice treated with MT 63–78 (30 mg/kg) or vehicle, 1 mouse treated with AICAR (400 mg/kg), and 1 mouse treated with FASN inhibitor C75 (30 mg/kg) were imaged with ^11^C-acetate PET before and after 24-h treatment with compounds. The FASN inhibitor C75 caused a 20% reduction in the uptake, whereas AICAR induced a slight increase (16%). No difference was observed with MT 63–78 (Fig 11A). These results are consistent with the role of activated AMPK in promoting nutrients uptake, their catabolism for ATP production, and mitochondrial biogenesis (Steinberg & Kemp, 2009). To investigate whether reduction in ^11^C-acetate uptake for lipids synthesis may be compensated for by increased uptake for its oxidation, we incubated LNCaP cells, previously treated with MT 63–78 and AICAR for 4 h, with ^14^C-acetate (2uCi) for 2 h. At the end of the treatment, the production of ^14^C-CO_2_ was measured. A significant increase in ^14^C-CO_2_ was observed, confirming that AMPK activation promotes acetate oxidation in the TCA (Fig 11B). No increase in ^14^C-CO_2_ production was observed after 30 min of AMPK activation, consistent with our previous data showing no difference in ATP levels at that time point (Fig 2C).

## Discussion

The master metabolic regulator AMPK has recently emerged as a potential therapeutic target for cancer treatment. AMPK activators such as AICAR and metformin have been shown to inhibit PCa growth in culture, in xenografts, and in genetically engineered mouse models (GEMMs) (Xiang *et al*, 2004; Huang *et al*, 2008; Zakikhani *et al*, 2008). Moreover, epidemiologic data including biguanides, physical activity, caloric restriction, and increased adiponectin levels have been reported to both activate AMPK and lower cancer risk, including PCa (Jiang *et al*, 2008; McTiernan, 2008; Li *et al*, 2010). However, AMPK-independent effects of drugs tested to date (Ben Sahra *et al*, 2008, 2011; Moreno *et al*, 2008; Garcia-Garcia *et al*, 2009; Kalender *et al*, 2010; Santidrian *et al*, 2010) prevented investigators to unequivocally determine whether AMPK activation was the necessary and sufficient mechanism of inhibiting carcinogenesis. In search for an adequate tool to address this unresolved question, we characterized and utilized in our experiments the novel AMPK direct activator MT 63–78. This new tool allowed us demonstrating that AMPK activation itself is necessary and sufficient for the suppression of PCa cell growth, in both androgen-dependent and CRPC models. Importantly, the growth inhibitory response to MT 63–78 was not affected by the status of the upstream AMPK-activating kinase LKB1. Since 30% of somatic NSCLCs harbor *LKB1* inactivating mutations, often associated with *KRAS* mutations (Mahoney *et al*, 2009; Shackelford & Shaw, 2009), our results open new therapeutic opportunities for LKB1 deficient and LKB1 deficient/KRAS activated tumors.

The use of a complementary approach with a mouse model of genetic ablation of α2 subunit in the context of FASN overexpression underscored the crucial role of AMPK pathway in PCa progression, previously demonstrated for other tumor models (Huang *et al*, 2008; Faubert *et al*, 2013) and further supports the potential of targeting AMPK axis for PCa treatment.

Cell cycle analysis shows that MT 63–78 induces mitotic arrest with accumulation/activation of cyclin B1, Aurora kinases A and B, and PLK1, involved in mitotic spindle assembly, chromosome segregation and cytokinesis. These results are in line with literature observations highlighting the role of AMPK as cell cycle regulator, beyond its metabolic activity (Lee *et al*, 2007; Vazquez-Martin *et al*, 2009a,b2009b,c2009c; Banko *et al*, 2011). From these studies it has emerged that a fine-tuned biphasic activation of AMPK is required for proper mitotic progression, whereas any alteration of AMPK expression or function (obtained by pharmacological sustained activation or AMPK depletion) alters its spatial and temporal regulation, resulting in microtubule misalignement, spindle misorientation, abnormal chromosome segregation followed by mitotic catastrophe and polyploidy (Vazquez-Martin *et al*, 2009b; Banko *et al*, 2011; Thaiparambil *et al*, 2012). Our data are also supported by recent experiments done in our laboratory, using synchronized HeLa cells as model, which show that increased *de novo* fatty acid synthesis concomitant to reduced AMPK activity and phosphorylation of ACC is required for cytokinesis initiation (Natalia Scaglia, unpublished results). In this view, AMPK orchestrates the completion of mitosis by coordinately regulating mitotic spindle assembly, chromosomal segregation, and cytoskeleton rearrangement with membrane synthesis. Thus, MT 63–78 induced mitotic arrest could be ascribed to both a persitent AMPK-mediated inhibition of *de novo* FA synthesis (metabolic role) as well as to mitotic spindle assembly/chromosome segregation abnormalities (non-metabolic role). Moreover, we observed induction of DNA damage signaling following prolonged mitotic arrest, which potentially serves as a mitotic duration checkpoint, responsible for eliminating cells that fail to progress through mitosis properly. Indeed, activation of the intrinsic apoptotic pathway was observed in our PCa models concurrent to Puma accumulation and reduction of Mcl-1 expression. This is consistent with the finding that AMPK activation by AICAR or upon block of glycolysis results in mTORC1 inhibition-dependent decrease of Mcl-1 through regulation of its translation (Pradelli *et al*, 2010). Interestingly, we also observed cell cycle arrest and apoptosis in p53-deficient context, suggesting that MT 63–78 might also be suitable for the treatment of p53 null/mutated PCas not responsive to biguanides (Ben Sahra *et al*, 2011).

PCa is characterized by alterations in the PI3k/Akt/mTOR pathway and by a unique lipogenic reprogramming, active both at tumor initiation and in advanced stages (Suburu & Chen, 2012). Since multiple components of the PI3k/Akt/mTOR pathway are deregulated in a wide variety of solid tumors, considerable attention has been focused on the biochemical and genetic characterization of this pathway in cancer and on developing new drugs to inhibit it (Wong *et al*, 2010; Laplante & Sabatini, 2012). However, activation of *de novo* lipogenesis has recently been recognized, not only as an adaptation to fulfill the metabolic requirements of highly proliferating cancer cells, but as a hallmark of tumorigenesis, tumor progression, chemoresistance, and development of androgen-resistance (Ettinger *et al*, 2004; Santos & Schulze, 2012). Newly synthesized lipids affect a number of cellular processes such as membrane biogenesis, signal transduction, intracellular trafficking, and lipid-based protein modification to promote prostate cancer cell proliferation and survival (Zadra *et al*, 2013). Indeed, overexpression and increased activity of FASN, a key enzyme responsible for the terminal step in FA synthesis, represents one of the most frequent phenotypic alterations in cancer cells (Brusselmans & Swinnen, 2009; reviewed in Flavin *et al*, 2011). Our data support the essential role of lipogenesis in PCa maintenance and progression and point out that AMPK-mediated repression of lipogenesis plays a dominant role in tumor growth inhibition.

Androgens are the major drivers of PCa carcinogenesis and progression and AR signaling is still active in castration resistant disease. Thus, targeting AR pathway remains the gold standard for both androgen sensitive and CRPCs. The AR antagonist MDV3100 and the CYP17A1 inibitor abiraterone have been FDA approved for the treatment of CRPC. However, despite encouraging results, emergence of treatment resistance represents a major limitation (Yuan *et al*, 2013). Previous studies have underscored the tight connection between AR signaling and increased *de novo* lipogenesis, expecially in the context of emergence of androgen independence (Swinnen *et al*, 1997; Ettinger *et al*, 2004), suggesting that targeting lipogenesis will be particularly beneficial in CRPC. Our data showing MT 63–78-mediated growth inhibitory effect in CRPC models and the discovery of lipogenesis inhibition as the effector arm of AMPK-mediated suppression of PCa growth, prompted us to evaluate whether combination of AR signaling inhibitors and direct AMPK activation would result in a potentiated growth inihibitory effect. Low concentration of MT 63–78 was sufficient to significantly enhance the rate of growth inhibition induced by AR antagonists bicalutamide, MDV3100 or by the steroidogenesis inhibitor abiraterone (recently also discovered as AR antagonist) (Richards *et al*, 2012). This enhanced anti growth effect also correlates with reduction in AR expression and activity (PSA production). These results support previous data using combination of bicalutamide and the indirect activator metformin (Colquhoun *et al*, 2012) and point to the crucial role of AMPK activation rather than the inhibition of the insulin pathway in mediating the additive effect of the combinatorial treatment. Results from the phase II clinical trial NCT01677897, which evaluates the impact of the addition of metformin to abiraterone in metastatic PCa patients, will also inform on the benefit of adjuct AMPK activators to current standard treatments.

PET is the most advanced technique for metabolic imaging and one of the most accurate tools for tumor staging in the pre-treatment and follow-up phases. Vavere *et al* showed a reduced tumor uptake of ^11^C-acetate in mice treated with FASN inhibitor C75, providing validation for development of ^11^C-acetate PET as measure of FA synthesis (Vavere *et al*, 2008). Thus, we investigated whether ^11^C-acetate could serve as *in vivo* biomarker of response to AMPK activation therapy. While our data with C75 fully confirm their findings, we report no decrease in ^11^C-acetate uptake following MT 63–78 treatment, showing that this is compatible with AMPK-mediated induction of ^11^C-acetate uptake for oxidation in the TCA cycle, concurrent with inhibition of FA synthesis. Our results thus discourage the use of ^11^C-acetate to monitor treatment with AMPK activators in the clinical setting, while the proliferation marker fluoro-L-thymidine (^18^F-FLT) might be a promising alternative (Habibollahi *et al*, 2013).

In conclusion, this study provides the biochemical and molecular underpinning for direct targeting of AMPK in PCa therapy, with the downregulation of lipogenesis representing the major downstream effect of this therapeutic approach. This study set also the groundwork for testing the combination of AMPK activators and AR signaling inhibitors in CRPC pre-clinical models.

## Materials and Methods

### Chemicals

A detailed list of the reagents used in the study is provided in the Supplementary materials and methods.

### Cell lines and culture conditions

Cell lines were maintained and cultured as described in the Supplementary materials and methods.

### Western blot analysis

Western blotting was performed as described in the Supplementary materials and methods.

### Pharmacokinetic experiments

MT 63–78 was administered i.p. or orally using the antiacid Mylanta as vehicle in three C57 BL/6 male mice. Compound concentration in the plasma was measured by HPLC/MS, as described in the Supplementary materials and methods.

### Measurement of AMPK activity

AMPK activity in total cell lysates was measured using CycLex AMPK Kinase Assay kit (MBL International Corporation) or alpha screen technology (Perkin Elmer), according to the manufacturer's instructions. *In vitro* AMPK activity was assessed using recombinant human AMPK (α1β1γ1, α2β1γ1, α1β2γ1, α2β2γ1) and alpha screen technology. Details are provided in the Supplementary materials and methods.

### Kinome screen

Kinase profiling was performed using The Upstate® KinaseProfiler service from Millipore.

### AMPK dephosphorylation assay

AMPK dephosphorylation experiments using recombinant AMPK α1β1γ1 were performed as described in the Supplementary materials and methods.

### Measurement of ATP, ADP, AMP levels

Measurment of intracellular ATP, ADP, AMP levels were carried out using commercial kits and HPLC, as described in the Supplementary materials and methods.

### Transient transfection experiments

Details regarding siRNA and plasmids are provided in the Supplementary materials and methods.

### Cell growth, viability, and soft agar assays

Cell growth was assessed by counting number of viable cells using the Vi-cell automatic counter (Beckman). Viability (% of viable cells/total cells) was also measured using the trypan blue exclusion method. Soft agar assays were performed as described in the Supplementary materials and methods.

### Cell cycle and apoptosis studies

Cell cycle and apoptosis studies were performed by flow citometry and western blotting as described in the Supplementary materials and methods.

### Hoechst staining

Staining with Hoechst 33342 was performed according to standard procedures. Details are provided in the Supplementary materials and methods.

### FASN activity

FASN activity in PCa cell lines was measured as previously described (Kuhajda *et al*, 1994). Details are provided in the Supplementary materials and methods.

### 2-^14^C-acetate incorporation into lipids and thin layer chromatography

2-^14^C-acetate incorporation into lipids and Thin Layer Chromatography (TLC) were performed as previously described (Bligh & Dyer, 1959; Bagnato & Igal, 2003) Details are provided in the Supplementary materials and methods.

### 2-^14^C -acetate oxidation experiments

2-^14^C -acetate oxidation experiments were performed using a standard protocol as described in the Supplementary materials and methods.

### Xenograft, GEMMs, and PET imaging

All animal studies were carried out in compliance with National and DFCI guidelines. A detailed description of animal studies (drug treatments, GEMMs, and PET imaging) is provided in the Supplementary materials and methods.

### Histopathology

Collection of murine prostate and histological procedures were performed as described in the Supplementary materials and methods. Hematoxylin and eosin staining was blindly evaluated by an expert pathologist (Sabina Signoretti) for the presence of prostate lesions.

### Measurement of serum PSA, glucose, and triglycerides

Serum PSA levels were measured using Total Prostate-Specific Antigen ELISA kit (Alpco). Glucose levels were measured glucometer (Abbott) and QuantiChrom™ Glucose Assay Kit (Bio Assays Systems). Triglycerides were measured by serum triglyceride Determination Kit (Sigma), as described by the manufacturer.

### Statistical analysis

Statistical analyses were done using two-tailed, unpaired t-test, Wilcoxon Mann-Whitney test, one-way analysis of variance (ANOVA) followed by post-hoc Bonferroni, Tukey's or Dunnett's tests, as required, and two-way ANOVA test. Analyses were performed using the Graphpad software, version 5d, StatPlus: mac 2009, and R statistic packages. *P* values < 0.05 were considered significant. Logistic regression analysis was performed using R statistics packages.

**The paper explained**

**Problem**

While metabolic reprogramming is now recognized as a hallmark of tumorigenesis and tumor progression, the development of metabolic-targeted therapy remains a challenge. Epidemiologic and experimental studies have identified the energy sensor AMPK as an ideal target not only for metabolic diseases but also for cancer. While biguanides such as metformin have cancer preventing/therapeutic properties, their metabolic effects beyond AMPK activation leave unanswered the question of whether AMPK activation *per se* is necessary and sufficient to affect tumor growth.

**Results**

Utilizing a novel, highly specific AMPK direct activator, we show that AMPK activation *per se* inhibits PCa cell growth *in vitro* and *in vivo*, whereas the allelic loss of AMPK catalytic subunits fosters PCa development. In addition, we report that AMPK anti-tumor effects are independent of the status of its upstream activator, the tumor suppressor LKB1. From a mechanistic standpoint, we demonstrate that inhibition of *de novo* lipogenesis is the dominant downstream effector of AMPK-mediated inhibition over mTORC1. Finally, we demonstrate that co-treatment of the direct AMPK activator with AR signaling inhibitors results in significantly enhanced growth inhibition in both androgen sensitive and CRPC models.

**Impact**

This study proposes AMPK as a crucial metabolic hub that can be targeted in PCa. Because suppression of lipogenesis is the predominant effector arm of AMPK activation, AMPK activators may be especially effective in lipogenesis-driven tumors. Combining AMPK activators with hormonal therapy results in a synergistic anti-cancer effect. PET imaging can be utilized as a non-invasive biomarker of efficacy of metabolic therapy.

## For more information

MT 63–78 patent:

http://www.google.com/patents/US20100009992
