# Supplementary material for: A novel direct activator of AMPK inhibits prostate cancer growth by blocking lipogenesis
Source: EMBO Mol Med. 2014 Feb 4;6(4):519–38. doi: 10.1002/emmm.201302734 (PMC3992078; doi:10.1002/emmm.201302734)
Supplement: Supplementary file 1 [file emmm0006-0519-sd1.pdf]

EMBO Molecular Medicine

A novel direct activator of AMPK inhibits prostate cancer growth by blocking lipogenesis.

Giorgia Zadra, Cornelia Photopoulos, Svitlana Tyekucheva, Pedram Heidari, Qing Ping Weng, Giuseppe Fedele, Hong Liu, Natalia Scaglia, Carmen Priolo, Ewa Sicinska, Umar Mahmood, Sabina Signoretti, Neal Birnberg and Massimo Loda

Corresponding author: Massimo Loda, Dana Farber Cancer Institute

Review timeline:

Submission date:	05 October 2012
Editorial Decision:	24 October 2012
Appeal:	24 October 2012
Additional Editorial Correspondence:	28 October 2012
Additional Author Correspondence :	07 March 2013
Resubmission:	09 March 2013
Editorial Decision:	18 April 2013
Revision received:	01 November 2013
Editorial Decision:	02 December 2013
Revision received:	14 December 2013
Accepted:	16 December 2013

Transaction Report:

(Note: With the exception of the correction of typographical or spelling errors that could be a source of ambiguity, letters and reports are not edited. The original formatting of letters and referee reports may not be reflected in this compilation.)

Editor: Roberto Buccione

1st Editorial Decision 24 October 2012

Thank you for the submission of your manuscript "Direct targeting of the energy sensor AMPK inhibits prostate cancer growth though blockade of the lipogenic switch: novel therapeutic perspective".

I have now had the opportunity to carefully read your paper and the related literature and I have also discussed it with my colleagues and with an expert member of the Editorial Board. I am afraid that we concluded that the manuscript is not well suited for publication in EMBO Molecular Medicine and have therefore decided not to proceed with peer review.

The manuscript reports on a novel small molecular weight AMPK activator, MT 63-78, which blocks both proliferation and anchorage-independent growth of prostate cancer cells, mostly by inhibition of fatty acid synthase and cholesterol synthesis. You also show that in a tumour xenograft model, MT 63-78 inhibited tumour growth with the expected molecular readout.

We appreciate that you also demonstrate the potential use of PET to monitor AMPK activation in vivo and the development of a gene signature strategy to identify a subset of patients with tumor features potentially suitable for treatment with AMPK activators.

Your work is certainly within the scope of EMBO Molecular Medicine but unfortunately we feel that, as it is, the manuscript falls short on a few important issues. The lack of details on the structure and the functional characterisation of MT 63-78 (i.e. specificity, kinase profiling panel and other) and the somewhat preliminary preclinical characterization of MT 63-78 (there are possibly additional and/or better-suited mouse models including for prostate cancer), prevents us from considering this manuscript further.

I am sorry that I could not bring better news this time.

Appeal 24 October 2012

Thank you very much for your reply and for the encouraging comments on the novelty and the appropriate molecular readouts in our experiments.

In terms of the important issues regarding the structure and the pre-clinical characterization of MT 63-78, we intentionally left these data out to emphasize the biochemical and cellular biology effects of direct AMPK activation, utilizing MT 63-78 merely as a tool compound. However, a patent has been issued on the drug, therefore the structure is available, and we can certainly provide all kind of pharmacokinetics, kinase panel (?akin to the ones published in J Biol Chem. 2007 Nov 9;282(45):32549-60 and Biochem J. 2007 Dec 15;408(3):297-315), glucose tolerance test, oral bioavailability etc, if you feel that would not distract from the paper. In terms of additional prostate cancer models, we have data on our AMPK conditional alpha1/2 knock outs that would provide the reverse genetic equivalent of the pharmacologic activation of AMPK. We have not yet tested other genetically engineered strains (we have most of them in our lab) but could do so should you deem it necessary.

If providing this information would be helpful in the re-consideration of our manuscript, please let us know and I will be happy to provide you with a revised version of the manuscript. This might take a short or a relatively longer time, depending on the data you'd like us to provide.

Additional Editorial Correspondence 28 October 2012

Thank you very much for your message.

I have now re-discussed your manuscript with my colleagues and the Editorial Board Member.

Considering the potential interest of your findings, we would be happy to consider a new manuscript that incorporated 1) data concerning pharmacokinetics, kinase panel, glucose tolerance test, etc. and 2) additional pre-clinical evidence on a prostate cancer model. The former data can be included as supplemental information.

Please consider that, technically, yours will be a new submission. However, and provided the manuscript is integrated as mentioned above, I will endeavor to process it rapidly for peer review.

Additional Author Correspondence 07 March 2013

We are finally planning to re-submit our manuscript by Zadra et al., entitled ≥Direct targeting of the energy sensor AMPK inhibits prostate cancer growth though blockade of the lipogenic switch≤ for your consideration in ≥EMBO Molecular Medicine≤. We apologize for the delay in the re-submission but it took us several months to address all your previous requests.

Specifically, in the revised manuscript we incorporated, as requested, the following new data:

a) the molecular structure of the novel small molecule MT 63-78;

b) the in vitro kinase panel screening to further demonstrate the specificity of the compound for AMPK;

c) pharmacokinetics studies and in vivo metabolic effects of MT 63-78 (glucose levels, glucose tolerance test);

d) a second mouse model (genetically engineered mouse model of AMPK alpha 2 subunit ablation) to support our in vivo data on the tumor suppressor function of AMPK in prostate cancer.

We are really grateful for your suggestions and we think that this improved version of the manuscript will be of greater significance and impact to the readership of EMBO Molecular Medicine.

We will re-submit in the next couple of days. We understand that it will be treated as an entirely new manuscript.

Resubmission 09 March 2013

We are re-submitting a manuscript by Zadra et al., entitled "Direct targeting of the energy sensor AMPK inhibits prostate cancer growth though blockade of the lipogenic switch" for your consideration in "EMBO Molecular Medicine".

This novel version of the manuscript addresses all the previous requests from the Editorial Board and we believe that this implemented version of the manuscript will be of greater significance to the readership of EMBO Molecular Medicine.

Specifically, the current version of the manuscript incorporated the following new data a) the molecular structure of the novel small molecule MT 63-78; b) in vitro kinase screening to further demonstrate the specificity of the compound for AMPK c) pharmacokinetics studies and in vivo metabolic effects of MT 63-78 (glucose levels, glucose tolerance test) d) a second mouse model (genetically engineered mouse model of AMPK alpha 2 subunit ablation) to support our in vivo data of the oncosuppressor function of AMPK in prostate cancer.

While metabolic reprogramming is now recognized as a classical hallmark of tumorigenesis and tumor progression, the development of metabolic-targeted therapy remains a challenge. Here, we show how interfering with cancer metabolism by targeting a fundamental metabolic gauge represents a valid approach for cancer therapy.

The energy sensor 5'AMP-activated kinase (AMPK) constitutes a hub for cellular metabolic control and links cell metabolism to growth via the negative regulation of anabolic pathways, significantly up-regulated in cancer cells, thus representing an ideal therapeutic target in oncology. In fact, several clinical trials in prostate and other cancers using the indirect AMPK activator metformin are currently underway.

Despite promising results using metformin in several tumor models and epidemiologic data pointing at biguanides as cancer preventing/therapeutic agents, whether AMPK activation per se is necessary and sufficient to affect tumor growth remains elusive.

In this study, we investigate the biological consequences of inducing a specific and direct activation of AMPK in cancer using prostate as model. To do this, we carefully characterized a novel direct AMPK activator (MT 63-78), obtained by screening a chemical library using recombinant purified AMPK. We demonstrate that AMPK activation per se inhibits prostate cancer cell growth in vitro and in xenograft models, inhibits anchorage-independent growth in soft-agar, induces cell cycle arrest in the G2-M phase, and apoptosis. The mechanism of action of the compound, while strictly AMPK-dependent, is independent of the status of its upstream kinase, the tumor suppressor LKB1, thus offering novel therapeutic opportunities for the treatment of LKB1-null/mutated tumors. Importantly, we show that the suppression of de novo lipogenesis plays the major role in AMPK-mediated prostate cancer growth inhibition compared to mTORC1 pathway inhibition, again opening new therapeutic perspectives. In addition, we perform positron emission tomography using

the lipid precursor 11C-acetate in xenograft models as a non-invasive assessment of AMPK activation in vivo. Taking a complementary approach, we further validate our results by demonstrating that the reduction of AMPK catalytic activity in a genetically engineered mouse model of AMPK subunit alpha 2 knockdown is sufficient to promote PCa development.

From the perspective of prostate cancer molecular classification and targeted-therapy, we describe two important results: 1) the development of a new small-molecule approach to prove the pre-clinical efficacy of directly targeting a master metabolic sensor; 2) the identification of a gene-signature in prostate cancer patients, indicative of the lipogenic phenotype associated with AMPK deactivation. Patient screening using this profile will potentially identify ideal candidates for the treatment with AMPK direct activators and/or inhibitors of lipogenesis.

In summary, we believe our results lay the groundwork for the development of specific AMPK activators as a novel therapeutic strategy for prostate and possibly other cancers and provide a novel molecular biomarker to guide the choice of adequate treatment options.

Thank you for your consideration. We look forward to your reply.

2nd Editorial Decision 18 April 2013

Thank you for the submission of your manuscript to EMBO Molecular Medicine. We have now heard back from the three Reviewers whom we asked to evaluate your manuscript.
You will see while the Reviewers are generally supportive of you work, they express a number of concerns that prevent us from considering publication at this time. I will not dwell into much detail, as the evaluations are detailed and self-explanatory. I would like, however, to highlight a few main points.

Reviewer 1 notes for instance that to better assess autophagy upon AMPK activation, LC3 fluxes should be measured and appropriate controls used. Reviewer 1 is also concerned that a castrate-resistant setting should be used to assess the impact of the compound. S/he also suggests that serum PSA levels would serve as useful indicators of anti-cancer activity. This Reviewer also lists other critical issues that require your intervention and mentions a few items that should be better discussed or supported with additional experimentation.

Reviewer 2 is concerned about the overall novelty of your findings and in this respect, would like you to explain how MC 63-78 is different/better from the compounds currently being tested. Connected to this, s/he also notes the lack of demonstration of specificity that, together with the fact that the compound is also effective on normal prostate epithelial cells, raises concerns on relevance. Finally, in agreement with Reviewer 1, this Reviewer also feels that demonstration of efficacy in a castration resistant setting is required. Although Reviewer 1 does not specifically call for you to perform these experiments, I would strongly suggest that you address this issue based also on Reviewer 2's request for further in vivo experimentation.

Reviewer 3 also addresses the issue of MC 63-78 selectivity and would like to see clear evidence for isoform selectivity. S/he also suggests that the FASN signature data is preliminary in nature and should be taken out. Reviewer 1 agreed with this during cross-commenting. I share this opinion and also agree with this Reviewer's assessment that the manuscript could be shortened and streamlined.

Considered all the above, while publication of the paper cannot be considered at this stage, we would be prepared to consider a revised submission, with the understanding that the Reviewers' concerns must be fully addressed, with additional experimental data where appropriate and that acceptance of the manuscript will entail a second round of review.

Please note that it is EMBO Molecular Medicine policy to allow a single round of revision only and that, therefore, acceptance or rejection of the manuscript will depend on the completeness of your responses included in the next, final version of the manuscript.

As you know, EMBO Molecular Medicine has a "scooping protection" policy, whereby similar findings that are published by others during review or revision are not a criterion for rejection.

However, I do ask you to get in touch with us after three months if you have not completed your revision, to update us on the status. Please also contact us as soon as possible if similar work is published elsewhere.

I look forward to seeing a revised form of your manuscript as soon as possible.

***** Reviewer's comments *****

Referee #1 (Comments on Novelty/Model System):

several prostate cancer cell lines were used.
Only LNCaP tumor volume is used to assess anticancer activity in vivo. Is this in intact male hosts? If so, why not use a castrate resistant LNCAP xenografts grown in castrated mice, where lipogenesis pathways may be more relevant biologically and clinically relevant (Ettinger et al Can Res)? A more appropriate design would have included castration +/- MT 63-78 and then measuring time to castrate resistant recurrent progression. Also in vivo tumor growth was only measured for 13 days, which is a very short treatment period for a slow growing model like LNCaP. Finally, serum PSA levels serve as a useful surrogate of anti-cancer activity and TV in the LNCaP model - was serum PSA measured?

Referee #1 (Remarks):

Re: AMPK direct activator MT 63-78
The authors report on a novel highly selective small molecule activator of AMPK that inhibits PCa cell growth in vitro and in vivo, and that allelic loss of AMPK catalytic subunits fosters PCa development in genetically engineered mice. MT 63-78 is a specific and potent direct activator of AMPK in PCa and that activation of AMPK in response to MT63-78 is independent of LKB1 when CaMKK is functional. They show that suppression of lipogenesis is the predominant mechanism responsible for AMPK-mediated PCa growth inhibition and derived a human gene-expression signature as a biomarker of PCas with increased de novo lipogenesis and AMPK deactivation. The studies are well conceived, thesis-like in scale and scope, convincing, and an important contribution to the field. While the report provides a strong foundation for future studies, it is contextually agnostic and would be improved by discussion on future co-targeting combinatorial approaches to manipulate cancer cell sensitivity by switching lipogenesis under context dependent conditions. For example, while they include AR+ and AR- prostate cancer cell lines, AR coordinated pathways remains the main driver of survival and progression, some discussion regarding interactions between AR, androgen deprivation, and AMPK activation should be provided.

Points:
1. Title: has incorrect word "through" not "though"?

2. The authors state that MT 63-78 exerts its effect by binding to the AMPK regulatory beta subunit. However it's not clear yet how the binding activates AMPK alpha activity. Does the binding enhance the interaction between alpha and beta subunits or does the binding sensitize the alpha subunit to be phosphorylated by upstream signaling? Or does drug lead to inhibition of dephosphorylation? In Fig 1 there is clear dose dependent activation of p-ACC downstream of AMPK phosphoryation; they should show one more panel showing downstream effects of MT63-78 on p-ACC +/- PP2Calpha
3. In Fig. 4A, B, C and D reports cell number counting (measured by either cell counter or trypan blue exclusion method) to determine "cell proliferation" in the 2nd paragraph on page 6. The cell number actually presents the balance after cell proliferation and cell death (apoptosis). Instead of "cell proliferation", "cell growth" is more appropriate esp. when agent induces cell death (Fig. 5). The authors should use "cell growth" instead of "cell proliferation" in the 2nd paragraph of page 6. Effects on cell proliferation can be determined by FACS as shown in Fig.5. Since both Fig 4 and 5 deal with in vitro studies of cell growth, proliferation, cell death and autophagy, they could be combined into one figure as they discuss the effects on.
4. While induction of autophagy upon AMPK activation has been previously reported, these authors found no increase of the autophagic markers Beclin1 and LC3-II (fig 5). However, in order to

measure the autophagy level, the LC3 flux should be examined. But this needs to be done under +/- conditions with chloroquire. To more accurately measure production of LC3II, the lysosomal enzyme inhibitor, chloroquine (CQ), is used to block degradation of LC3II protein. In addition, LC3 immunofluorescent staining for LC3 puncta is another method to exam autophagy levels. A positive control for treatment induced autophagy should also be included.
5. In Fig 8a, only LNCaP tumor volume is used to assess anticancer activity in vivo. Is this in intact male hosts? If so, why not use a castrate resistant LNCAP xenografts grown in castrated mice, where lipogenesis pathways may be more relevant biologically and clinically relevant (Ettinger et al Can Res)? A more appropriate design would have included castration +/- MT 63-78 and then measuring time to castrate resistant recurrent progression. Also in vivo tumor growth was only measured for 13 days, which is a very short treatment period for a slow growing model like LNCaP. Finally, serum PSA levels serve as a useful surrogate of anti-cancer activity and TV in the LNCaP model - was serum PSA measured? While I do not think that these experiments need be done for this paper, discussion of biologic and clinical relevance of combining with AR pathway inhibitors in CRPC should be included as future directions.
6. The pd markers p-ACC and p-Raptor levels do not appear significantly different between the two groups. Contextually, AMPK activation may have more pd effects and anti-cancer activity under conditions of post-castration development resistance.
7. The gene expression signatures in fig 11 are from treatment-naÔve prostate cancers; since many studies link increased lipogenesis in prostate cancer with emergence of castration resistance, and the authors themselves refer to the importance of targeting therapy in molecularly segmented patient subpopulations, it is important to understand the role AMPK in the context of AR targeting agents and emergence of AR pathway inhibition. Also, new drug development mostly, if not always, needs to focus in the castrate resistant state. For this reason it would be relevant to include gene lists of CRPC in this signature.
8. Other AMPK activators (such as AIACR) have been reported to suppress growths of several types cancers. Does MT 63-78 affect growth of other types of cancers?

Referee #2 (Remarks):

Previous studies demonstrated that de novo lipogenesis and upregulation of the mTOR pathway occurs frequently in human prostate cancer. AMPK serves as an energy sensor that controls metabolism and cell growth, and it is hypothesized in the present study that this could serve as a therapeutic target. A small molecule target, MC 63-78, was identified in this study, and functions assessed. Key reported findings are that MTs 63-78: induces direct activation of AMPK and prevents its dephosphorylation; activates AMPK without altering energy levels; does not require LKB1, inhibits prostate cancer proliferation and growth in soft agar, promotes G2-M arrest and apoptosis, is partially reliant on inhibition of mTOR1, elicits anti-growth effects via suppression of lipogenesis, and alters human tumor xenograft growth in vivo. Finally, a gene expression signature that could potentially serve as a biomarker for tumors with the altered lipogenesis and AMPK inactivation was identified--it is hypothesized that this could be used to identify the subset of patients likely to respond to AMPK activators.

Critique: This is an interesting preclinical study that adds to the body of evidence suggesting AMPK could be a therapeutic target in prostate cancer. Novel aspects of the study include identification of the small molecule, functional assessment of the small molecule, and identification of a gene signature that might identify tumors that might respond to AMPK activators. While the study is generally well conducted, the following issues limit the impact of the findings:
1. The concept that AMPK can suppress tumor growth and progression is not new, and direct AMPK inhibitors are already being tested in the clinical setting. While development of the MTV 63-78 small molecule is appreciated as a potentially new and important advance, it is unclear how this compound differs in function from those already developed and under clinical assessment. This should be determined.
2. Many of the cellular effects depend on readouts in only one AR positive prostate cancer cell line, LNCAP, and one AR- negative cell line of limited clinical relevance. It is unclear whether the effects of the compound are limited to prostate cancer cells, exert effects in other tumor types, or are limited to malignant versus non-transformed cells. Lack of demonstration for specificity is a major concern. This concern is further enhanced by the demonstration in figure 4 that the compound

showed major effects on normal prostatic epithelial cells.
3. As AMPK inhibitors would likely be used in combination with androgen deprivation therapy (standard of care for metastatic disease), the impact of this compound under conditions mimicking castration therapy should be determined.
4. The potential effects on cell cycle and cell death are interesting but not compelling as shown. These endpoints should be assessed in a broader spectrum of model systems, flow traces shown (for cell cycle), and underlying mechanisms discerned.
5. Xenograft studies in figure 8 are an important element of the study. The changes seen, however, will were not robust. Demonstration of the fact in the second model system, for example using models of castration resistant disease, would enhance enthusiasm for the findings.

Referee #3 (Comments on Novelty/Model System):

Please see my justifications in the remarks to the authors.

Referee #3 (Remarks):

This is an interesting and thorough manuscript describing the effects of a new and previously-uncharacterised small molecule activator of AMPK which acts via the beta-1 subunit. There is increasing recognition that the LKB1/AMPK axis may provide an interesting therapeutic target in some forms of cancer though, as the authors point out, the use of agents pointing towards this conclusion such as metformin can be criticised given a possible spectrum of off-target effects.

The authors propose that MT 68-73 may provide a more selective agent to activate AMPK (by binding to the beta subunit) than A-769662, albeit acting in a similar way at the molecular level. They provide convincing a detailed data describing the effects of the drug on the growth of specific cancer cell types, notably those derived from prostate cancers.

Major.

1. Is MT 68-73 absolutely selective for AMPK beta1 over beta2? For the experiments shown in Fig, 1B and Fig. 2E it would seem important to present the equivalent experiments using/targeting respectively the beta2 complexes.
2. The identification of a gene signature activated by FASN expression (Fig. 11) seems rather redundant in the main manuscript given that no evidence is provided that these will genuinely be targeted either at the transcriptomic or post-translational level by activated AMPK (even though it's a perfectly reasonable prediction that lipogenic pathways will be affected by the actions on ACC and HMG-CoA reductase). Whether tumors with a particularly elevated lipogenic gene signature would be more or less susceptible to treatment is also not completely clear. In the absence of such a test (i.e correlating suspectibility to cell growth inhibition with degree of "lipogenic reprogramming") this part of the manuscript feels unfinished and should transferred to the Supplementary section or removed altogether

Minor

1. The experiments shown in Fig. 1B are convincing but one wonders how the potency of the new drug compares to that of A-769662 in this assay? Please comment.

2. Fig. 1. MT 63-78 is a highly hydrophobic molecule which may serve as a protonophore and mitochondrial uncoupler to impair ATP synthesis. This appears not to be a major problem (Fig. 2C) but one would like to be further reassured by measurements of intracellular ADP or AMP in addition to ATP. I note that the kit used purportedly measures ATP/ADP ratio rather than total ATP? Please clarify.

3. Inhibition of MST-1, a major executor of apoptosis via Hippo signalling, may be problematic in the context of cancer treatments. Comment?

4. Page 5: "In contrast to AICAR and metformin, the growth inhibitory effect of MT 63-78 was strictly dependent on the expression of AMPK (Fig 4C)." Data for the effects of AICAR and metformin appear to be missing and should be included or the appropriate literature cited.
4. The paper is over-long. Removal of the FASN signature data may help but I suspect further pruning by 10 % overall could easily be achieved.

1st Revision - authors' response 01 November 2013

We are submitting the revised version of our manuscript entitled “Direct targeting of the energy sensor AMPK inhibits prostate cancer growth through blockade of the lipogenic switch” for consideration for publication in *EMBO Molecular Medicine*. The new, extensively revised version of the manuscript addresses all the points requested by the reviewers (see below).

Specifically, we now address specificity and selectivity issues of MT 63-78 and compare the effects of MT 63-78 with the only current available AMPK direct activator A-769662 that displays a similar mechanism of action. We provide the mechanistic underpinnings of MT 63-78-induced mitotic arrest and apoptosis. Novel *in vitro* data are provided on the growth inhibitory effect of MT 63-78 in several castration resistant prostate cancer (CRPC) models. We demonstrate that combinatorial treatment of MT 63-78 and AR signalling inhibitors Bicalutamide, Enzalutamide (MDV3100), and Abiraterone results in enhanced inhibition of cell growth with respect to monotherapy in both androgen dependent and castration resistant cell lines. As requested by the reviewers, we removed the experiments relative to the gene expression signature, which was deemed to be too premature for publication. We also included additional *in vivo* data with higher concentration and prolonged treatment with MT 63-78, as requested. As result of these changes we have modified the list of authors of the new manuscript (Travis Gerke, Toshiro Migita, and Lorelei Mucci have been removed; Qing Ping Weng has been added).

Importantly, extensive efforts were put into attempts at generating adequate CRPC xenograft models in castrate nude mice (see detailed response to reviewers below). Unfortunately, we succeeded only in obtaining CL1 and 22Rv1 xenograft models *in vivo*, out of 4 lines we attempted, but these displayed either growth rates that were too fast and/or underwent extensive necrosis, precluding adequate assessment of response. In order to perform experiments with our compound, which would be comparable to the hormonal naïve setting, we would have required significant additional time. Thus, while we are currently working on CRPC models for *in vivo* AMPK activation testing, we feels this now falls beyond the scope of the current manuscript. We are confident, however, that all the novel data included totally fulfil the scope of this manuscript and will be of great interest for the readers of *EMBO Molecular Medicine*.

Below, please find the point-by-point response to the reviewers:

<u>Referee 1</u>

The authors report on a novel highly selective small molecule activator of AMPK that inhibits PCa cell growth in vitro and in vivo, and that allelic loss of AMPK catalytic subunits fosters PCa development in genetically engineered mice. MT 63-78 is a specific and potent direct activator of AMPK in PCa and that activation of AMPK in response to MT63-78 is independent of LKB1 when CaMKK2b is functional. They show that suppression of lipogenesis is the predominant mechanism responsible for AMPK-mediated PCa growth inhibition and derived a human gene-expression signature as a biomarker of PCas with increased de novo lipogenesis and AMPK deactivation. The studies are well conceived, thesis-like in scale and scope, convincing, and an important contribution to the field.

We thank this reviewer for the comments on our work.

1. *Only LNCaP tumour volume is used to assess anticancer activity in vivo. Is this in intact male hosts? If so, why not use a castrate-resistant LNCAP xenografts grown in castrated mice, where lipogenesis pathways may be more relevant biologically and clinically relevant (Ettinger et al Can Res). A more appropriate design would have included castration +/- MT63-78 and then measuring time to castrate resistant recurrent progression.*

While I do not think that these experiments need be done for this paper, discussion of biologic and clinical relevance of combining with AR pathway inhibitors in CRPC should be included as future directions.

We found the suggestion of testing the effect of MT 63-78 compound in a castrate resistant setting important for our studies. Castration-resistant lines have been derived *in vitro* by culturing LNCaP cells in the absence of androgen (CL1 cells) or from xenografts serially propagated in mice after castration-induced regression and relapse of the parental, androgen-dependent xenograft (C4-2 and C4-2B from LNCaP xenograft, and 22Rv1 from CWR22 xenograft). We injected subcutaneously C4-2, C4-2B, CL1, and 22Rv1 lines (these same lines were used in our *in vitro* studies) in nu/nu castrated mice to establish the best castration-resistant xenograft model to use for MT 63-78 testing. Unfortunately none of the C4-2 and C4-2B injected mice formed tumours. In contrast, 22RV1 and CL1 cells grew extremely rapidly forming tumours, which became rapidly necrotic and began to shrink as soon as a week after injection. Thus, these were inadequate models for our drug testing. While we are currently attempting to optimize all these models, including the development of a castrate resistant LNCaP xenograft model (the development of which should take several months), we have included these in our future directions, as suggested by the Reviewer.

We provided new and exciting data supporting the therapeutic benefit of a combining AMPK activators and AR signalling inhibitors. Specifically, we tested the growth inhibitory effect of the combinatorial treatment of MT 63-78 with the AR antagonists Bicalutamide and MDV3100, and the CYP17A1 inhibitor Abiraterone in several androgen sensitive and castration resistant PCa models, showing that combining MT 63-78 and AR signalling inhibitors significantly reduces PCa cell growth further than either monotherapy. We also measured the effect of AMPK direct activator MT 63-78 on AR receptor expression levels and activity (using PSA levels as readout) and in *combination* with AR signalling inhibitors. These new data are shown in Figure 9. As suggested by the Reviewer, a discussion of the biologic and clinical relevance of combining MT 63-78 with the AR pathway inhibitors in CRPC has been included in the revised version of the manuscript (see revised Introduction and Discussion).

2. *In vivo tumour growth was only measured for 13 days, which is a very short treatment period for a slow growing model like LNCaP.*

We agree that MT 63-78 i.p. injection for 14 days in the LNCaP model was not a sufficiently long treatment period. Thus, we repeated the *in vivo* experiments with LNCaP xenografts, treating the mice for 21 days and using 60 mg/kg of drug, since no side effects were previously observed. By increasing the exposure time and the compound concentration, we observed a stronger effect of MT 63-78 in tumour growth inhibition *in vivo* than that previously observed with 30 mg/kg/d for 14 days. These new data are now included as Figure 10D. We could not prolong the treatment for more than 21 days since the tumour volume of some control mice had already exceeding the size limit imposed by the IACUC at Dana-Farber Cancer Institute.

3. *Finally, serum PSA levels serves as a useful surrogate of anti-cancer activity and tumour volume in the LNCaP model - was serum PSA measured?*

As requested, we measured PSA in this novel set of mice for which serum was available. These new data are in Figure 10F.

4. *The pd markers p-ACC and p-Raptor levels do not appear significantly different between the two groups (treated and untreated).*

We agree with the Reviewer that the difference in the two groups, untreated and treated, using 30 mg/kg/d, despite being significant, is not striking. However, when we repeated the experiment, an increase in the concentration and duration of the treatment in LNCaP xenograft models resulted in a stronger increase in p-ACC and p-Raptor in treated tumours with respect to controls. These data have now been included as Figure 10E.

5. *Does the binding of MT 63-78 to the AMPK regulatory beta subunit enhance the interaction between alpha and beta subunits, sensitize the alpha subunit to be phosphorylated by upstream signalling or lead to inhibition of dephosphorylation? The authors should show one more panel showing downstream effects of MT63-78 on p-ACC +/- PP2Calpha.*

This is an excellent point. We performed *in vitro* assay where we assess the effect of *MT63-78* on recombinant AMPK α1β1γ1 in the presence or absence of the phosphatase *PP2Calpha* (Figure 1C). We demonstrate that MT 63-78 leads to inhibition of AMPK dephosphorylation akin to the results obtained with A-769662 (Abbott Laboratories), using the same experimental procedure (Sanders et al, 2007).

6. *The cell number (measured by either cell counter or trypan blue exclusion method) actually presents the balance between cell proliferation and apoptosis. Instead of "cell proliferation", "cell growth" is the more appropriate term. Since both Fig 4 and 5 deal with in vitro studies of cell growth, proliferation, cell death and autophagy, they could be combined into one figure as they discuss the effects on.*

We thank the Reviewer for the observation and suggestions. “Cell proliferation” has been changed to “cell growth”. Extensive new data have been included on the effect of MT 63-78 on cell growth, cell cycle, and apoptosis and the underpinning molecular mechanisms of these effects in several androgen-sensitive and castration-resistant prostate cancer models. Thus, in the revised version of the manuscript, figures have been re-arranged accordingly:

a. Figure 4 now contains new cell growth data in several CRPC lines, b. Supporting Information Fig 6 contains novel data of MT 63-78-mediated cell growth inhibition in 12 different tumour cell lines, c. Figure 5 contains new data on cell cycle effects in both androgen sensitive and castration-resistant cells as well as in-depth molecular characterization of MT 63-78 induced-mitotic arrest d. Figure 6 contains new data on apoptosis in both androgen sensitive and castration-resistant cells and the respective molecular characterization of these effects.

7. *While induction of autophagy upon AMPK activation has been previously reported, these authors found no increase of the autophagic marker Beclin1 and LC3-II. However, in order to measure the autophagy level, the LC3 flux should be examined under +/- conditions of the lysosomal enzyme inhibitor chloroquine to block degradation of LC3II protein. A positive control for treatment-induced autophagy should also be included.*

As suggested, we performed the experiment under conditions of +/- with chloroquine and also using another lysosomal inhibitor, Bafilomycin A1. As a control we used treatment with rapamycin, a well-established inducer of autophagy. These new data show moderate increase in LC3-II in LNCaP cells after 24 hrs of treatment. In PC3 cells, the same effect was observed but only after 48 hrs. We included

these revised data in Figure 6C. No induction of autophagy was observed at earlier time points (6 hrs, not included in the figure).

8. *The gene expression signatures in fig 11 are from treatment-naive prostate cancers; since many studies link increased lipogenesis in prostate cancer with emergence of castration resistance, and the authors themselves refer to the importance of targeting therapy in molecularly segmented patient subpopulations, it is important to understand the role AMPK in the context of AR targeting agents and emergence of AR pathway inhibition. Also, new drug development mostly, if not always, needs to focus in the castrate resistant state. For this reason it would be relevant to include gene lists of CRPC in this signature.*

We greatly appreciate the comments and suggestions of the Reviewer. Since Reviewer 3 and the Editor also feel that the proposed lipogenic signature is still too preliminary and suggest that it should be removed from the manuscript, this part has been taken out from the current version.

9. *Other AMPK activators (such as AICAR) have been reported to suppress growths of several types cancers. Does MT 63-78 affect growth of other types of cancers?*

We thank the Reviewer for pointing out this aspect. We generated new data showing that MT 63-78 is able to inhibit the growth of a variety of cancer types, including the LKB1 null lung cancer A549, and the BRAF mutated thyroid cancer cell line KTC. In total, we tested MT 63-78 in 12 other cancer types (breast, lung, skin, esophagus, liver, stomach. thyroid, colon, pancreas, mantle cell lymphoma, bone, cervix), showing that the compound is effective in all of them. As mentioned above, these new data are included as Supporting Information Fig 6.

10. *Title: has incorrect word "through" not "though"?*

Title has been corrected.

<u>Referee 2</u>

This is an interesting preclinical study that adds to the body of evidence suggesting AMPK could be a therapeutic target in prostate cancer. Novel aspects of the study include identification of the small molecule, functional assessment of the small molecule, and identification of a gene signature that might identify tumours that might respond to AMPK activators.

We thank the reviewer for these positive comments

11. *The concept that AMPK can suppress tumour growth and progression is not new, and direct AMPK activators are already being tested in the clinical setting. While development of the MT 63-78 small molecule is appreciated as a potentially new and important advance, it is unclear how this compound differs in function from those already developed and under clinical assessment. This should be determined.*

Aside from metformin, which is an indirect activator of AMPK, we are not aware of any other direct AMPK activator that has reached the clinical setting as of yet. The well-characterized AMP mimetic AICAR does not show AMPK specificity. Substantial evidence, in fact, supports the notion that AICAR exerts AMPK-independent effects, mainly on AMP regulated enzymes (including glucokinase, glycogen phosphorylase, glycogen synthase, fructose 1,6-biphosphates and on mitochondrial oxidative phosphorylation (Vincent M et al, 1991; Longnus et al, 2003; Shang J et al, 2004; Guigas B et al, 2007; Guigas B et al, 2009). In terms of cancer therapy, recent papers reported AMPK-independent apoptosis and cell cycle arrest induced by AICAR in several cancer types

including chronic lymphocytic leukemia (Santindrian AF et al., 2010), breast cancer (Garcia Garcia C et al, 2010) and prostate (Park HU et al, 2012). In addition, despite rare studies in which AICAR was utilized in patients with myocardial ischemia and during cardiac bypass surgery, its unfavourable pharmacokinetic properties (i.e. high effective concentration, poor bioavailability) and severe metabolic complications like lactic acidosis and increased uric acid production, make AICAR a poor candidate for long-term use (Goodyear L, 2008). Finally, in terms of comparison with MT 63-78, our *in vitro* data show that MT 63-78 is significant more potent than AICAR in activating AMPK in PCa cell lines (Figure 2B, Supporting Information Fig1B).

To specifically address the question of the Reviewer, we included novel data comparing MT 63-78 with A-769662 (from Abbott Laboratories), the only current available small molecule, which acts with a similar mechanism. Thus, we analyzed: 1) MT 63-78 and A-769662 *in vitro* potency using four different recombinant AMPK heterotrimers (a1b1g1, a2b1g1, a1b2g1, a2b2g1); 2) AMPK activity in PCa cells under treatment with MT 63-78 or A-769662 3) The effect on cell growth of several androgen sensitive and castration-resistant PCa cells, following treatment with MT 63-78 or A-769662. Our data show that:

a) *In vitro*, both MT 63-78 and A-769662 show maximal AMPK activation in β1 subunit-containing heterotrimers, with a superior effect for A-769662. However, at high doses MT 63-78 is able to activate b2-containing heterotrimers, whereas A-769662 shows minimal or no activation, suggesting a potential low-affinity binding of MT 63-78 to the b2 subunit, especially when complexed to the a2 subunit (these novel data are now included as Supporting Information Fig 4A).

b) The potency of MT 63-78 is far superior than A-769662 in PCa cell-based context. Indeed, our data show that AMPK activity measured in LNCaP and PC3 cell lysates, using alpha screen assay, is significantly higher under treatment with low doses of MT 63-78 with respect to A-769662 (these data are now included as Supporting Information Fig 1B).

c) MT 63-78 induces cell growth inhibition (in both androgen sensitive and AI cell lines) at concentration 16-40 times lower than A-769662, according to the different PCa cell lines. These data are now included as Supporting Information Fig 5C.

d) Finally, MT 63-78 displays good oral availability (Supporting Information Fig 1A), in contrast to A-769662 (Cool et al., 2006), suggesting MT 63-78 as a better candidate for translation into the clinical setting.

12. *Many of the cellular effects depend on readouts in only one AR positive prostate cancer cell line, LNCAP, and one AR- negative cell line of limited clinical relevance. It is unclear whether the effects of the compound are limited to prostate cancer cells, exert effects in other tumour types, or are limited to malignant versus non-transformed cells. Lack of demonstration for specificity is a major concern. This concern is further enhanced by the demonstration in figure 4 that the compound showed major effects on normal prostatic epithelial cells.*

We have addressed this issue in response to point 6 to reviewer 1. To reiterate, we now included new data showing the cell growth inhibitory effect of MT 63-78 in more adequate castration resistant PCa cell models. We observed a significant growth inhibitory effect of the compound in all CRPC models tested. These data are now included in Figure 4B.

We also demonstrated that MT 63-78 anti-tumor activity is not limited to prostate cancer cells. Indeed we tested other 12 different types of cancers. Please see response to point 9 to reviewer 1. These new data are included as Supporting Information Fig 6.

Regarding the concern on the effect of MT 63-78 on non-transformed prostate cell line, we are not surprised to see an inhibitory effect on iPrEC and RWPE-1 at the concentration we used (25 uM, 50uM) since these are immortalized rapidly growing non-transformed models. However, this effect was always lower than that in all the PCa cell lines we tested, including CRPC cells. We also performed additional experiments with progressive reduction in concentration of the compound, which demonstrate that at limiting concentration of the drug, PCa cells remain sensitive to the compound whereas non-transformed cells become resistant. These data are now shown as Supporting Information Fig 5 A, B. In addition, if we were to speculate on the use of direct AMPK activators in the clinical setting, effects on adjacent normal prostatic tissue might not be as undesirable as in other organs.

13. *As AMPK activators would likely be used in combination with androgen deprivation therapy (standard of care for metastatic disease), the impact of this compound under conditions mimicking castration therapy should be determined.*

We agree with the Reviewer regarding the importance of evaluating the effect of combining androgen deprivation therapy and MT 63-78. Please see response to point 1 to reviewer 1. Briefly, we now include new data showing that MT 63-78 significantly enhances the effect of growth inhibition induced by anti-androgens (Biclautamide, MDV3100) or inhibitors of androgen synthesis (CYP17A1 inhibitor Abiraterone) in androgen sensitive LNCaP cells and 3 models of CRPC (22Rv1, C4-2, CL1 cells). We show that MT 63-78 reduces AR expression and combinatorial treatments result in further reduction of AR and PSA expression levels with respect to monotherapy (Figure 9 and Supporting Information Fig 10). Discussion of biologic and clinical relevance of combining AMPK activators with AR pathway inhibitors in CRPC has also been included in the main text of the revised manuscript (Introduction and Discussion).

14. *The potential effects on cell cycle and cell death are interesting but not compelling as shown. These endpoints should be assessed in a broader spectrum of model systems, flow traces shown (for cell cycle), and underlying mechanisms discerned.*

As suggested by the reviewer, we performed cell cycle analyses in a broader spectrum of CRPC cell lines, using shorter time points (24 hrs) with respect to previous data (48/72 hrs). We confirmed MT 63-78-mediated G2-M arrest in all the PCa cell models analysed. We also included results form non-prostate cell lines, including colon (HCT116), thyroid (KTC-1), and stomach (Hs7-16T). In the new version of the manuscript, all cell cycle profiles have been showed (Figure 5A, Supporting Information Fig 7A). Morphological characterization and Hoechst staining of arrested cells allowed us to determine that MT 63-78 indeed induces a mitotic arrest (Figure 5B). Western blot analysis of cell lysates as well as conditioned media from arrested cells confirmed positivity for the mitotic marker phospho-histone H3. In line with this, we observed accumulation of cyclin B1 and increased expression and activity of key mitotic players Aurora kinase A, B, PLK1, involved in mitotic spindle assembly, alignment, chromosome segregation, etc (Figure 5C). Our results suggest that PCa cells, under persistent activation of AMPK with MT 63-78, enter in mitosis but they are not able to undergo a proper cytokinesis program due to arrest before anaphase (most likely at pro-metaphase) due to spindle assembly/alignment abnormalities. These data are in agreement with previous findings by Menendez et al, showing abortive cytokinesis followed by mitotic catastrophe in cancer cells under sustained activation of AMPK (Menendez et al, 2009). Our results are also supported by recent observations from Natalia Scaglia in our lab (co-author of the manuscript) using synchronized HeLa as model system. Her experiments show that increased *de novo* fatty acid synthesis, associated with reduced AMPK activity and ACC phosphorylation, is necessary for HeLa cells to exit mitosis. Citation of these unpublished data has now been included in the Discussion. The role of AMPK in mitosis, its spatial and temporal regulation, as well as mitotic anomalies derived from alterations of its expression/function have been extensively studied using different approaches and model systems (Lee JH et al 2007, Vazquez-Martin A et al, 2009; Banko M et al, 2011, Thaiparambil JT et al, 2012). References to these studies have been included.

We also performed novel experiments showing that MT 63-78-induced mitotic arrest is associated with increased phosphorylation of histone H2AX (γ-H2AX) (marker of DNA damage). These results (Figure 5D) are in keeping with prolonged mitotic arrest inducing telomeric deprotection and activation of DNA damage signalling (Hayashi et al., 2012), resulting in either cell death or skipping cytokinesis and entering the subsequent G1 phase of the cell cycle. In our case, MT 63-78-induced mitotic arrest is associated with activation of the intrinsic apoptotic pathway. As suggested by the reviewer, we performed in depth analysis of apoptosis in a broader spectrum of castration-resistant PCa cell lines (Figure 6A, B). To discern the underlying mechanisms, we evaluated the expression of several pro and anti-apoptotic proteins, which regulate the intrinsic pathway. We observed accumulation of pro-apoptotic Puma, concurrent to reduction of anti-apoptotic Mcl-1. These data are now included as Figure 6A.

15. *Xenograft studies in figure 8 are an important element of the study. The changes seen, however, were not robust. Demonstration of the fact in the second model system, for example using models of castration resistant disease, would enhance enthusiasm for the findings.*

Please see response, points 1 and 2 to reviewer 1.

Referee 3

There is increasing recognition that the LKB1/AMPK axis may provide an interesting therapeutic target in some forms of cancer though, as the authors point out, the use of agents pointing towards this conclusion such as metformin can be criticized given a possible spectrum of off-target effects. The authors propose that MT 68-73 may provide a more selective agent to activate AMPK (by binding to the beta subunit) than A-769662, albeit acting in a similar way at the molecular level. They provide convincing a detailed data describing the effects of the drug on the growth of specific cancer cell types, notably those derived from prostate cancers.

We thank the reviewer for these comments.

16. *MT 68-73 absolutely selective for AMPK beta1 over beta2? For the experiments shown in Fig, 1B and Fig. 2E it would seem important to present the equivalent experiments using/targeting respectively the beta2 complexes.*

We thank the reviewer for pointing out this aspect. To address it, we performed the knock down of AMPK b2 subunit in LNCaP and PC3 cells (as previously done for the b1 subunit) and we measured AMPK activity and phosphorylation of ACC in b2 subunit-silenced cells under treatment with MT 63-78 (Supporting Information Fig 4B).

As described above, we also performed *in vitro* experiments with four specific recombinant heterotrimers of AMPK (a1b1g1, a2b1g1, a1b2g1, a2b2g1) and we assessed AMPK activity under MT 63-78 treatment by measuring ACC phosphorylation as readout (alpha screen technology) (see response point 11 to reviewer 2).

Our results demonstrate that while high concentrations of MT 63-78 are able to induce a moderate activation of b2-containing heterotrimers (a2b2g1) in *in vitro* assays, silencing experiments in PCa cell models still suggest that MT 63-78 activates AMPK mainly by binding the b1 subunit in a cell-based context. (Figure 2F, Supporting Information Fig 4B)

17. *The identification of a gene signature activated by FASN expression seems rather redundant Whether tumours with a particularly elevated lipogenic gene signature would be more or less susceptible to treatment is also not completely clear…this part of the manuscript feels unfinished and should transferred to the Supplementary section or removed altogether.*

We appreciate the comments and the suggestions of the Reviewer, shared also by the Editor. Thus, we removed the gene signature from the revised version of the manuscript.

18. *The experiments shown in Fig. 1B are convincing but one wonders how the potency of the new drug compares to that of A-769662 in this assay?*

By using four specific AMPK recombinant complexes (a1b1g1, a2b1g1, a1b2g1, a2b2g1) and ACC phosphorylation as readout for AMPK activity in alpha screen assays, we observed that the *in vitro* potency of A-769662 is superior in β1 subunit-containing heterotrimers (a1b1g1, a2b1g1), whereas the *in vitro* potency of MT 63-78 is superior in β2 subunit-containing heterotrimers (Supporting

Information Fig 4A). However, in PCa cell-based context the potency of MT 63-78 is far superior than A-769662 (Supporting Information Fig 1B). See also answer provided for point 11 to reviewer 2.

19. *MT 63-78 is a highly hydrophobic molecule which may serve as a protonophore and mitochondrial uncoupler to impair ATP synthesis. This appears not to be a major problem (Fig. 2C) but one would like to be further reassured by measurements of intracellular ADP or AMP in addition to ATP. I note that the kit used purportedly measures ATP/ADP ratio rather than total ATP? Please clarify.*

The assay we performed with the EnzyLight ™ADP/ATP Ratio Assay Kit (ELDT-100, BioAssay Systems) involves two steps. The first step consists in the measurement of intracellular ATP levels, which we report as Figure 2C. The second step allows establishing the ratio ADP/ ATP. We now confirmed the same results using the EnzyLight ™ATP Assay Kit (EATP-100, BioAssay Systems), which only measures ATP levels (not shown). As requested by the reviewer, we also measured intracellular ADP levels in LNCaP and PC3 cells treated with the compound using ADP Assay Kit (EADP-100, BioAssay Systems). We include these new data as Figure 2D. Our data confirmed no significant changes in intracellular ADP levels at the concentrations of the compound we used in the study. Moreover, we measured AMP, ADP, ATP levels by HPLC in HepG2 cells, and we calculated AMP/ATP and ADP/ATP ratios, confirming that there is no change of these ratios at drug concentrations used in the study. Indeed, we only observed significant decrease in intracellular ATP and increase in AMP and ADP levels (as well as AMP/ATP and ADP/ATP ratios) when the concentration of MT 63-78 was increased to 200uM (Supporting Information Fig 2).

20. *Inhibition of MST-1, a major executor of apoptosis via Hippo signalling, may be problematic in the context of cancer treatments. Comments?*

We thank the reviewer for pointing out this aspect. We know that Akt (which is frequently activated/ mutated in prostate cancer) can inhibit MST-1 resulting in the reduction of the execution of hippo signaling-mediated apoptosis. We tested MT 63-78 in LNCaP and PC3 cells and our data showed that, in contrast to staurosporine, MT 63-78 induces apoptosis without the concomitant activation of MST-1, suggesting that the hippo signaling pathway is not playing a relevant role in mediating the induction of apoptosis by MT 63-78. We did not include these data in the revised version of the manuscript for space limitation; however we are ready to provide them, if requested.

21. *"In contrast to AICAR and metformin, the growth inhibitory effect of MT 63-78 was strictly dependent on the expression of AMPK (Fig4C)." Data for the effects of AICAR and metformin appear to be missing and should be included or the appropriate literature cited.*

We apologize for how the sentence was formulated in the original version of the paper. We meant to say that the growth inhibitory effect of MT 63-78 was dependent on the intact activity of AMPK. Indeed, we measured the cell growth rate of AMPK $\alpha1^{-/-}$ and $\alpha2^{-/-}$ MEFs (to mimic the absence of catalytic activity of the kinase) and MEFs wt under treatment with MT 63-78, AICAR, and metformin for 72 hrs. We observed that metformin and AICAR were still able to inhibit AMPK $\alpha1^{-/-}$ and $\alpha2^{-/-}$ cell growth, whereas MT 63-78 did not. Thus, these data provide evidence that the catalytic activity of AMPK is required for the anti-growth effect of MT 63-78 (current Figure 4C).

Our results are also in line with data in the literature. It has been demonstrated that AICAR and metformin induce AMPK- independent cell cycle arrest in S-phase and G0/G1 phase of cell cycle, respectively in prostate cancer cells (Ben Sahra I et al. 2008; Park HU et al., 2009). Moreover, recent papers reported AMPK-independent apoptosis induced by AICAR in AMPK $\alpha1^{-/-}$ and $\alpha2^{-/-}$ MEFs (González-Gironès DM et al., 2013) and in several types of cancers including chronic lymphocytic leukemia (Santindrian AF et al., 2010) and breast (Garcia-Garcia C et al, 2010). These manuscripts have now been cited.

22. *The paper is over-long. Removal of the FASN signature data may help but I suspect further*

pruning by 10 % overall could easily be achieved.

As suggested by both the reviewer and the Editor, FASN signature data have been removed in the revised version of the manuscript.

We thank the reviewers and the Editor for their thoughtful comments. We believe our exhaustive response to these has greatly improved the manuscript, which is now extensively revised.

We look forward to hearing from you.

3rd Editorial Decision 02 December 2013

Thank you for the submission of your revised manuscript to EMBO Molecular Medicine. We have now received the enclosed reports from the referees that were asked to re-assess it. As you will see the reviewers are now supportive and I am pleased to inform you that we will be able to accept your manuscript pending the following final amendments:

1) We would need a short list (up to 5) of bullet points that summarize the key NEW findings. The bullet points should be designed to be complementary to the abstract and will be used online in our new platform (coming January 2014).

2) I would like to try to make the title a bit more impactful. For instance, what do you think of the following alternatives "Direct targeting of the energy sensor AMPK inhibits prostate cancer growth by blocking the lipogenic switch" or "A new compound targeting AMPK inhibits prostate cancer growth by blocking the lipogenic switch" (it would be nice to mention the new compound in the title...)? Would you care to suggest an alternative(s) yourself?

Finally, we are now encouraging the publication of source data, particularly for electrophoretic gels and blots, with the aim of making primary data more accessible and transparent to the reader. Would you be willing to provide a PDF file per figure that contains the original, uncropped and unprocessed scans of all or at least the key gels used in the manuscript? The PDF files should be labeled with the appropriate figure/panel number, and should have molecular weight markers; further annotation may be useful but is not essential. The PDF files will be published online with the article as supplementary "Source Data" files. If you have any questions regarding this just contact me.

Please submit your revised manuscript within two weeks. I look forward to seeing a revised form of your manuscript as soon as possible.

***** Reviewer's comments *****

Referee #1 (Comments on Novelty/Model System):

this revision adequately addresses concerns raised in the initial review and in my opinion is significantly improved and acceptable for publication in EMBO Molecular Medicine

Referee #2 (Comments on Novelty/Model System):

The revised study addressed the major concerns of the previous critique, including the addition of new models. The study should be of impact to the prostate cancer field, and will be well cited.

Referee #3 (Remarks):

The authors have dealt thoroughly with the questions raised. No further issues.
