# Supplementary figures and images for: A novel direct activator of AMPK inhibits prostate cancer growth by blocking lipogenesis
Source: EMBO Mol Med. 2014 Feb 4;6(4):519–38. doi: 10.1002/emmm.201302734 (PMC3992078; doi:10.1002/emmm.201302734)

FIGURE 1 panel C

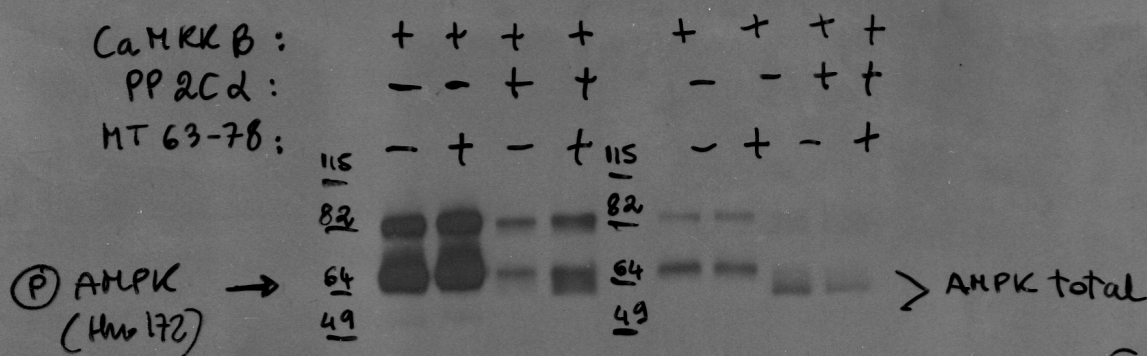

Exposure 5 sec  
ECL  
08/18/2012

Supplement: Supplementary file 2 [file emmm0006-0519-sd2.pdf]
