# Supplementary material for: A novel direct activator of AMPK inhibits prostate cancer growth by blocking lipogenesis
Source: EMBO Mol Med. 2014 Feb 4;6(4):519–38. doi: 10.1002/emmm.201302734 (PMC3992078; doi:10.1002/emmm.201302734)
Supplement: Supplementary file 3 [file emmm0006-0519-sd3.pdf]

FIGURE 2 PANEL A
LEFT (LNCaP cells)

②

- Exposure for:
a) phospho - Raptor
b) phospho - AMPK
c) Raptor total
d) AMPK total

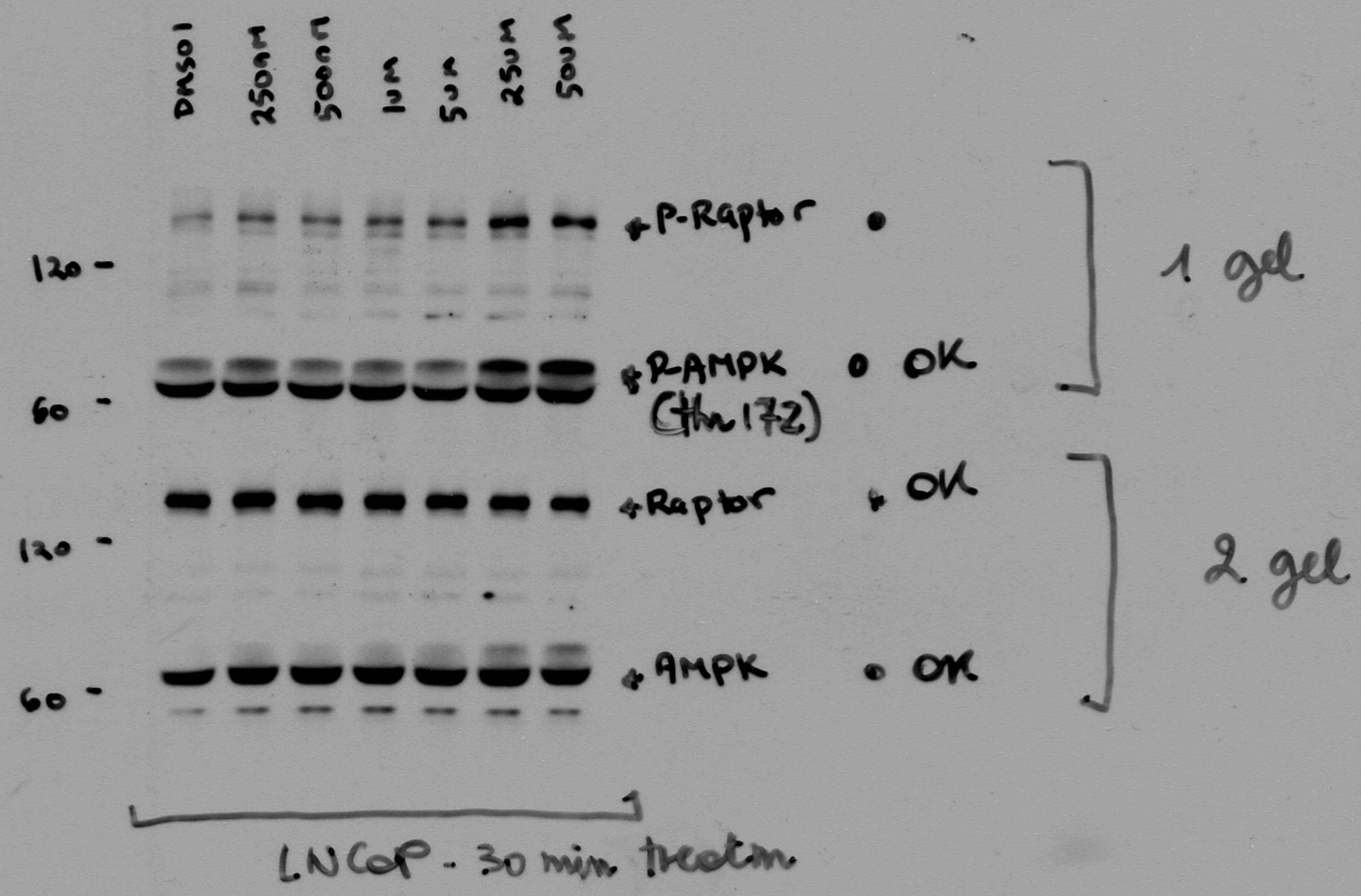

LN: 63-78 × 30 min - 30ug

7/22/2009 samples

Samples were loaded in duplicate in 2 gels, run and transfered together

exp 2.5 min
ELL
6.29.10

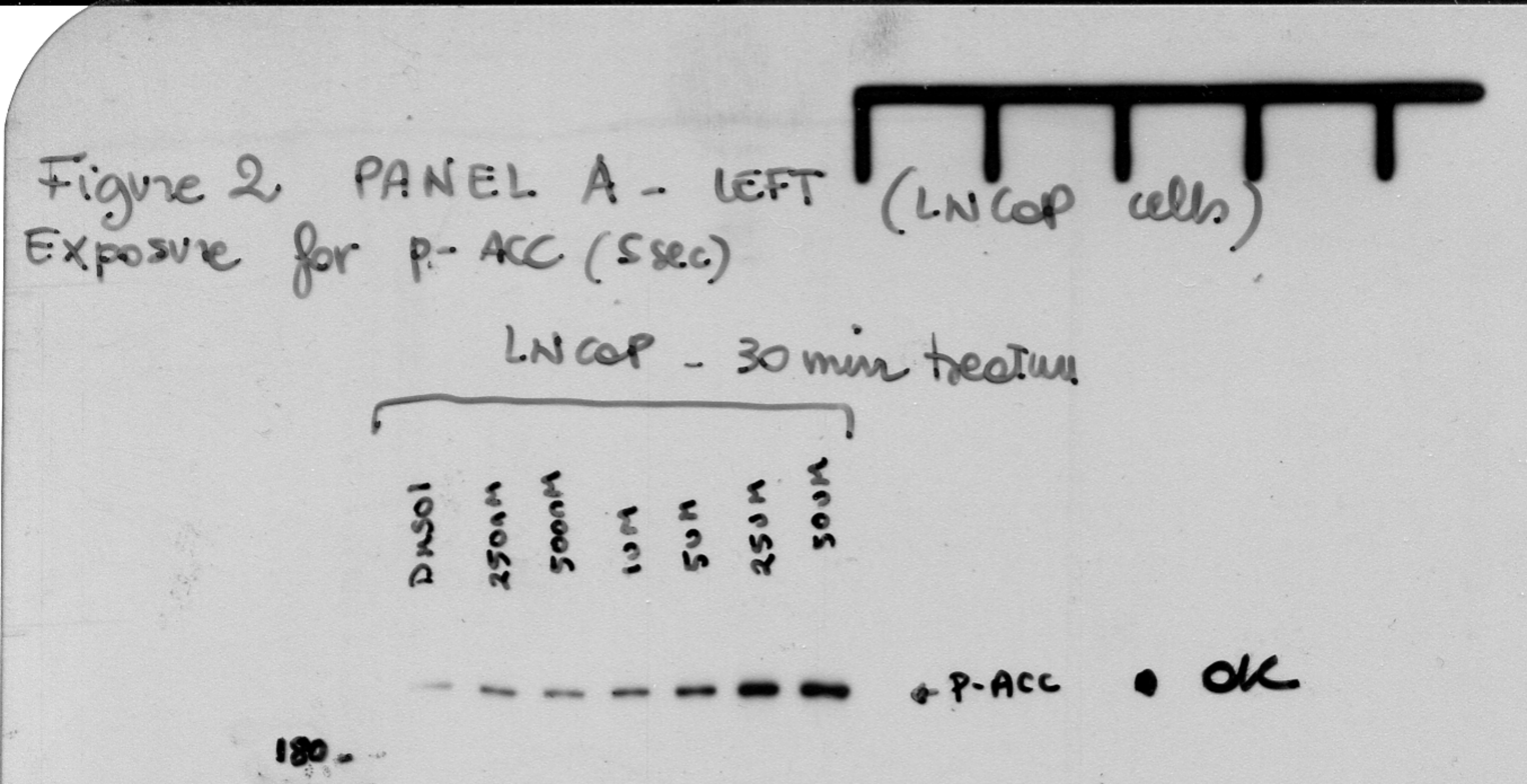
Figure 2 PANEL A - LEFT (LNCaP cells)
Exposure for p-ACC (5 sec)
LNCaP - 30 min treatment
DMSO
250nM
500nM
1uM
5uM
25uM
50uM
P-ACC
OK
180

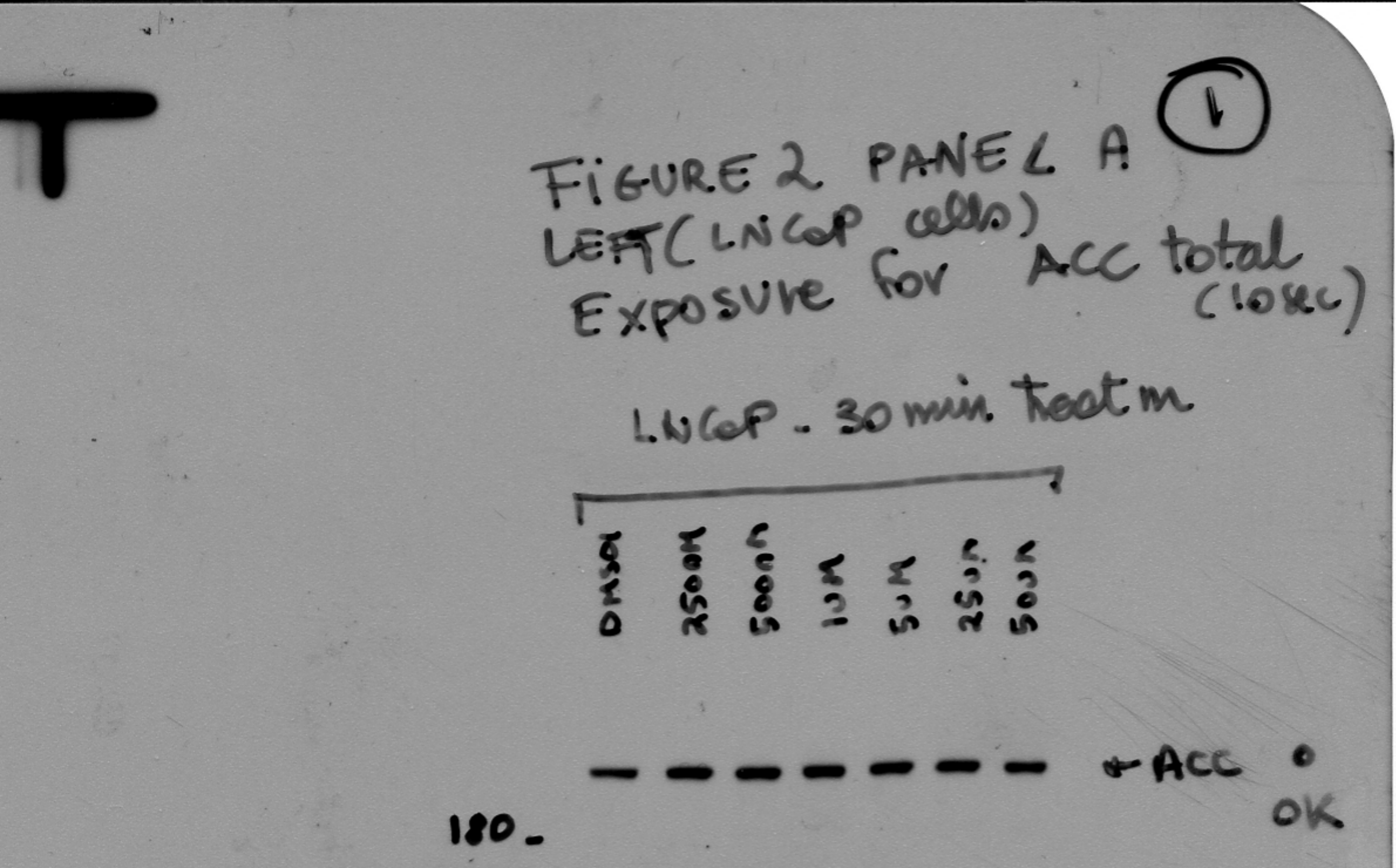
1
FIGURE 2 PANEL A
LEFT (LNCaP cells)
Exposure for ACC total
(10sec)
LNCaP - 30 min Treatm
DMSO
250nM
500nM
1uM
5uM
25uM
50uM
← ACC
OK
180 -

FIGURE 2 PANEL A- LEFT (LNCaP cells)

LNCaP - 30 min treatm

6

Exposure for VINCULIN (15 sec) 6.30.10

DMSO | 250nM | 500nM | 1uM | 5uM | 25uM | 50uM

120 -

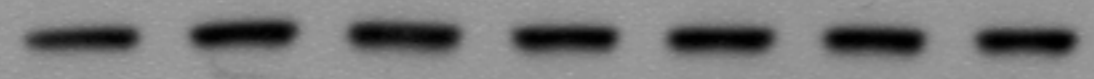

VINC (Rap Mem) • ovc

FIGURE 2 PANEL A - RIGHT (PC3 cells)

Exposure used for P-ACC (30 sec)

12.9.10

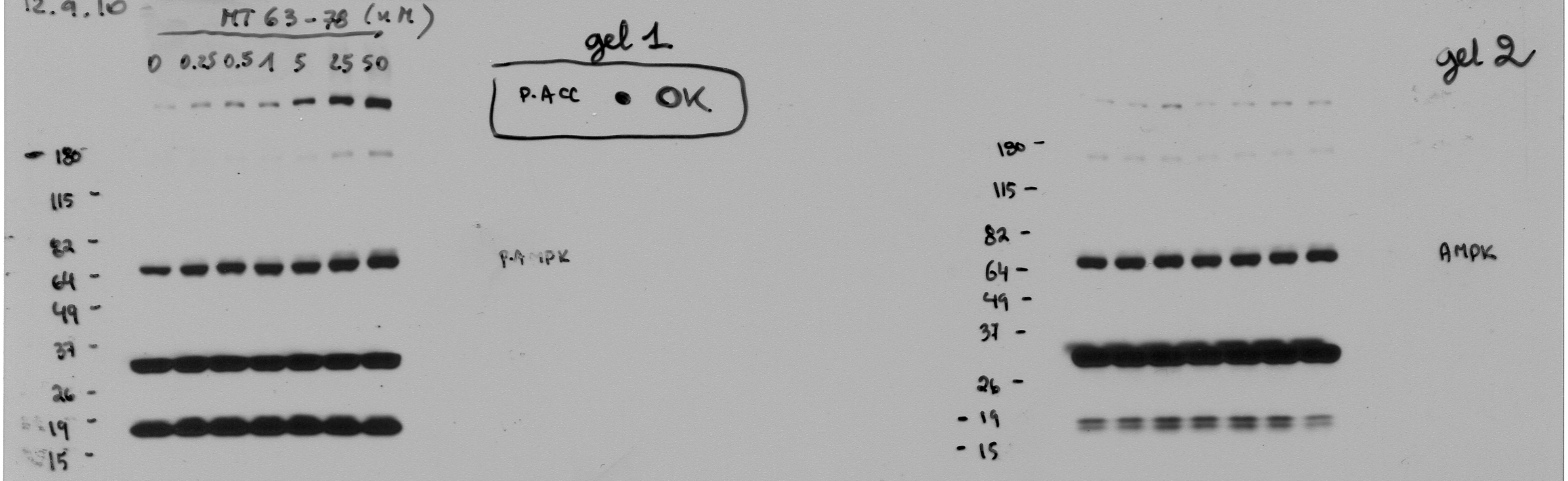

Samples were prepared in duplicate and loaded on 2 gels (gel 1, 2)

30 sec
ECL
12.9.10

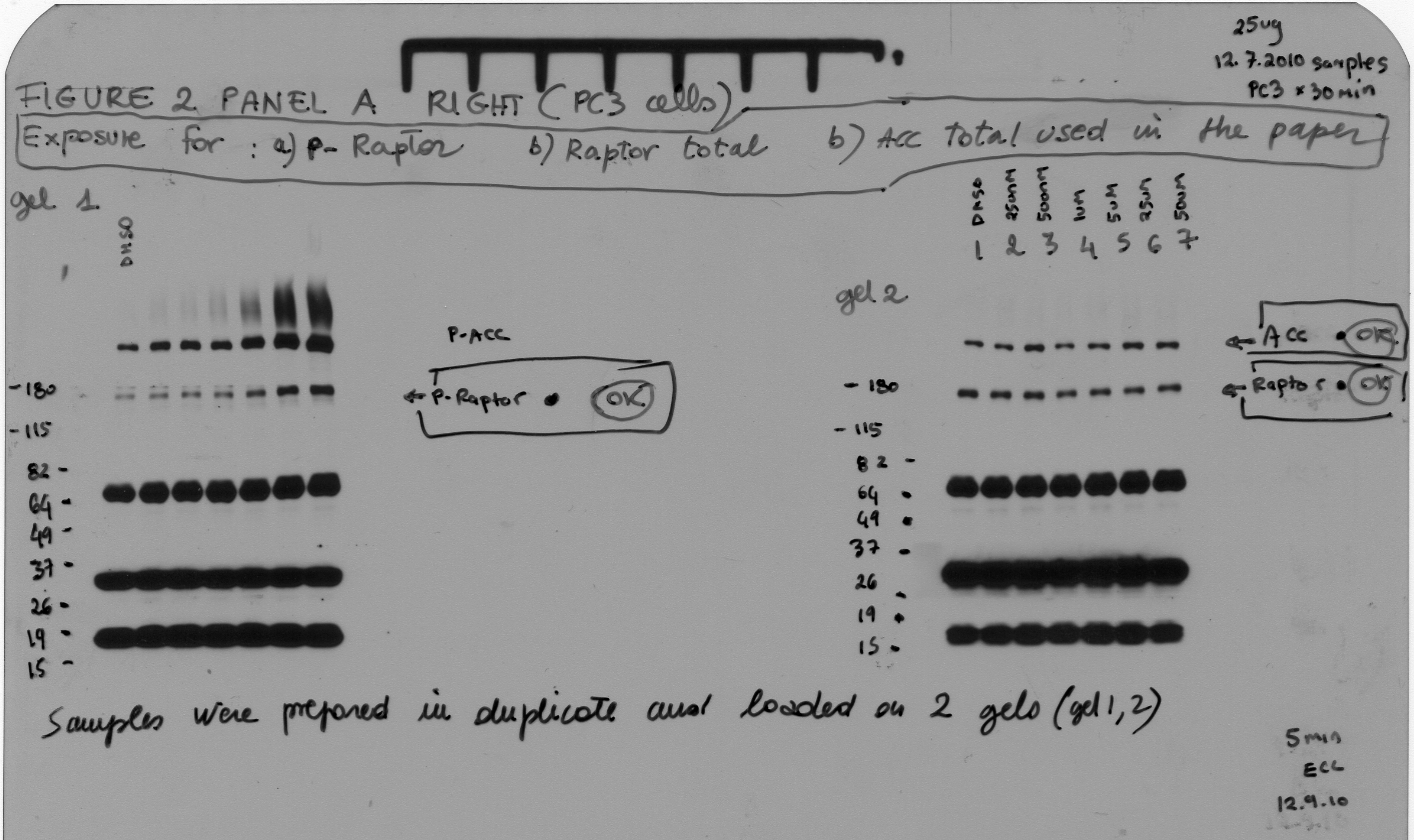

25ug
12.7.2010 samples
PC3 x 30 min
FIGURE 2 PANEL A RIGHT (PC3 cells)
Exposure for : a) P- Raptor b) Raptor total b) Acc total used in the paper
gel 1.
DMSO
P-ACC
P-Raptor • OK
-180
-115
82
64
49
37
26
19
15
DMSO 25nM 50nM 1uM 5uM 25uM 50uM
1 2 3 4 5 6 7
gel 2
Acc • OK
Raptor • OK
-180
-115
82
64
49
37
26
19
15
Samples were prepared in duplicate and loaded on 2 gels (gel 1, 2)
5 min
ECL
12.9.10

25ug
12.7.10 samples
PC3 x 30min

FIGURE 2 PANEL A RIGHT (PC3 cell).

Exposure for: • P-AMPK
• AMPK Tot
(10 sec)

12.9.10

Legend
1 = DMSO
2 = 250 nM MT63-78
3 = 500 nM MT63-78
4 = 1 µM MT63-78
5 = 5 uM MT63-78
6 = 25 uM MT63-78
7 = 50 uM MT63-78

gel 1

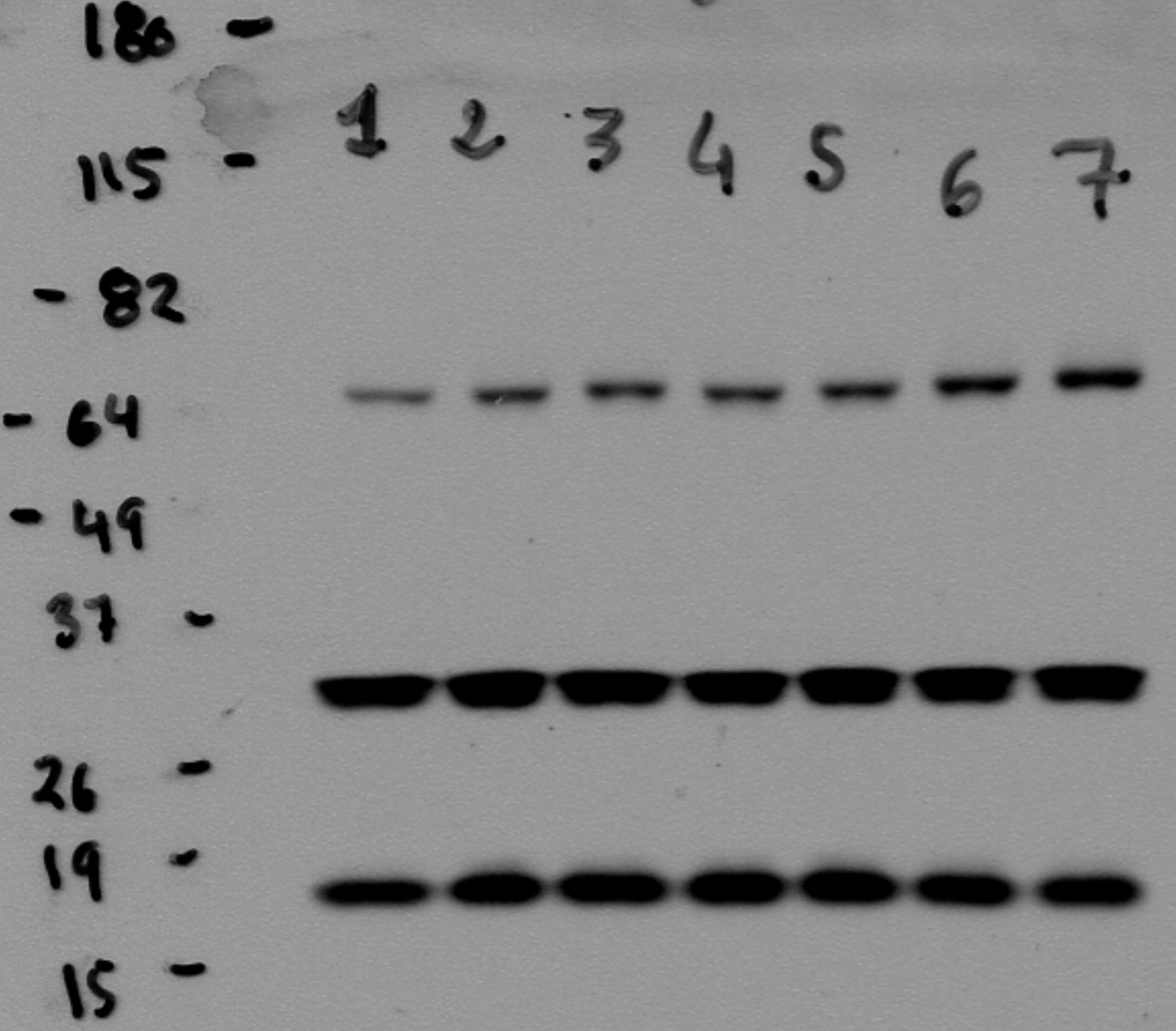

P-AMPK • (3) OK

gel 2

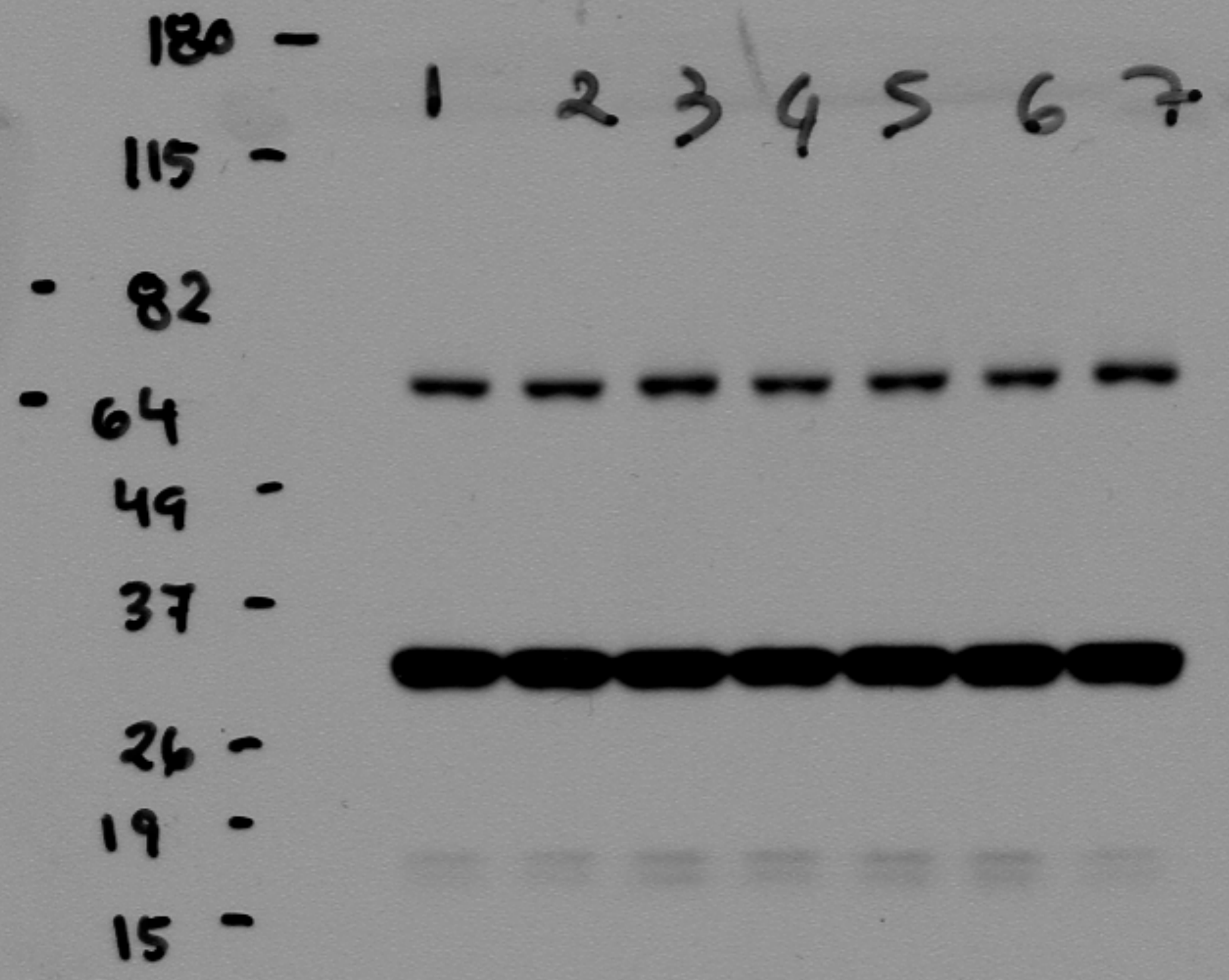

AMPK • OK

Samples were prepared in duplicate and loaded on 2 gels (gel 1, 2)

10 Sec (2nd)
ECL
12.9.10

MT 63-78

PC3 - 30 min treatment

0 | 250 nM | 500 nM | 1 uM | 5 uM | 25 uM | 50 uM

180

115

180

115

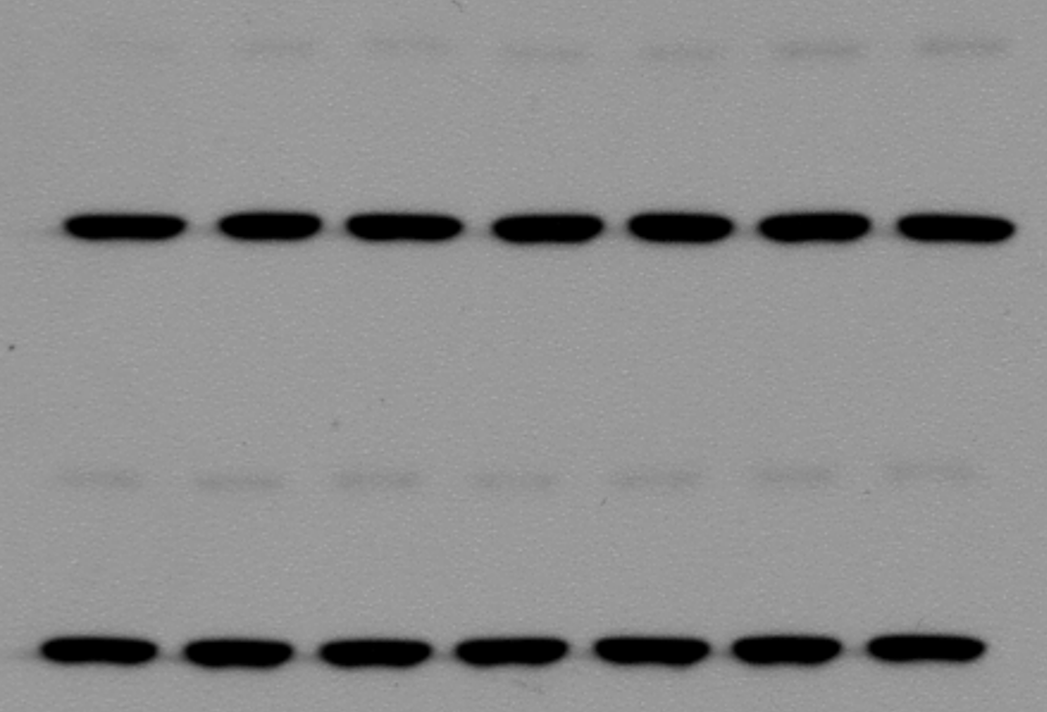

← VINCULIN (on p-Raptor membrane) gel 1

← VINCULIN (on Raptor membrane) • OK gel 2

1 2 3 4 5 6 7

Re-blot

5 sec ECL

12/10/2010

FIGURE 2 PANEL A RIGHT (PC3 cells)

Exposure for VINCULIN used in the paper (5sec)

LEGEND:

1 = DMSO (0uM 63-78)
2 = 250 nM MT63-78
3 = 500 nM " "
4 = 1 µM " "
5 = 5 uM " "
6 = 25 uM " "
7 = 50 uM " "

Samples were prepared in duplicate and loaded on 2 gels (gel 1,2)

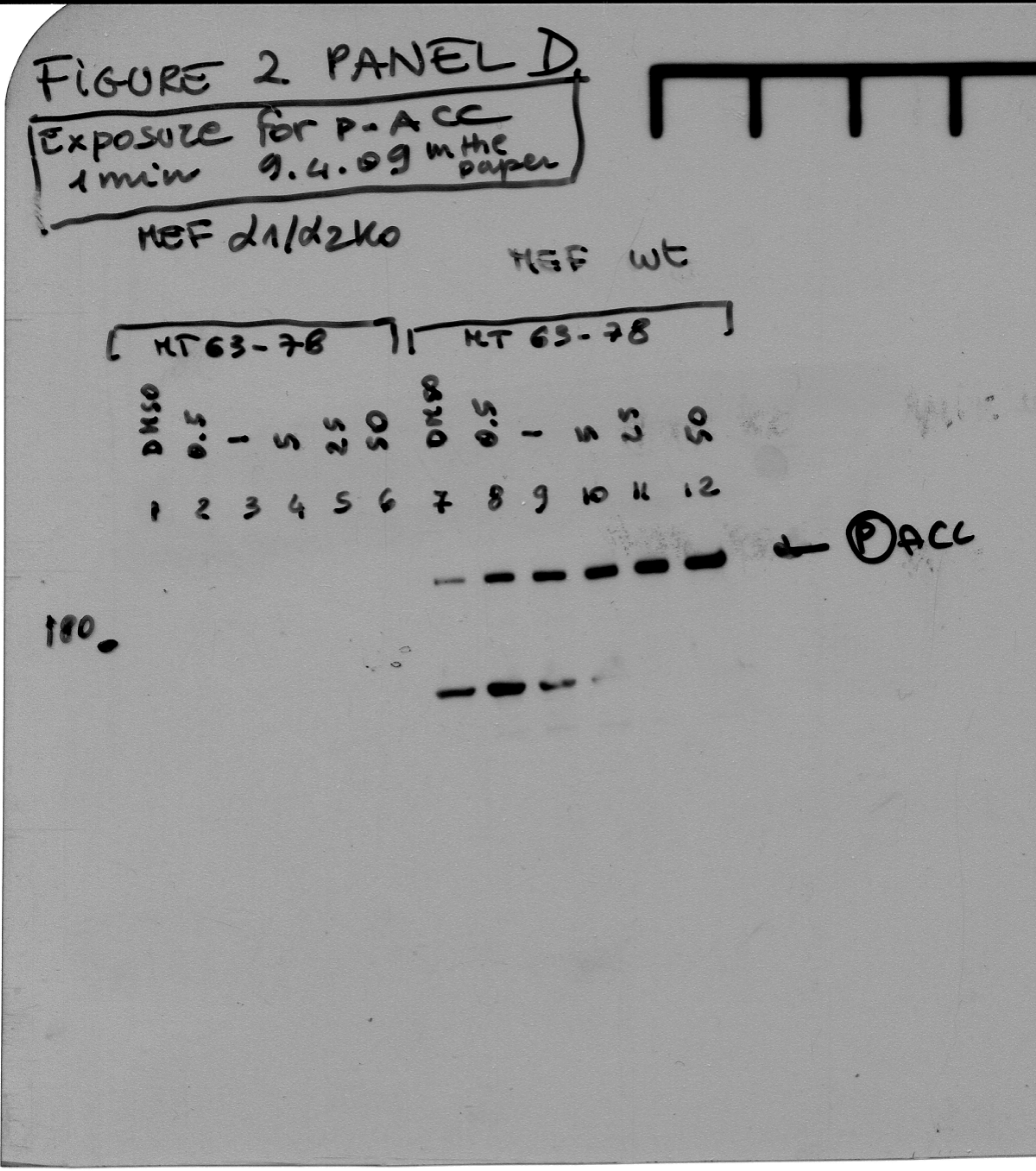
FIGURE 2 PANEL D
Exposure for p-ACC
1 min 9.4.09 in the paper
MEF α1/α2 KO
MEF wt
MT 63-78
MT 63-78
DMSO 0.5 1 5 25 50
DMSO 0.5 1 5 25 50
1 2 3 4 5 6 7 8 9 10 11 12
Ⓟ ACC
180

FIGURE 2 PANEL D

Exposure for ACC total (10 sec) 9.4.09 in the paper

MEF d1/d2 KO — MEF wt

DMSO	500nM	1uM	5uM	25uM	50uM	DMSO	500nM	1uM	5uM	25uM	50uM	MT63-78
1	2	3	4	5	6	7	8	9	10	11	12	

ACC ← [illegible]0K

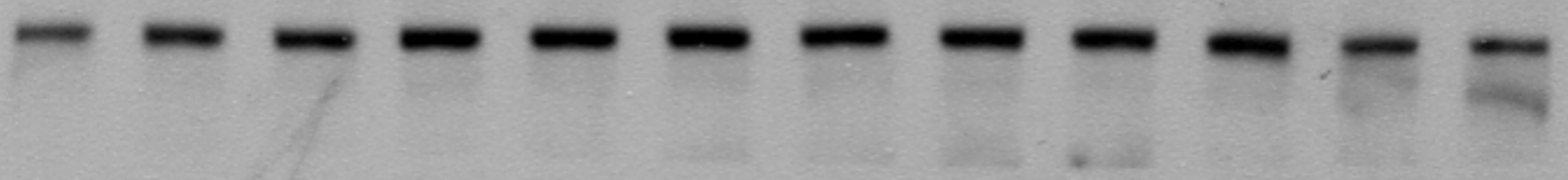

180 –

FIGURE 2 PANEL D

Exposure for
Ⓟ RAPTOR used
in the popn (8 min)
9.04.09

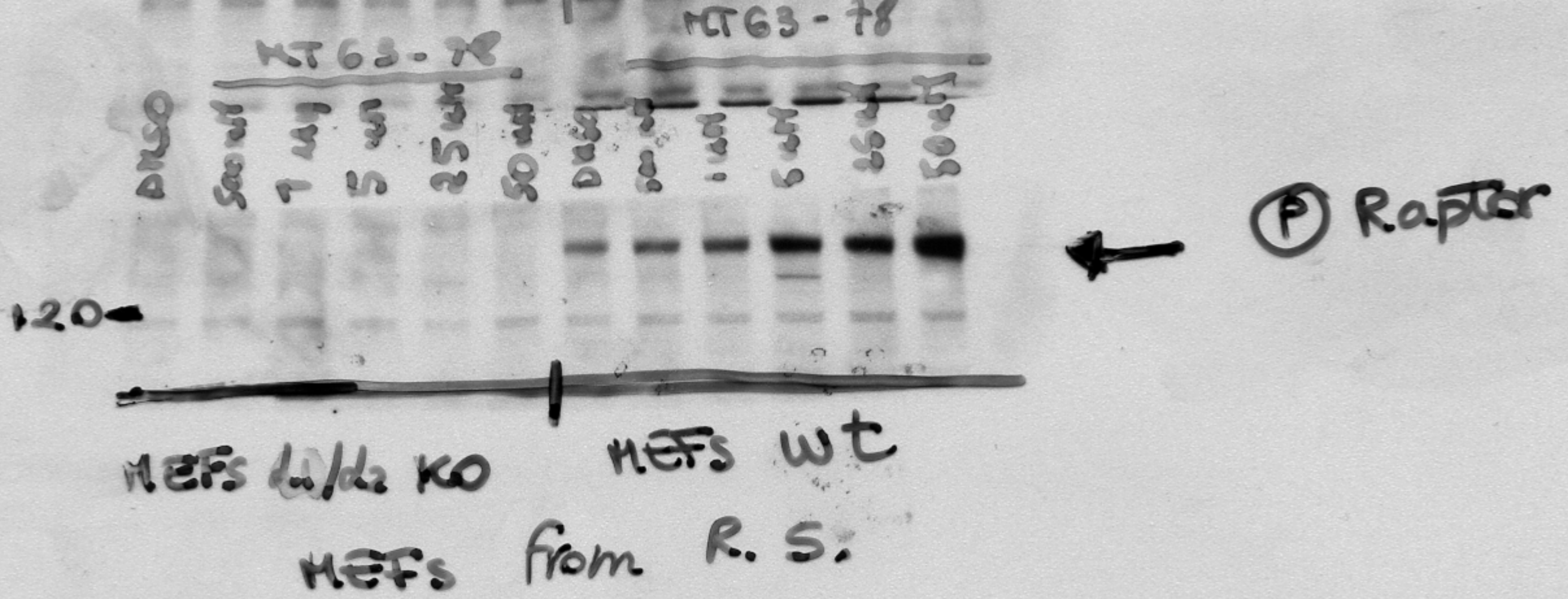

FIGURE 2 PANEL D

Exposure for RAPTOR TOTAL (1 min) used in the paper

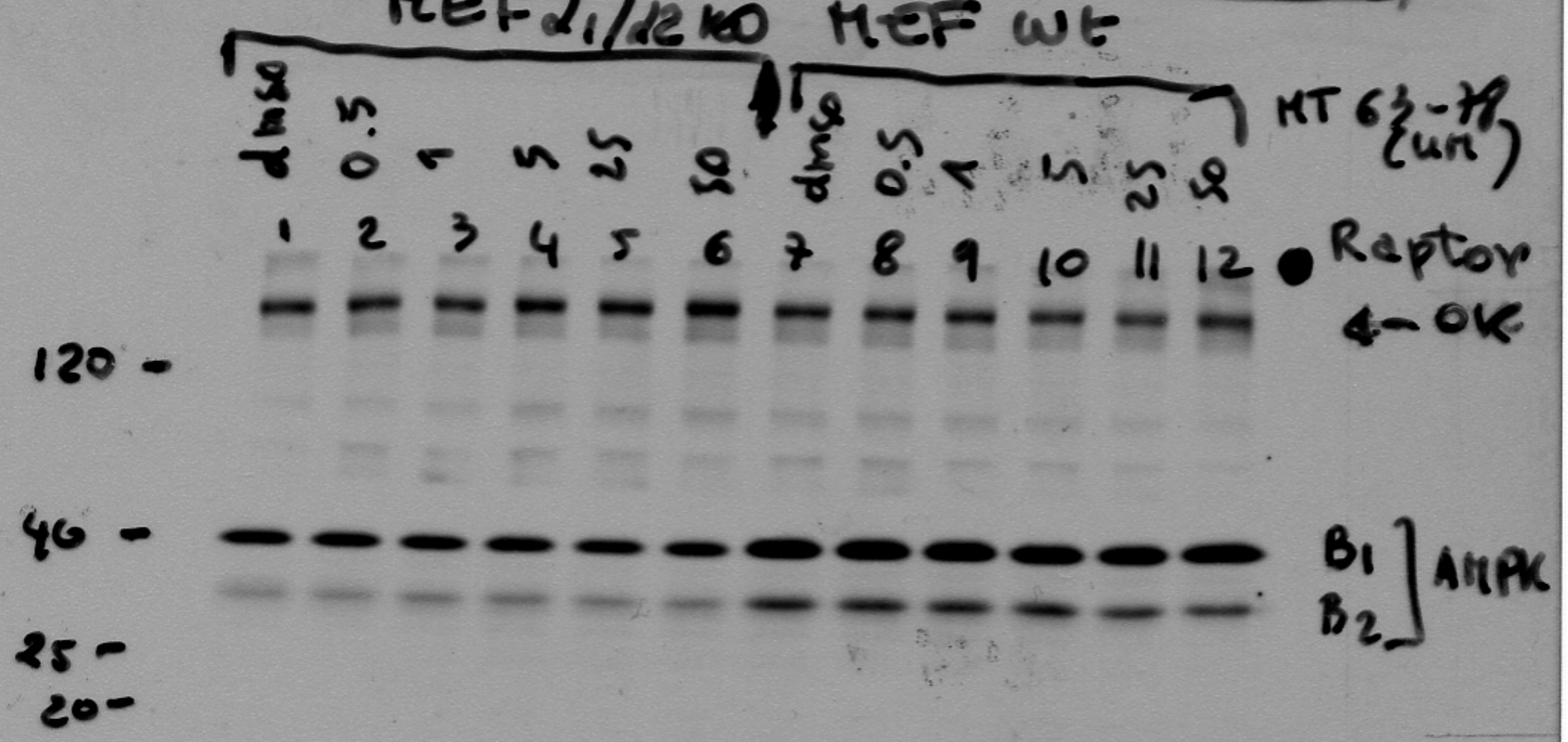

FIGURE 2 PANEL D

Exposure for P-AMPK 30sec

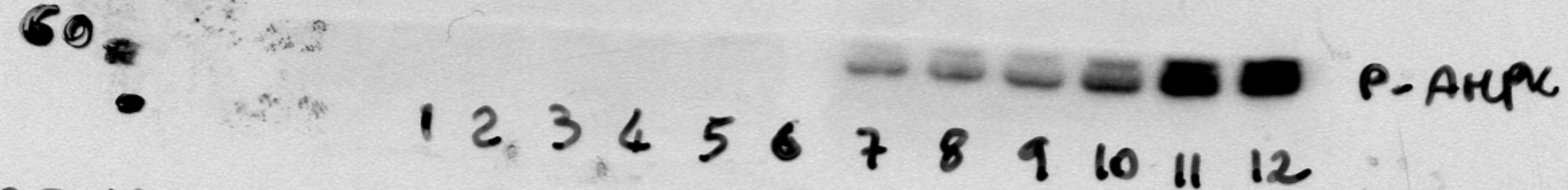

LEGEND = 1 = 0 uM MT63-78
2 = 0.5 uM 〃
3 = 1 uM 〃
4 = 5 uM 〃
5 = 25 uM 〃
6 = 50 uM 〃

MEF d1/d2 KO

7 = 0 uM MT63-76
8 = 0.5 uM 〃
9 = 1 uM 〃
10 = 5 uM 〃
11 = 25 uM 〃
12 = 50 uM 〃

MEF wt

FIGURE 2 PANEL D

Exposure for AMPK total in the paper
(10sec) 9.4.09

MT63.78 x 30min

MEF α1/α2 KO — MEF WT

DMSO	500nM	1μM	5μM	25μM	50μM	DMSO	500nM	1μM	5μM	25μM	50μM
1	2	3	4	5	6	7	8	9	10	11	12

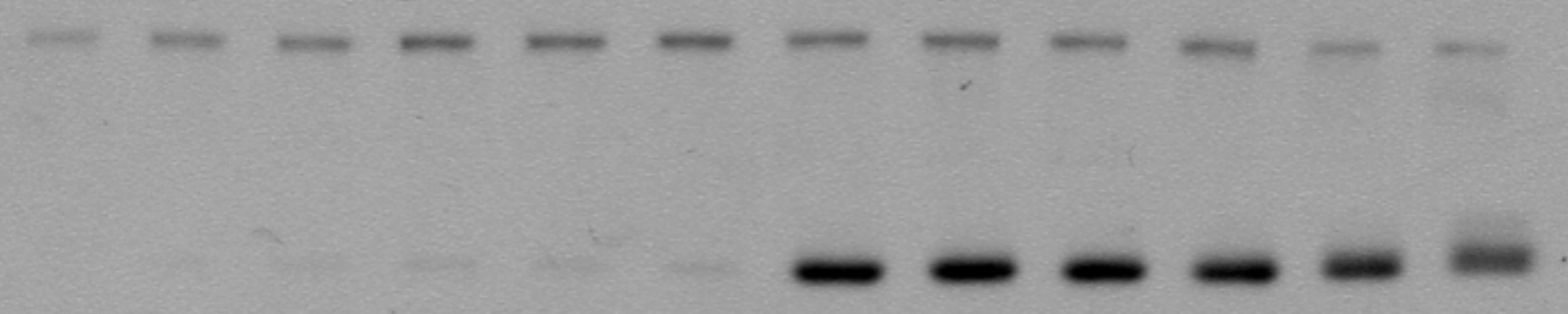

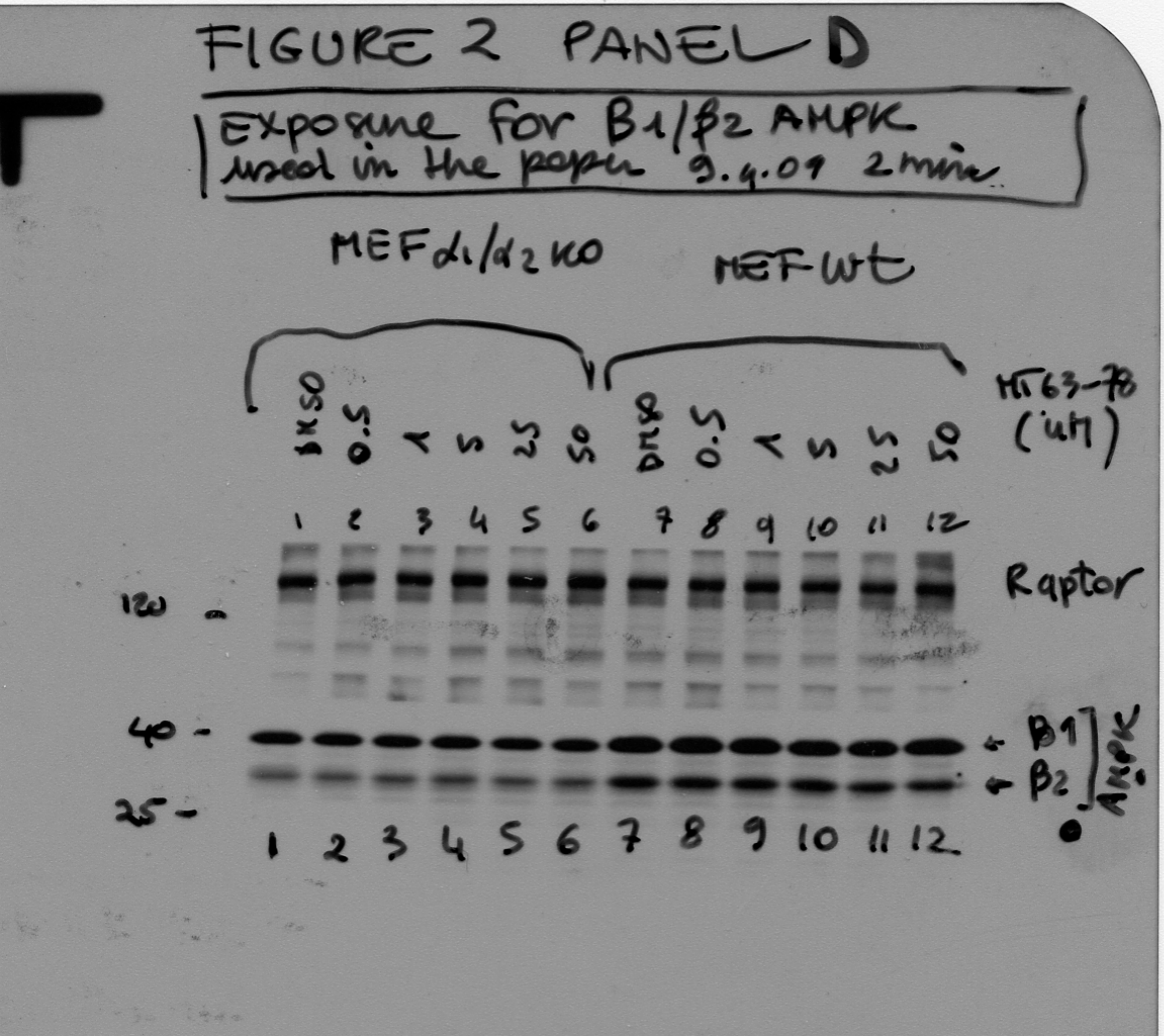
FIGURE 2 PANEL D
Exposure for β1/β2 AMPK
used in the paper 9.4.09 2 min
MEF α1/α2 KO
MEF wt
MT63-78 (uM)
DMSO 0.5 1 5 25 50 DMSO 0.5 1 5 25 50
1 2 3 4 5 6 7 8 9 10 11 12
Raptor
120
40
25
β1
β2
AMPK
1 2 3 4 5 6 7 8 9 10 11 12

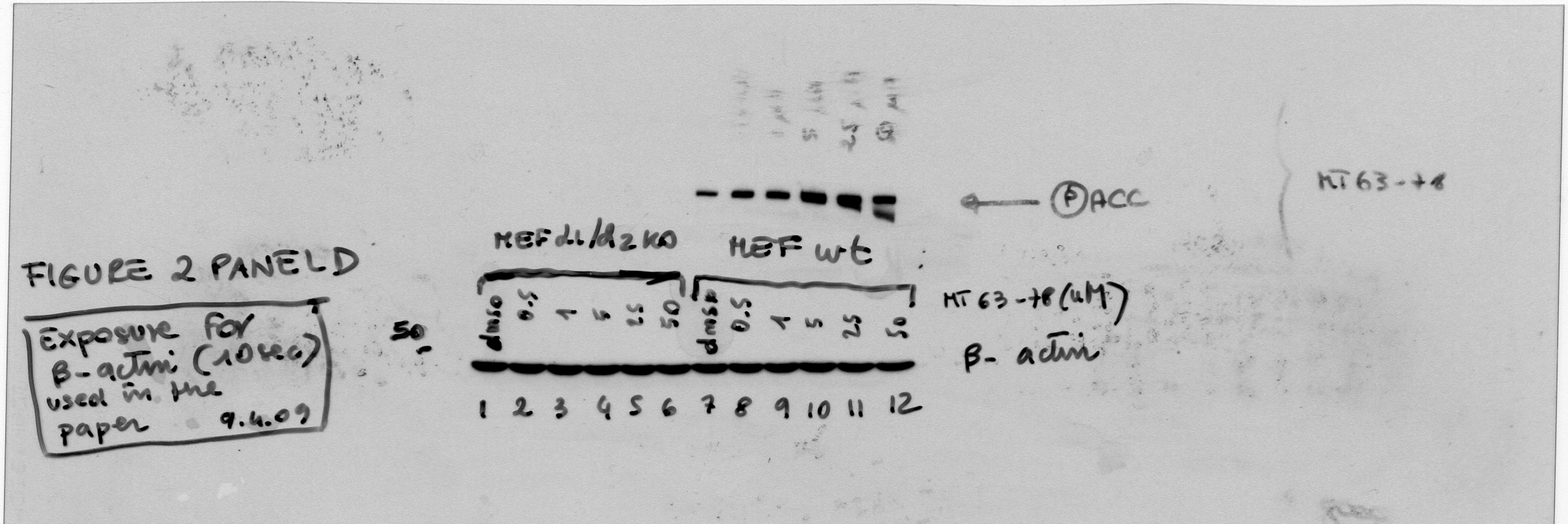
FIGURE 2 PANEL D
Exposure for β-actin (10 sec) used in the paper 9.4.09
MT 63-78
P ACC
MEF d1/d2 KO
MEF wt
MT 63-78 (uM)
50
dmso 0.5 1 5 25 50
dmso 0.5 1 5 25 50
β- actin
1 2 3 4 5 6 7 8 9 10 11 12
