# Supplementary material for: A novel direct activator of AMPK inhibits prostate cancer growth by blocking lipogenesis
Source: EMBO Mol Med. 2014 Feb 4;6(4):519–38. doi: 10.1002/emmm.201302734 (PMC3992078; doi:10.1002/emmm.201302734)
Supplement: Supplementary file 4 [file emmm0006-0519-sd4.pdf]

FIGURE 3 PANEL B-LEFT (Hela)

Hela : 63-78 x 30 min

gel 1

DMSO | 500nM | 1uM | 5uM | 10uM | 25uM | 50uM

Exposure for P-ACC used in the paper (2.5 min) 11.5.10

P-ACC ok

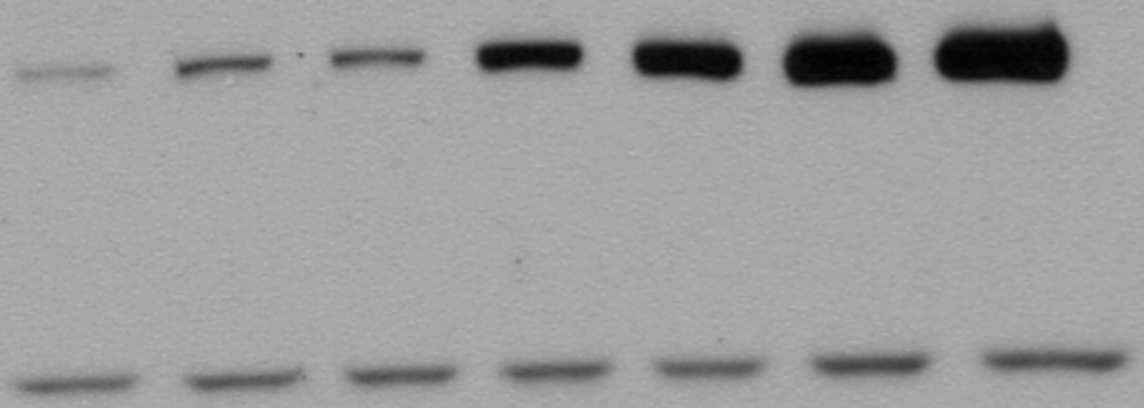

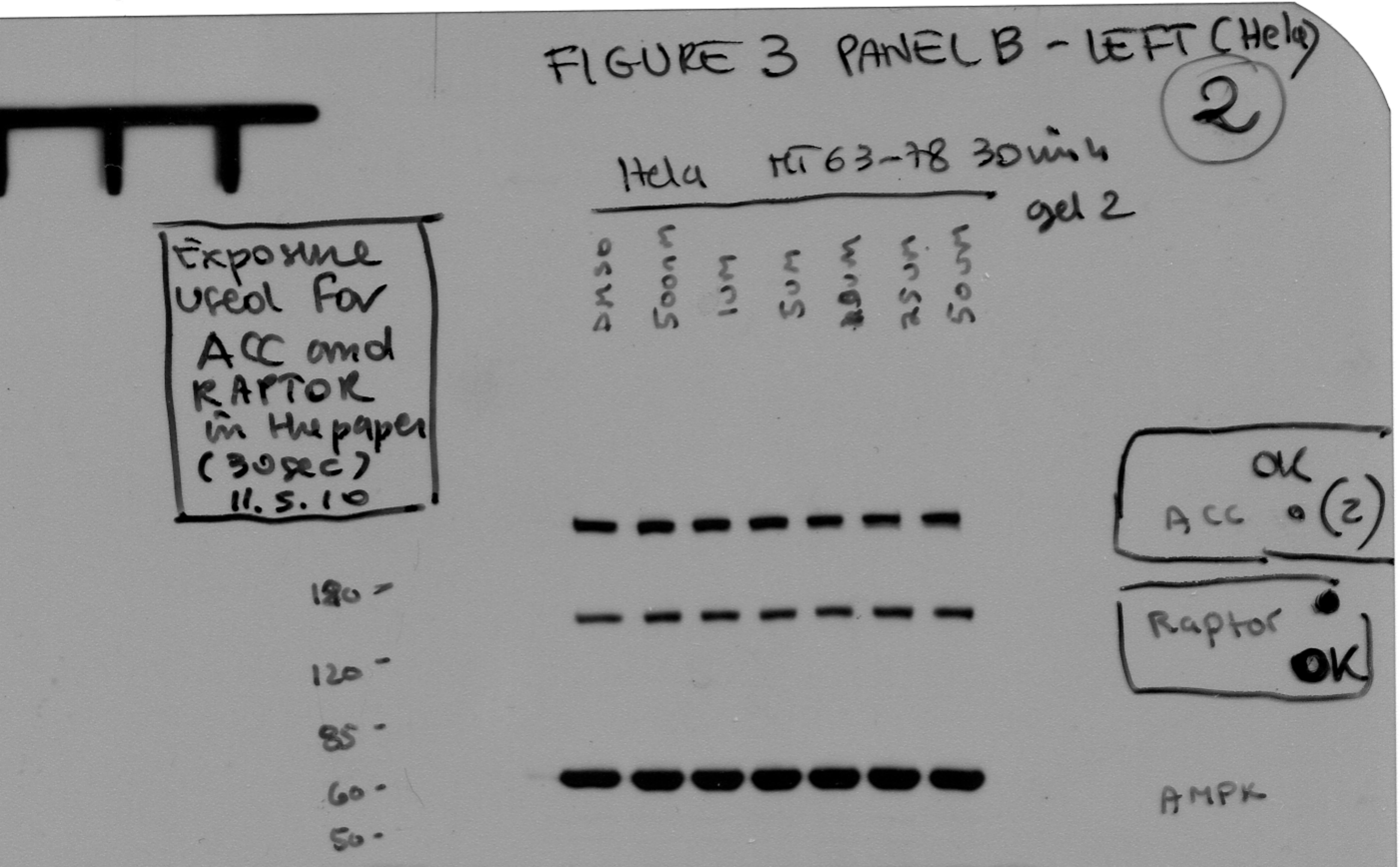
FIGURE 3 PANEL B - LEFT (Hela)
2
Hela KT 63-78 30 min
gel 2
DMSO
500nM
1uM
5uM
10uM
25uM
50uM
Exposure used for ACC and RAPTOR in the paper (30 sec) 11.5.10
OK
ACC (2)
Raptor
OK
AMPK
180
120
85
60
50

FIGURE 3 PANEL B
LEFT (HeLa cells)

Exposure for
Ⓟ Raptor
used in the
paper (3 min)
11.6.2010

1 2 3 4 5 6 7

Ⓟ Raptor →

180
120

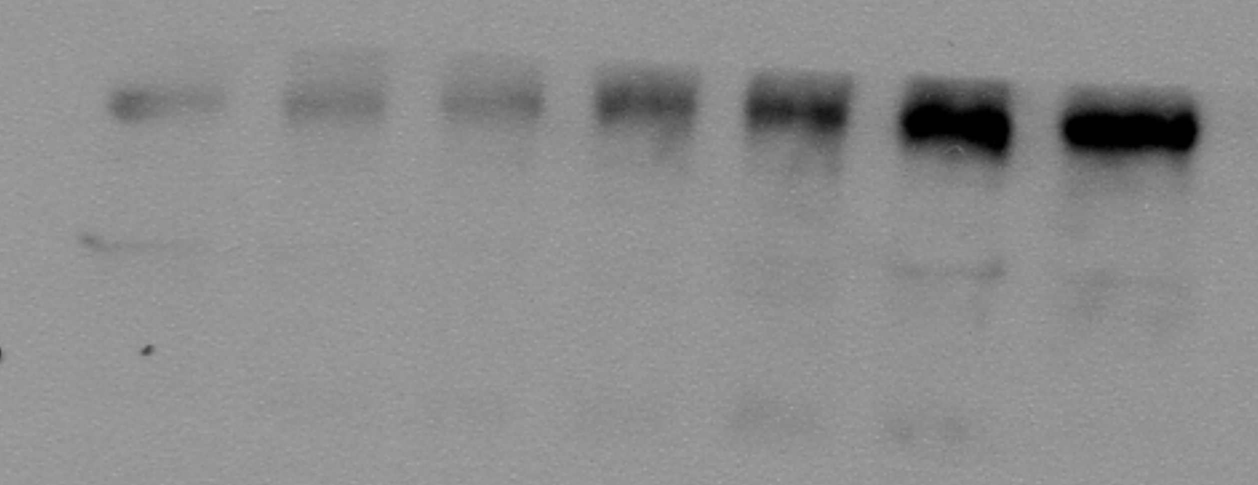

• HeLa
ok

1 = 0
2 = 250 nM
3 = 500 nM
4 = 1 uM
5 = [illegible] uM
6 = 25 uM
7 = 50 uM

MT 62.38

ECL
3 min
11/6/2010

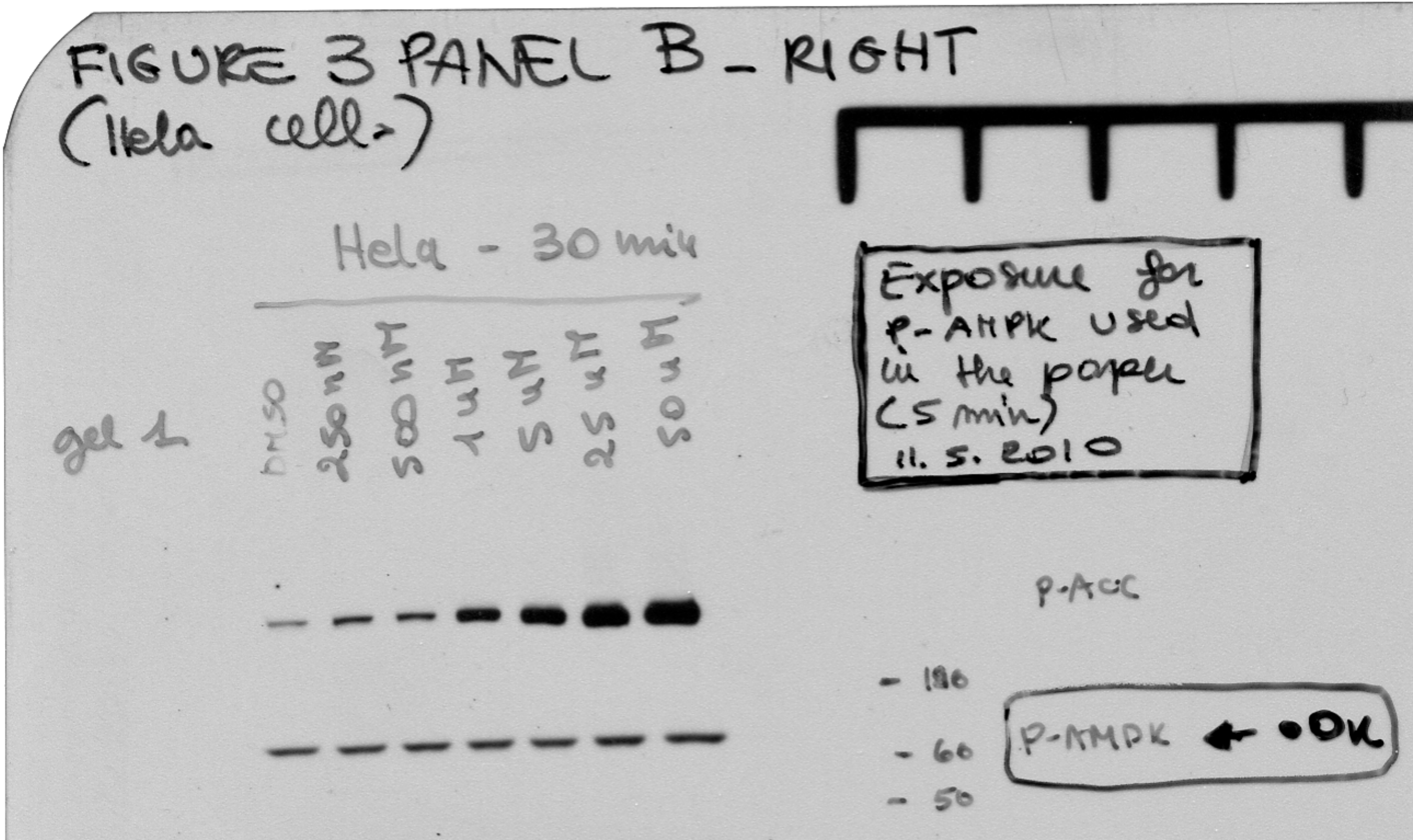
FIGURE 3 PANEL B _ RIGHT
(Hela cells)
Hela - 30 min
gel 1
DMSO
250 μM
500 μM
1 mM
5 mM
25 mM
50 mM
Exposure for p-AMPK used in the paper (5 min)
11. 5. 2010
p-ACC
- 180
- 60
- 50
P-AMPK ← OK

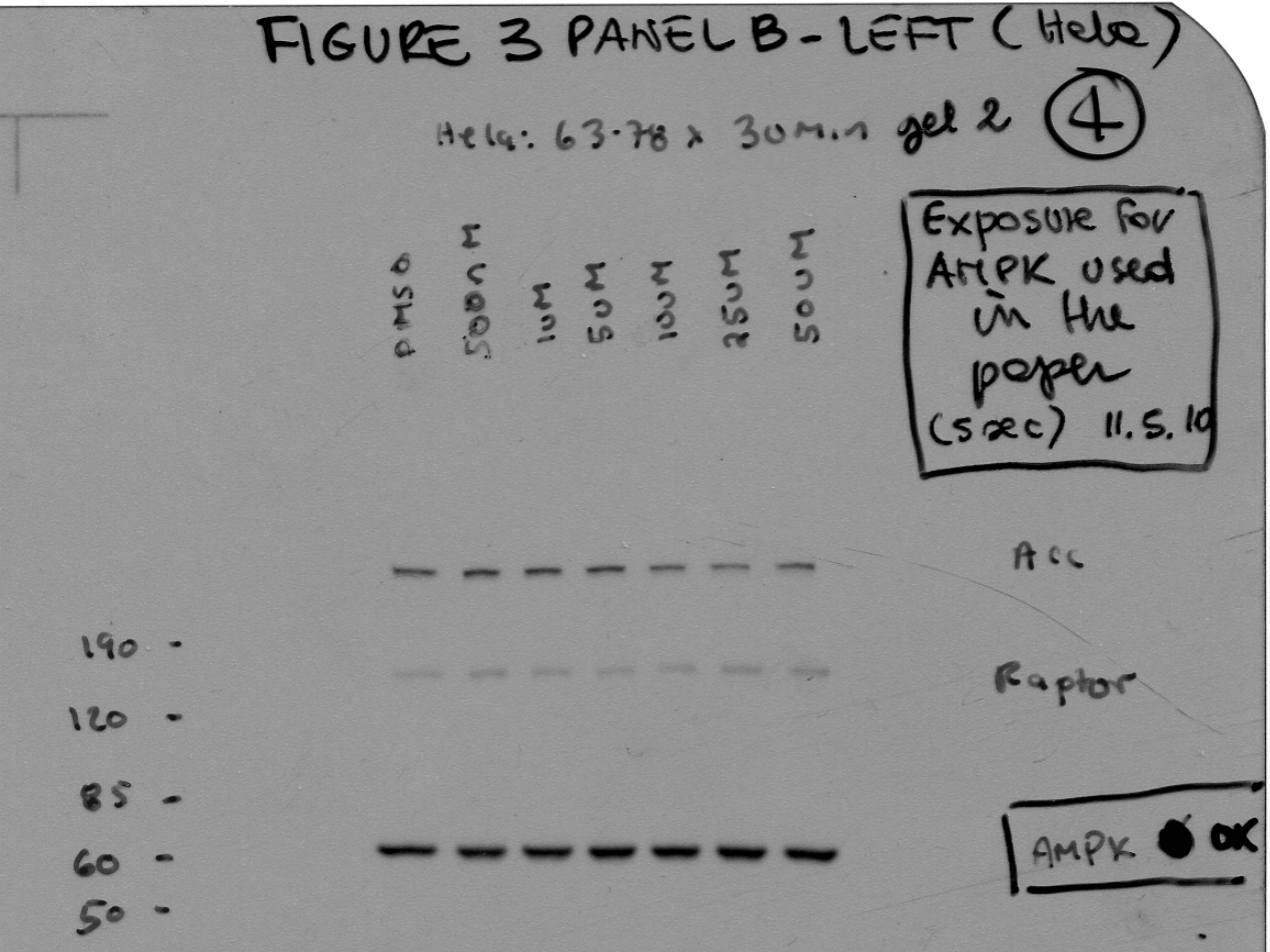

FIGURE 3 PANEL B - LEFT (Hela)
Hela: 63-78 x 30min gel 2
4
Exposure for AMPK used in the paper (5sec) 11.5.19
DMSO
500nM
1uM
5uM
10uM
25uM
50uM
Acc
Raptor
AMPK OK
190
120
85
60
50

Hela - 30 min

HT 63 - 78

FIGURE 3 PANEL B
RIGHT (Hela)

dmso | 250 nM | 500 nM | 1 uM | 5 uM | 25 uM | 50 uM

Exposure for
vinculin used
in the paper
(5 sec)
11.10.10

OK
re-blot

120 -

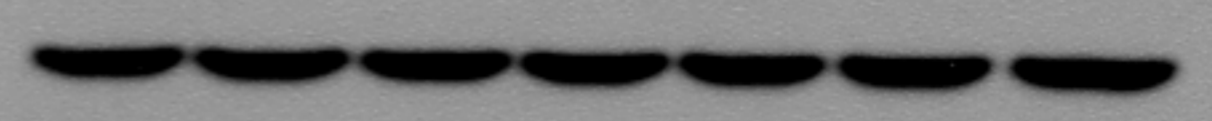

• VINCULIN
(gel 1)

Hela x 30 min (63-78)

- 30 ug

2 Film / 5 sec
ECL
11.10.10

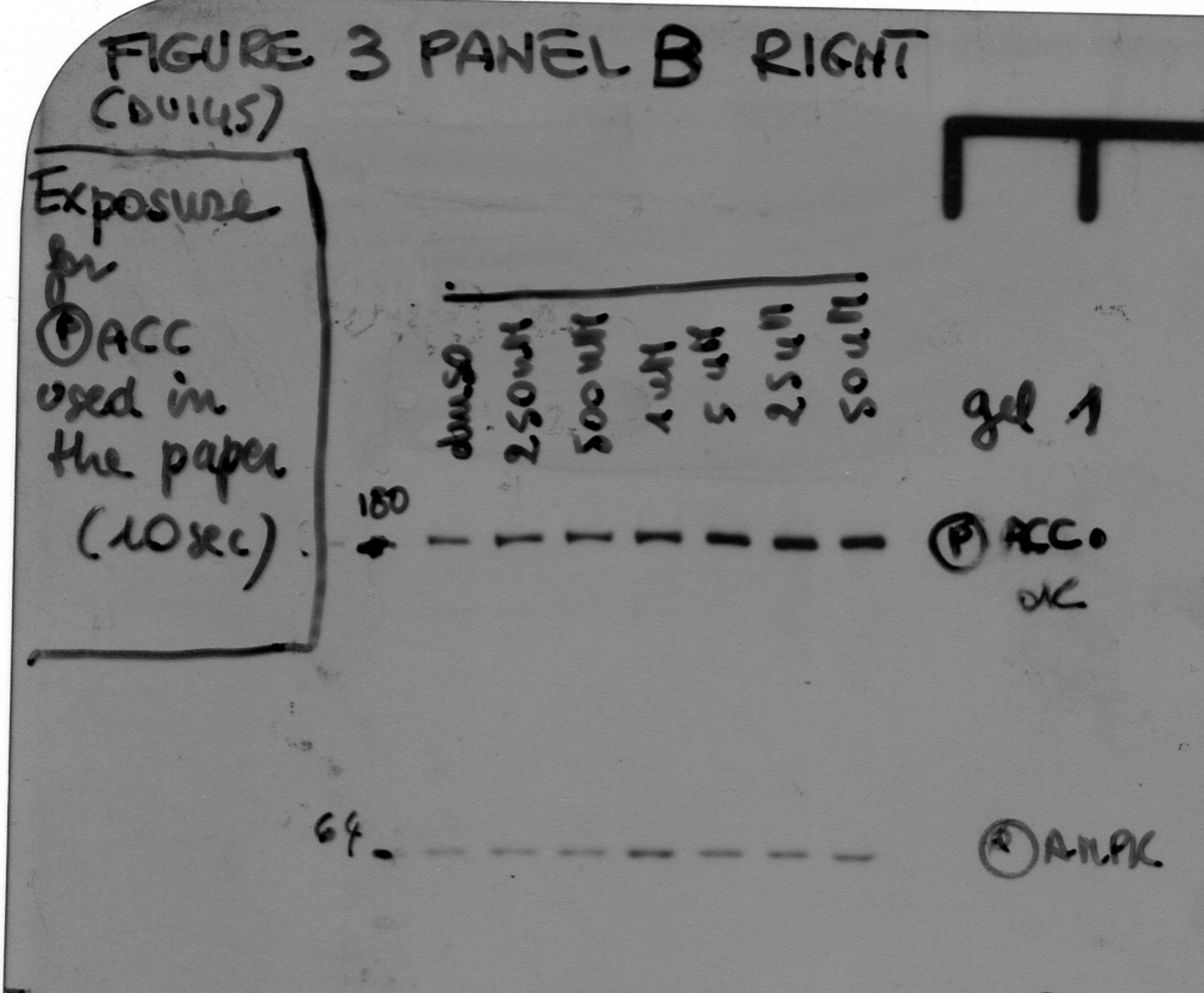
FIGURE 3 PANEL B RIGHT
(DU145)
Exposure for (P)ACC used in the paper (10sec)
dmso
250nM
500nM
1uM
5uM
25uM
50uM
gel 1
180
(P)ACC.
ok
64
(P)AMPK

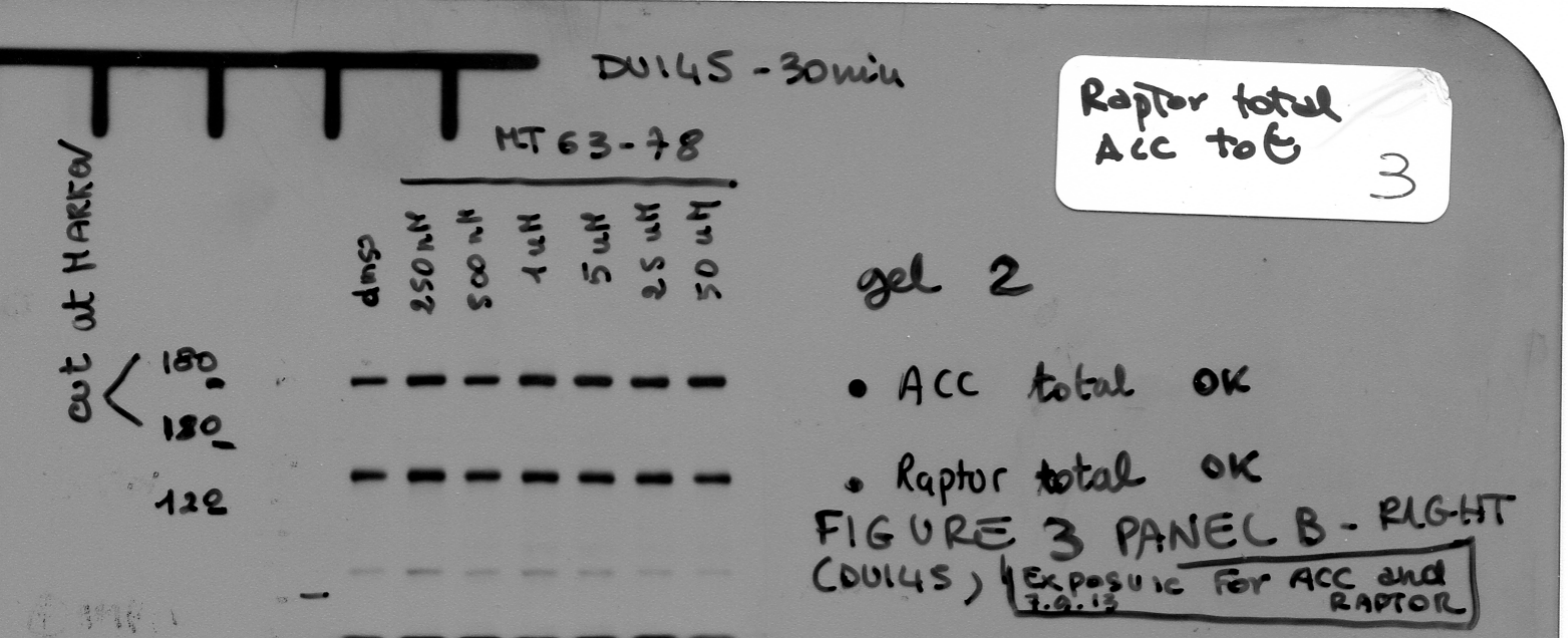
DU145 - 30min
MT63-78
dmso
250 nM
500 nM
1 uM
5 uM
25 uM
50 uM
cut at Marker
180
180
122
Raptor total
ACC tot
3
gel 2
• ACC total OK
• Raptor total OK
FIGURE 3 PANEL B - RIGHT
(DU145)
Exposure for ACC and RAPTOR
7.9.13

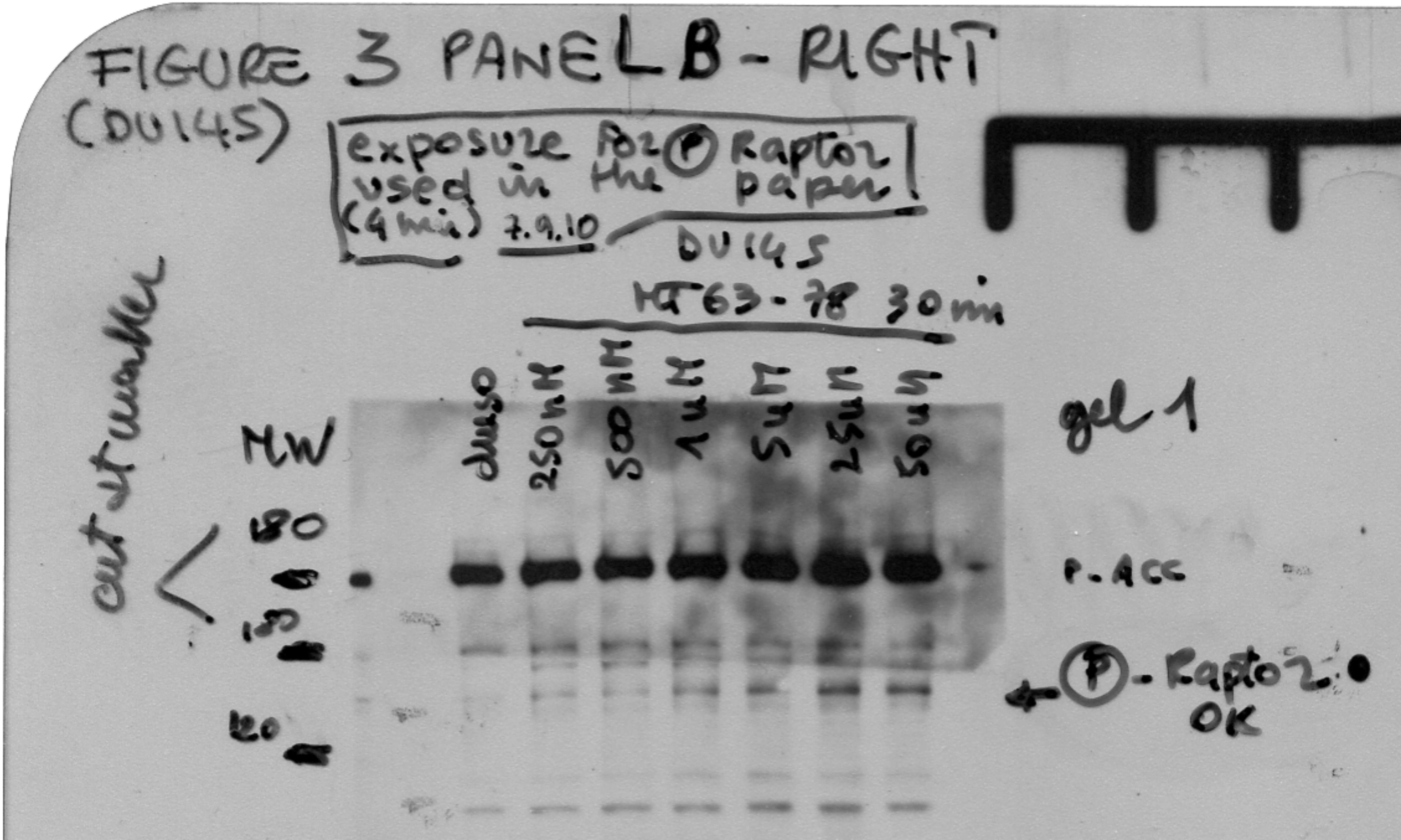
FIGURE 3 PANEL B - RIGHT
(DU145)
exposure for (P) Raptor used in the paper
(4 min) 7.9.10
DU145
HT63-78 30 min
dmso
250 nM
500 nM
1 uM
5 uM
25 uM
50 uM
MW
180
130
120
cut at marker
gel 1
P-ACC
(P)-Raptor
OK

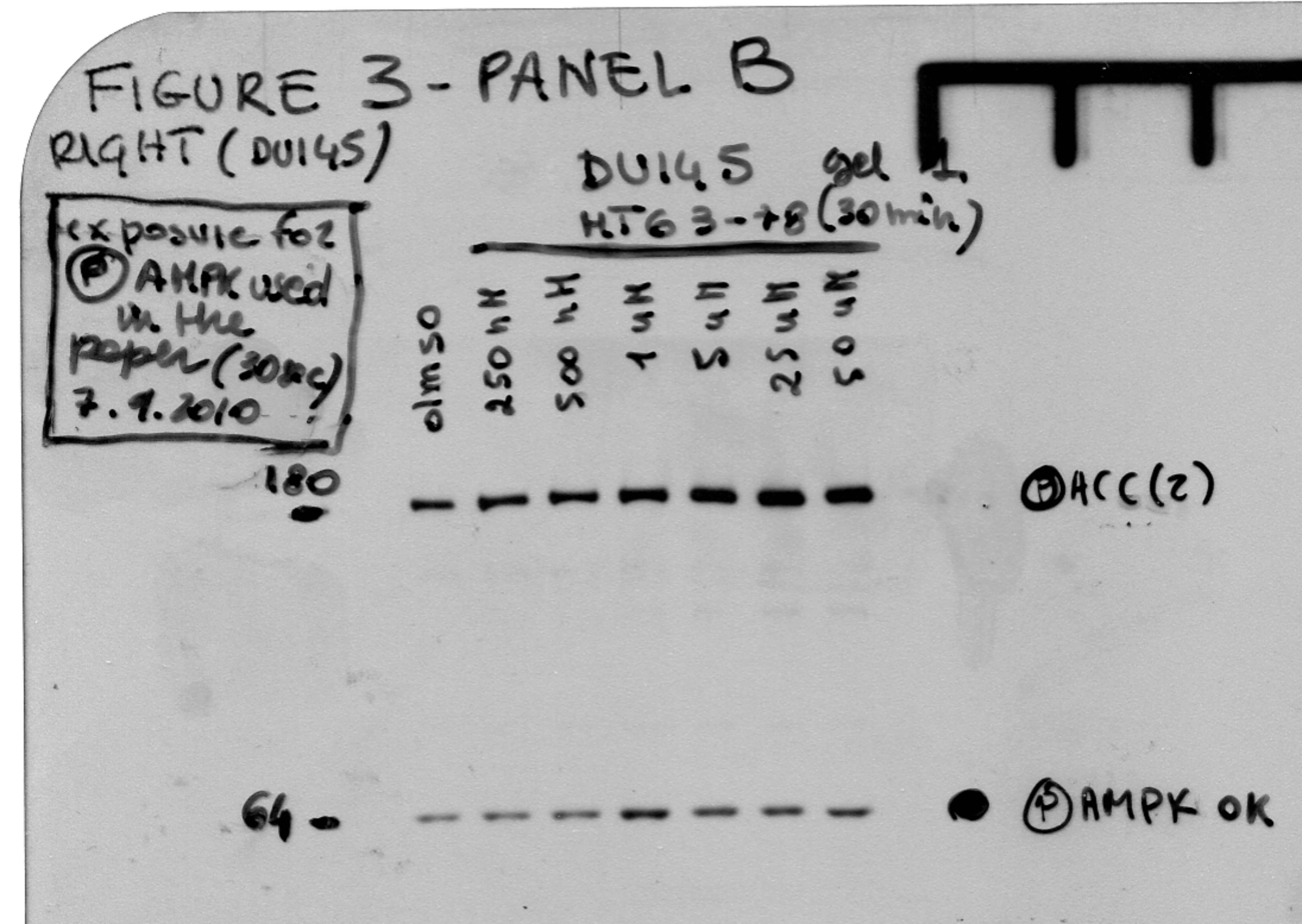
FIGURE 3 - PANEL B
RIGHT (DU145)
exposure for (P) AMPK used in the paper (30sec) 7.9.2010 ?
DU145 gel 1.
HT63-78 (30 min)
dmso
250 nM
500 nM
1 uM
5 uM
25 uM
50 uM
180
(P) ACC (2)
64
(P) AMPK OK

FIGURE 3
PANEL B RIGHT
(DU145)

AMPK tot AMPK tot
(P) 4EBP1
(P) ACC
(1)

DU145 - 30 min

MT 63-78

gel 2

Exposure for AMPK total used in the paper (10 sec)
7.09.10

dmso 250 nM 500 nM 1 µM 5 µM 25 µM 50 µM

120
64
49

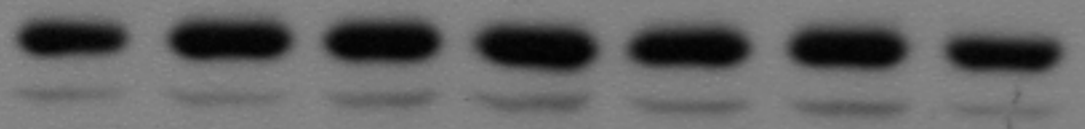

AMPK tot

FIGURE 3 PANEL B
RIGHT - DU145

Exposure for vinculin used in the paper (1 sec)
7.12.10

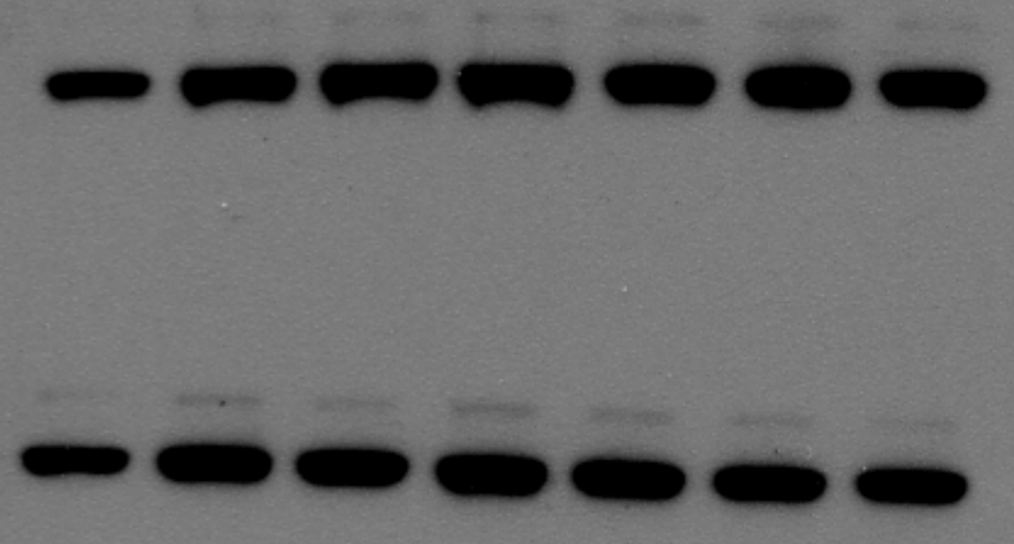

120 - re-blot on VINCULIN (P-Rap Mem) gel 1

- 120 re-blot on VINCULIN (Rap Mem) • OK gel 2

1 2 3 4 5 6 7

DU145 - 30 min

1 = dmso
2 = 250 nM
3 = 500 nM
4 = 1 uM
5 = 5 uM
6 = 25 uM
7 = 50 uM

(2–7) MT63-78

quick ECL 7/12/10

FIGURE 3 PANEL D

①

Exposure for P- ACC used in the paper (1x)

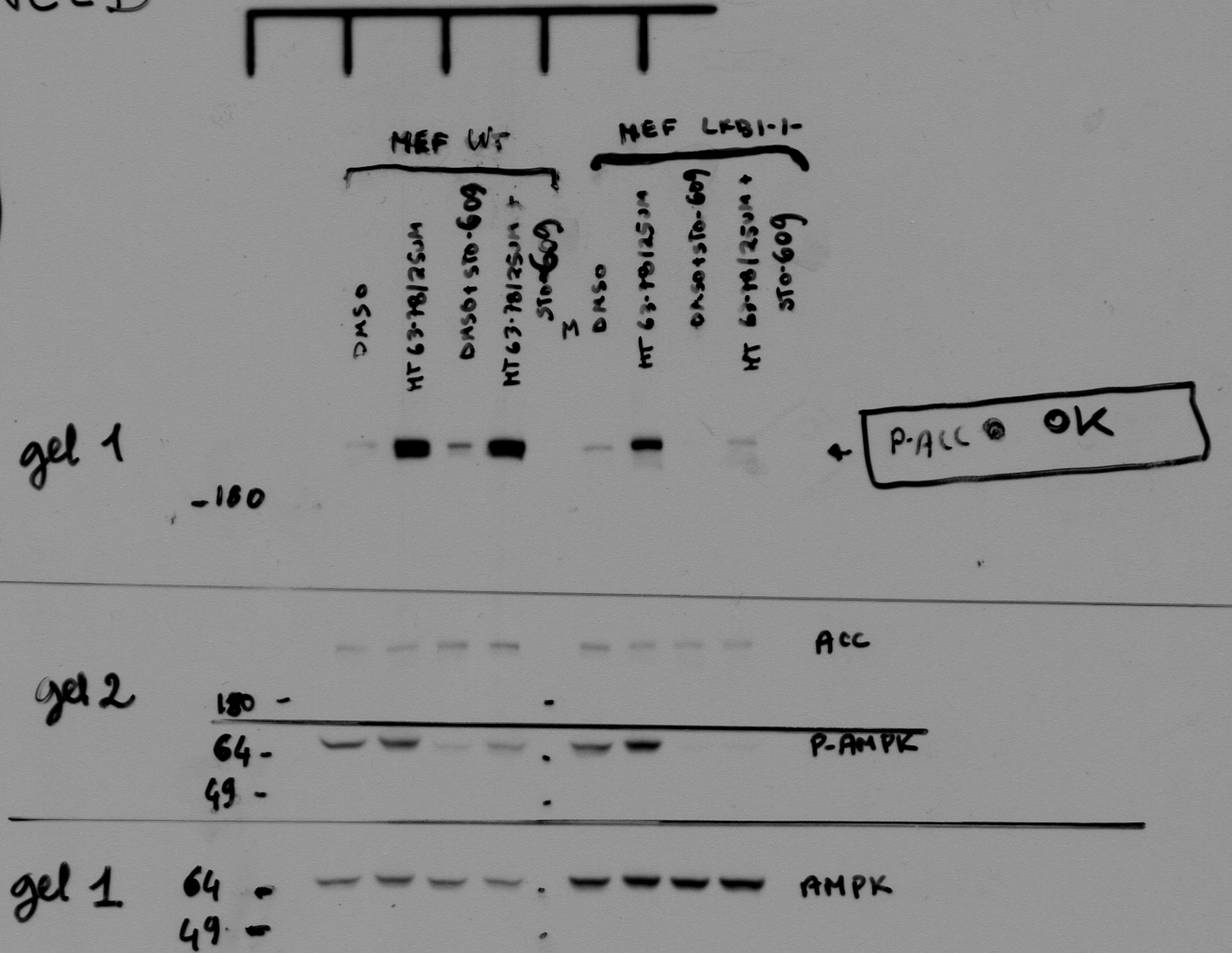


Samples were prepared in duplicate and loaded on 2 gels (gel 1 and 2)

- 25ug
- 8/17/2010 samples

quick ECL
8.24.10

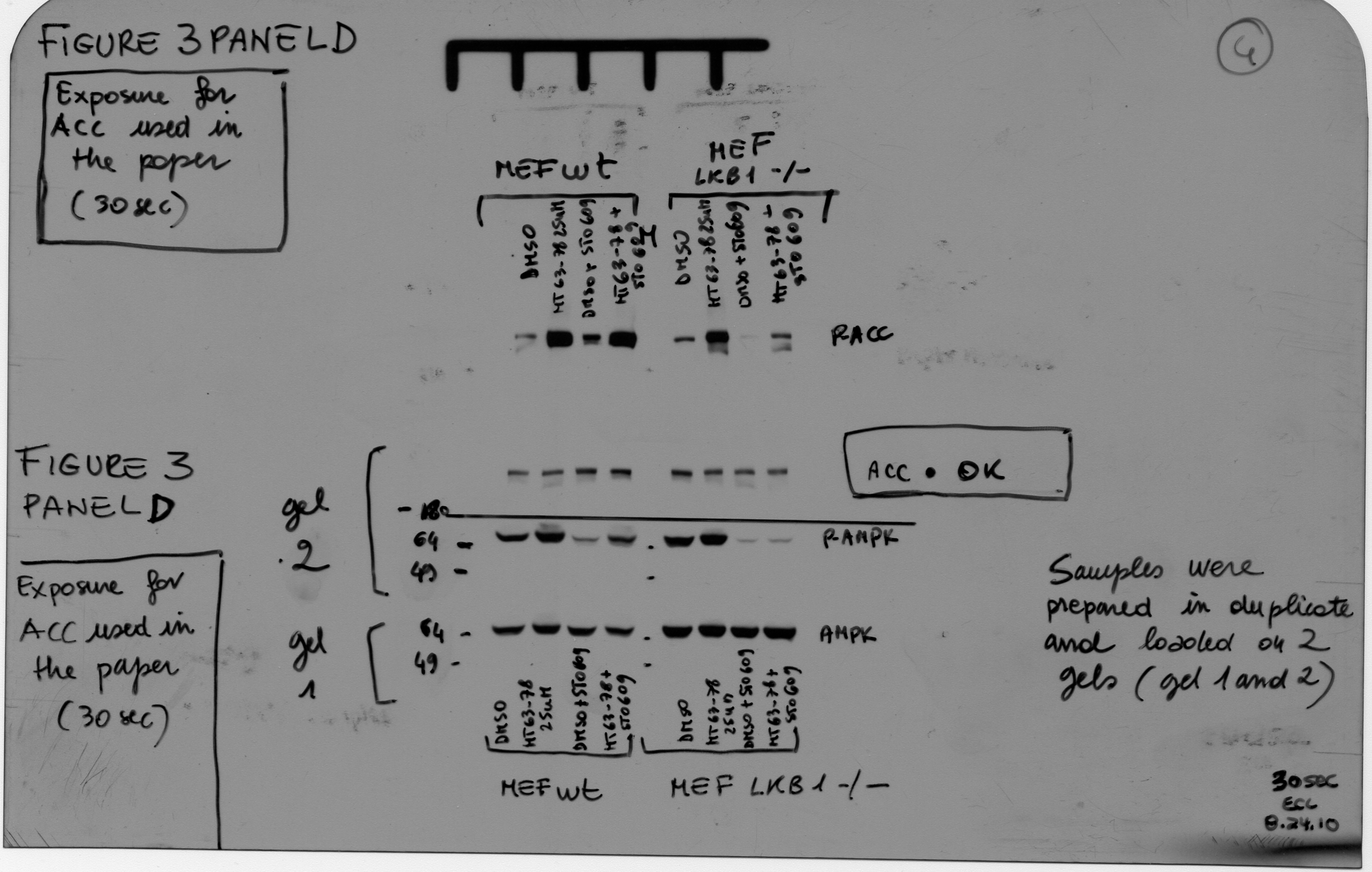
FIGURE 3 PANEL D
Exposure for ACC used in the paper (30 sec)
4
MEF wt
MEF LKB1 -/-
DMSO
MT63-78 25uM
DMSO + STO609
MT63-78 + STO609
PACC
FIGURE 3 PANEL D
ACC • OK
gel 2
180
64
49
P-AMPK
Samples were prepared in duplicate and loaded on 2 gels (gel 1 and 2)
Exposure for ACC used in the paper (30 sec)
gel 1
64
49
AMPK
MEF wt
MEF LKB1 -/-
30 sec
ECL
8.24.10

FIGURE 3 PANEL D

③

Exposure for: (P)-Raptor and Raptor used in the paper (5 min)

MEF WT | MEF LKB1-/-

DMSO, 63-78/25nM, DMSO+STO-609, 63-78/25nM+STO-609, M, DMSO, 63-78/25nM, DMSO+STO-609, 63-78/25nM+STO-609

gel 1 - 180 - 115

gel 2 - 180 - 115

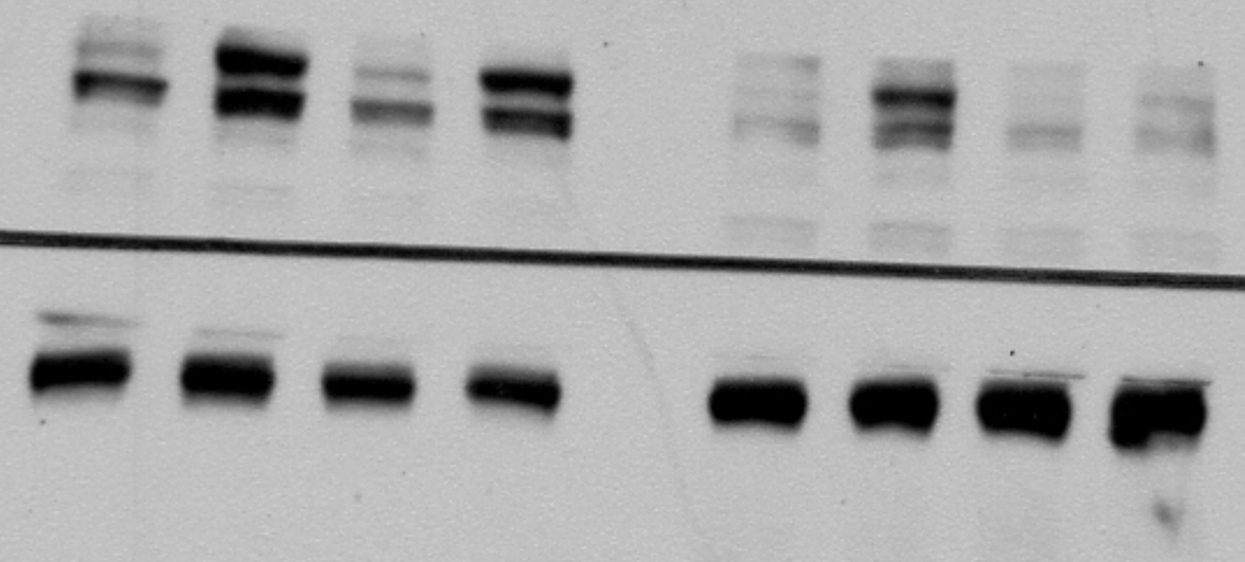

* P-Raptor● OK

* Raptor● OK

- 25ug
- 8/17/2010 samples

Samples were prepared in duplicate and loaded on 2 gels (gel 1 and 2)

5 min
ECL
8.24.10


-25ug
-8/17/2010 samples

Samples were prepared in duplicate and loaded on 2 gels (gel 1 and 2)

5min
ECL
8.24.10

FIGURE 3, PANEL D



Exposure for
- P-AMPK
- AMPK used in the paper (10 sec)

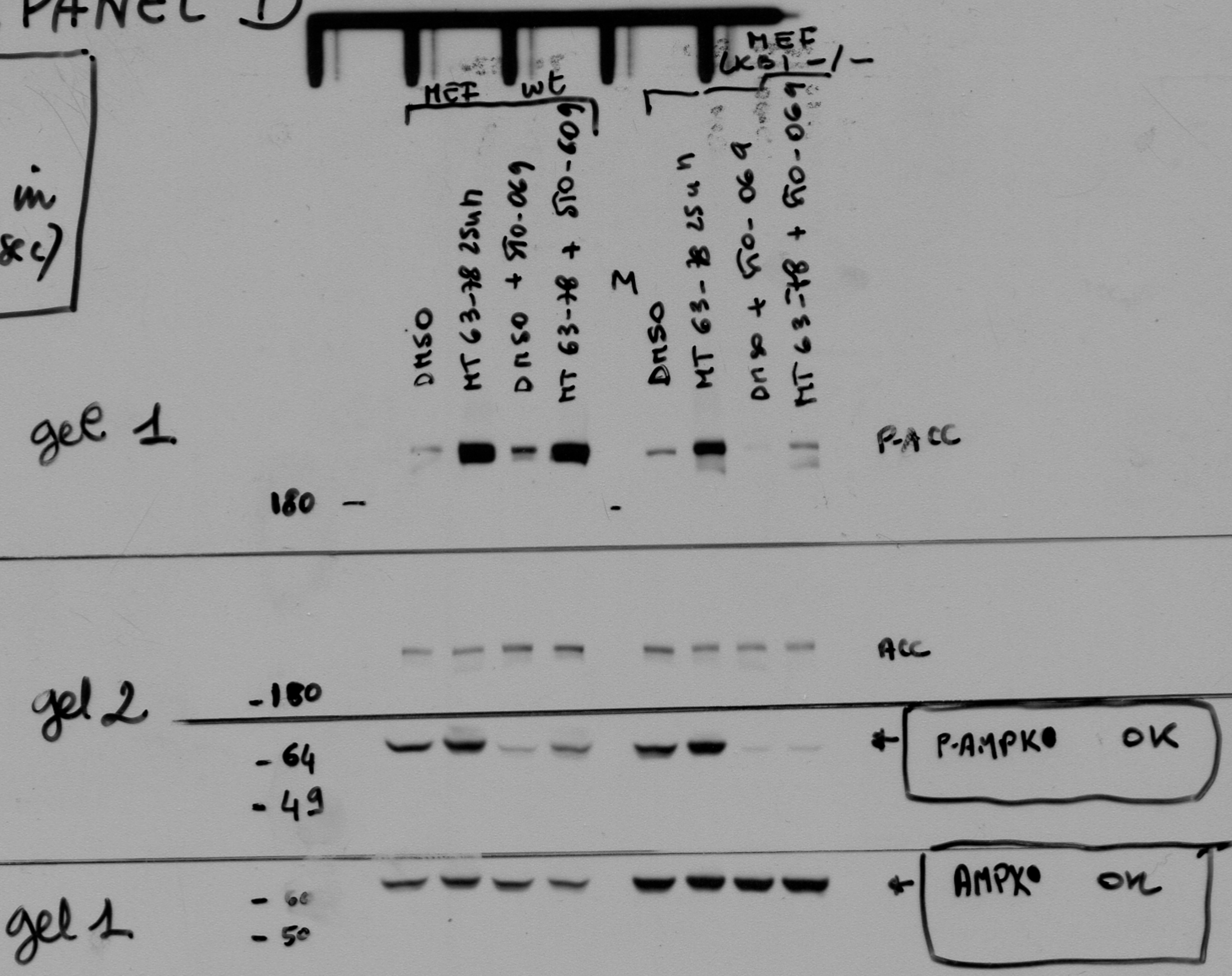


Samples were prepared in duplicate and loaded on 2 gels (gel 1 and 2)

10 sec
ECL
8.24.10

# FIGURE 3 PANEL D

Exposure for vinculin used in the paper (5sec)

Re-blot

(5)

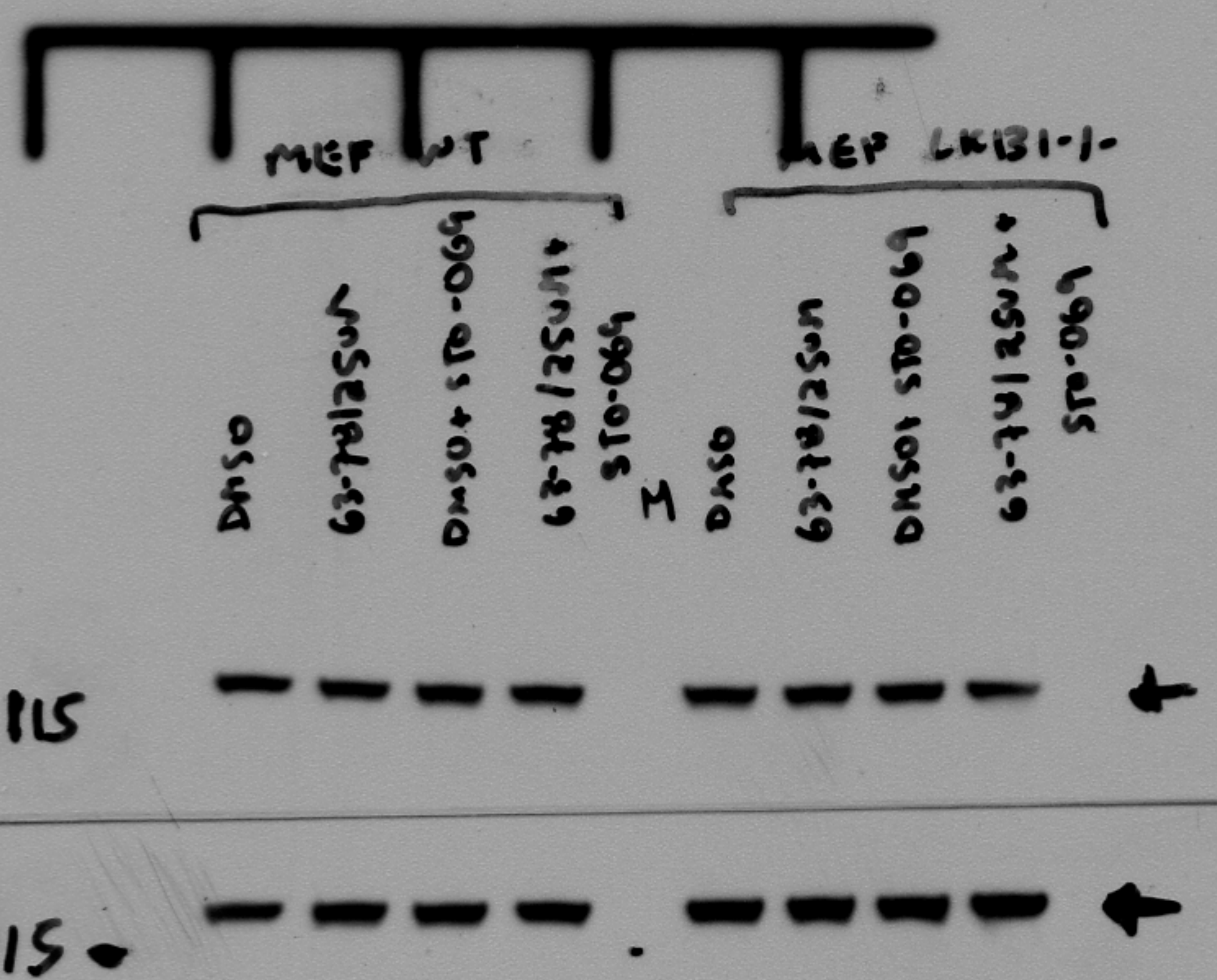


gel 1 -115

VINCULIN re-blot on P-Raptor Membrane ● OK

gel 2 115

"VINCULIN re-blot on Raptor membrane

- 25ug
- 8/17/2010 samples

Samples were prepared in duplicate and loaded on 2 gels (gel 1 and 2)

-25ug

- 8/17/2010 samples

2 films/5sec
ECL
8.25.10
