# Supplementary material for: A novel direct activator of AMPK inhibits prostate cancer growth by blocking lipogenesis
Source: EMBO Mol Med. 2014 Feb 4;6(4):519–38. doi: 10.1002/emmm.201302734 (PMC3992078; doi:10.1002/emmm.201302734)
Supplement: Supplementary file 5 [file emmm0006-0519-sd5.pdf]

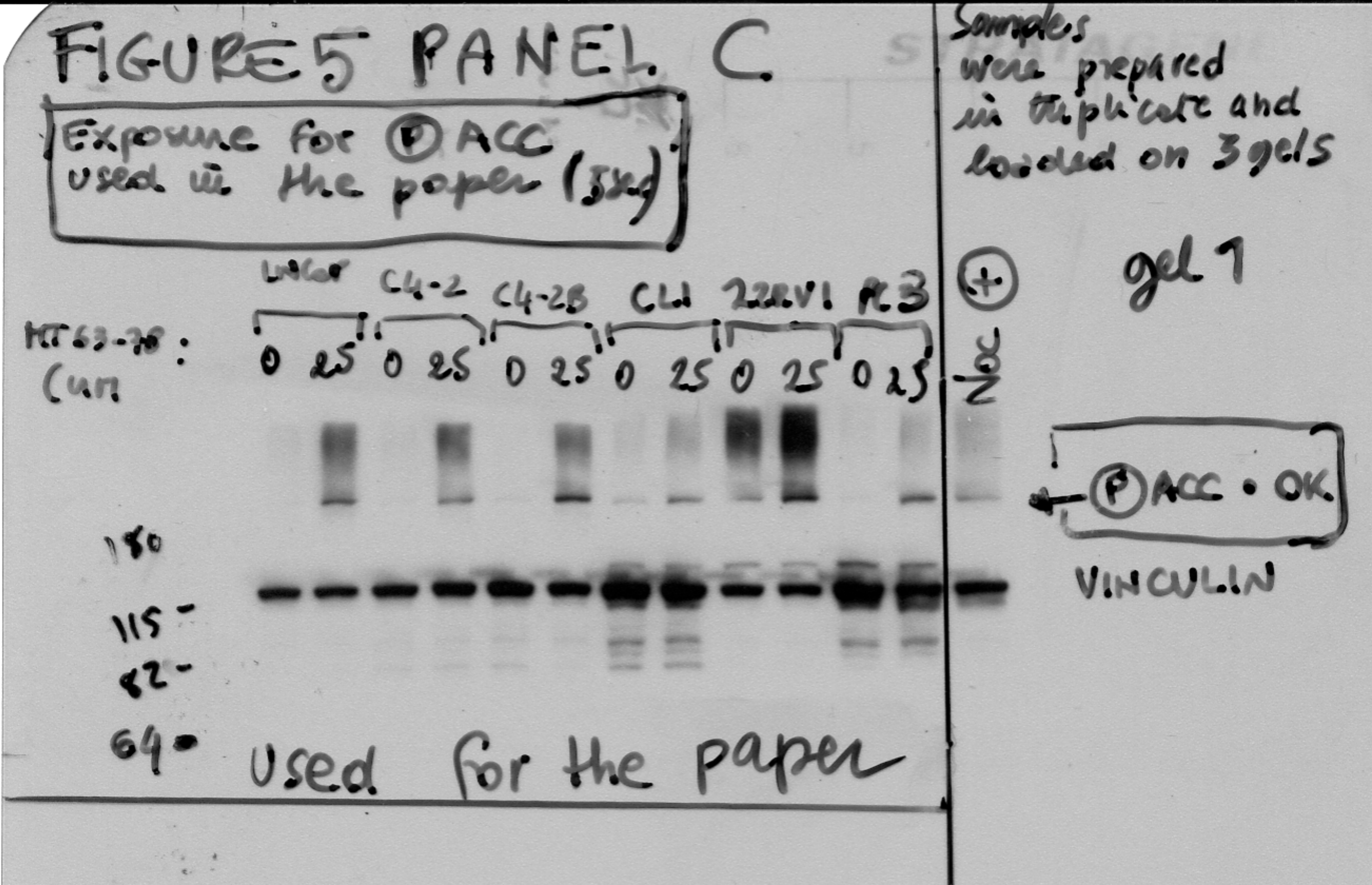
FIGURE 5 PANEL C
Exposure for (P) ACC used in the paper (5sec)
Samples were prepared in triplicate and loaded on 3 gels
gel 1
LNCaP
C4-2
C4-2B
CL1
22RV1
PC3
(+)
Noc
HTI 63-78 :
(uM)
0 25 0 25 0 25 0 25 0 25 0 25
(P) ACC • OK
VINCULIN
180
115
82
64
Used for the paper

Samples were prepared in triplicate and loaded on 3 gels

FIGURE 5C

CELLS

gel 2

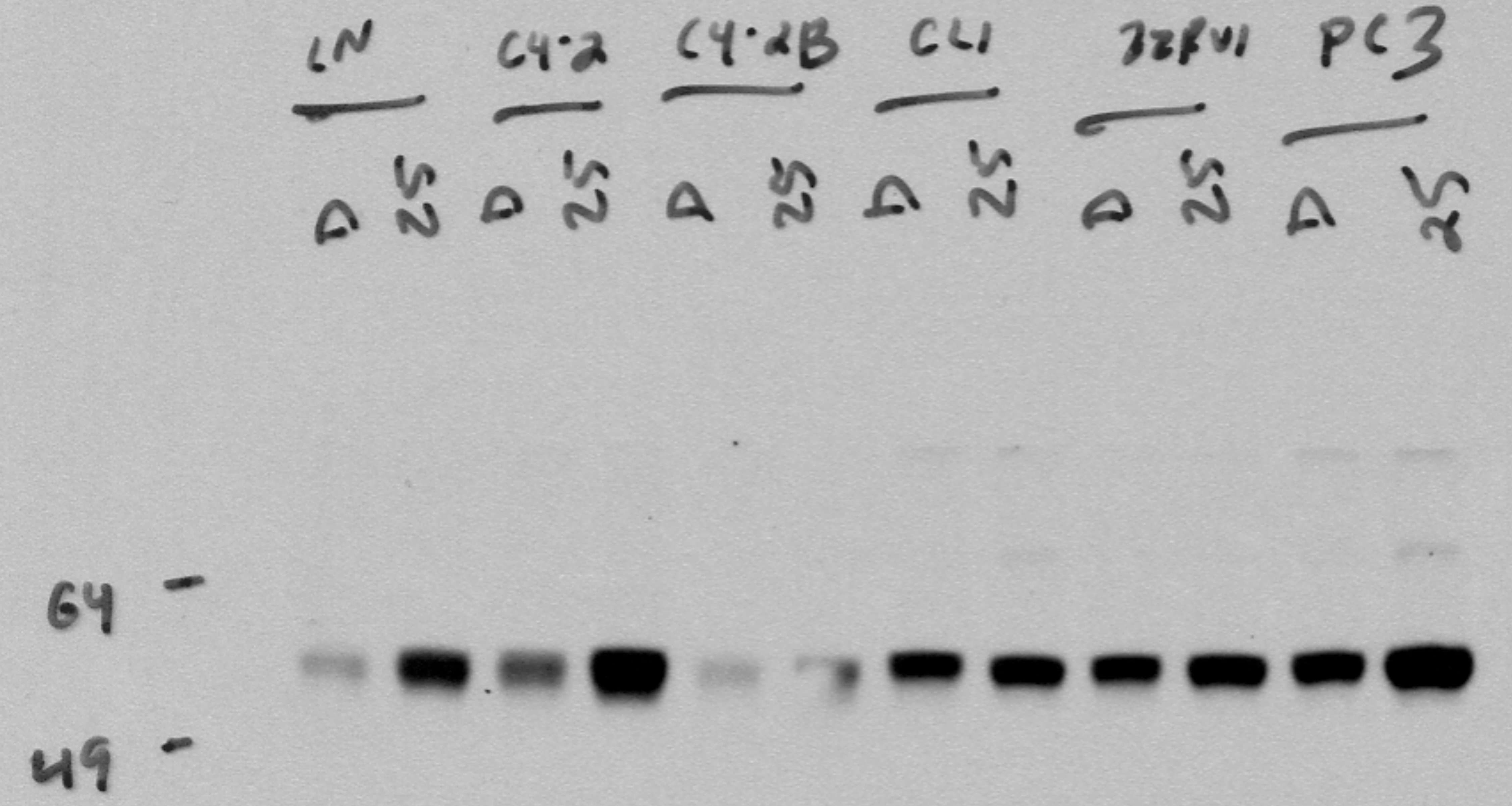

· cyclin B1 (re-blot on PLK1 membrane) 30 sec

OK

9.26.13

Exposure used for
· cyclin B1 (re-blot)
used in the paper (30 sec)

2

FIGURE 5 C.

CELLS

Exposune for Aurora A used in the paper (short exposune)

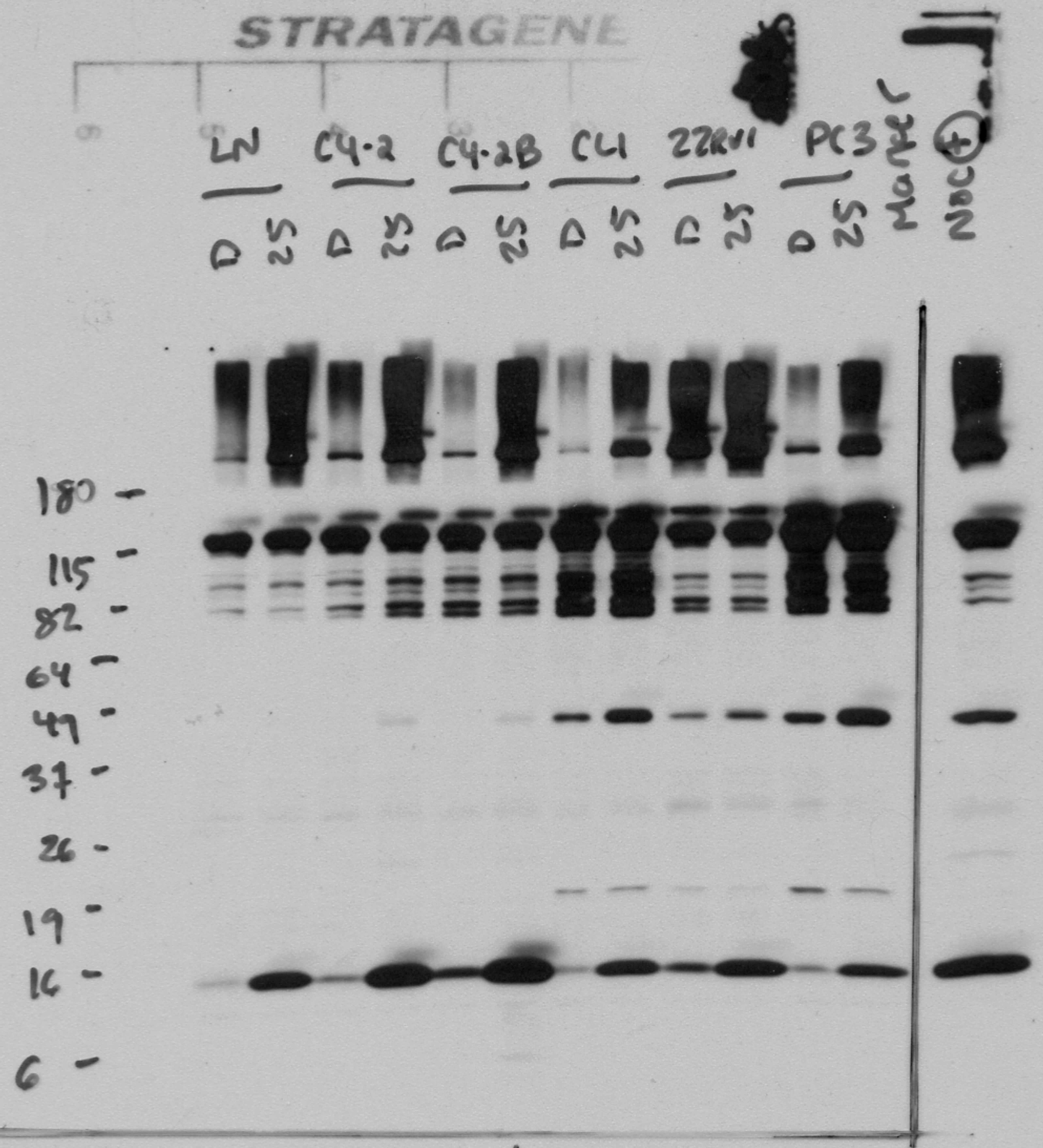

Used in the paper

gel 3

P-ACC

vinc

• Aurora A OK (short exp)

P-Histone-H2AX

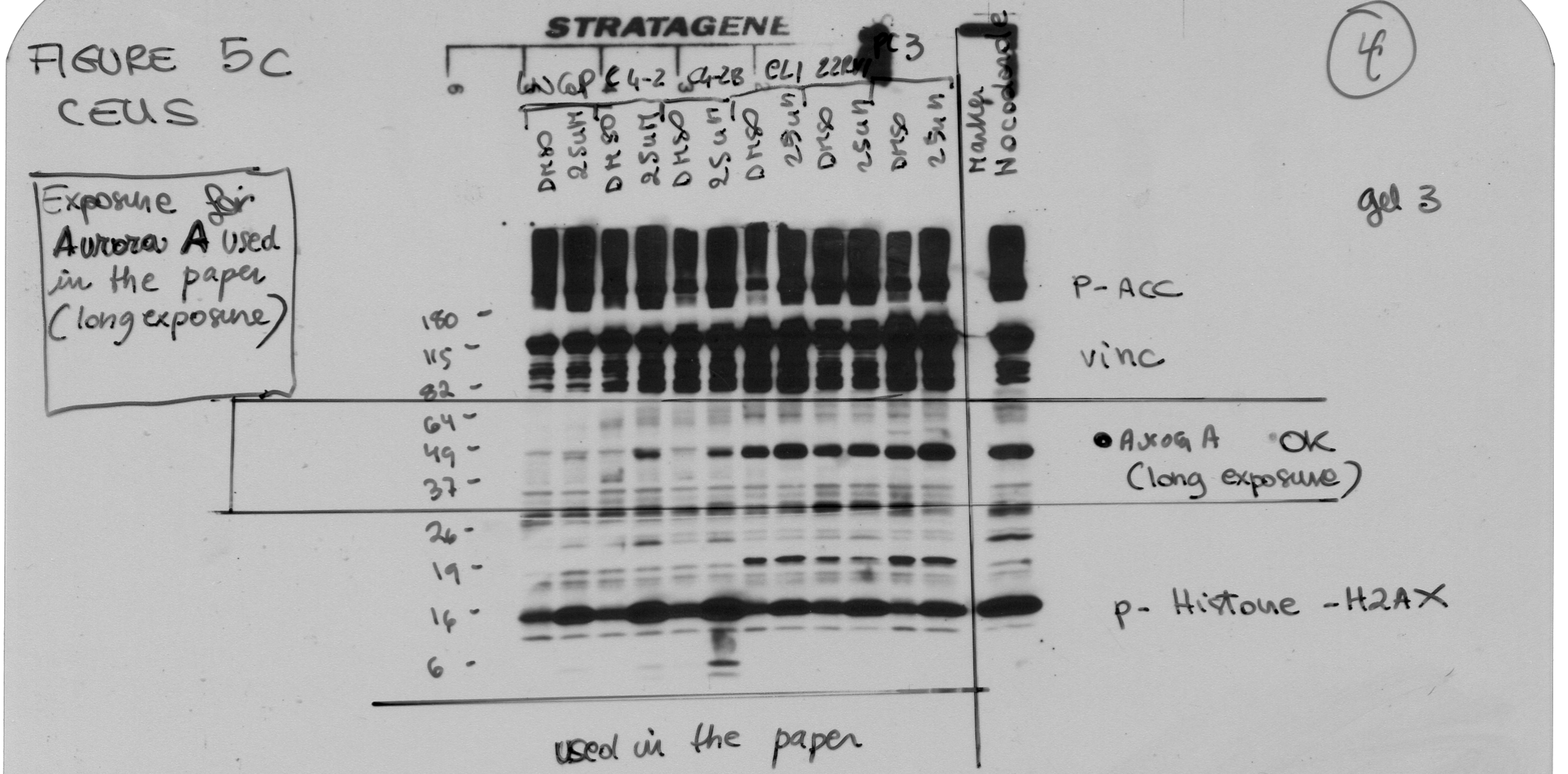
FIGURE 5C
CELLS
4
STRATAGENE
LNCaP
C4-2
C4-2B
CL1
22RV1
PC3
DMSO
2.5uM
Mankey
Nocodazole
Exposure for Aurora A used in the paper (long exposure)
gel 3
180
115
82
64
49
37
26
19
14
6
P-ACC
vinc
Aurora A OK (long exposure)
p-Histone-H2AX
used in the paper

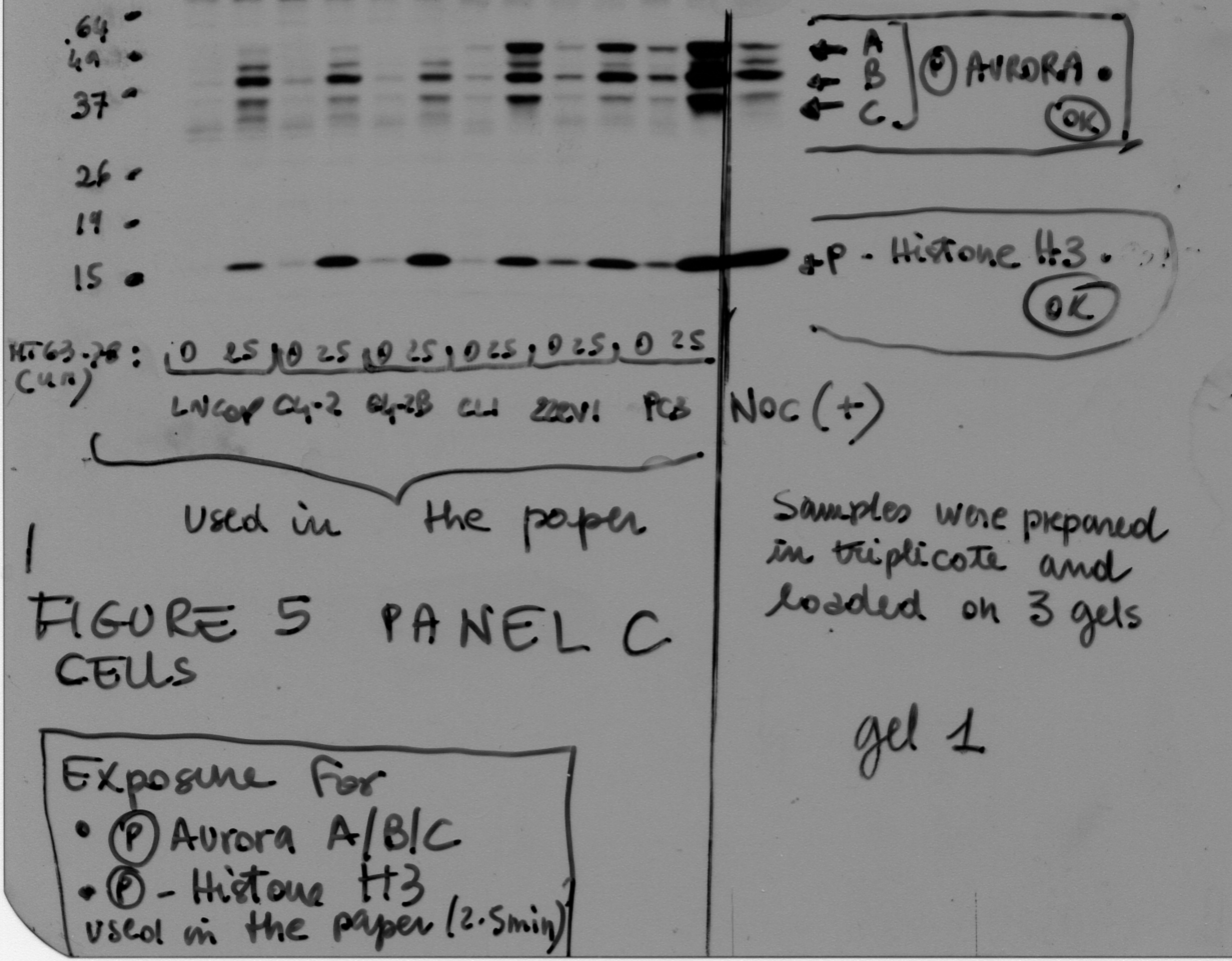
64
49
37
26
19
15
A
B
C
(P) AURORA
OK
P - Histone H3
OK
0 2.5 0 2.5 0 2.5 0 2.5 0 2.5 0 2.5
LNCaP
C4-2
C4-2B
CL1
22Rv1
PC3
Noc (+)
Used in the paper
FIGURE 5 PANEL C
CELLS
Exposure for
• (P) Aurora A/B/C
• (P) - Histone H3
used in the paper (2.5min)
Samples were prepared in triplicate and loaded on 3 gels
gel 1

FIGURE 5C

CELLS

Exposure for
- PLK1
- AURORA B

used in the paper
(5 min)

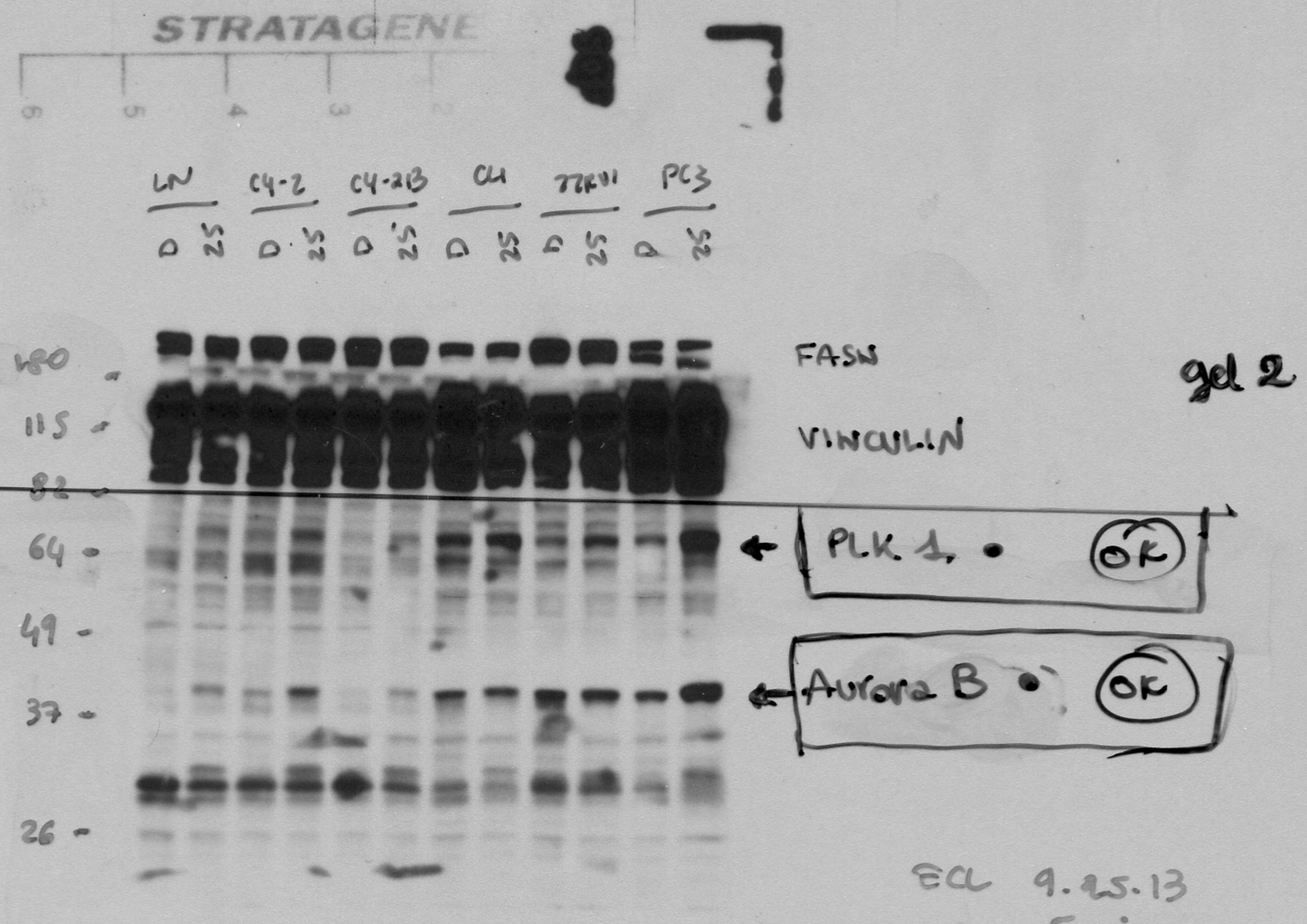

Samples were prepared in triplicate and loaded on 3 gels.

ECL 9.25.13
5 min

FIGURE 5C - CELLS

Exposure for VINCULIN used in the paper (5 sec)

Samples were prepared in Triplicate and loaded on 3 gels.

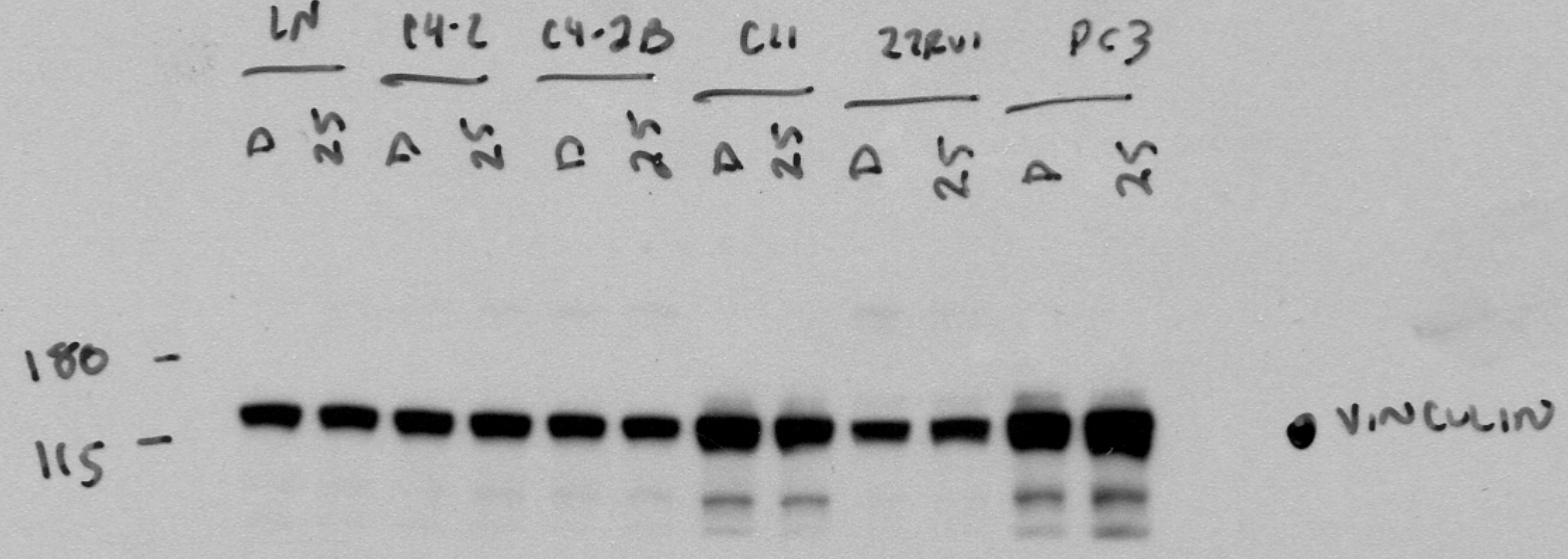

gel 2

OK

2 films 15 sec

9.25.13

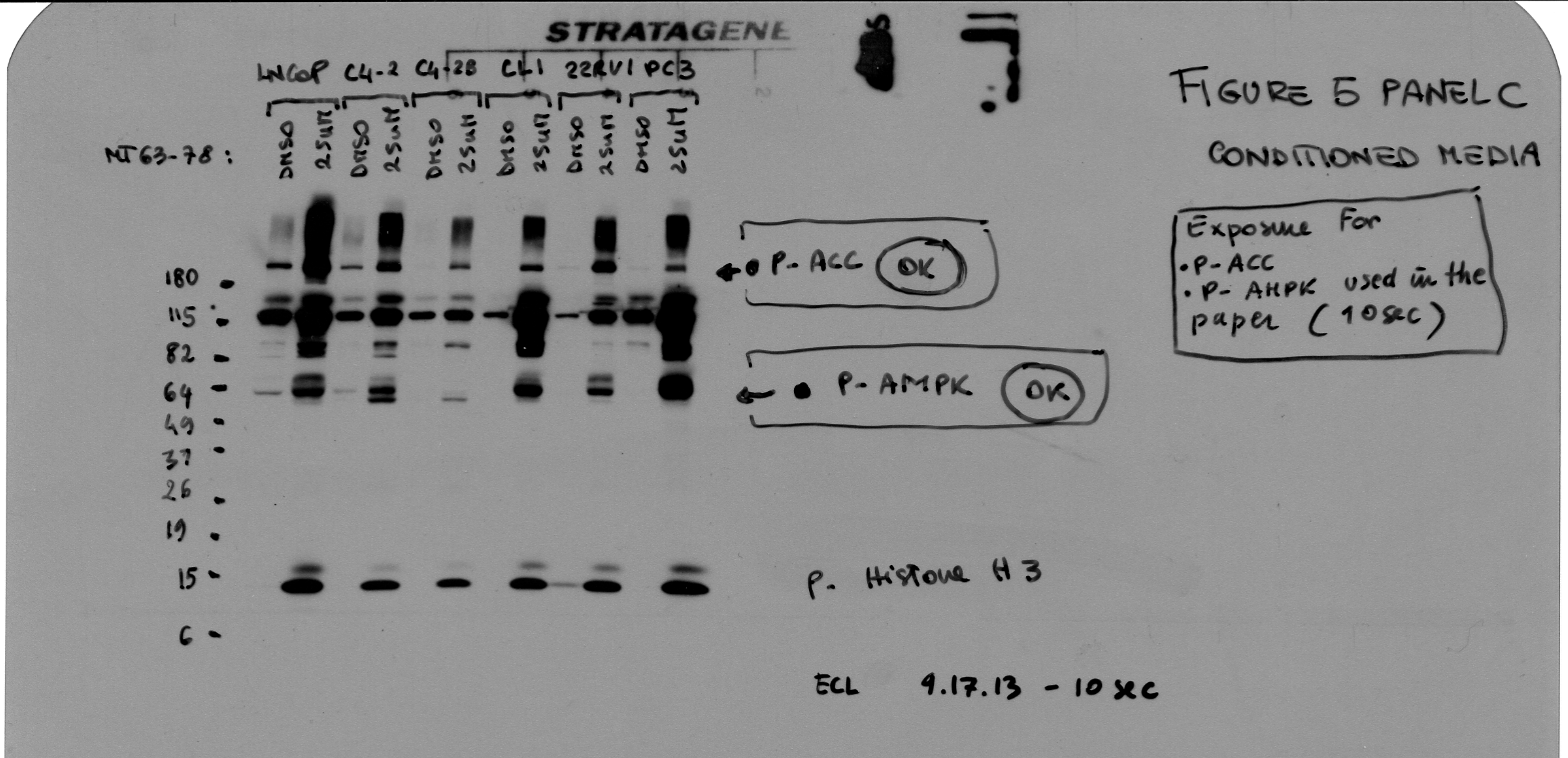
STRATAGENE
FIGURE 5 PANEL C
CONDITIONED MEDIA
LNCaP C4-2 C4-2B CL1 22RV1 PC3
MT63-78 :
DMSO 25uM DMSO 25uM DMSO 25uM DMSO 25uM DMSO 25uM DMSO 25uM
180
115
82
64
49
37
26
19
15
6
• P- ACC OK
• P- AMPK OK
P. Histone H3
Exposure for
• P- ACC
• P- AMPK used in the paper (10 sec)
ECL 9.17.13 - 10 sec

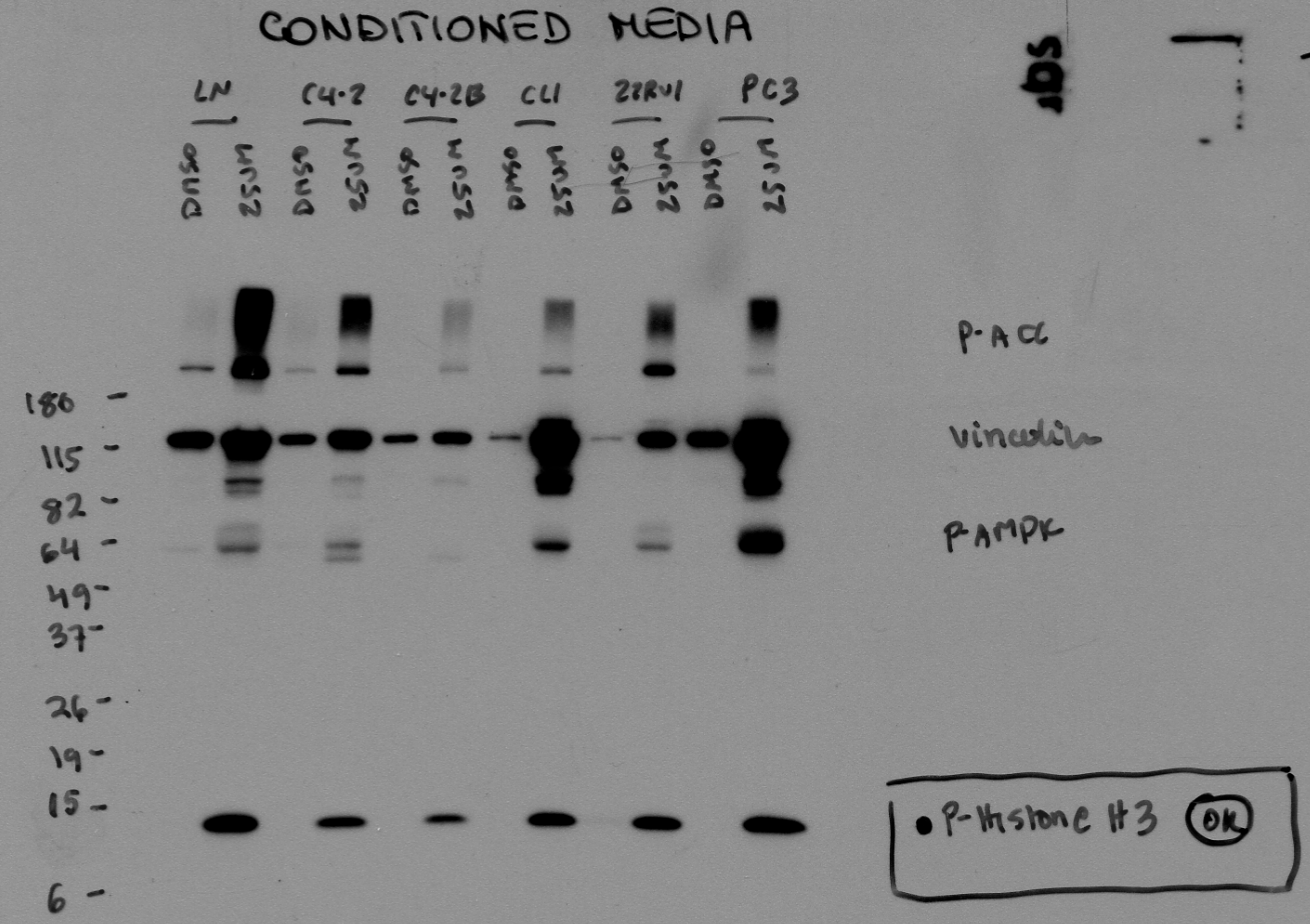

FIGURE 5 PANEL C
CONDITIONED MEDIA

Exposure for p-Histone H3 used in the paper (1 sec)

quick (2nd try)

9.17.13

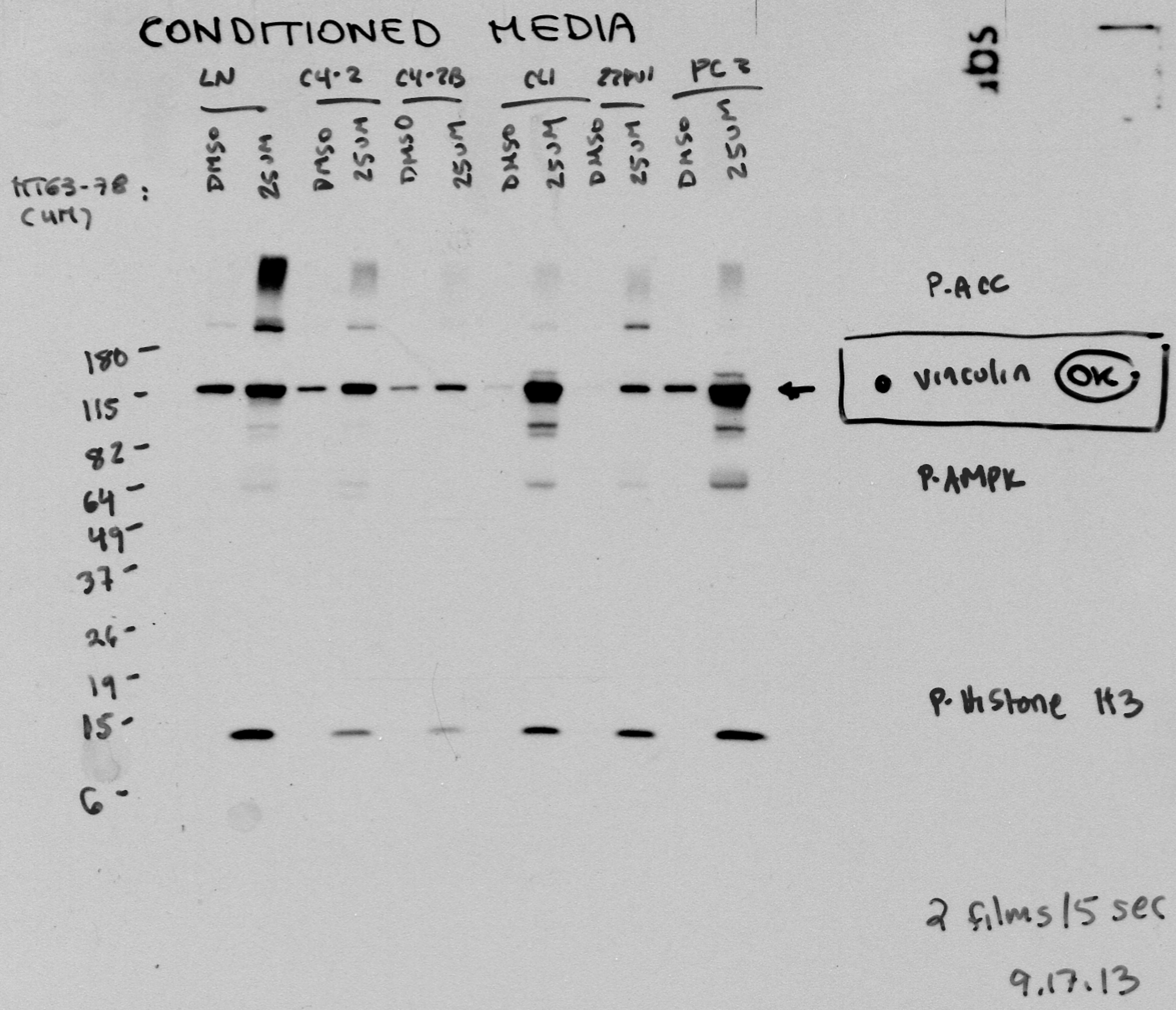

FIGURE 5 PANEL C
CONDITIONED MEDIA

Exposure for VINCULIN used in the paper (5 sec)

FIGURE 5 PANEL C

CONDITIONED MEDIA

Exposure for cyclin B1 used in the paper (5 min)

CONDITIONED MEDIA

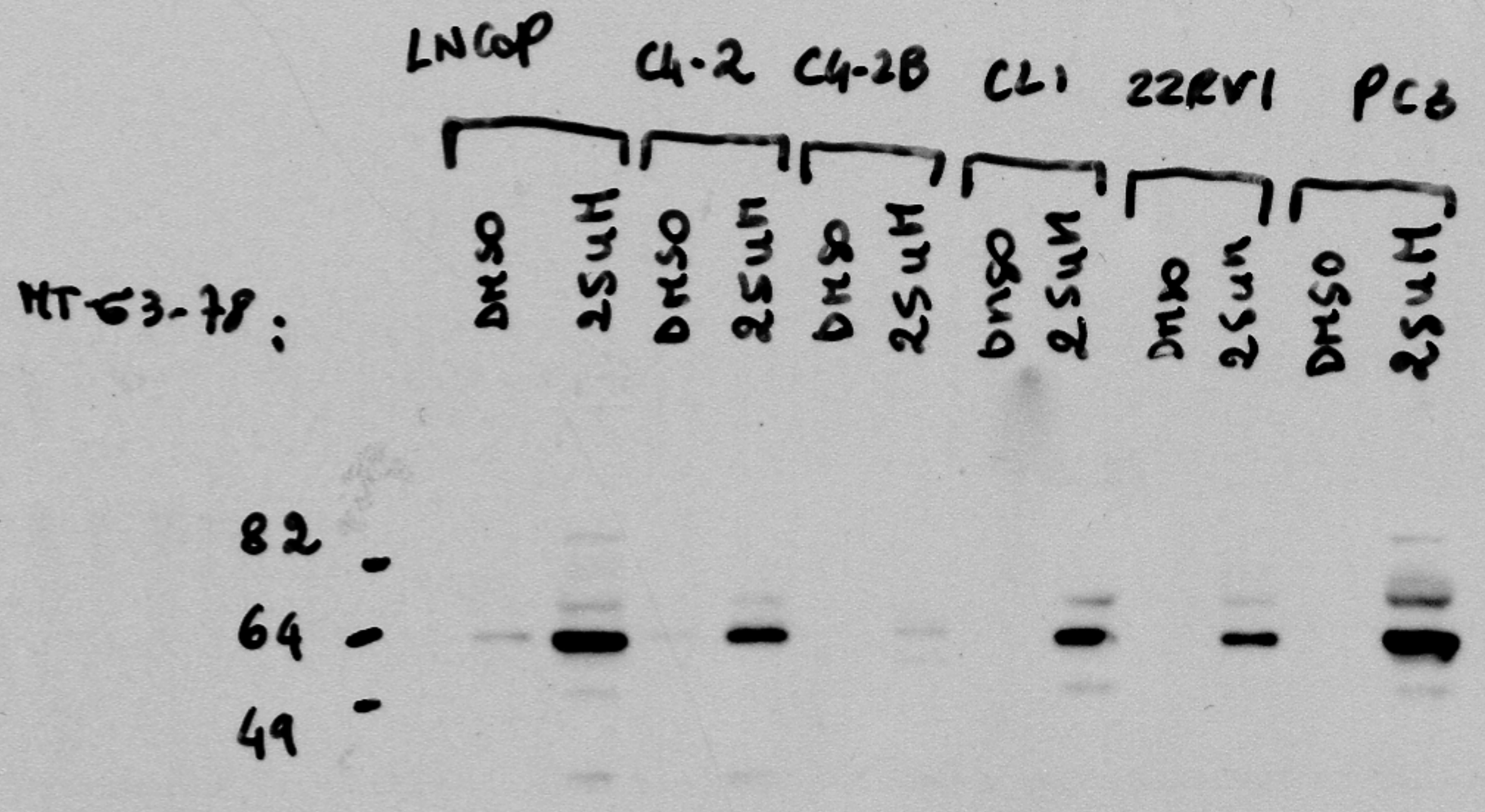

← cyclin B1 (re-blot on P-AMPK membrane)

• OK

ECL+ 5 min

09.18.13

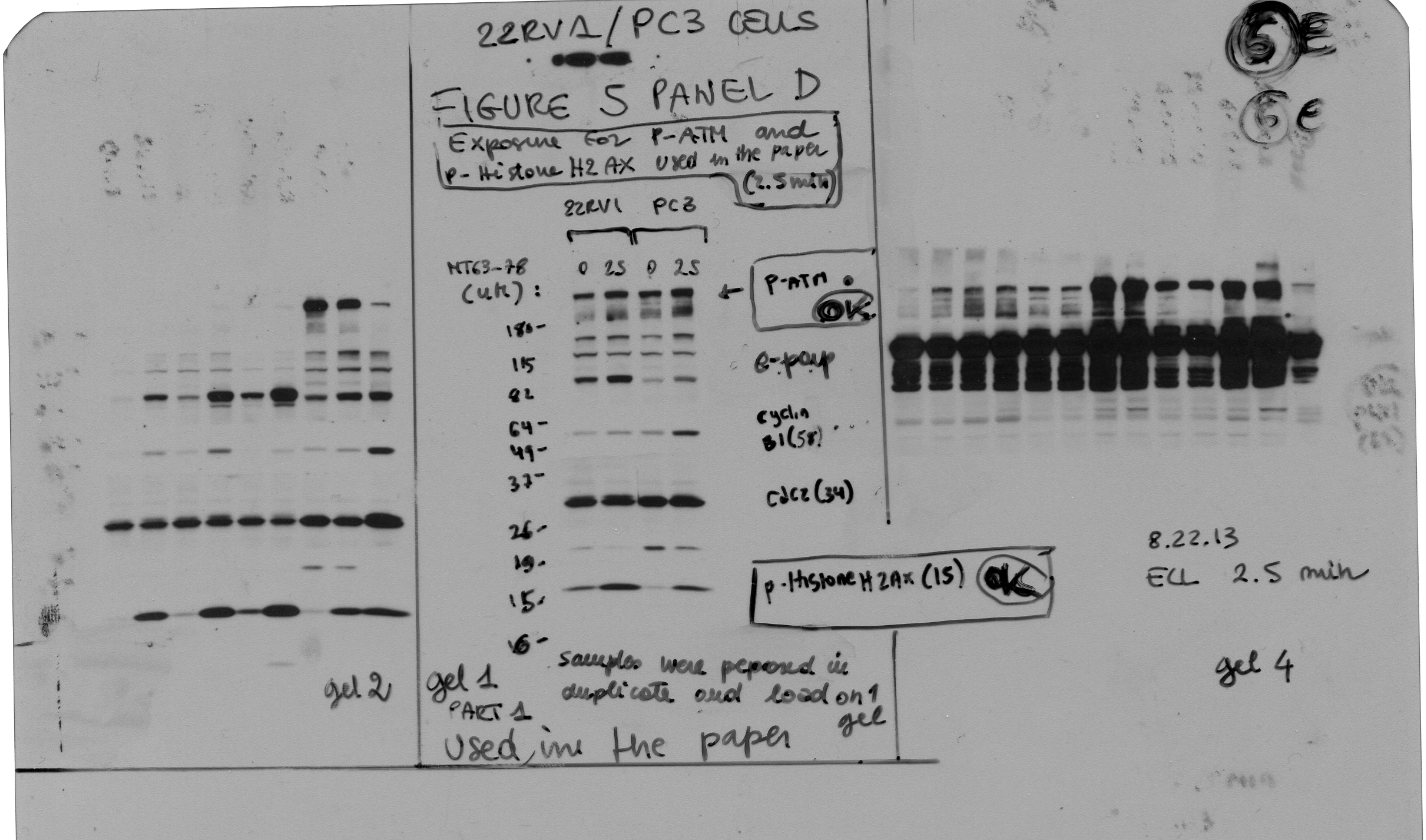
22RV1/PC3 CELLS
FIGURE 5 PANEL D
Exposure for P-ATM and p-Histone H2AX used in the paper (2.5 min)
22RV1 PC3
MT63-78 (uM):
0 25 0 25
180-
115
82
64-
49-
37-
26-
19-
15-
6-
P-ATM
OK
cyclin B1(58)
cdc2(34)
p-Histone H2AX (15)
OK
samples were prepared in duplicate and load on 1 gel
gel 2
gel 1
PART 1
Used in the paper
8.22.13
ECL 2.5 min
gel 4
5E
6E

LNCaP - CL1 cells

FIGURE 5 PANEL D

Exposure for VINCULIN used on the paper

LNCaP C4-2 C4-2B CL1 NOC (+)

MT 63-78 (uM): 0 2.5 0 2.5 0 2.5 0 2.5

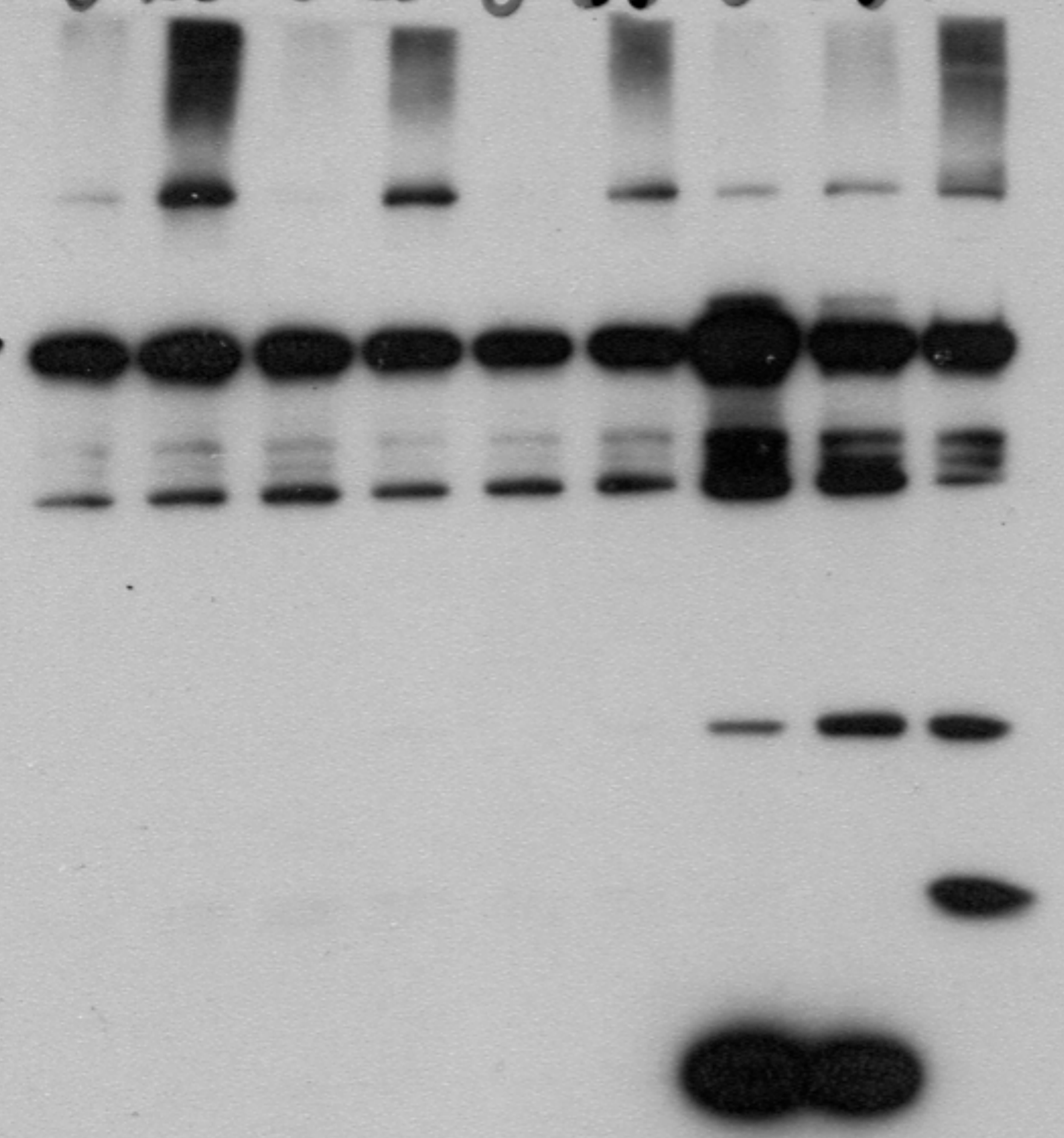

gel 3

samples were prepared in duplicate and loaded on 2 gels (gel 2 and 3)

used in the paper

22RV1 / PC3 CELLS

FIGURE 5 PANEL D

Exposure for VINCULIN used in the paper (1 sec)

22RV1 PC3

MT 63-78 (uM): 0 2.5 0 2.5

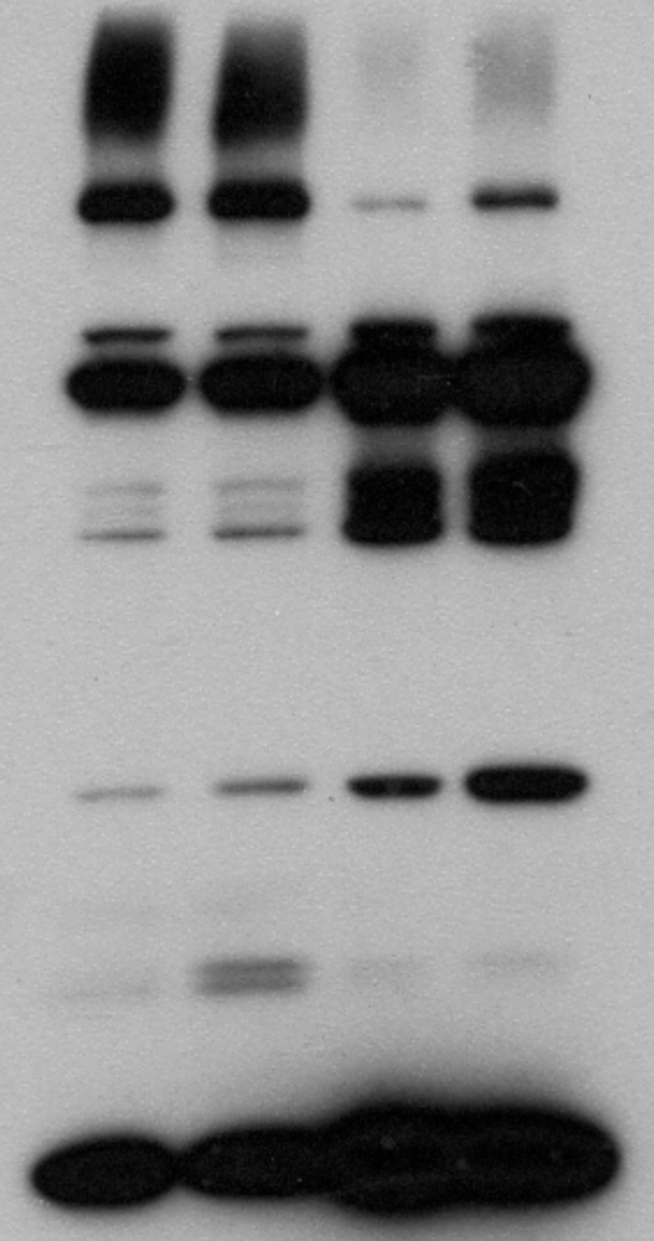

gel 1 PART 2

samples were prepared in duplicate and loaded on gel 1.

Quick

8.22.13

used in the paper

1

STRA

22RV1 / PC3

FIGURE 5 PANEL D

exposure for p-Histone H3 used in the paper

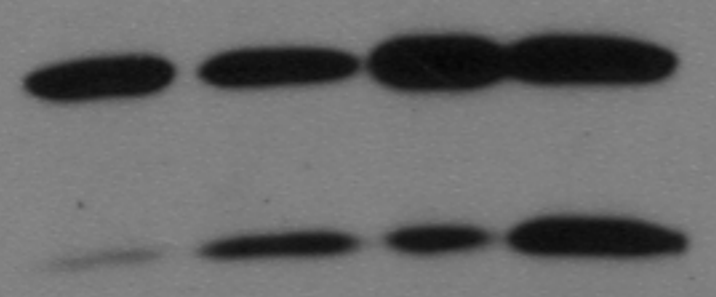

8.23.13 Re blot gel 1 part 2

FIGURE 5 PANEL D

Exposure for (P) ATM used in the paper

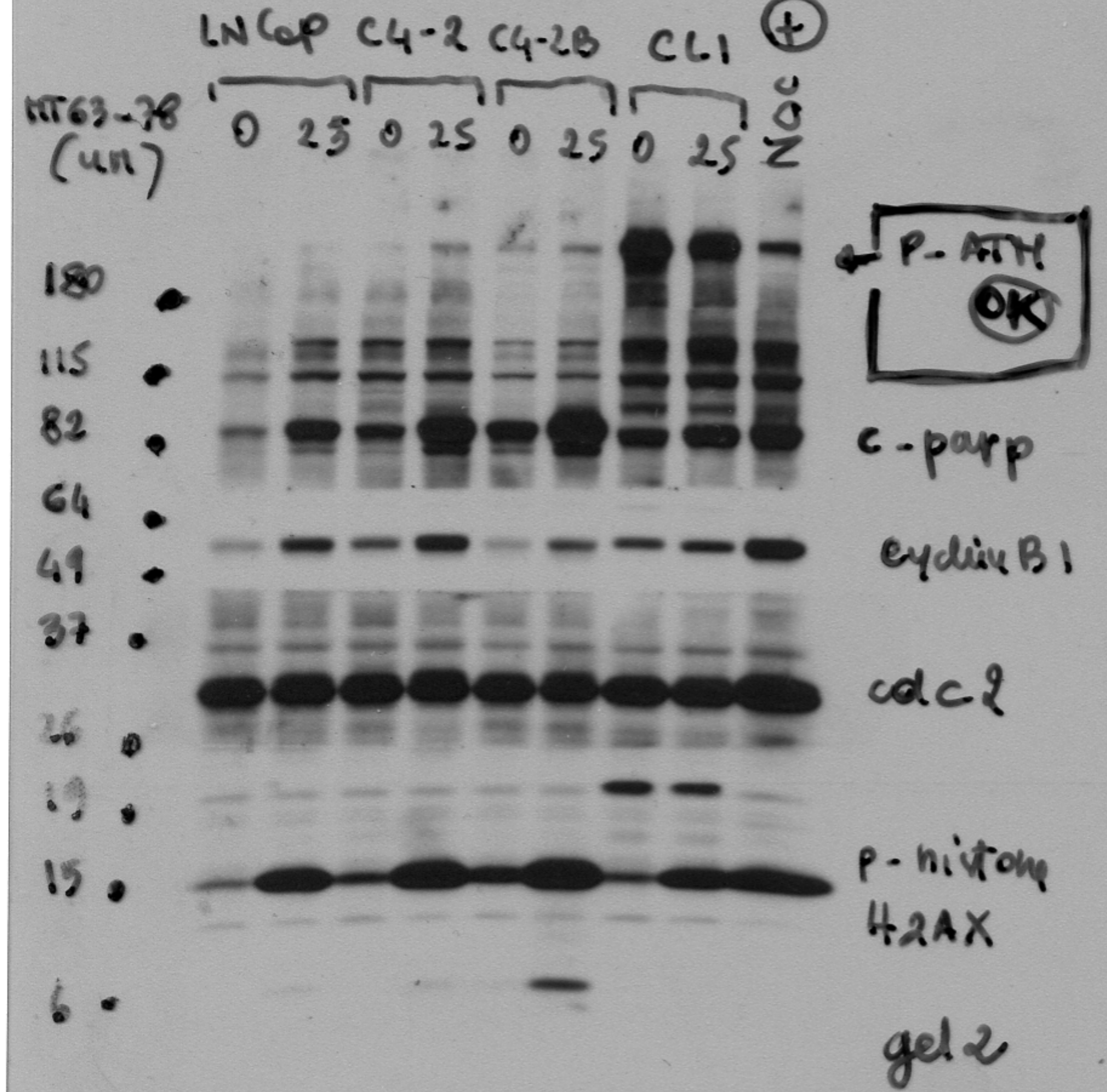

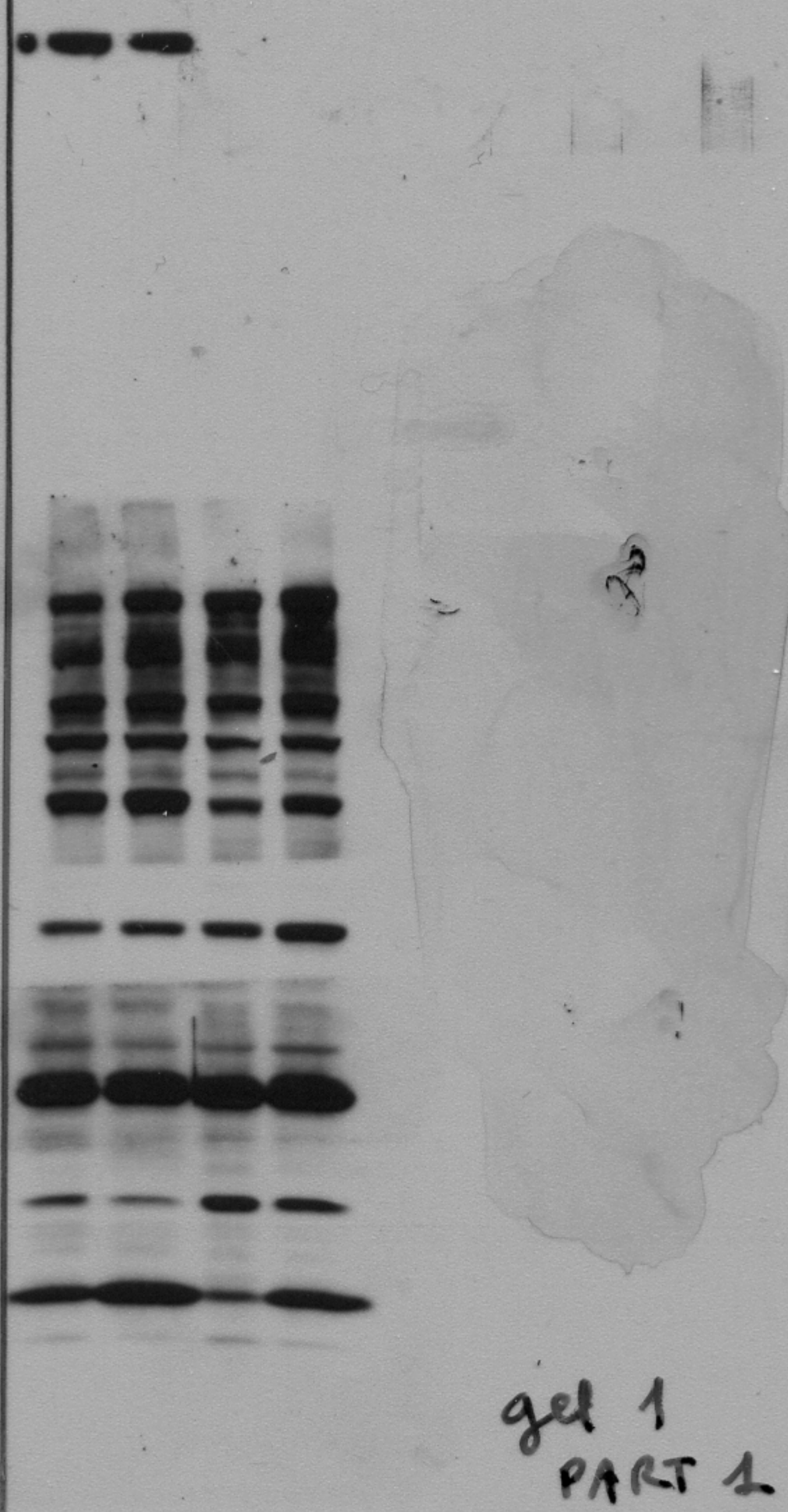

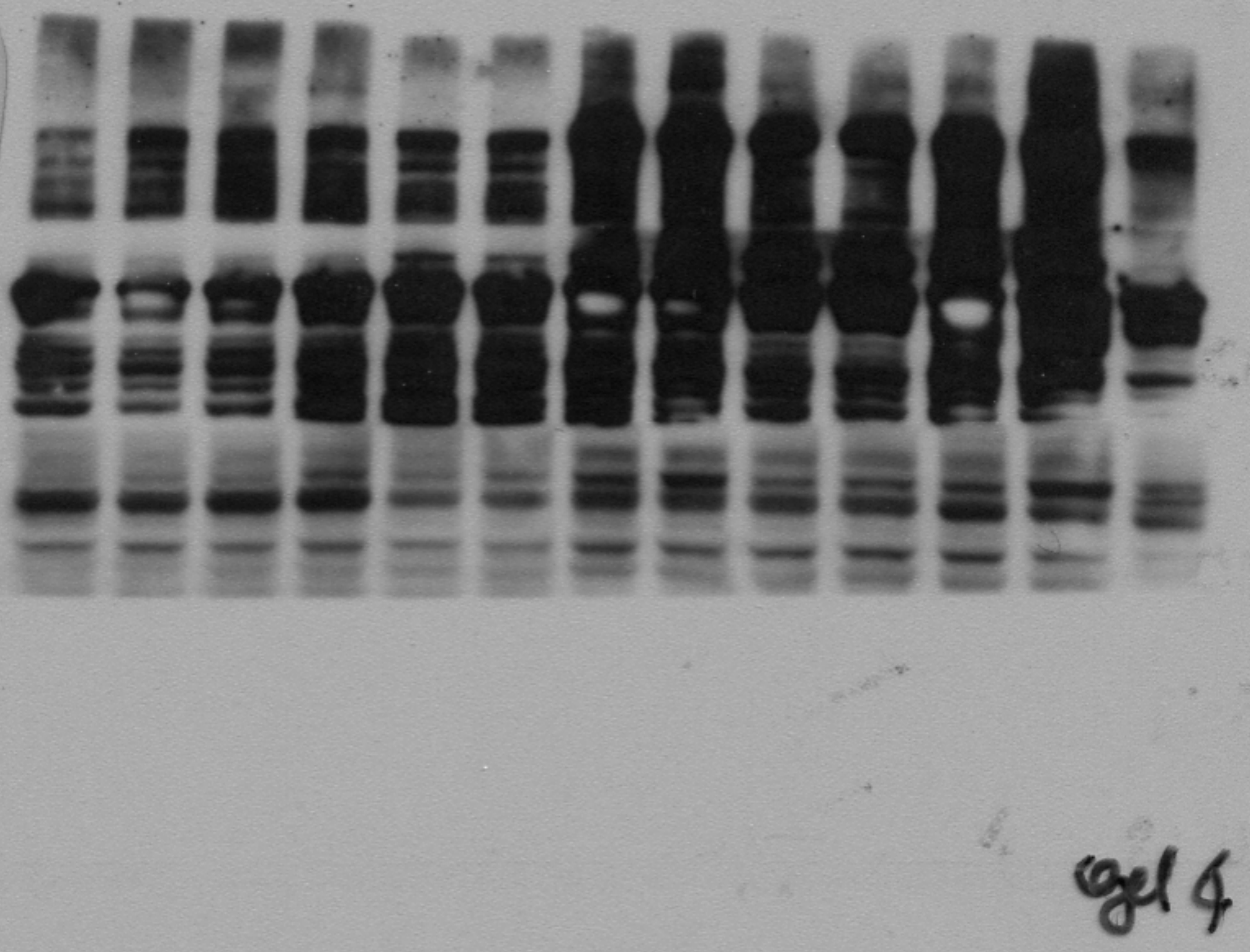

Samples were prepared in duplicate and loaded on 2 gels (gel 2 and 3)

USED IN the paper.

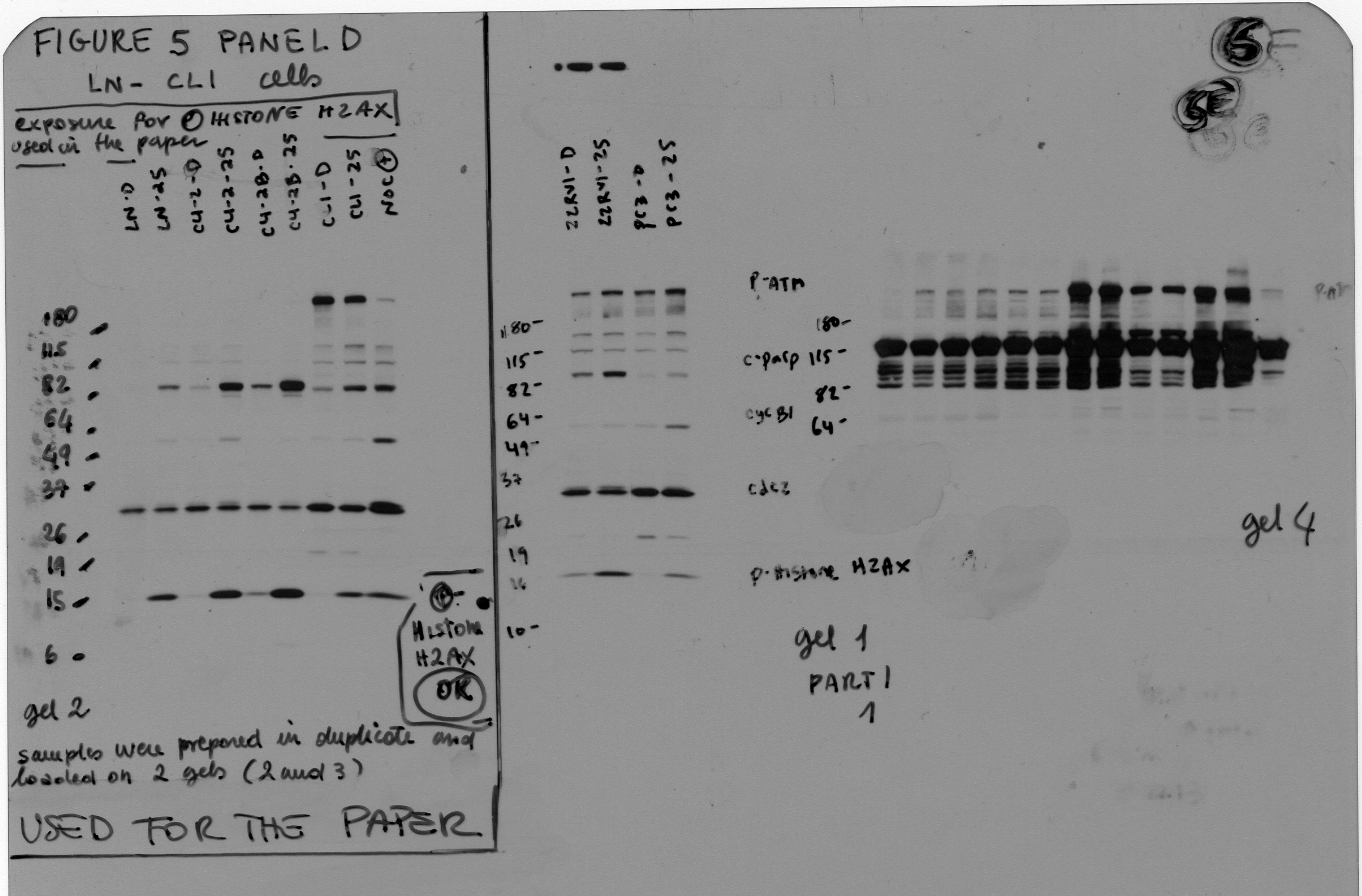
FIGURE 5 PANEL D
LN- CL1 cells
exposure for ⊕ HISTONE H2AX used in the paper
LN-D
LN-25
C4-2-D
C4-2-25
C4-2B-D
C4-2B-25
CL1-D
CL1-25
NOC⊕
180
115
82
64
49
37
26
19
15
6
⊕ HISTONE H2AX OK
gel 2
samples were prepared in duplicate and loaded on 2 gels (2 and 3)
USED FOR THE PAPER
22RV1-D
22RV1-25
PC3-D
PC3-25
180
115
82
64
49
37
26
19
16
10
P-ATM
C-parp
cyc B1
cdc2
P-Histone H2AX
180
115
82
64
gel 1
PART 1
1
gel 4
5E
5E

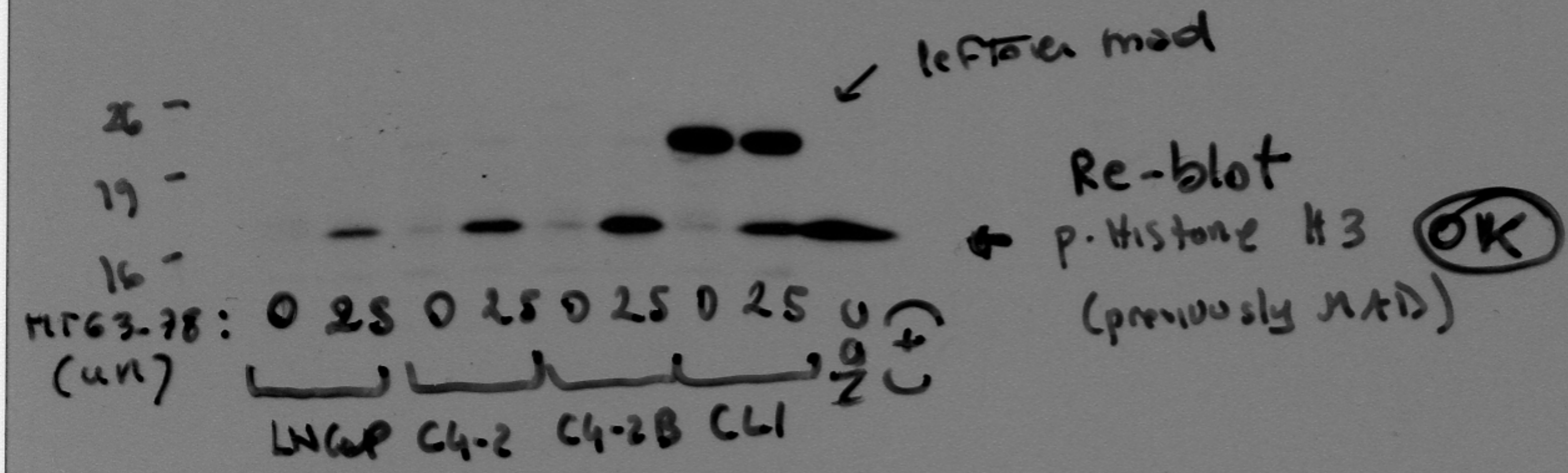

8.23.13 Re-blot gel 3

Samples were prepared in duplicate and loaded on 2 gels (gel 2 and 3)
