# Supplementary material for: A novel direct activator of AMPK inhibits prostate cancer growth by blocking lipogenesis
Source: EMBO Mol Med. 2014 Feb 4;6(4):519–38. doi: 10.1002/emmm.201302734 (PMC3992078; doi:10.1002/emmm.201302734)
Supplement: Supplementary file 6 [file emmm0006-0519-sd6.pdf]

LNCaP p28
10-20% gel invitrogen

FIGURE 6 PANEL C - LNCaP

mds tabs

Exposure For P-ACC

1 = dmso
2 = MT 63-78 25uM
3 = Rapa 10nM
4 = dmso + CQ 10uM
5 = MT 63-78 + CQ 10uM
6 = Rapa + CQ 10uM
7 = dmso + Bafilo 400nM (6h)
8 = MT63-78 + Bafilo 400nM (6h)
9 = Rapa + Bafilo 400nM (6h)

LNCaP treatment 24h

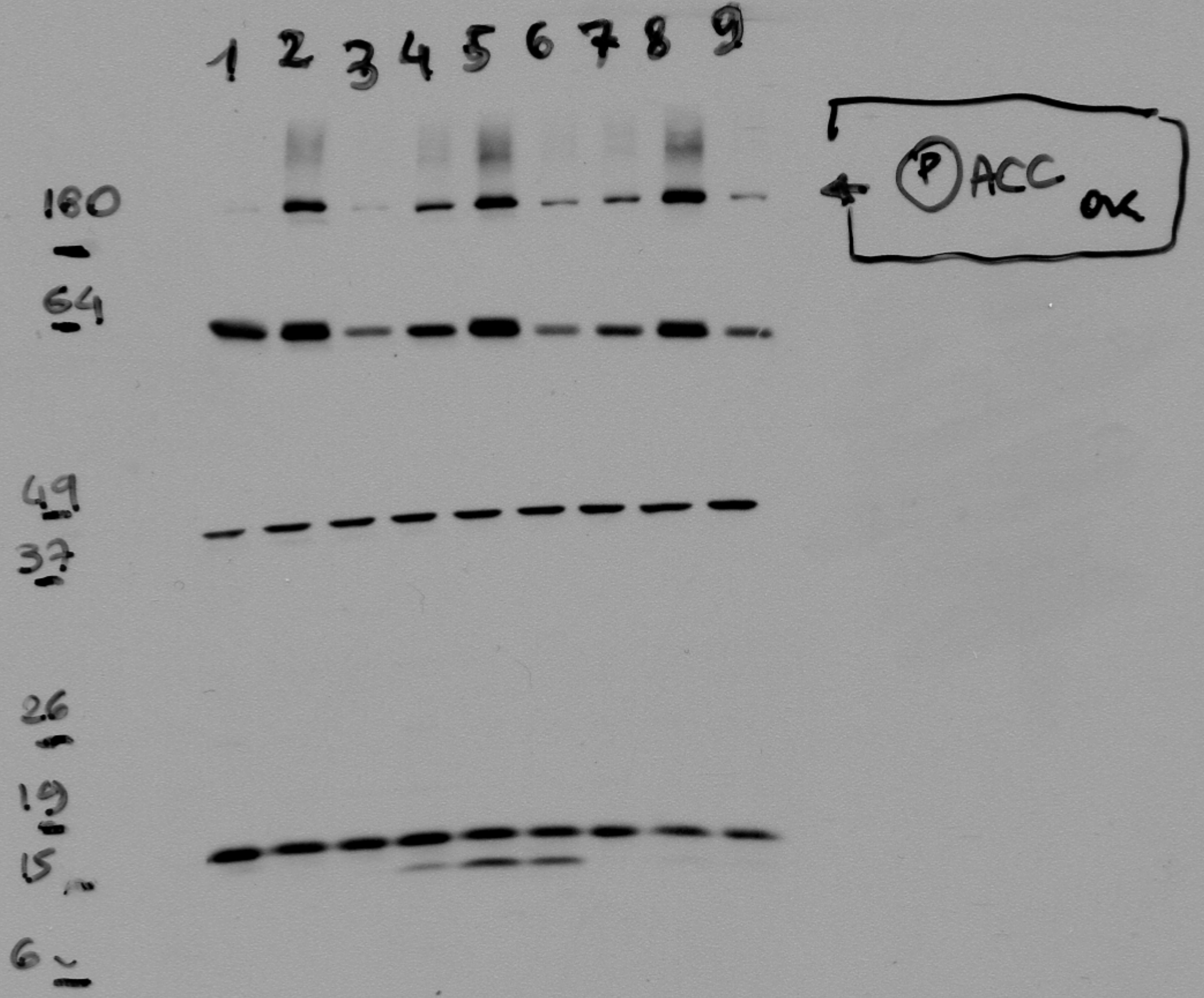

Exp. Time 5sec
05/3/2013

(8)

Figure 6 panel C [redacted]

LNCaP p28

gel 4-20%

FOR LC3 I/II long exposure

LNCaP

1 2 3 4 5 6 7 8 9

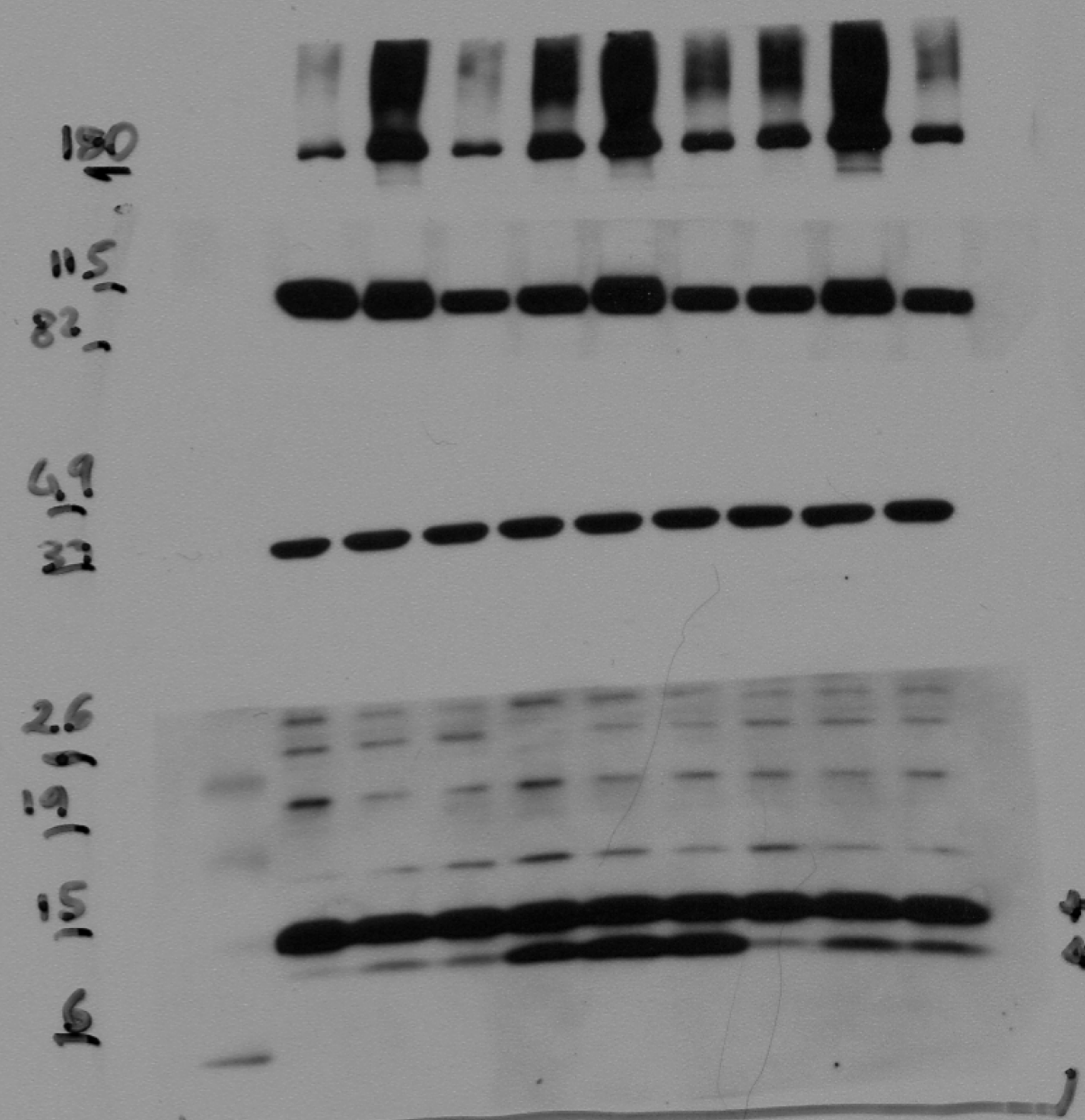

LNCaP treatment 24hr

1 = DMSO
2 = MT63-78 25uM
3 = Rapa 10nM

4 = DMSO + CQ 10uM
5 = MT63-78 25uM + CQ 10uM
6 = Rapa 10nM + CQ 10uM

7 = DMSO + Bafilo 400nM (6hr)
8 = MT63-78 25uM + Bafilo (6hr)
9 = Rapa 10nM + Bafilo (6hr)

Exposure Time 2 minutes

STRATAGENE

LNCaP p28
gel 4-20% invitrogen

STRATAGEN

Figure 6 PANEL C - LNCaP

EXPOSURE FOR
- β-ACTIN
- LC3 I/II (short exposure)

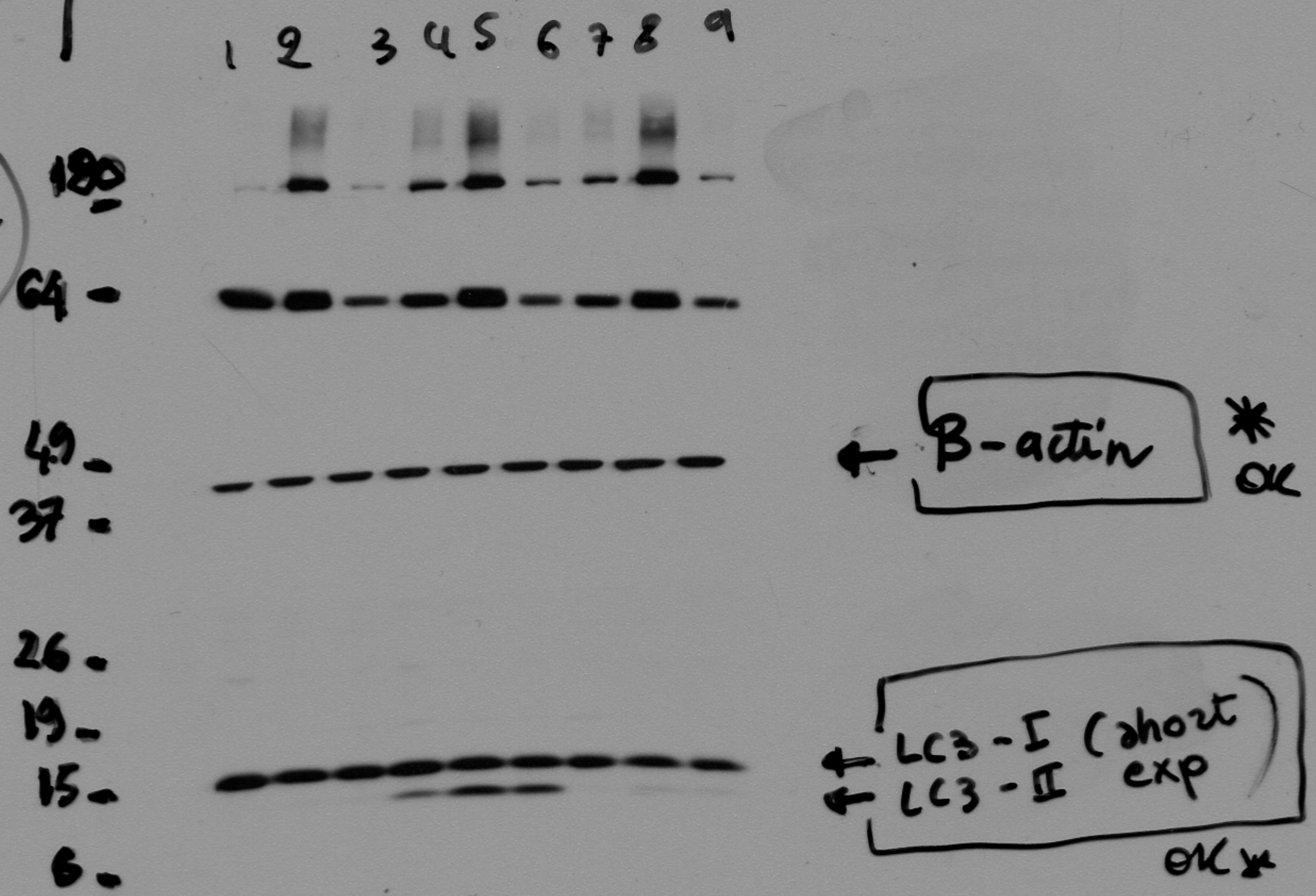

LNCaP 24hr treatment

1 = dmso
2 = MT 63-78 25uM
3 = Rapa 10nM
4 = dmso + CQ 10uM
5 = MT 63-78 + CQ 10uM
6 = Rapa + CQ 10uM
7 = dmso + Bafilo 400nM (6h)
8 = MT 63-78 + Bafilo (6h)
9 = Rapa + Bafilo (6h)

15 sec ECL
05/3/2013

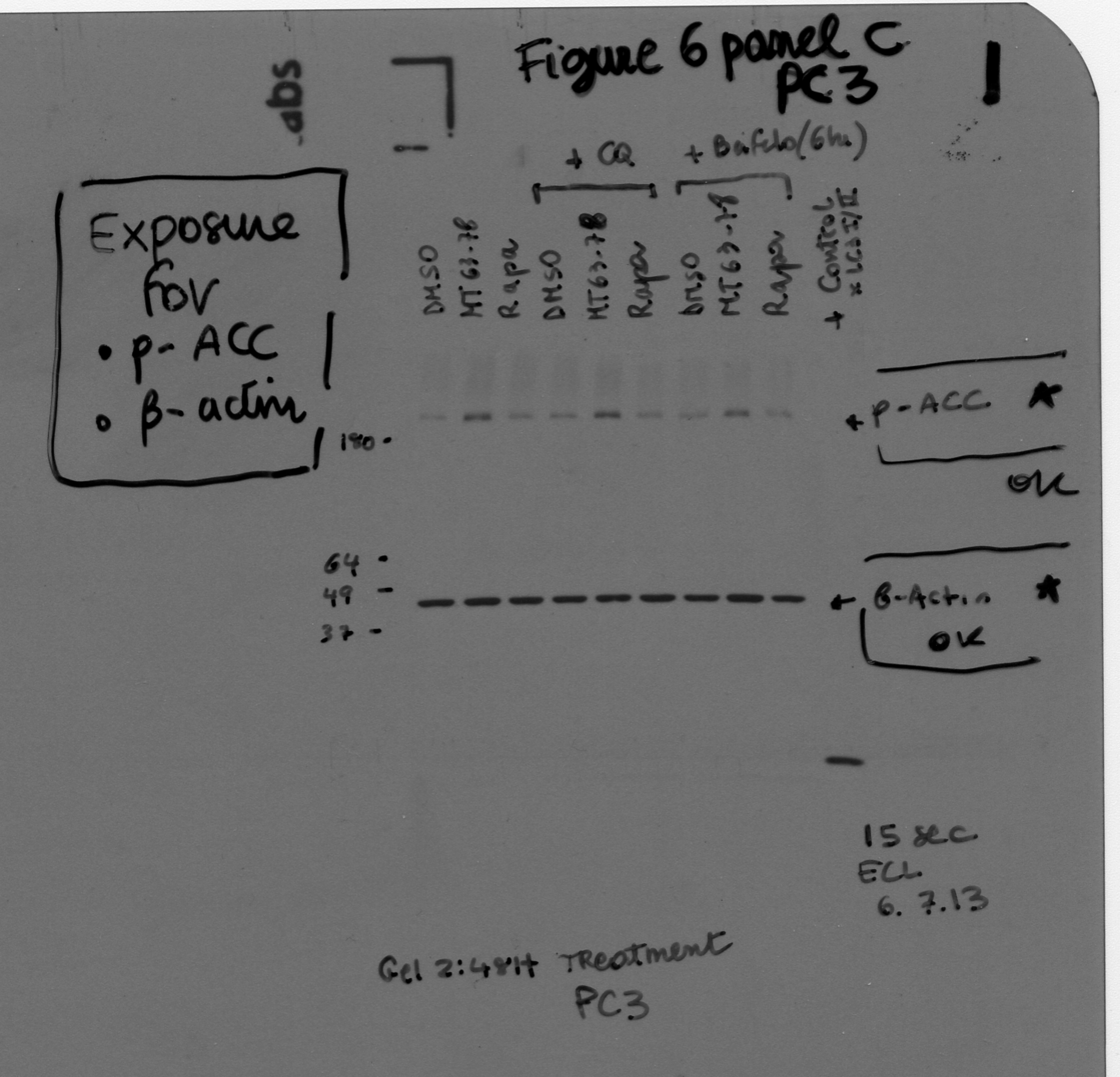
Figure 6 panel C
PC3
abs
Exposure for
• p-ACC
• β-actin
+ CQ
+ Bafilo(6hr)
DMSO
MT63-78
Rapa
DMSO
MT63-78
Rapa
DMSO
MT63-78
Rapa
+ Control
× LC3 I/II
190
64
49
37
← p-ACC ★
OK
← β-Actin ★
OK
15 sec
ECL
6. 7.13
Gel 2: 48H Treatment
PC3

Figure 6 panel C

PC3

2

+ CQ   + BafiLo

Exposure for LC3I/II Short

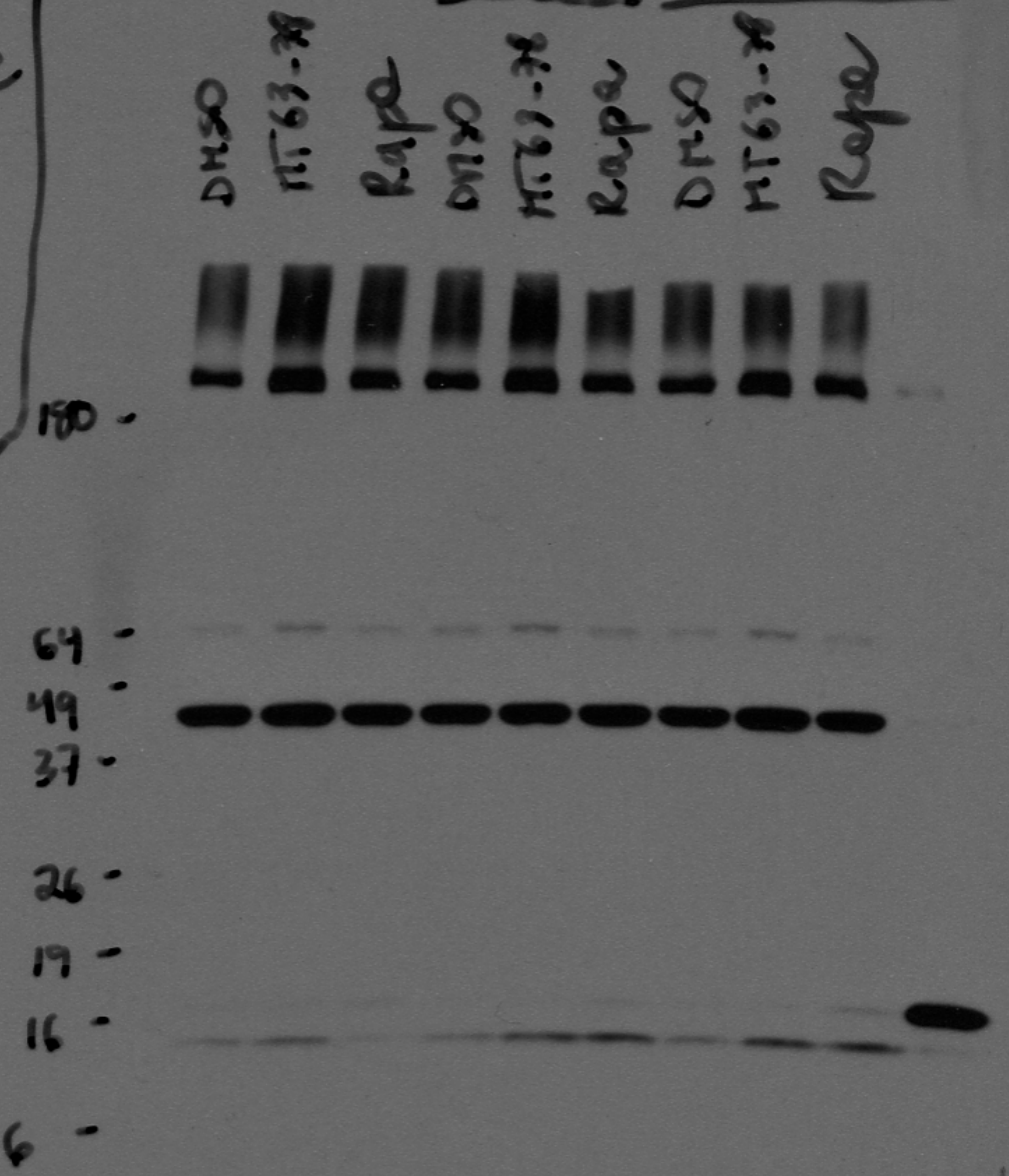

ok

← LC3 I/
↖ II
short exposure

15 sec
ECL
6.7.13
upper film

Gel 2: 48H Treatment PC3

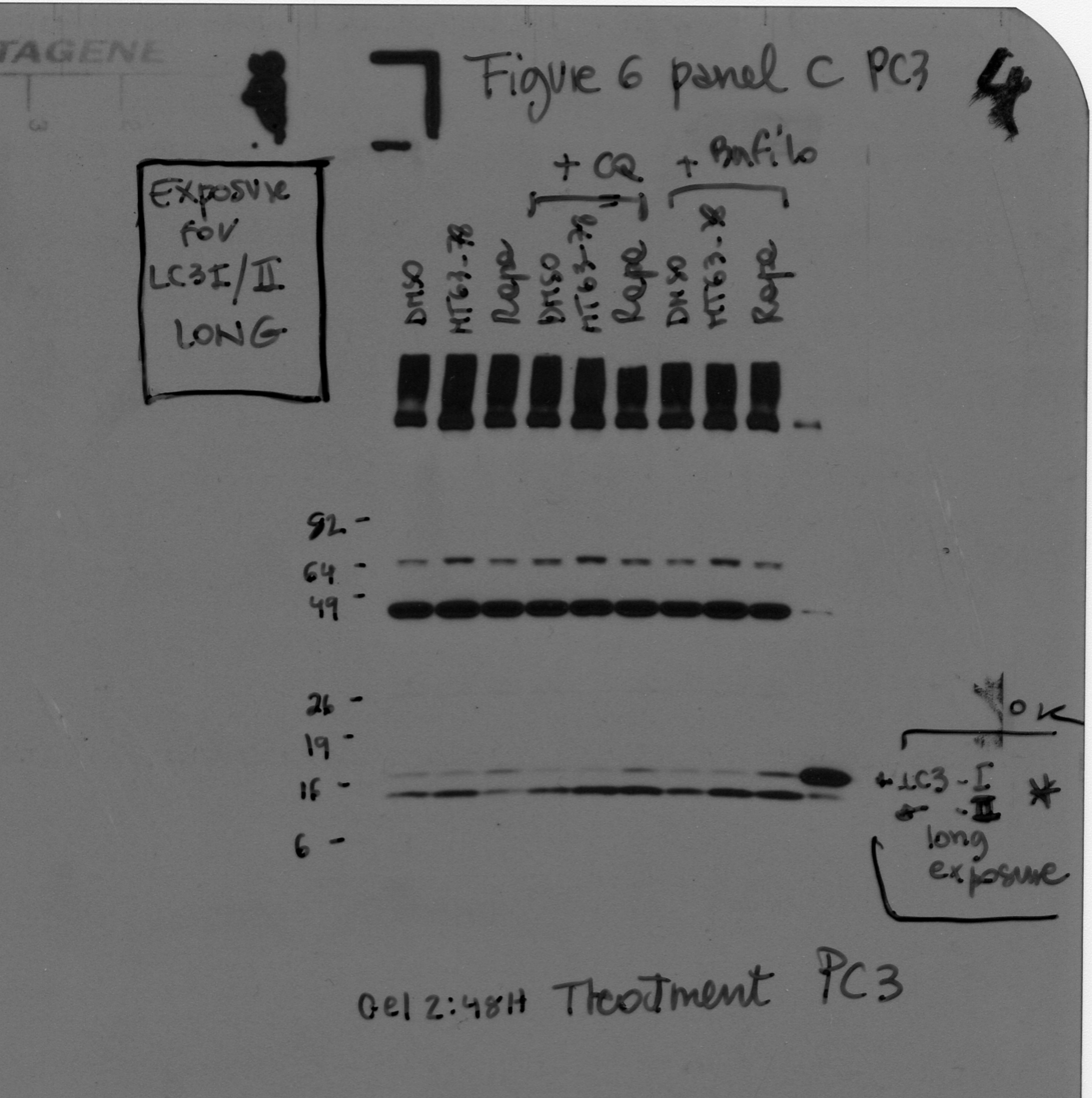

Figure 6 panel C PC3
4
Exposure for LC3I/II LONG
+ CQ
+ Bafilo
DMSO
Rapa
DMSO
Rapa
DMSO
Rapa
92
64
49
26
19
16
6
OK
LC3 - I
II
long exposure
Gel 2: 48H Treatment PC3
