# Supplementary material for: A novel direct activator of AMPK inhibits prostate cancer growth by blocking lipogenesis
Source: EMBO Mol Med. 2014 Feb 4;6(4):519–38. doi: 10.1002/emmm.201302734 (PMC3992078; doi:10.1002/emmm.201302734)
Supplement: Supplementary file 7 [file emmm0006-0519-sd7.pdf]

FIGURE 7 PANEL A
LEFT (LNCaP cells)

Exposure used for P-ACC in the paper (10 sec) 10.27.09

samples were prepared in quadruplicate and loaded on 4 gels

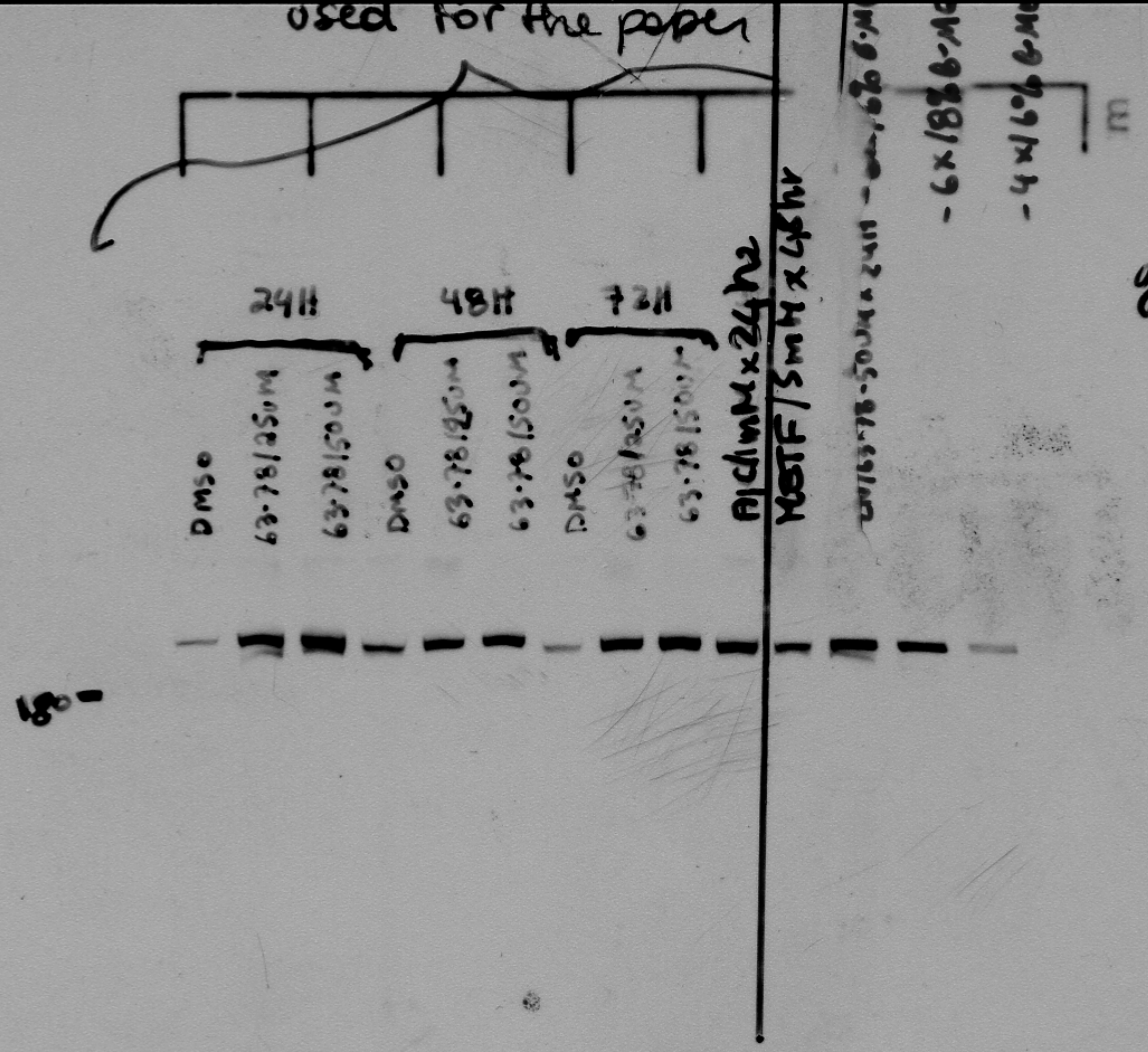

gel 1

(P) ACC

← (P) ACC • OK

(1)

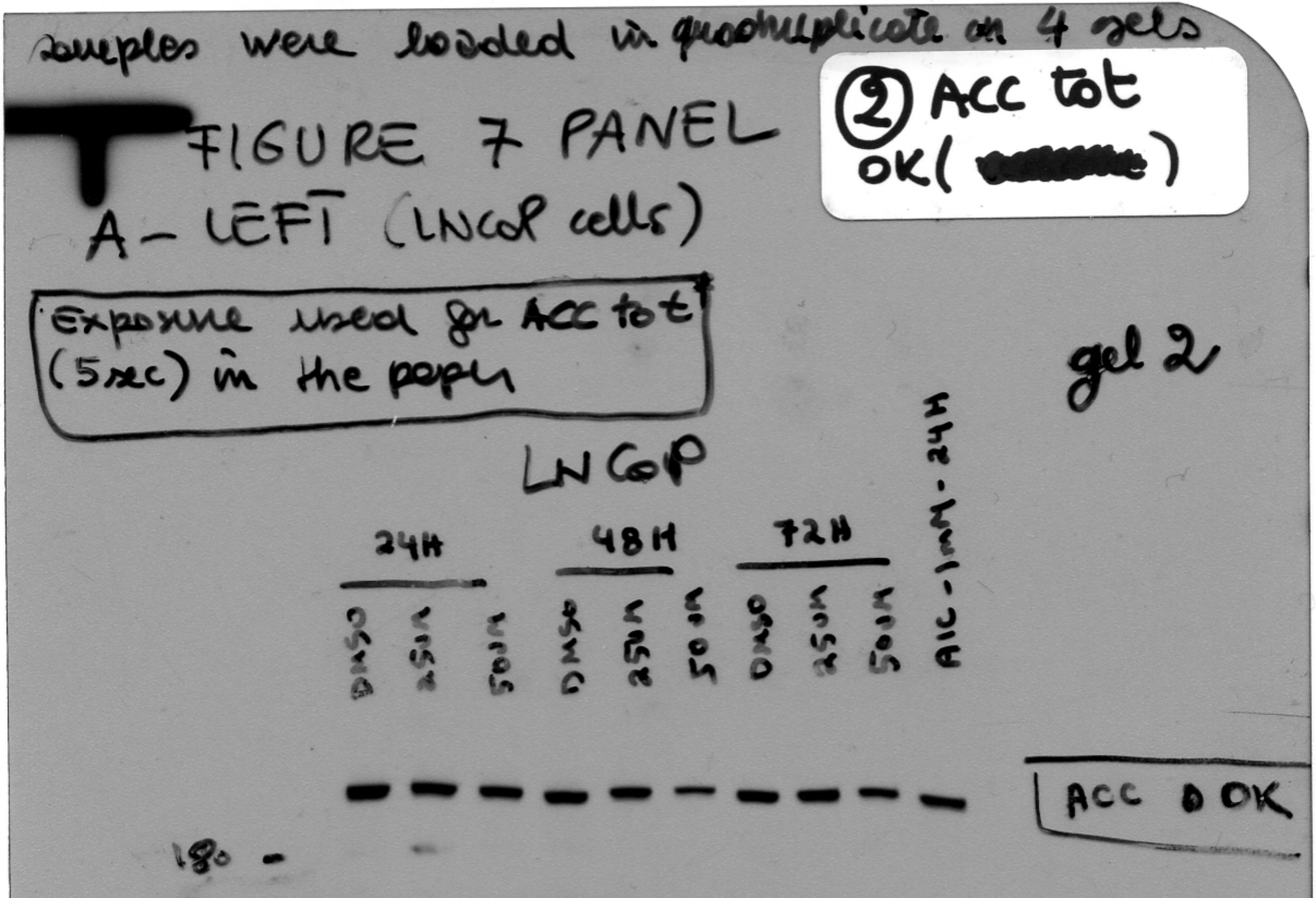

samples were loaded in quadruplicate on 4 gels
FIGURE 7 PANEL A - LEFT (LNCaP cells)
② ACC tot
OK([redacted])
Exposure used for ACC tot (5sec) in the paper
gel 2
LNCaP
24H
48H
72H
DMSO
25uM
50uM
DMSO
25uM
50uM
DMSO
25uM
50uM
AIC-1mM-24H
ACC OK
180 -

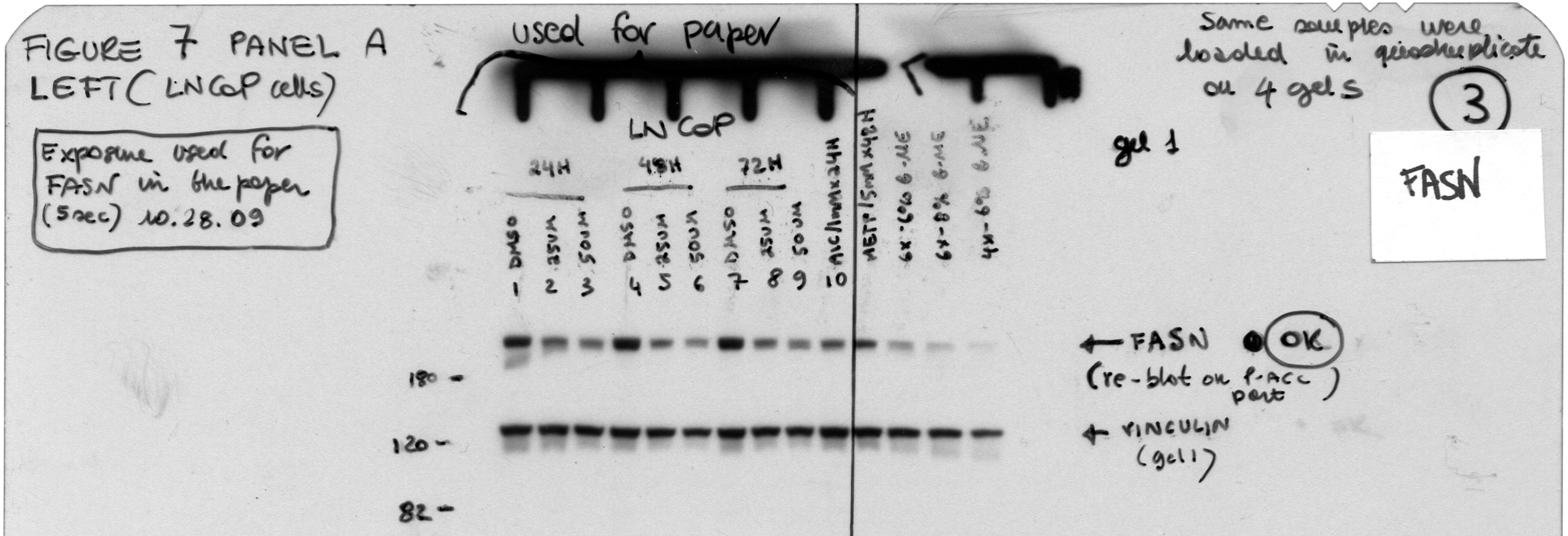
FIGURE 7 PANEL A
LEFT (LNCaP cells)
Exposure used for FASN in the paper (5sec) 10.28.09
used for paper
LNCoP
24H
48H
72H
1 DMSO
2 25uM
3 50uM
4 DMSO
5 25uM
6 50uM
7 DMSO
8 25uM
9 50uM
10 AIC/1mM x 24H
METF/5mM x 48H
6x-6% 6-ME
6x-8% 6-ME
4x-6% 6-ME
180 -
120 -
82 -
Same samples were loaded in quadruplicate on 4 gels
3
gel 1
FASN
← FASN OK
(re-blot on P-ACC part)
← VINCULIN (gel 1)

④ Ⓟ Raptor → better gel ☆

samples were prepared in quadruplicate and loaded on 4 gels

gel 2

FIGURE 7 PANEL A
LEFT (LNCaP cells)

Exposure for Ⓟ Raptor used in the paper

XT63-78	24hr			48hr			72hr			AICAR 1mM 24hr
	DMSO	25uM	50uM	DMSO	25uM	50uM	DMSO	25uM	50uM	

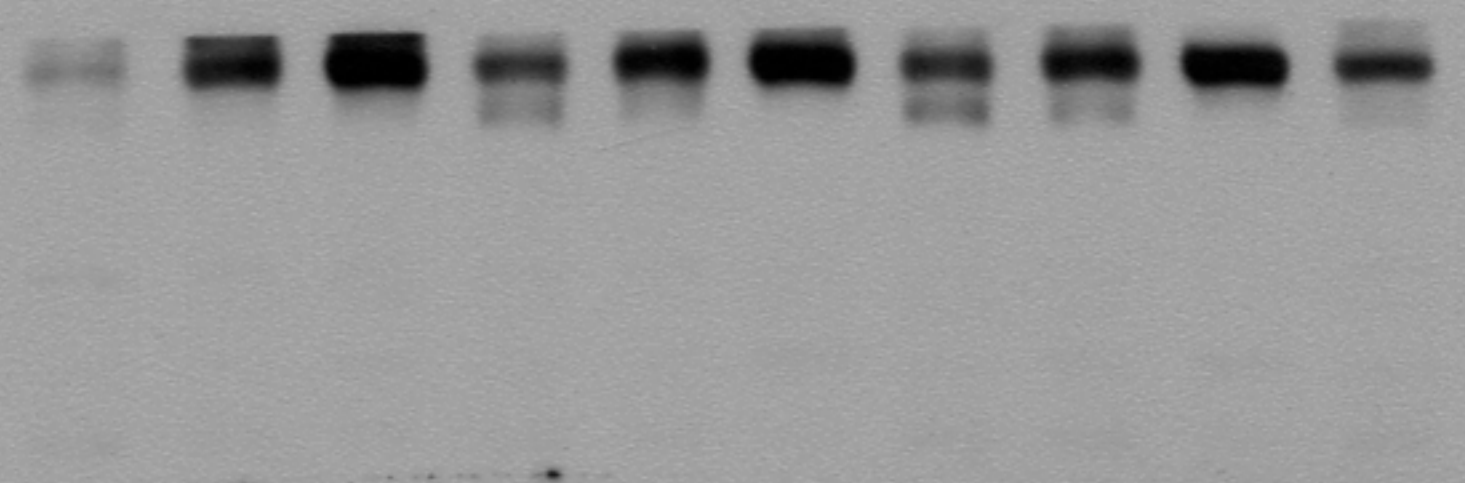

samples were prepared in quadruplicate and loaded on 4 gels

FIGURE 7, PANEL A LEFT

(LNCaP cells)

Exposure used for raptor total (exp 5 min) used in the paper

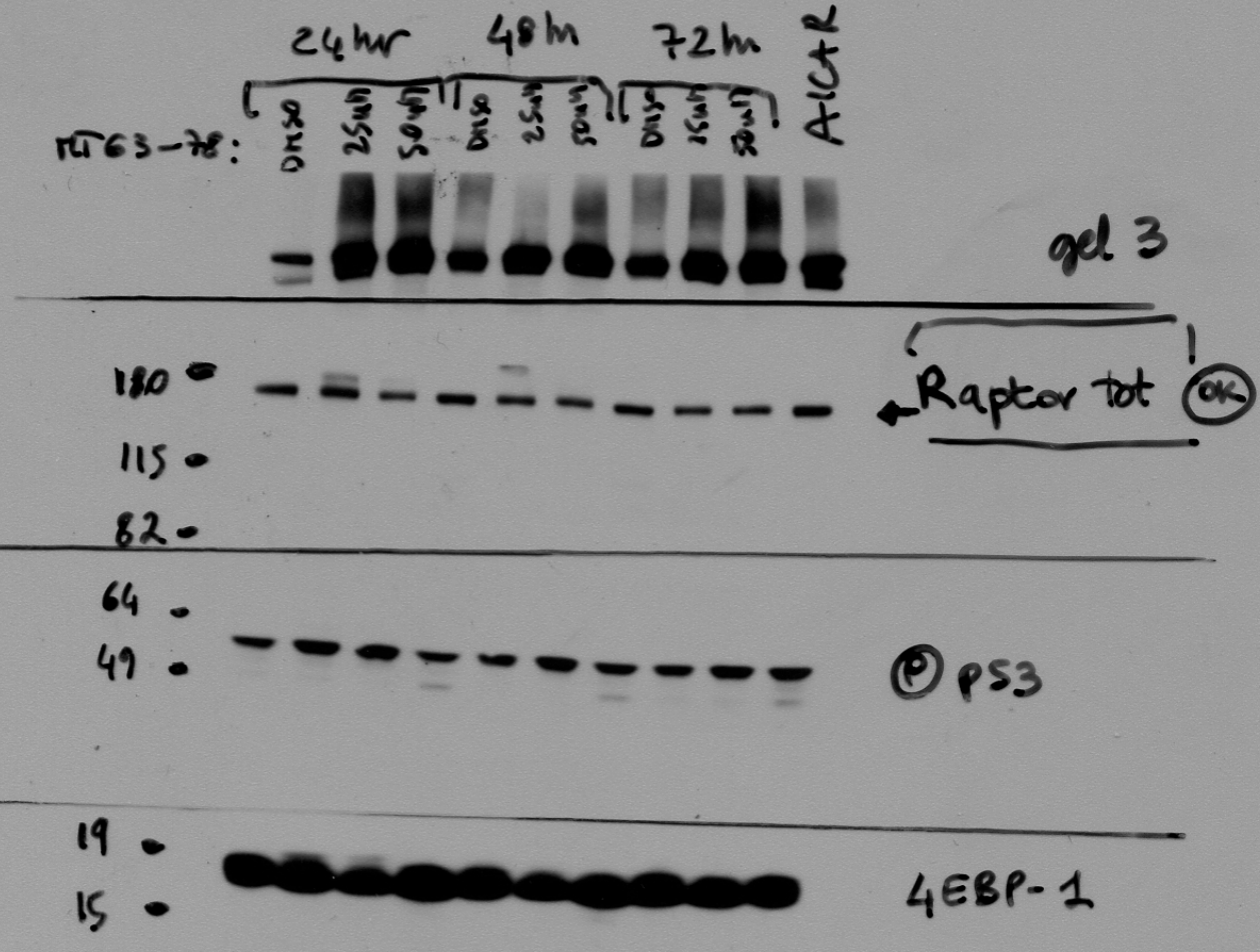

FIGURE 7 PANEL LEFT (LNCaP cells)

used x paper

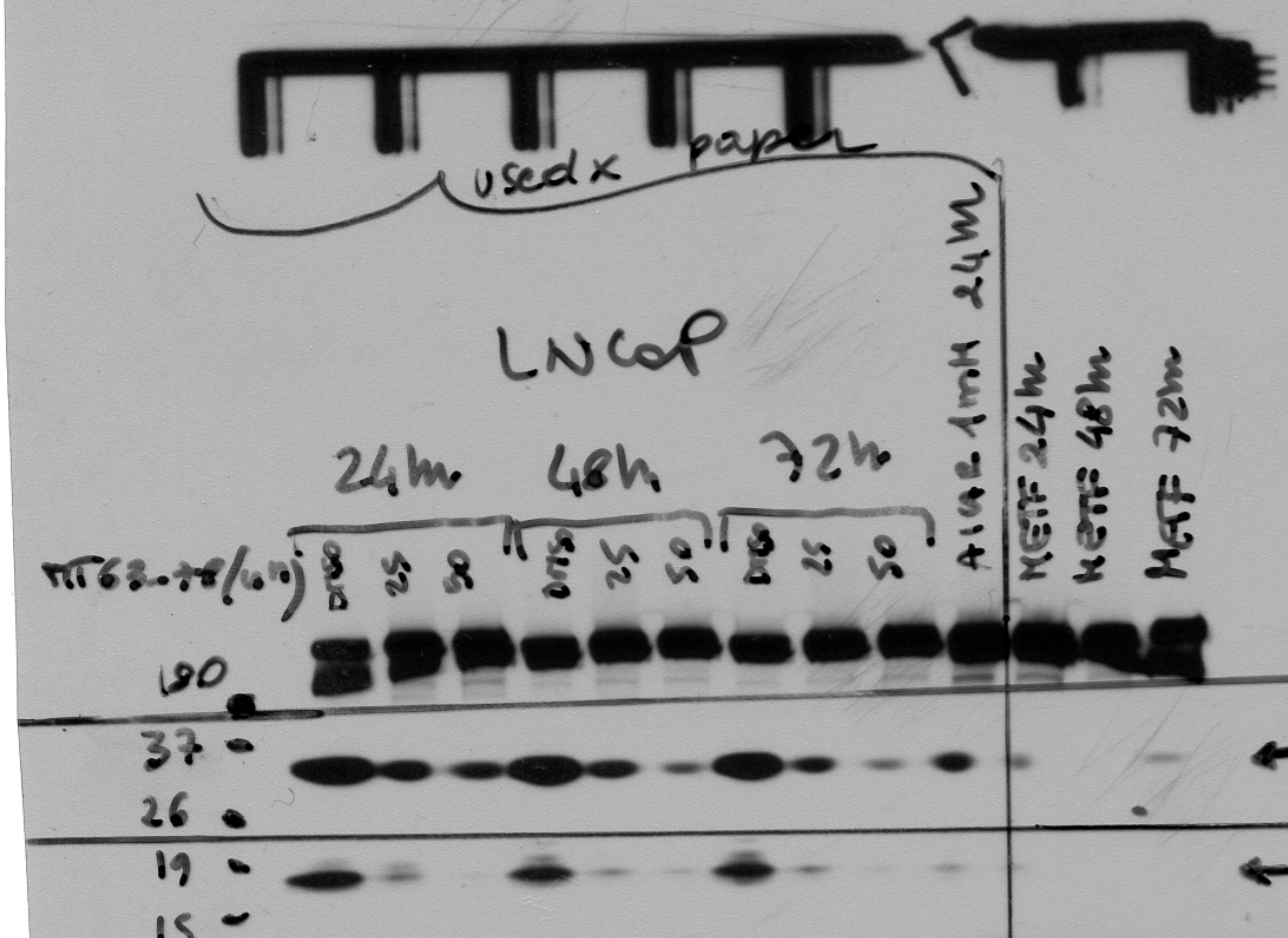

Exposure used for P S6RP and P 4EBP-1 in the paper (2.5 min)

ECL
2.5 min

Samples were prepared in quadruplicate and load on 4 gels.

P S6
P 4EBP1

FIGURE 7 PANEL A LEFT (LNCaP cells)

Exposure used for S6 total in the paper (15 sec)

3.7 –
26 –

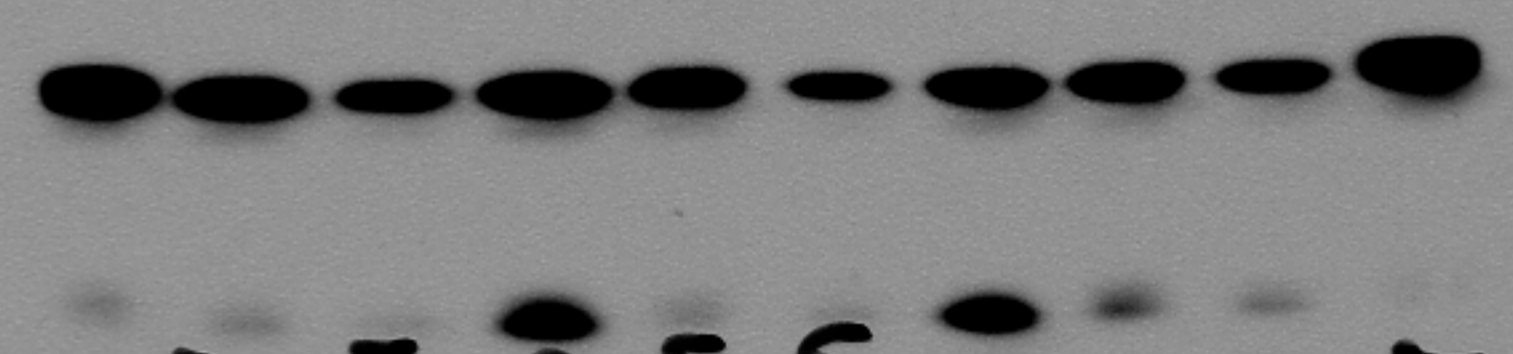

← S6 total (gel 3)

HT63-78

DMSO	25uM	50uM	DMSO	25uM	50uM	DMSO	25uM	50uM	AICAR 1mM x 24hr
24hr			48hr			72hr			

ECL
exp. 15 sec

samples were prepared in quadruplicate and loaded on 4 gels

S6 tot

samples were prepared in quadruplicate and loaded on 4 gels

FIGURE 7 PANEL A - LEFT (LNCaP)

Exposure used in the paper for 4EBP-1 (2.5 min)

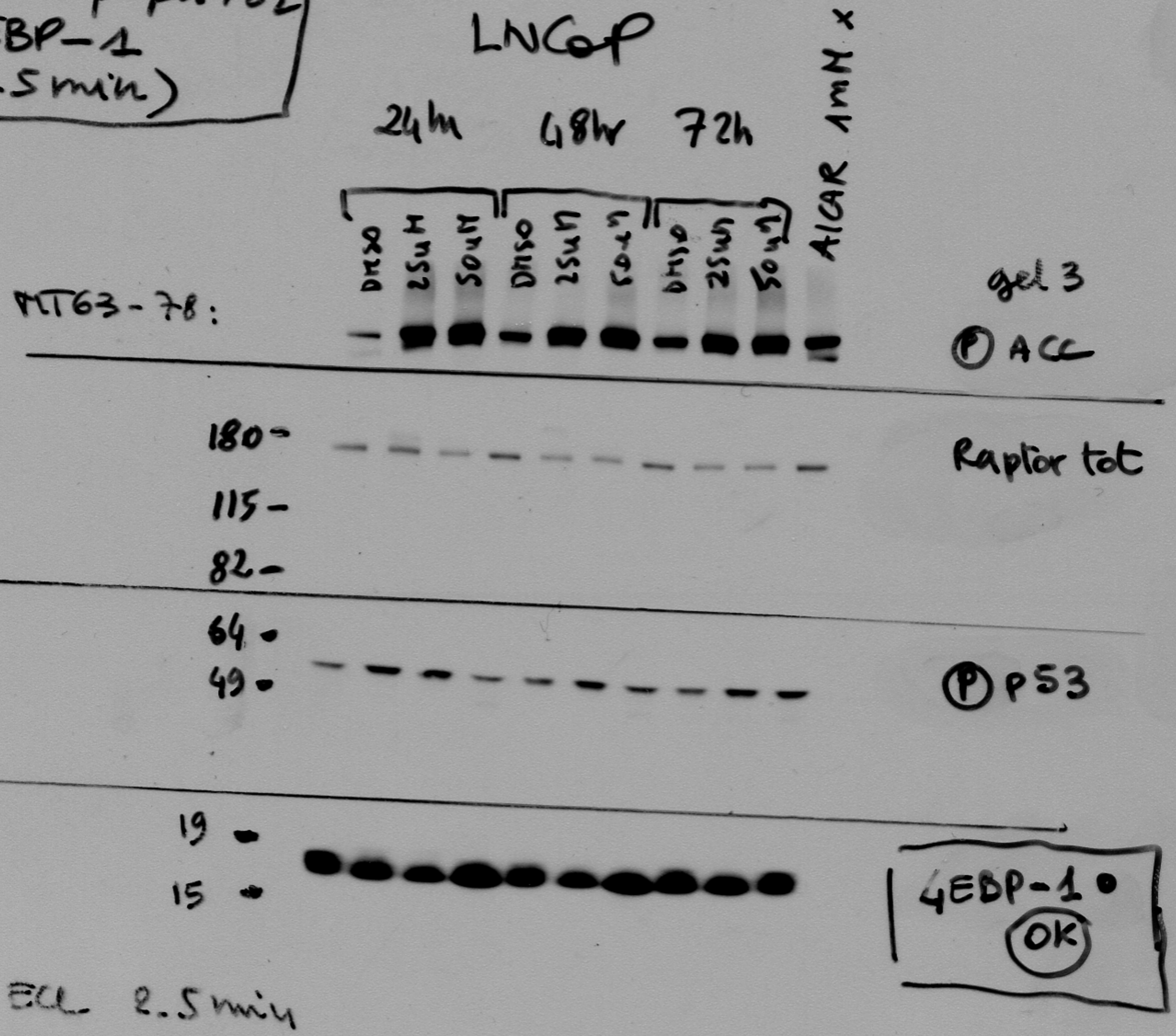

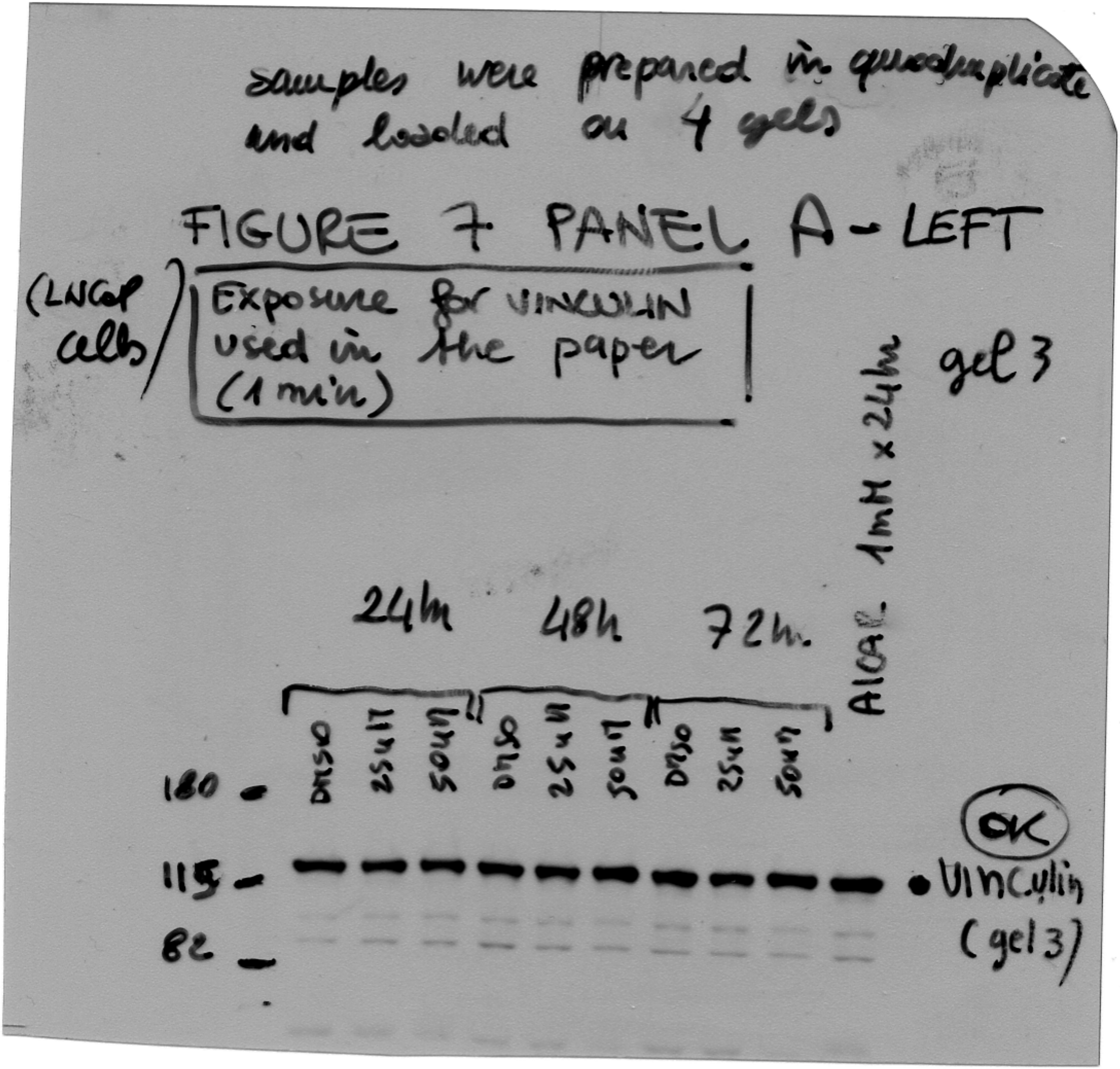
samples were prepared in quadruplicate and loaded on 4 gels
FIGURE 7 PANEL A - LEFT
(LNCaP cells)
Exposure for VINCULIN used in the paper (1 min)
gel 3
AICAR 1mM x 24h
24h
48h
72h
DMSO
25uM
50uM
DMSO
25uM
50uM
DMSO
25uM
50uM
180
115
82
OK
Vinculin
(gel 3)

FIGURE 7 PANEL A
RIGHT (PC3 cells)

Exposure for P-ACC used in the paper (30 sec)

used in the paper

24hr 48h 72h

HT 63-78 (uM): DMSO 25 50 DMSO 25 50 DMSO 25 50 | AICAR 1mM x24hr | KNRK | Hela (nuclear)

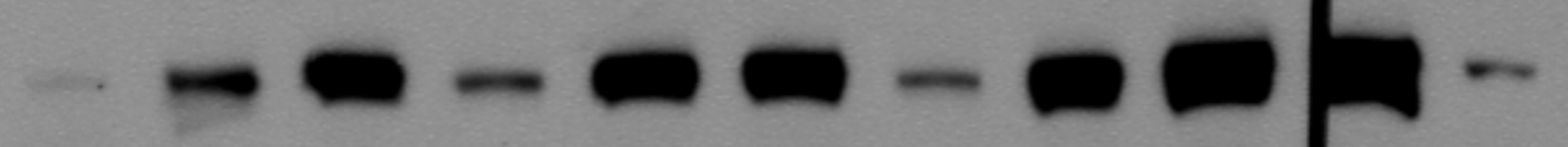

P)ACC ←

180 –

PC3 x 63-78 (9.10.09 samples)

gel 1

Samples were prepared in quintuplicate and loaded on 5 gels

PACC 1

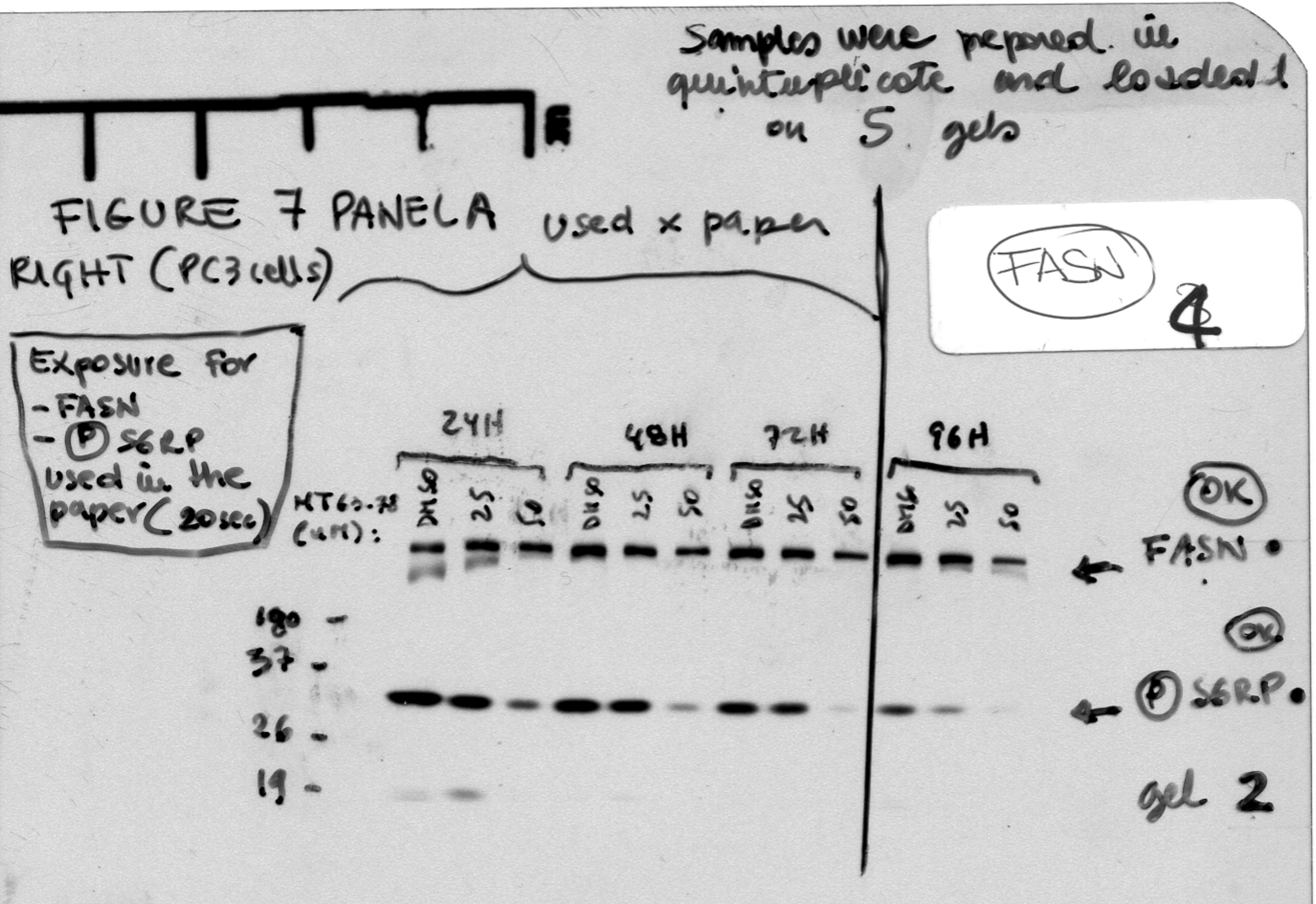

Samples were prepared in quintuplicate and loaded 1 on 5 gels
FIGURE 7 PANEL A
RIGHT (PC3 cells)
used x paper
FASN
4
Exposure for
- FASN
- (P) S6RP
used in the paper (20 sec)
24H
48H
72H
96H
DMSO
25
50
OK
FASN
180
37
26
19
(P) S6RP
gel 2

FIGURE 7 PANEL A RIGHT (PC3 cells)

Exposure for (P) Raptor used in the paper (30 sec)

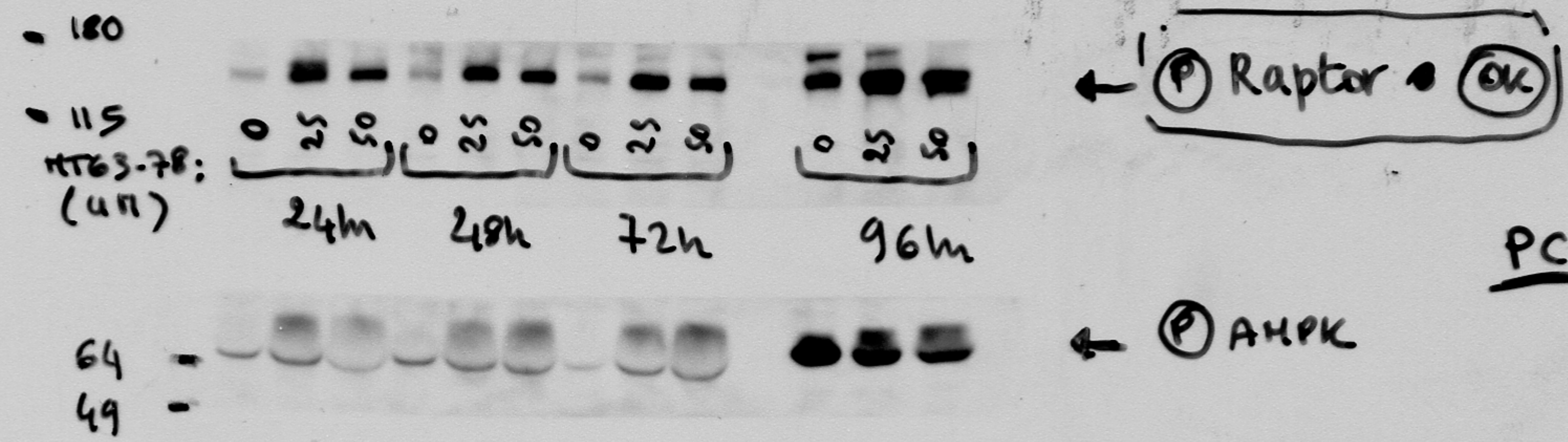

PC3

gel 4

Samples were prepared in quatuplicate and loaded on 4 gels

FIGURE 7
PANEL A RIGHT
(PC3 cells)

Exposure for Raptor used in the paper (5 min)

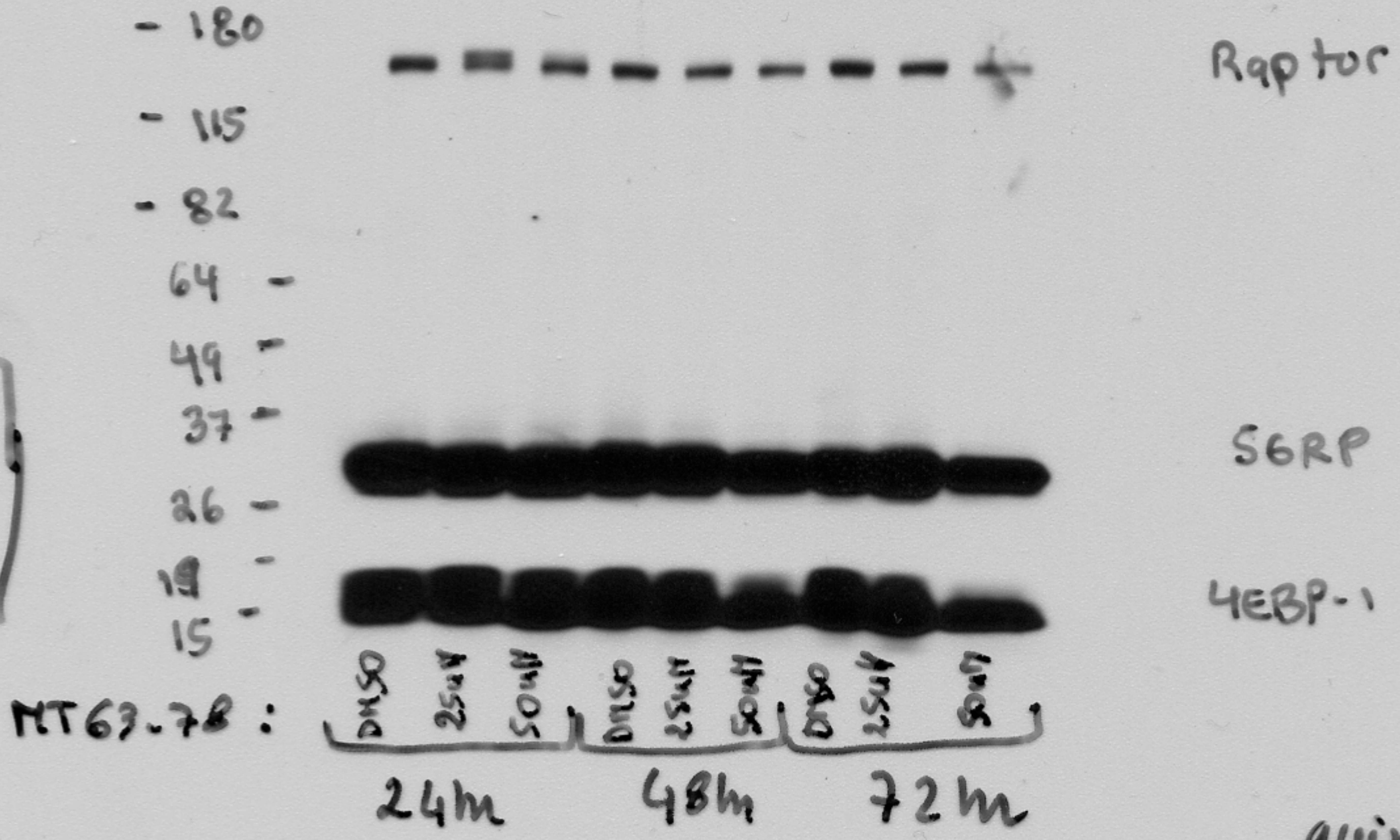

OK

gel 3

Samples were prepared in quintuplicate and loaded on 5 gels

Samples were loaded in quintuplicate and loaded on 5 gels

FIGURE 7
PANEL A RIGHT (PC3 cells)

exposure used for S6 in the paper (15sec)

gel 3

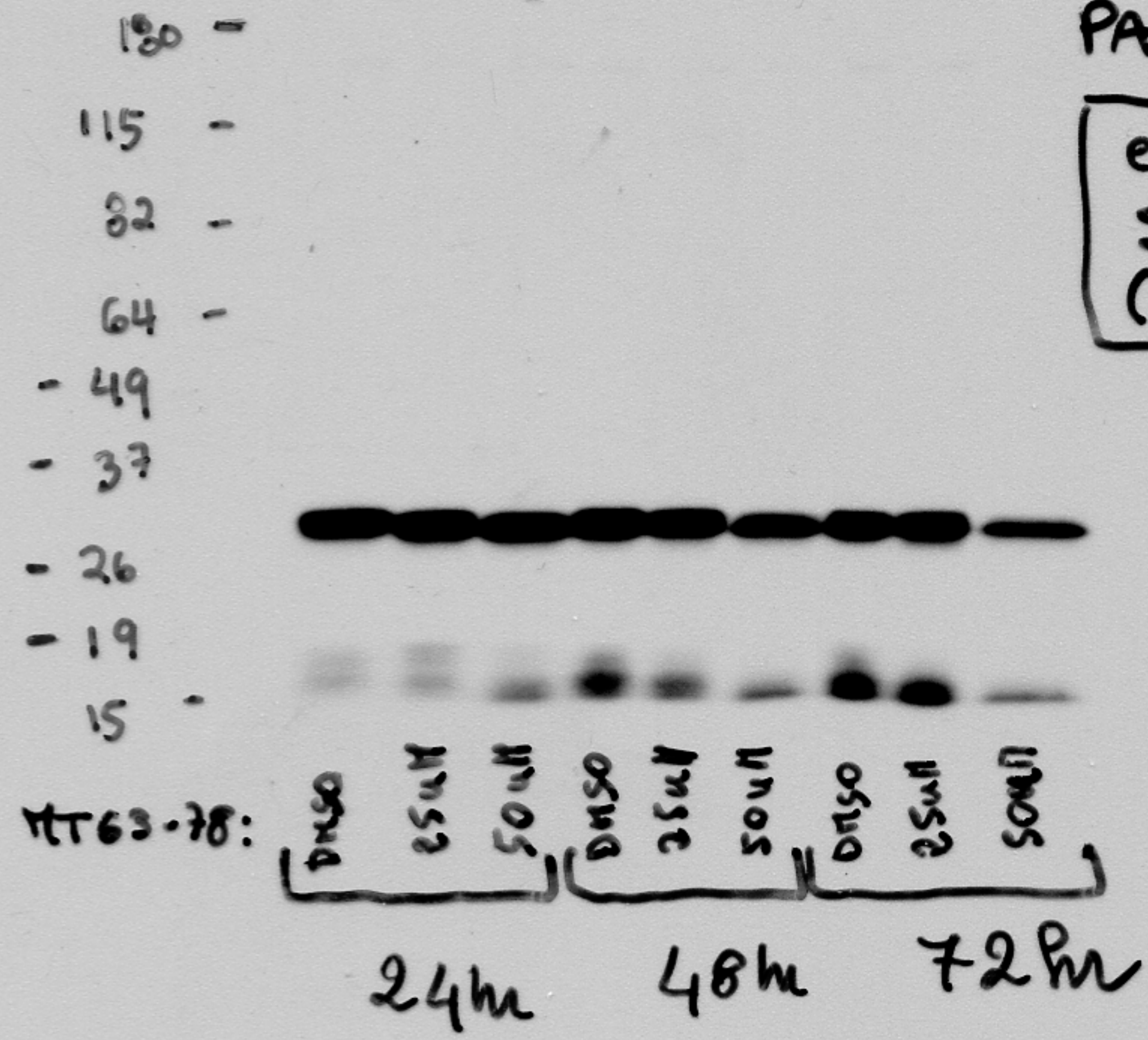

S6RP • OK

4EBP-1

FIGURE 7 PANEL A
RIGHT (PC3 cells)

Exposure for
(P) 4EBP-1 used
in the paper
(1 min)

HT63-78 (uM) :

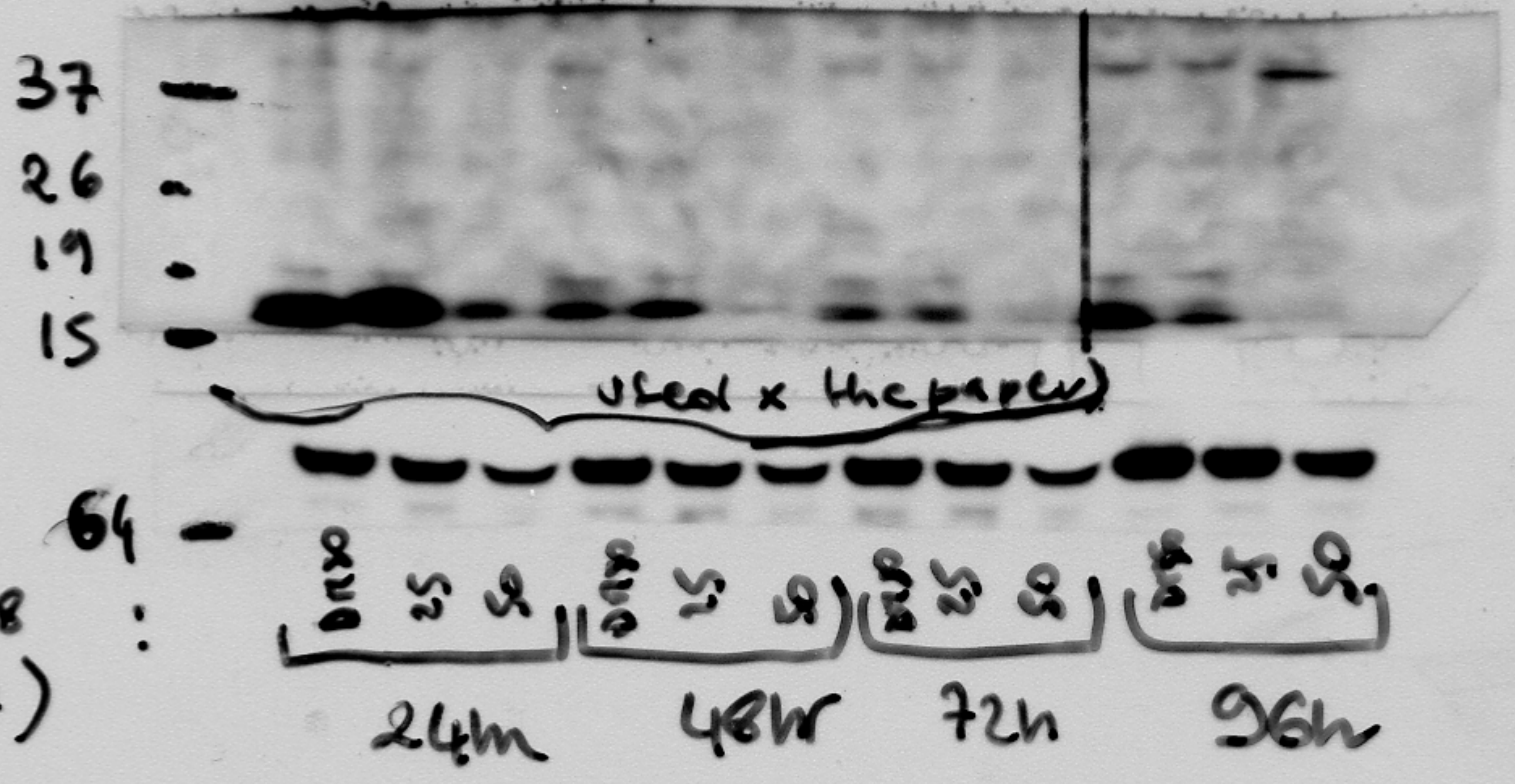

← (P) 4EBP-1 (OK)

← Beclin

gel 5

samples were prepared in quintuplicate
and loaded on 5 gels.

Samples were loaded in quintuplicate and lodded .5 gels

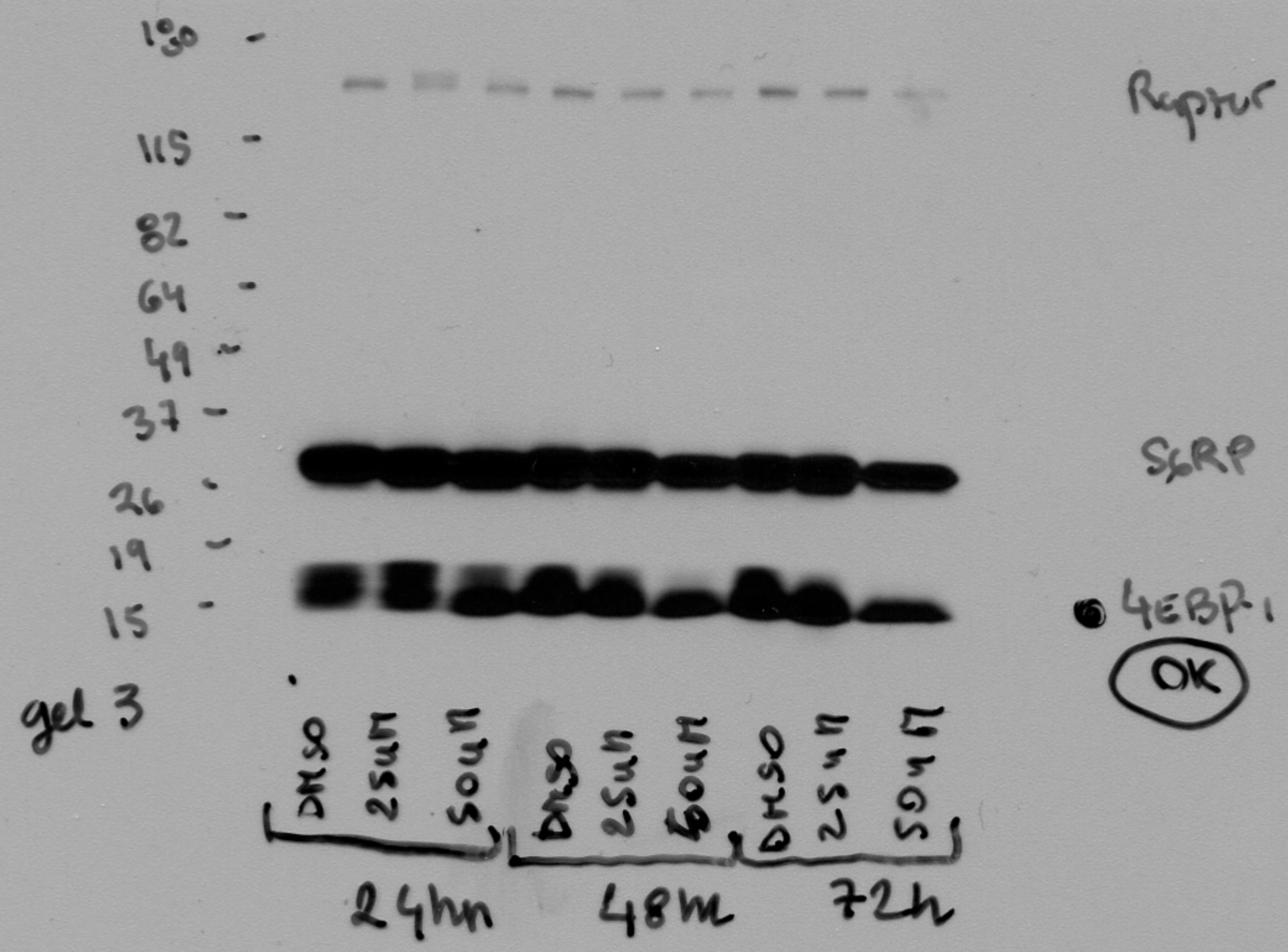

FIGURE 7 PANEL A RIGHT (PC3 cells)

Exposure used for 4EBP-1 in the paper (1 min)

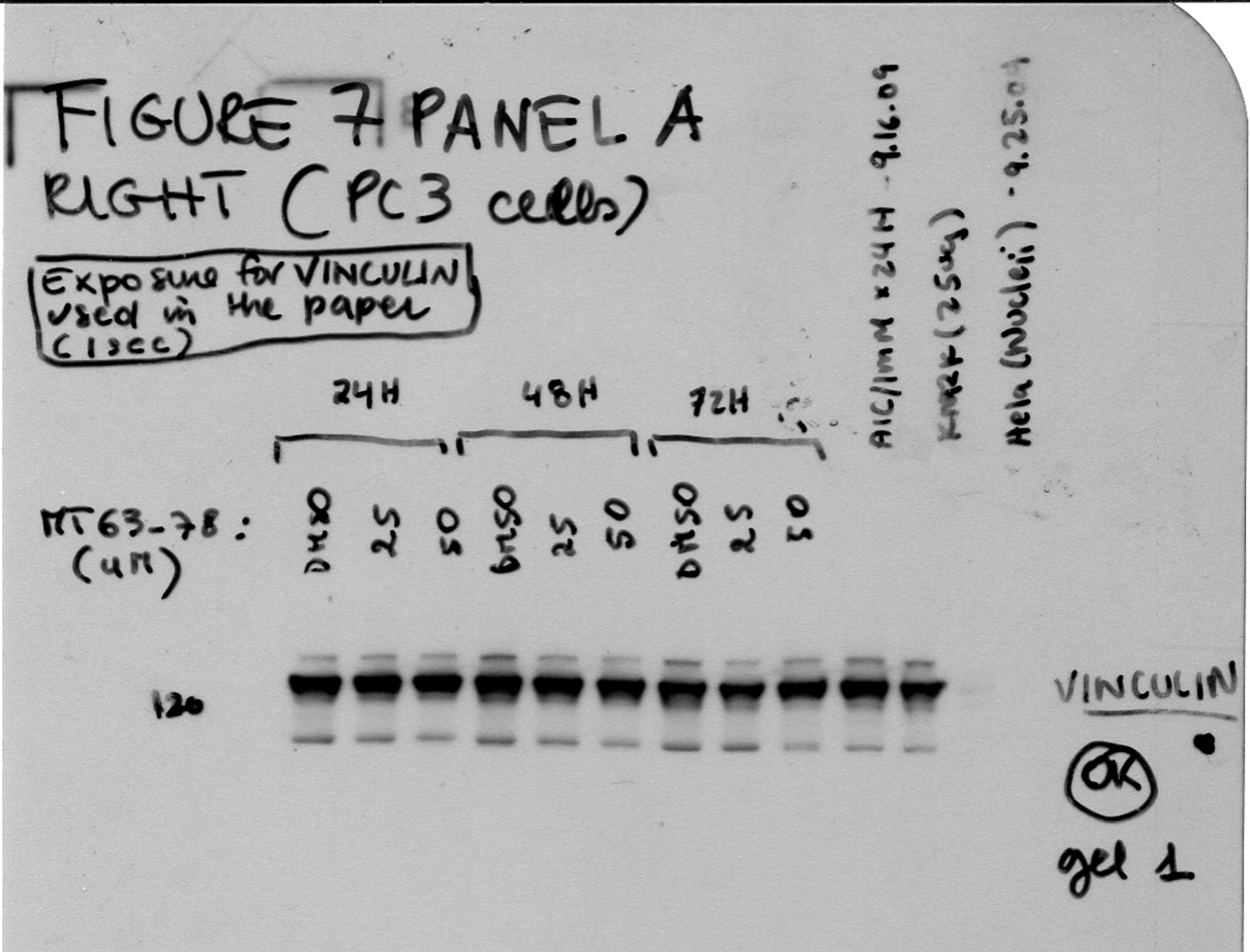
FIGURE 7 PANEL A
RIGHT (PC3 cells)
Exposure for VINCULIN used in the paper (1sec)
24H
48H
72H
MT63-78 : (uM)
DMSO
25
50
DMSO
25
50
DMSO
25
50
AIC/1mM x 24H - 9.16.09
Hela (Nuclei) - 9.25.09
120
VINCULIN
OK
gel 1

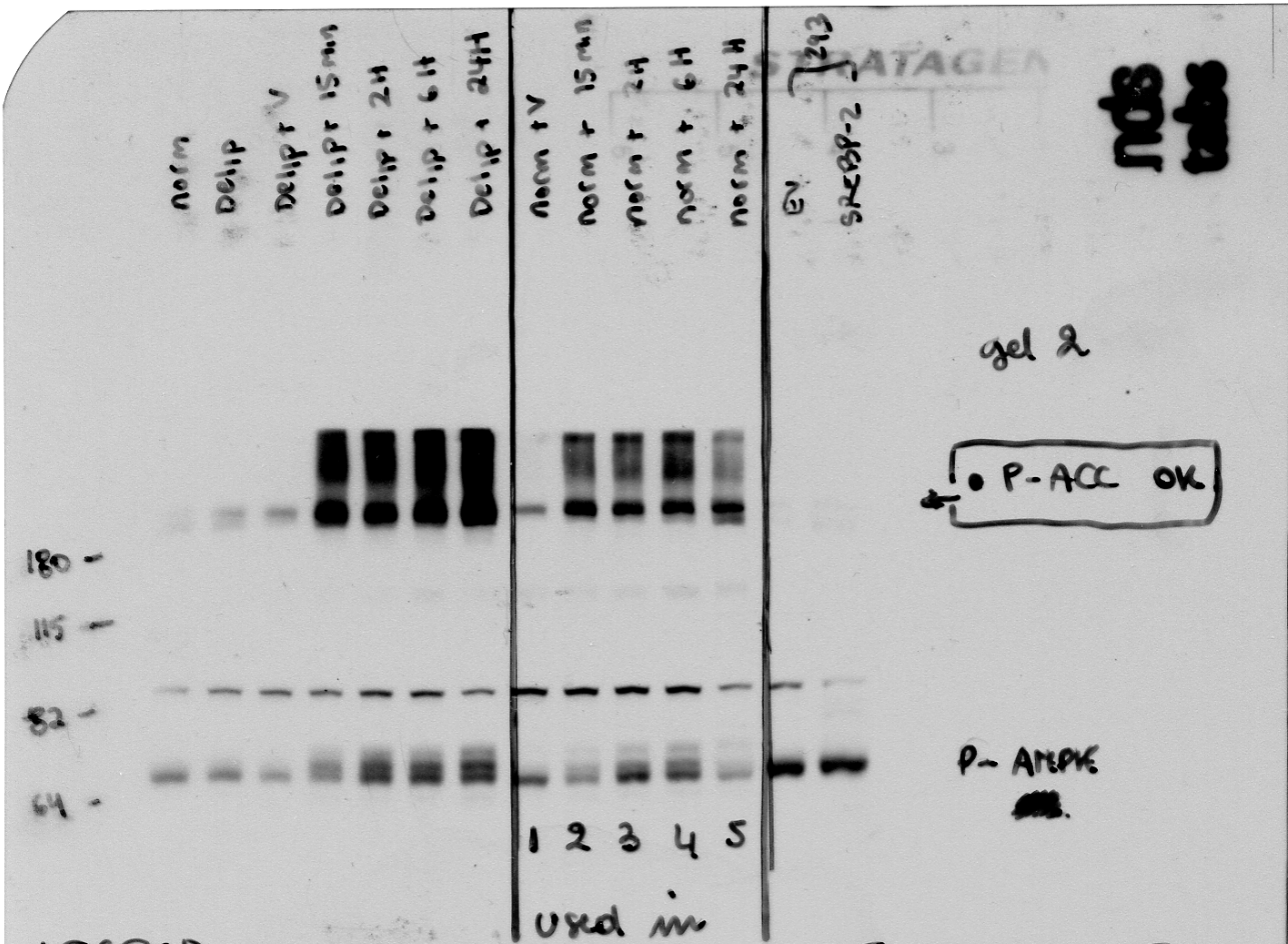

FIGURE 7
PANEL B

LEGEND

1. = complete + Full medium RPMI + dmso

2 = // + MT63-78 30 min

3 = // // 2 hr

4 = // // 6 hr

5 = // // 24 hr

Exposure used for P-ACC in the paper (1sec

samples were prepared in duplicate and loaded on 2 gels

FIGURE 7 PANEL B

(1)

Exposure for
• FASN
• β-actin used in the paper (1 sec)

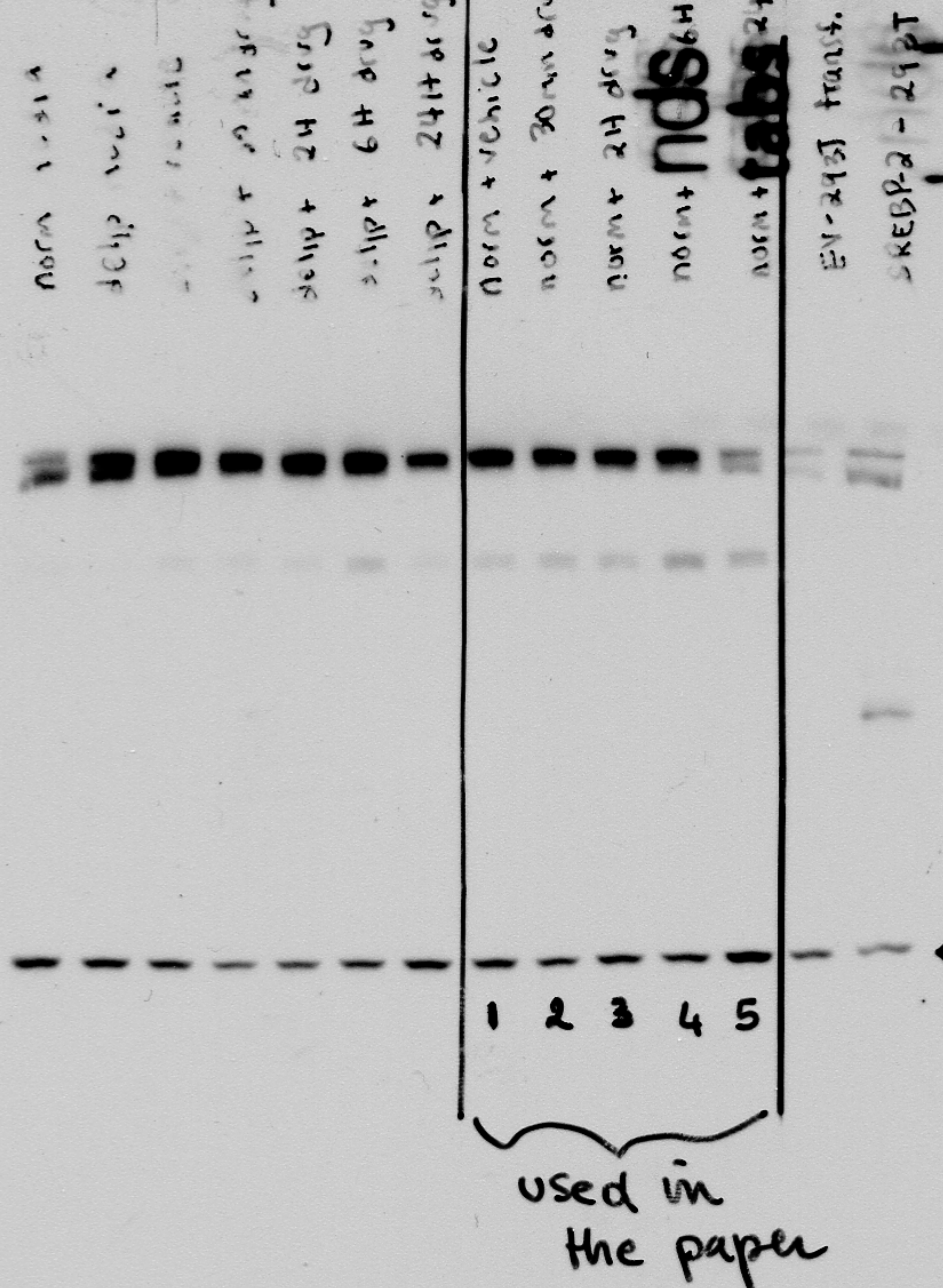

LNCaP cells

gel 1

← • FASN (OK)

← • β-actin (OK)

LEGEND:

1 = complete RPMI + dmso
2 = " " + MT63-78 30min
3 = " + MT63-78 2hr
4 = " " + MT63-78 6hr
5 = complete RPMI + MT63-78 24hr

Samples were prepared in duplicate and loaded on 2 gels

FIGURE 7 PANEL B

Exposure for SREBP-1 precursor

Used in the paper (10 sec)

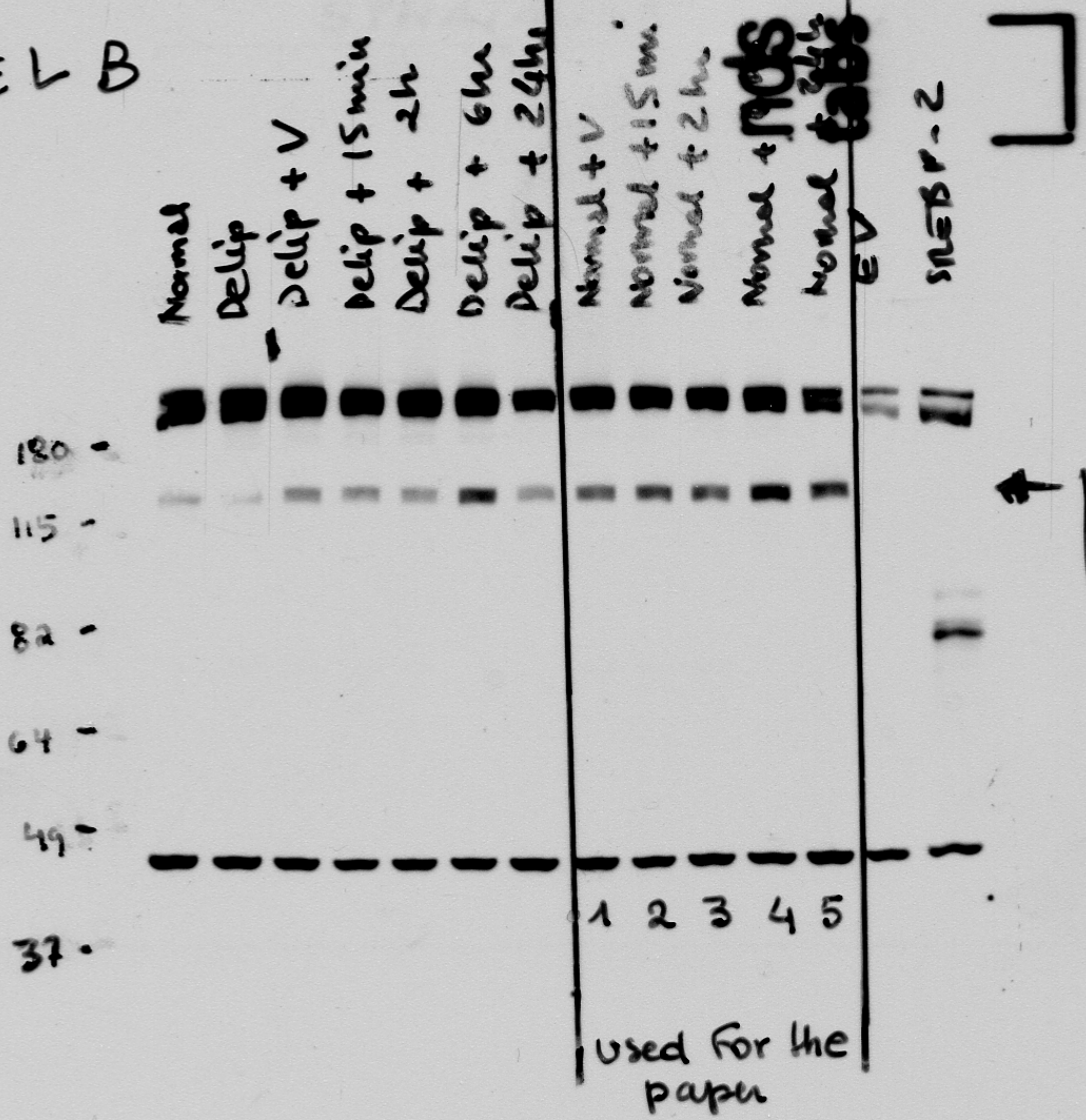

FASN

• precursor (OK) (~125 KD)

SREBP-1

mature (~60-70 KD)

β-actin

LNCaP cells gel 1

LEGEND.

1 = complete RPMI + dmso

2 = // + HT63-48 30 m

3 = // // 2 hr

4 = // // 6 hr

5 = // // 24 hr

samples were prepared in duplicate and loaded on 2 gels

FIGURE 7 PANEL B

Exposure for
mature SREBP-1
used in the
paper (1 min)

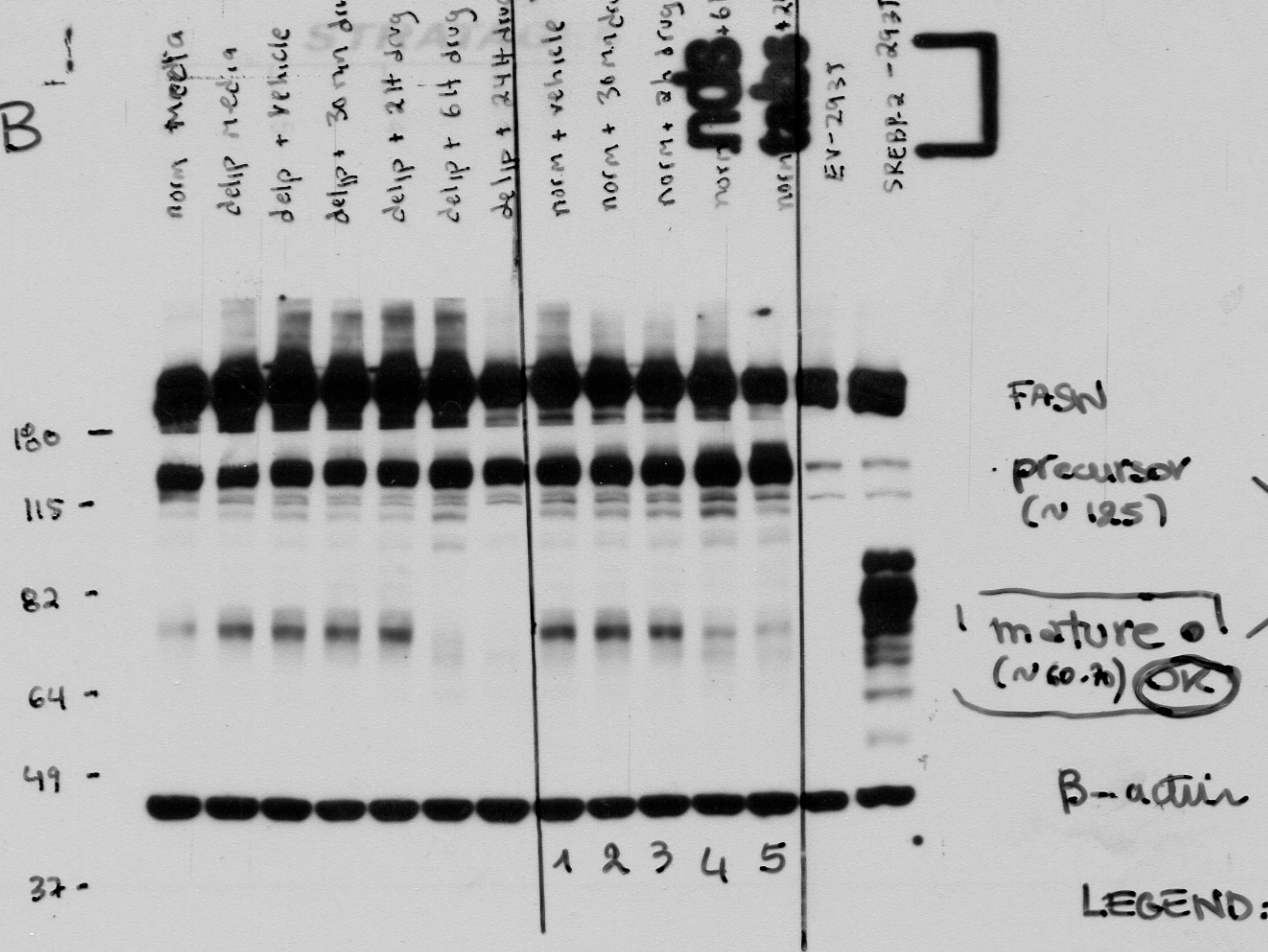

gel 1

samples were prepared in duplicate and loaded on 2 gels

used for the paper

LNCaP cells

LEGEND: ↳ full medium

1. = complete RPMI + dmso

2 = complete RPMI + HT63-78 30min

3 = // // 2h

4 = // // 6h

5 = // // 24h

gel 1 PART 1 — 72h — gel 1 PART 2

DMSO | 63-78/125nM | Rapa/1nM | Rapa/100nM | DMSO | 63-78/125nM | Rapa/1nM | Rapa/100nM

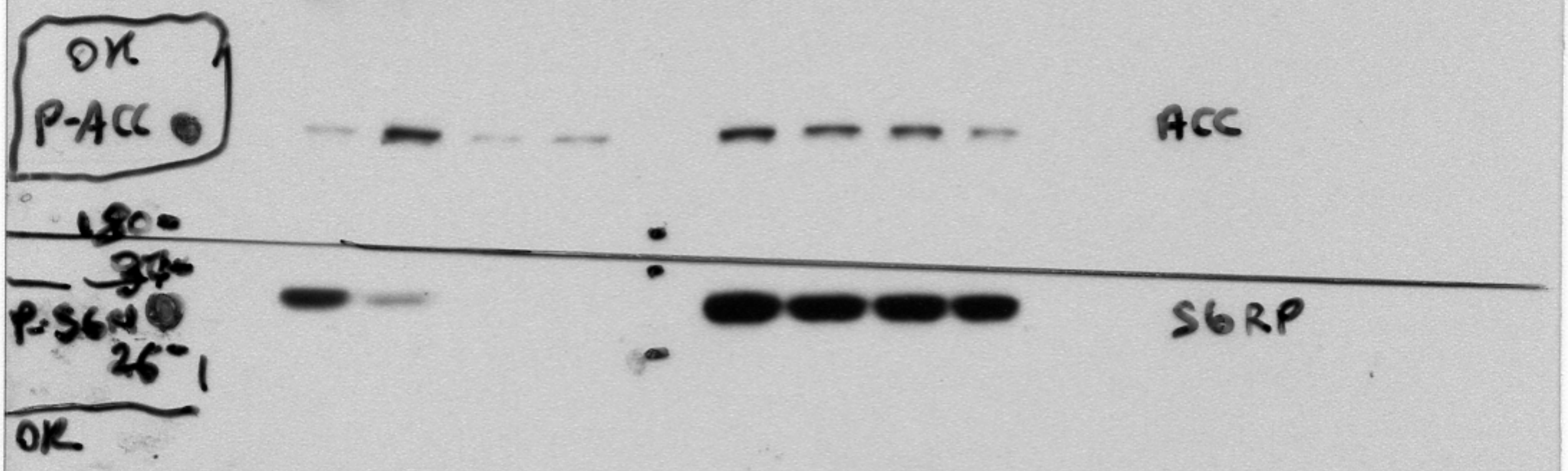

FIGURE 7 PANEL D- LEFT
(LNCaP cells)

Exposure for
P-ACC and P-S6RP
used in the paper

Samples were prepared in duplicate and loaded on 1 gel (gel 1)

2.5min
ECL
8.12.10

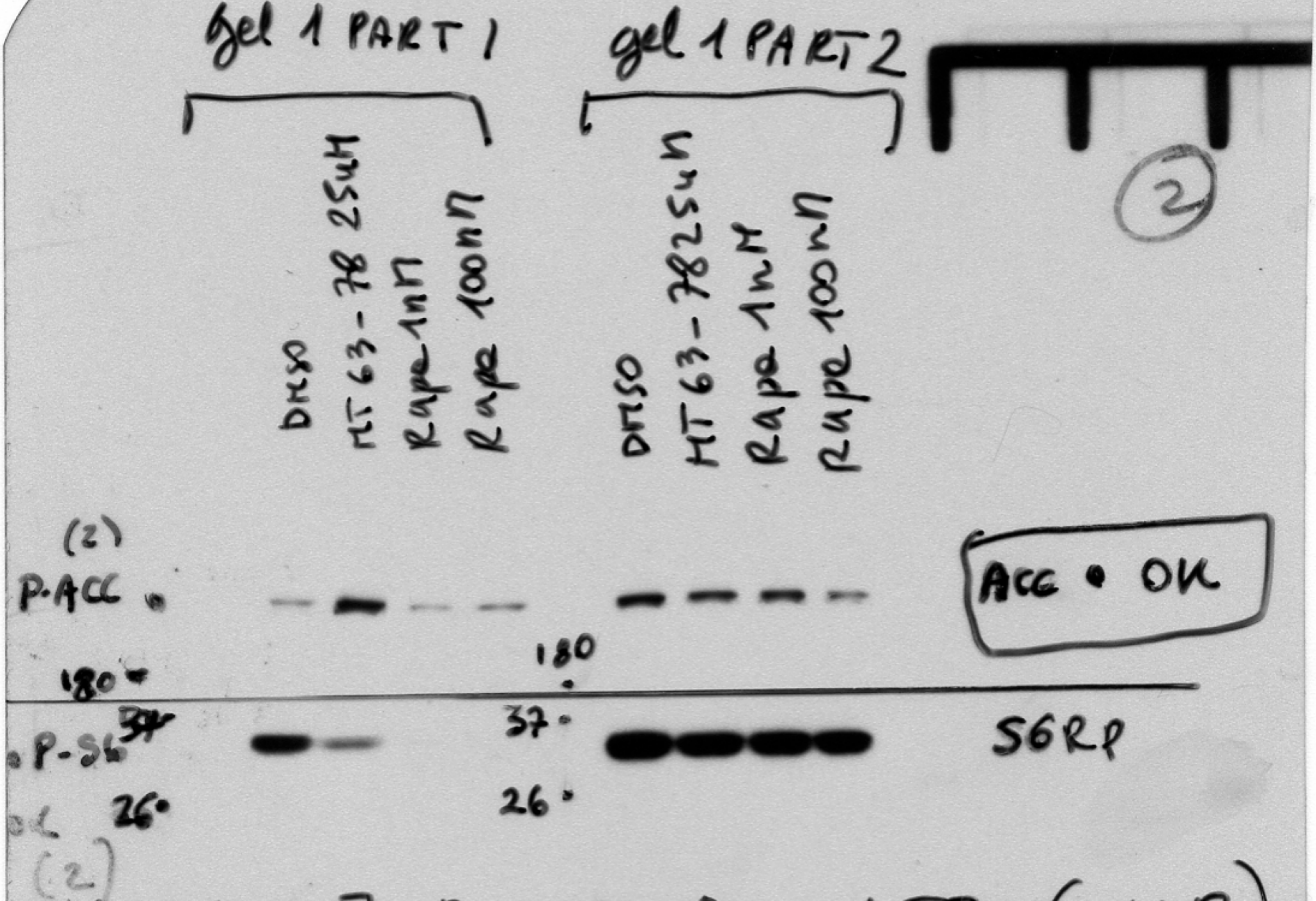

FIGURE 7 PANEL D - LEFT (LNCaP cells)

Exposure for ACC total used in the paper (5min)

Samples were prepared in duplicate and loaded on 1 gel (gel 1

ECL
5min
8.12.10

FIGURE 7 PANEL D
LEFT (LNCaP cells)

Exposure for p-Raptor and Raptor used in the paper (25 min)

72 hrs

DMSO | 63-781 25uM | Rapa 1nM | Rapa 100nM

gel 1
PART 1

180 —
115 —

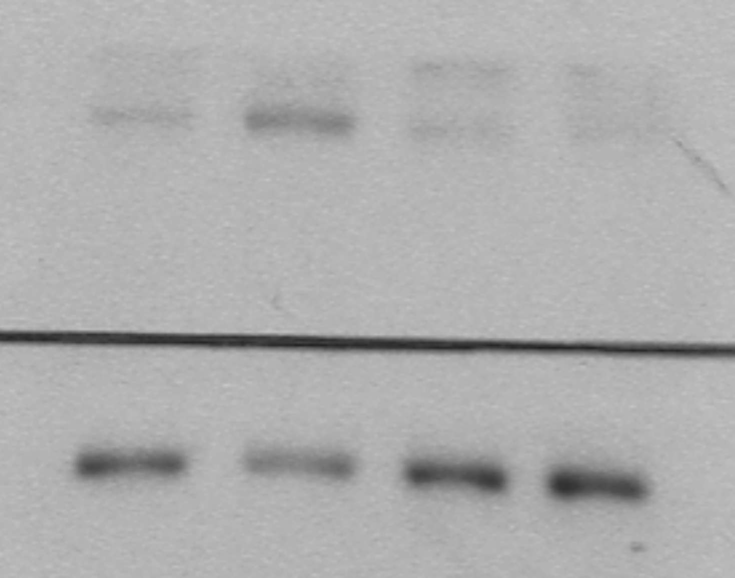

p-Raptor • OK

gel 1
PART 2

180 —
115 —

Raptor • OK

Samples were prepared in duplicate and loaded on 1 gel (gel 1)

25min
ECL+
8.12.10

LNCaP

4

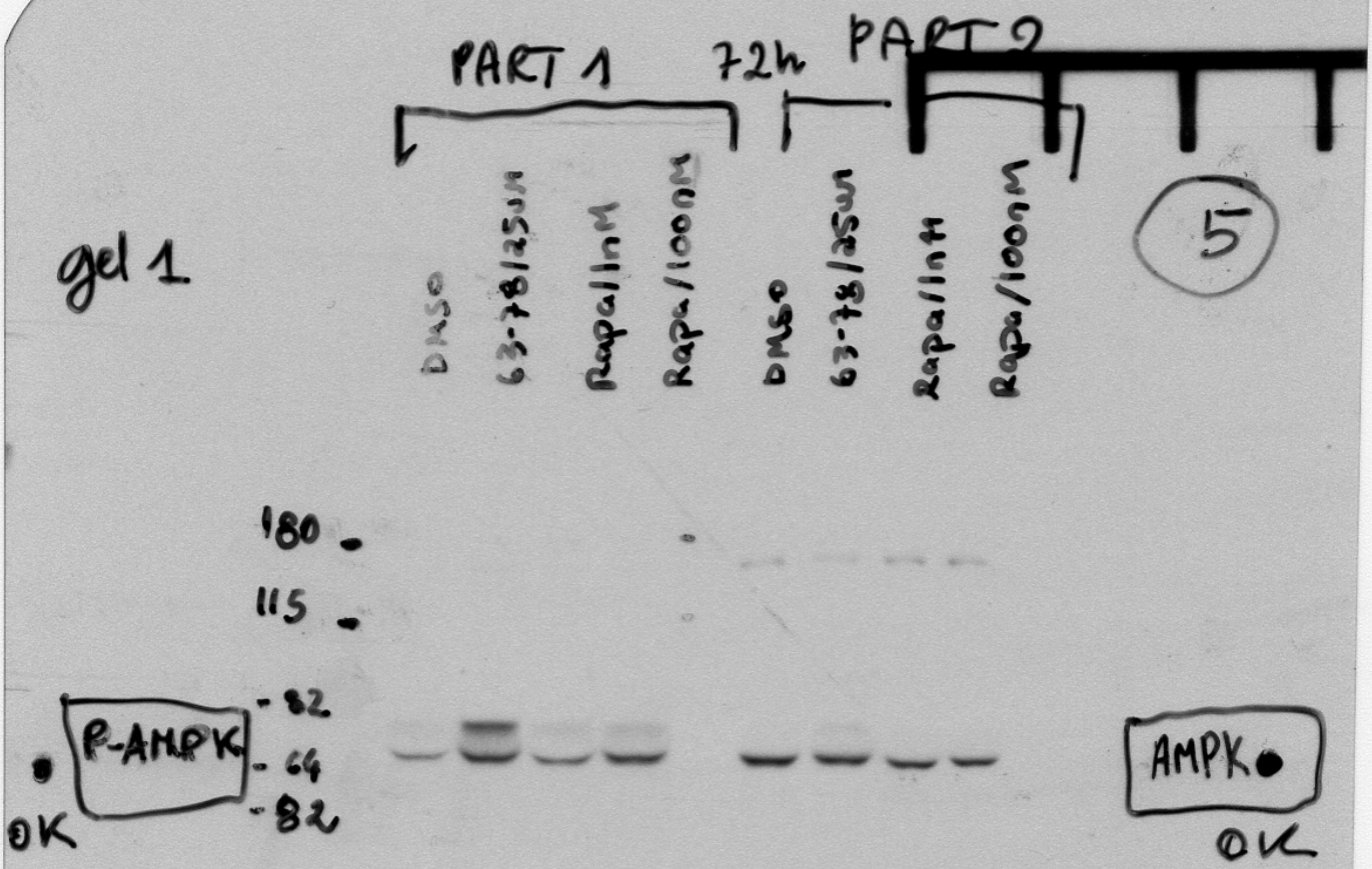

FIGURE 7 PANEL D - RIGHT
(LNCaP cells)

Exposure for
P-AMPK and
AMPK used in the
paper

Samples were prepared in
duplicate and loaded on
1 gel

ECL
8.12.10

3

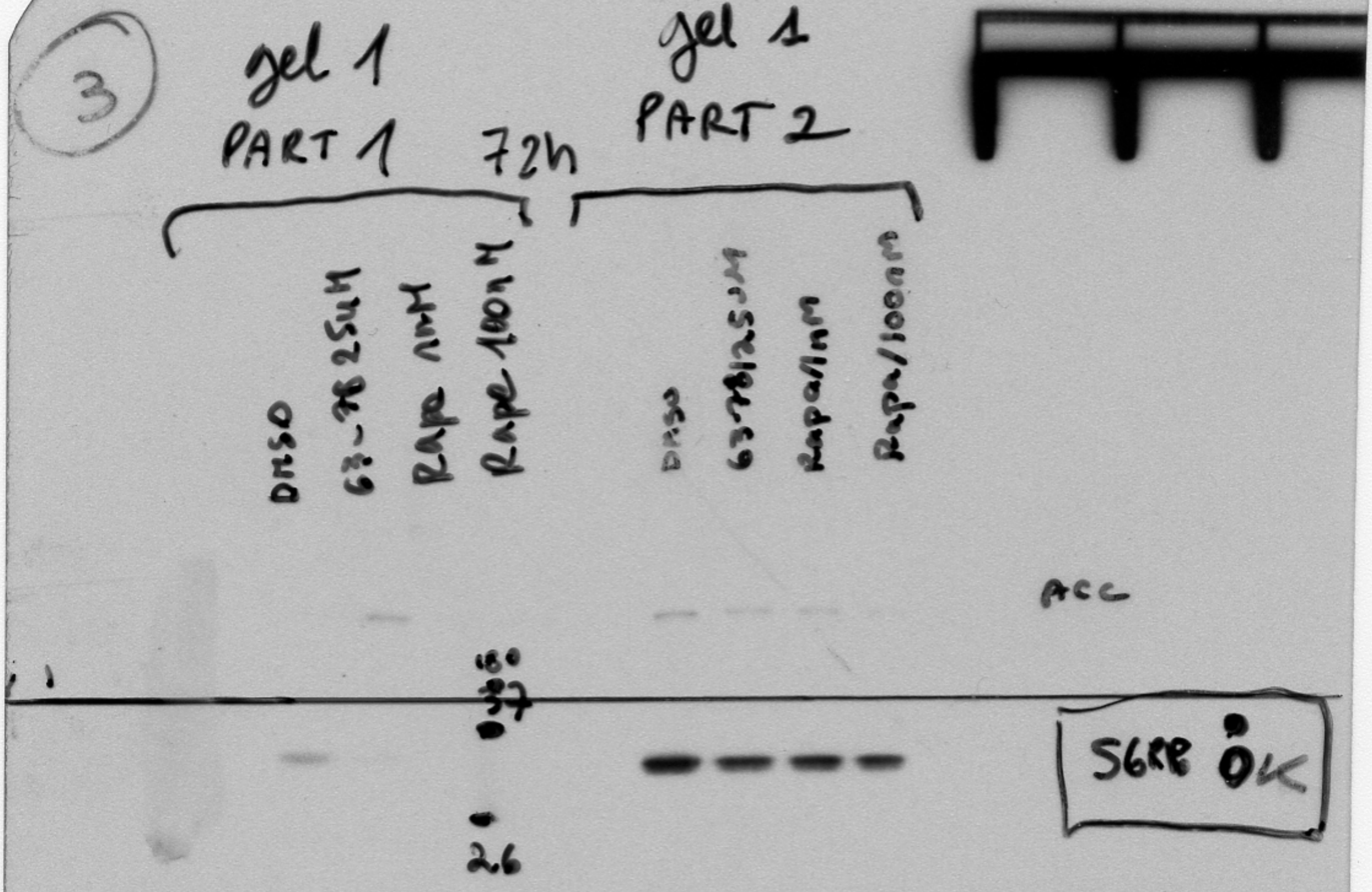

FIGURE 7 PANEL D - LEFT (LNCaP cells)

Exposure for S6RP used in the paper (5sec)

ECL 5sec
8.12.10

Samples were prepared in duplicate and loaded on 1 gel (gel 1)

6

72hr

DMSO
63-78/25uM
Rapa/1nM
Rapa/100nM

Re-blot

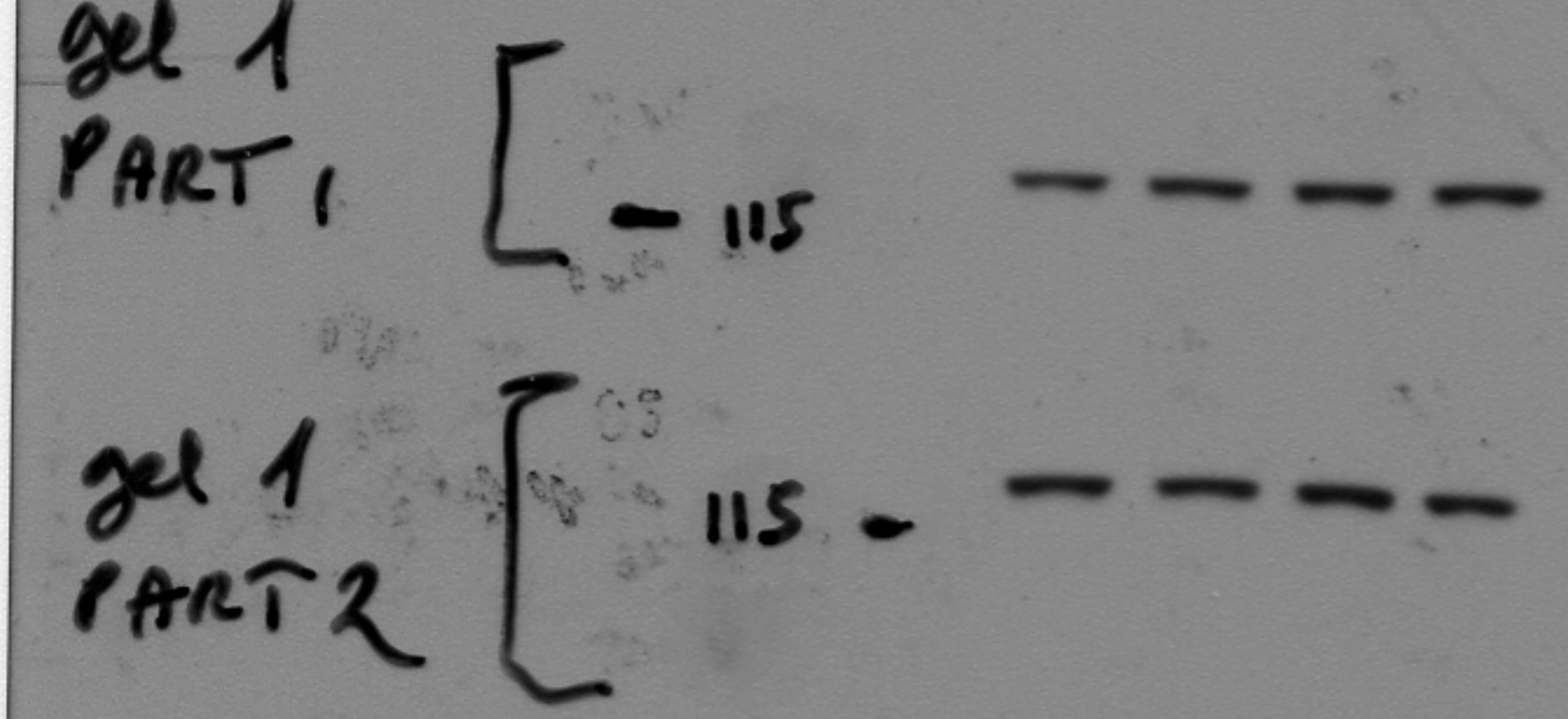

• VINCULIN on P-Raptor memb (PART1

VINCULIN on RAPTOR membrane (PART 2)

FIGURE 7 PANEL D - LEFT
(LNCaP cells)

Exposure for VINCULIN used in the paper

Samples were prepared in duplicate and loaded on 1 gel (gel 1)

LNCaP

ECL
8.13.10

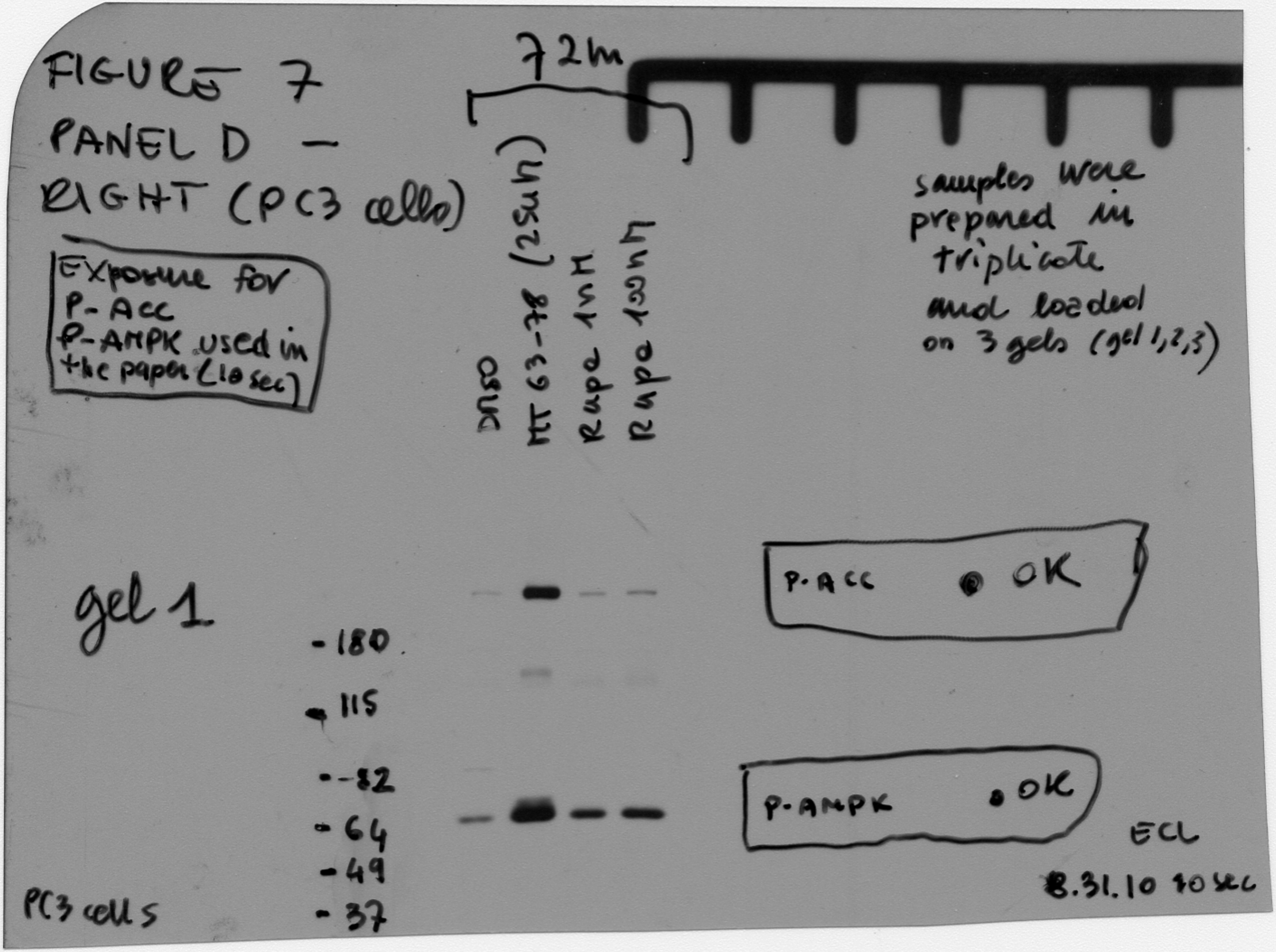

FIGURE 7
PANEL D –
RIGHT (PC3 cells)
Exposure for
P-ACC
P-AMPK used in
the paper (10 sec)
72h
DMSO
MT 63-78 (25uM)
Rapa 1nM
Rapa 100nM
samples were
prepared in
triplicate
and loaded
on 3 gels (gel 1,2,3)
gel 1
- 180
- 115
- 82
- 64
- 49
- 37
PC3 cells
P-ACC • OK
P-AMPK • OK
ECL
8.31.10 10 sec

TT.

FIGURE 7
PANEL D-RIGHT
(PC3 cells)

samples were
prepared in
triplicate and
loaded on
3 gels

Exposure for
ACC and
Raptor used
in the paper
(5 MIN)

gel 2

72h

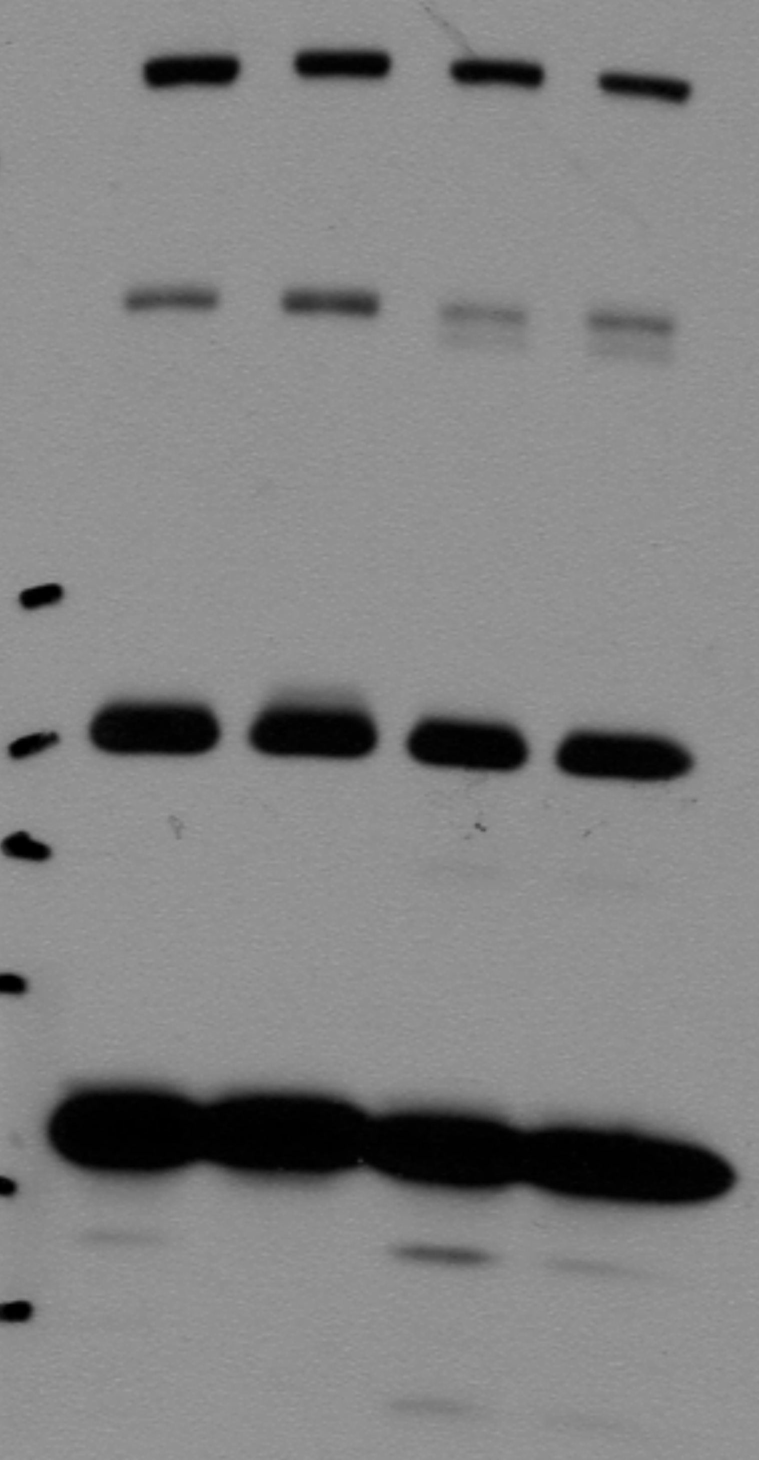

5min
ECL
8.31.0

Samples were prepared in triplicate and loaded on 3 gels (gel 1, 2, 3)

FIGURE 7
PANEL D - RIGHT
(PC3 cells)

Exposure for p-Raptor used in the paper (5 min)

gel 1

72hr

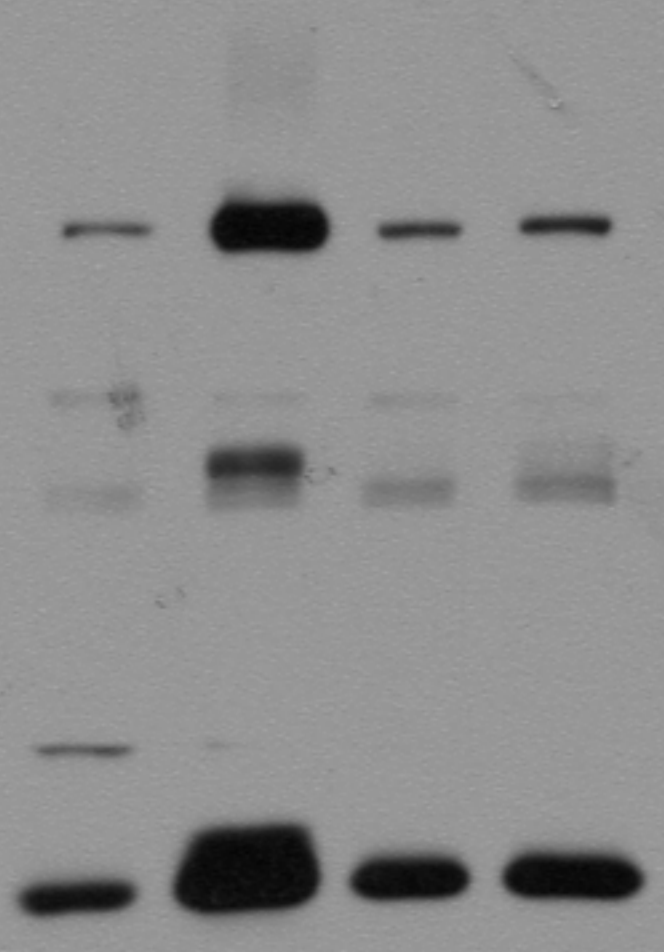

ECL
5 min

8.31.10

-25 ug.

PC3

FIGURE 7
PANEL D – RIGHT (PC3 cells)

Exposure for AMPK used in the paper (1 min)

72 hrs

DMSO
KT 63-78 / 25uM
Rapa 1nM
Rapa 100nM

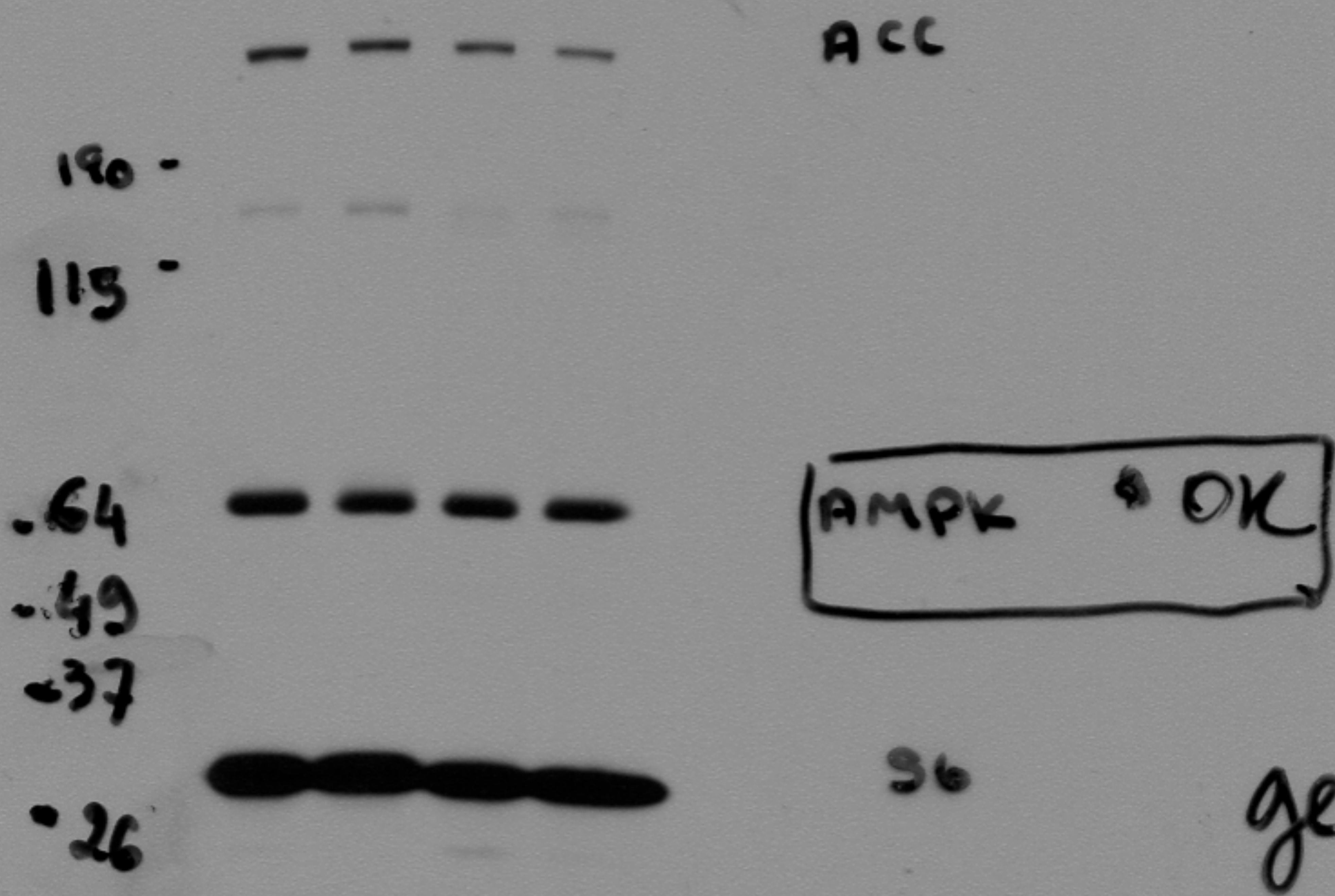

gel 2

Samples were prepared in triplicate and loaded on 3 gels (gel 1, 2, 3)

PC3

1 min ECL 8.31.10

Samples were prepared in triplicate and loaded on 3 gels (gel 1,2,3)

FIGURE 7
PANEL D - RIGHT
(PC3 cells)

72h

DMSO
KT 63-76 (25uM)
Rapa 1uM
Rapa 100nM

Exposure for S6 used in the paper

gel 2 72h

64
49
-37
-26

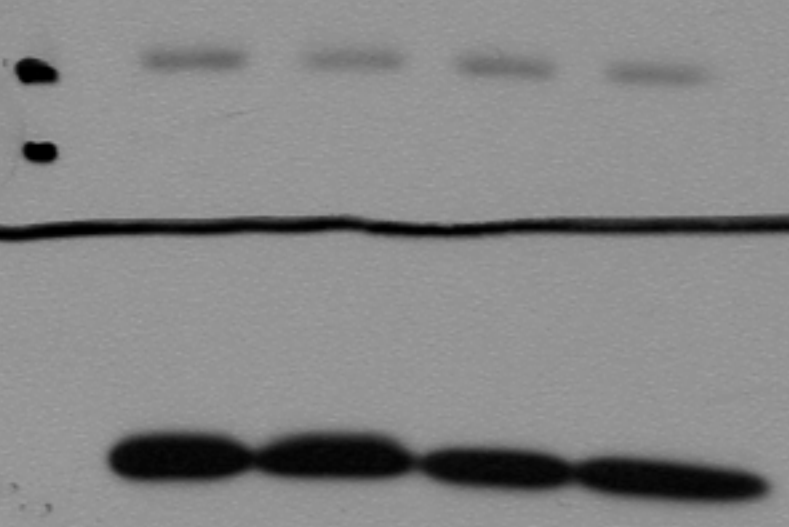

AMPK

S6 RP OK

PC3 cells

10 sec
ECL
8.31.10

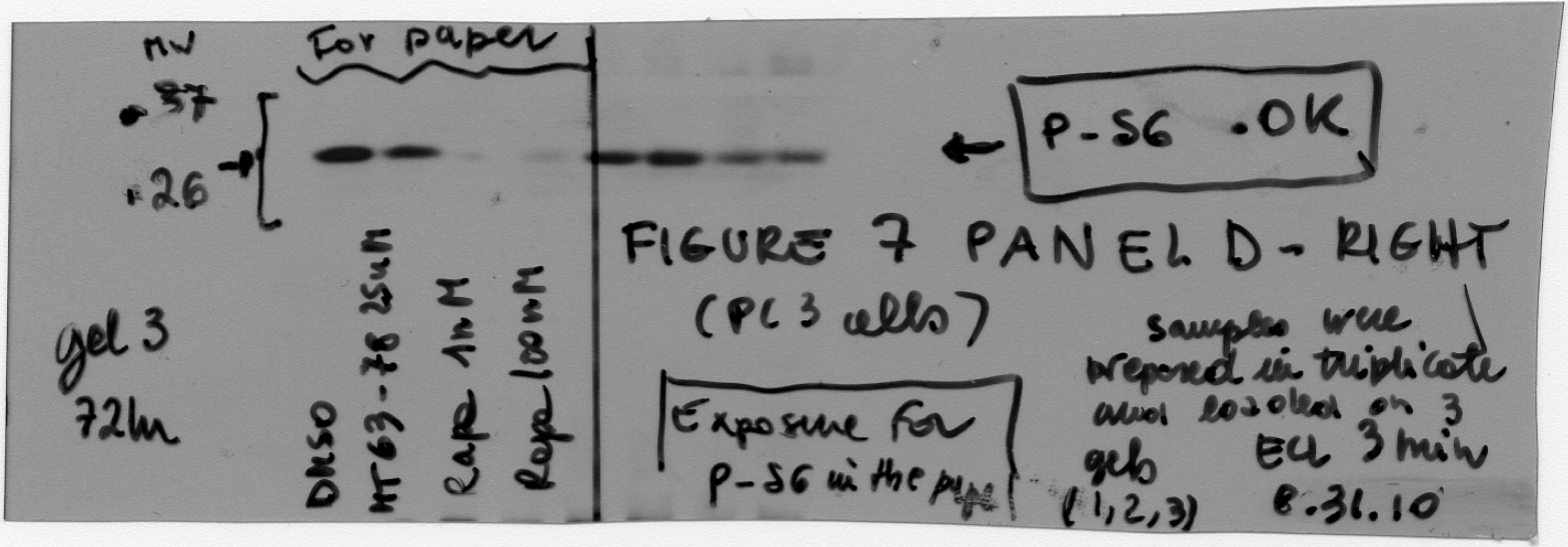
MW
For paper
37
26
P-S6 .OK
FIGURE 7 PANEL D - RIGHT
(PC3 cells)
gel 3
72h
DMSO
MT63-78 25uM
Rapa 1nM
Rapa 100nM
Exposure for
P-S6 in the p[illegible]
samples were
prepared in triplicate
and loaded on 3
gels
(1,2,3)
ECL 3 min
8.31.10

FIGURE 7
PANEL D - RIGHT
(PC3 cells)

PC3x72H: Rapa Exp

- 25ug
- 8.27.2010 samples

Exposure for VINCULIN used in the paper

DMSO | 63-T812.5uM | Rapa/1nM | Rapa/100nM

gel 1

72h

-115

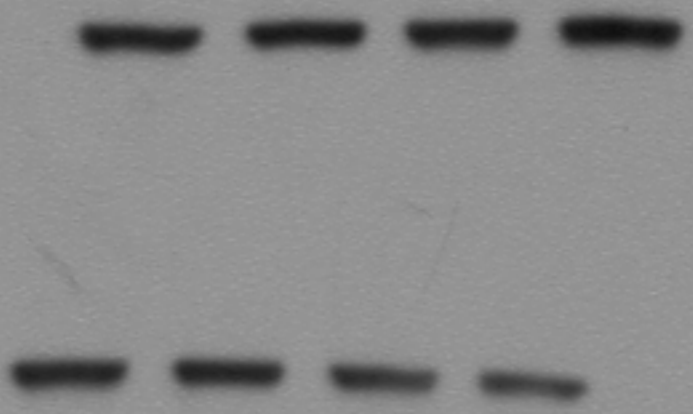

gel 2

-115

REBLOT

VINCULIN on p-Raptor membrane OK

VINCULIN on Raptor membrane

ECL
9.1.2010
