# Supplementary material for: A novel direct activator of AMPK inhibits prostate cancer growth by blocking lipogenesis
Source: EMBO Mol Med. 2014 Feb 4;6(4):519–38. doi: 10.1002/emmm.201302734 (PMC3992078; doi:10.1002/emmm.201302734)
Supplement: Supplementary file 8 [file emmm0006-0519-sd8.pdf]

FIGURE 10, PANEL B

Exposure for
- P-ACC
- P-AMPK
used in the paper
(5 min)
2.9. 2011

Sept/October xenograft
2010 experim.

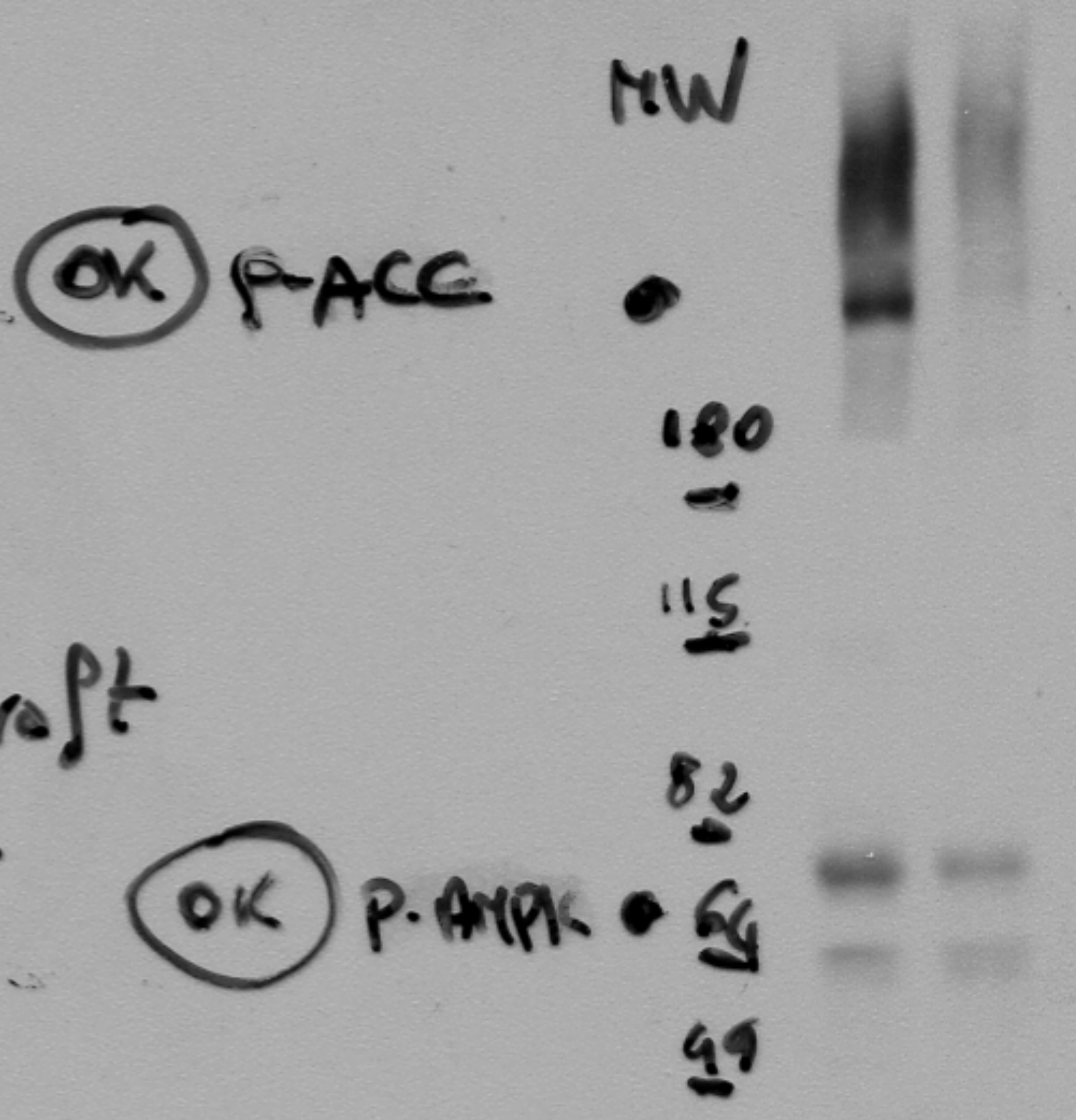

FIGURE 10
PANEL B

Exposure for
P- Raptor used in the
paper (5 min)
2.9.2011

970 - treatment (MT63-78)
964 - Control

MW
180 -

(OK) P-Raptor •

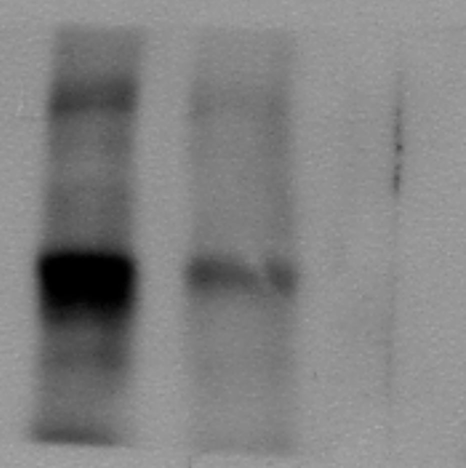

Sept/Oct xenograft
2010 experiment
ECL plus 5 min

2.9.2011

FIGURE 10, PANEL B

Exposure for P-S6 used in the paper (10 sec) 2.9.2011

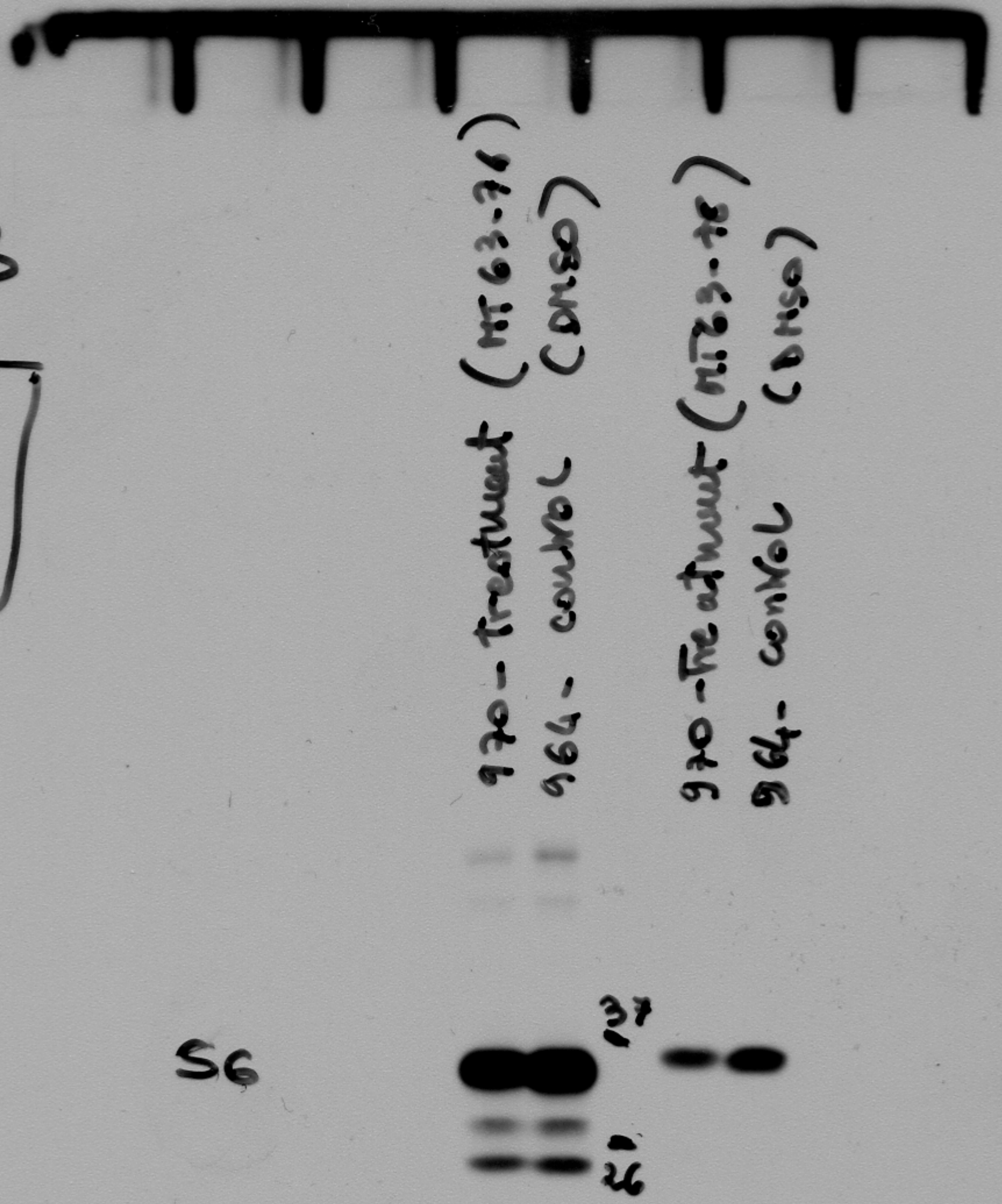

← (P) S6 . OK

ECL 10 sec
2. 9. 2011

(MT63-78)

970 - treatment

964 - control

OK

• VINCULIN → 115

(Re-blot)

180

82

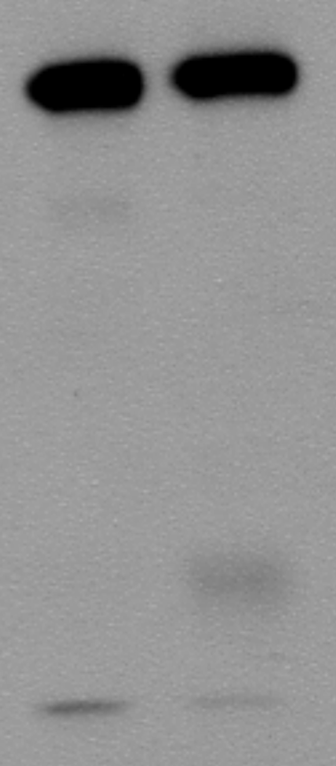

FIGURE 10, PANEL B

Exposure for VINCULIN used in the paper (1 sec)

2.10.11

ECL quick

2.10.2011
