# Supplementary material for: A novel direct activator of AMPK inhibits prostate cancer growth by blocking lipogenesis
Source: EMBO Mol Med. 2014 Feb 4;6(4):519–38. doi: 10.1002/emmm.201302734 (PMC3992078; doi:10.1002/emmm.201302734)
Supplement: Supplementary file 9 [file emmm0006-0519-sd9.pdf]

**A**

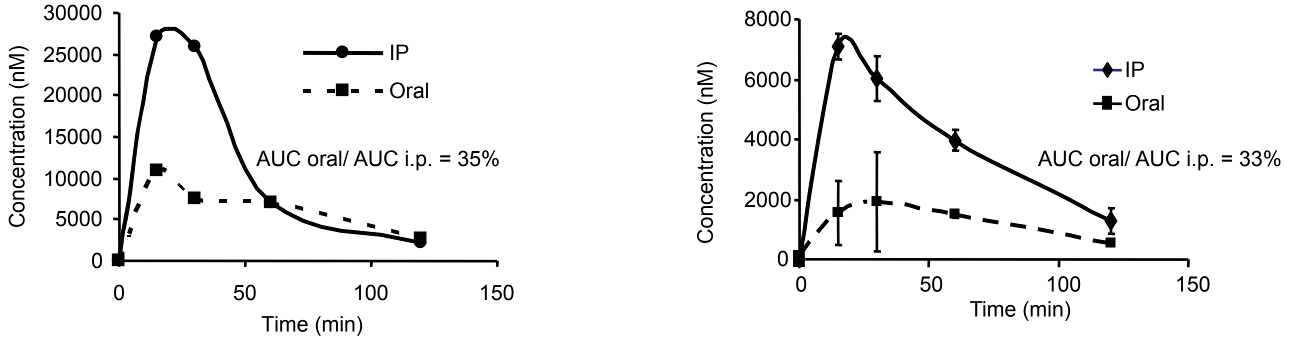


**B**

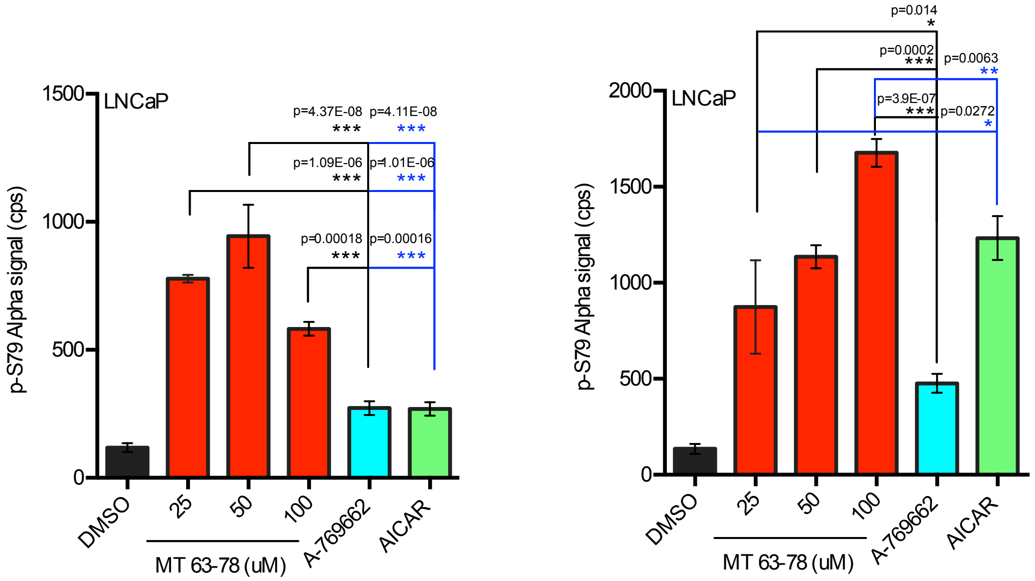


**Supporting Information Fig 1. Pharmacokinetic and potency characterization of MT 63-78.**

**A.** Pharmacokinetic of MT 63-78. MT 63-78 was administered in three mice i.p. or orally using Mylanta as vehicle. In the left panel, 10 mg/kg of MT 63-78 were administered i.p. or orally in C57BL/6 mice. Each data point represents the pooled plasma collected from the three mice at the different time points and analyzed by LC/MS/MS. In the right panel, 2 mg/kg of MT 63-78 were administered i.p. or orally in C57BL/6 mice. Plasma was collected at each time point and analyzed separately. Results are expressed as mean ±SD. Oral bioavailability was calculated as the ratio AUC (oral dose)/AUC (i.p. dose) as described in Supporting Materials and Methods.

**B.** AMPK activity in LNCaP and PC3 cells measured with Alpha Screen Assay, as described in Supporting Materials and Methods. Assay was performed in cell lysates following 30-min treatment with MT 63-78, A-769662 (100uM), and AICAR (2mM). Results are expressed as means ±SD of three independent samples. One-way ANOVA test, followed by Tukey's post hoc test for multiple comparisons was performed and significant p values are reported on the bar graphs. Cps= count per second.
