# Supplementary material for: A novel direct activator of AMPK inhibits prostate cancer growth by blocking lipogenesis
Source: EMBO Mol Med. 2014 Feb 4;6(4):519–38. doi: 10.1002/emmm.201302734 (PMC3992078; doi:10.1002/emmm.201302734)
Supplement: Supplementary file 10 [file emmm0006-0519-sd10.pdf]

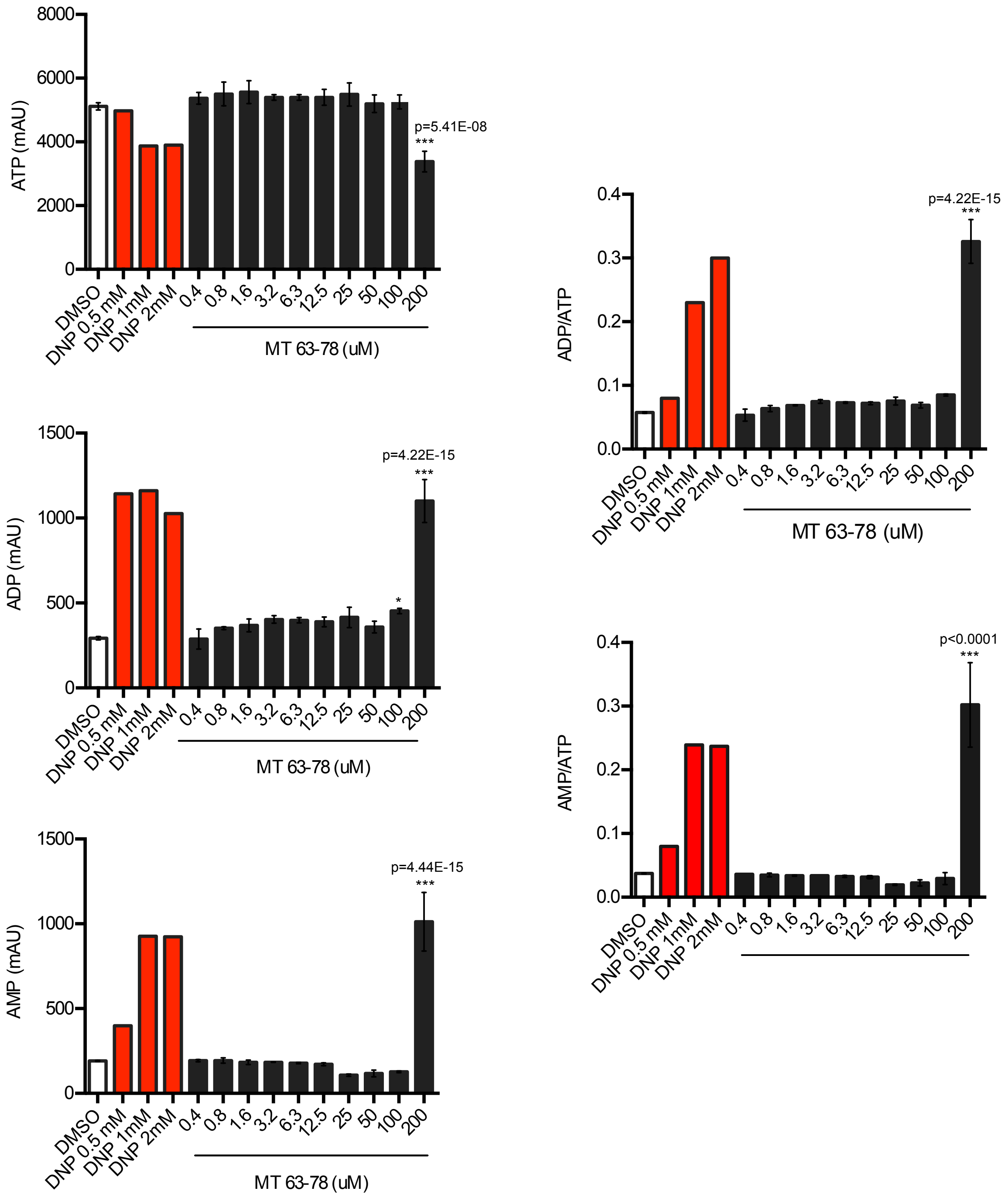

Supporting Information Fig 2. Measurement of intracellular ATP, ADP, and AMP levels in HepG2 cells.

ATP, ADP, and AMP were measured in HepG2 cell lysates, following 30-min treatment with MT 63-78 at the indicated concentrations, using HPLC. AMP/ATP and ADP/ATP were also calculated. Results are expressed as means ±SD of three independent samples. One-way ANOVA test, followed by Dunnett's post hoc test for multiple comparisons was performed and significant p values (treatment *vs* DMSO) are reported on the bar graphs. Single samples treated with different concentrations of the uncoupler Dinitrophenol (DNP) were used as control. mAU= milliabsorbance units.
