# Supplementary material for: A novel direct activator of AMPK inhibits prostate cancer growth by blocking lipogenesis
Source: EMBO Mol Med. 2014 Feb 4;6(4):519–38. doi: 10.1002/emmm.201302734 (PMC3992078; doi:10.1002/emmm.201302734)
Supplement: Supplementary file 11 [file emmm0006-0519-sd11.pdf]

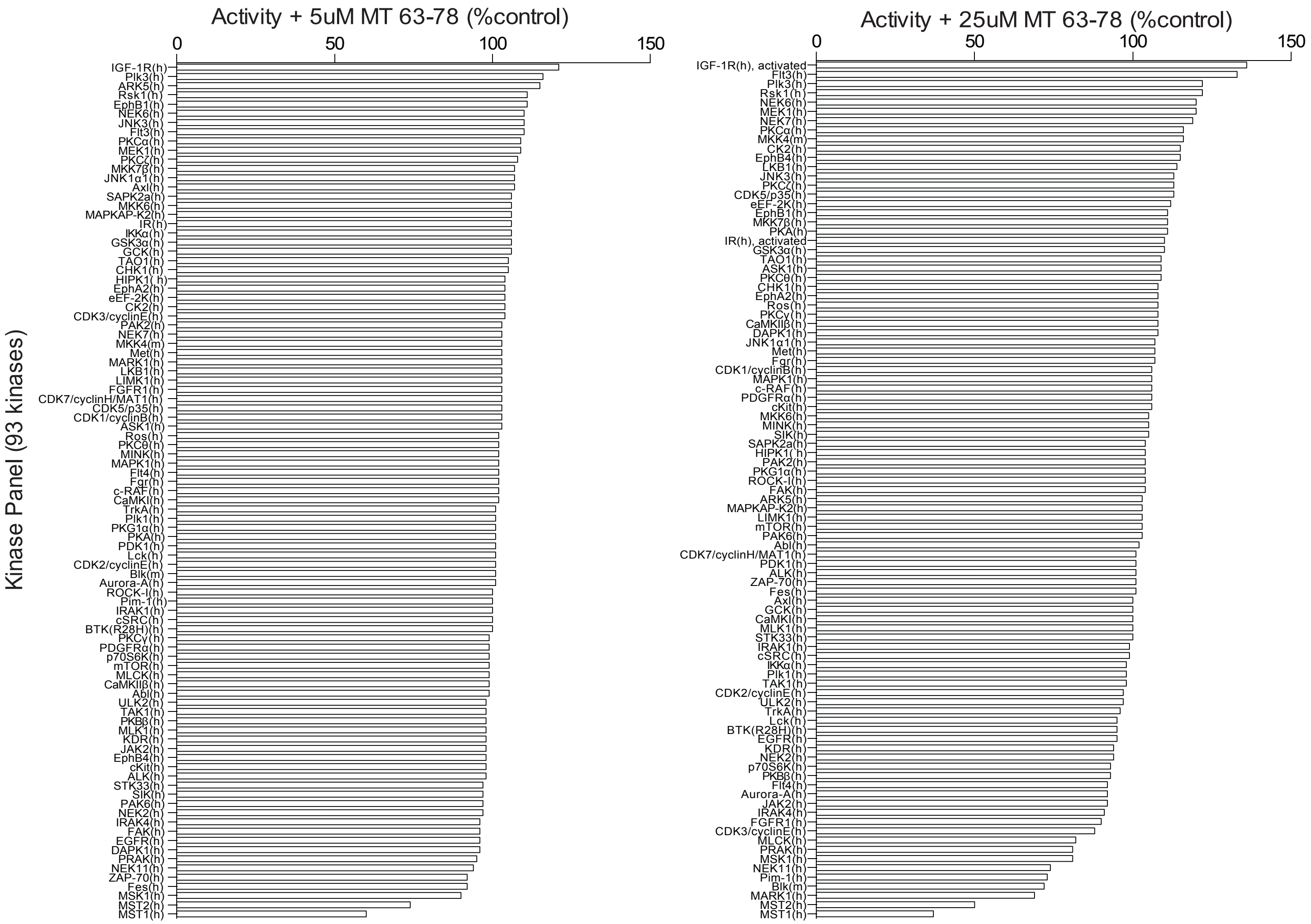

Supporting Information Fig 3. Characterization of MT 63-78 specificity.

Effect of MT 63-78 (5uM, left panel and 25 uM, right panel) on the activity of a panel of 93 protein kinases. Results are shown in rank order as the percentage of activity when compared to a control without compound.
