# Supplementary material for: A novel direct activator of AMPK inhibits prostate cancer growth by blocking lipogenesis
Source: EMBO Mol Med. 2014 Feb 4;6(4):519–38. doi: 10.1002/emmm.201302734 (PMC3992078; doi:10.1002/emmm.201302734)
Supplement: Supplementary file 12 [file emmm0006-0519-sd12.pdf]

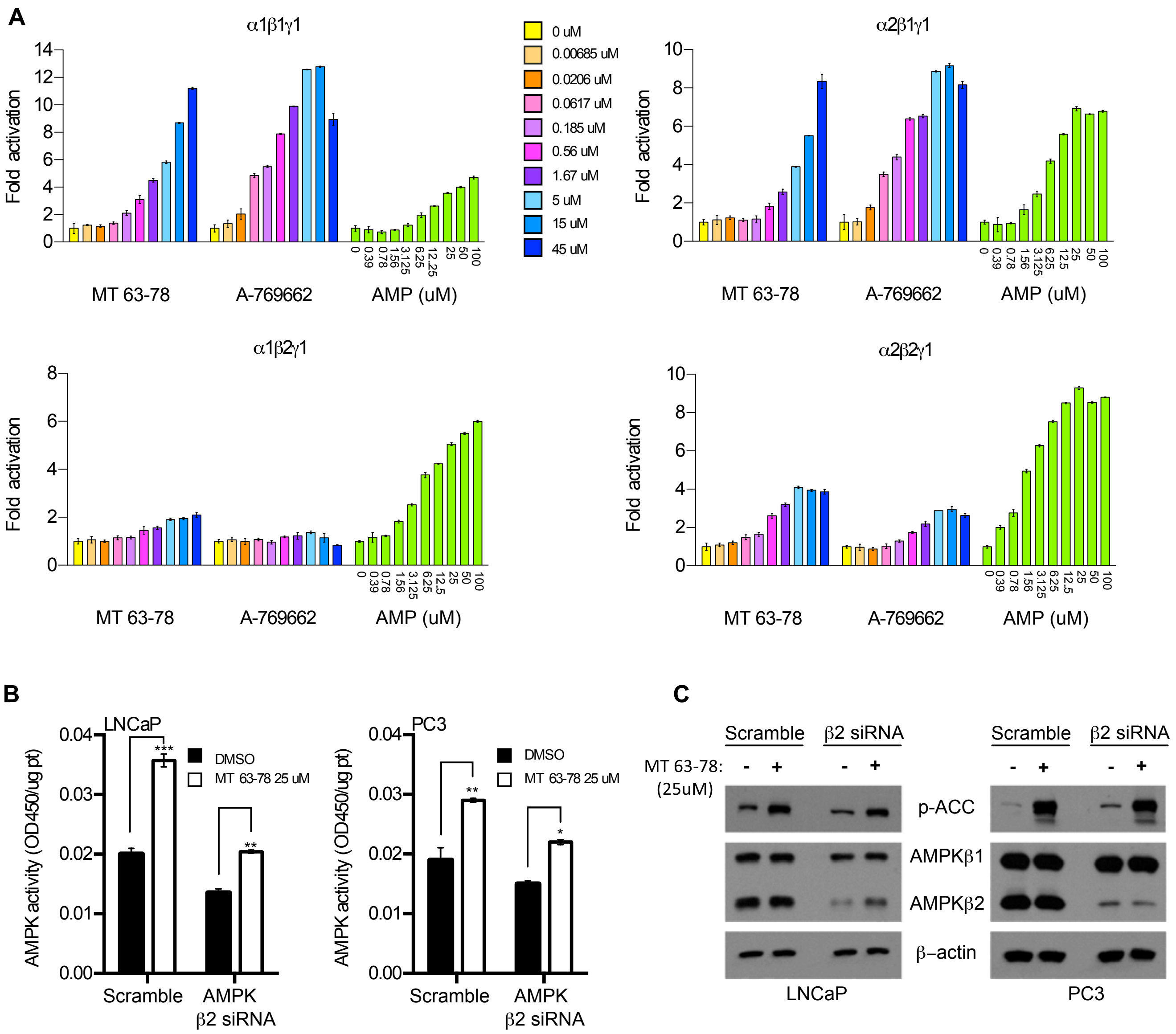

Supporting Information Fig 4. Characterization of MT 63-78 specificity.

A. *In vitro* activity of four different recombinant AMPK heterotrimers (α1β1γ1, α2β1γ1, α1β2γ1, α2β2γ1), following 30-min treatment with MT 63-78, A-769662, and AMP at the indicated concentrations. AMPK activity was measured using alpha screen assay, as as described in Supporting Materials and Methods. Results are expressed as fold activation of basal level (DMSO treatment) set as 1.

B. AMPK activity in LNCaP and PC3 cells transfected with AMPK β2 subunit siRNA, following 30-min treatment with MT 63-78 (MT) or DMSO. AMPK activity was measured with CycLex AMPK Kinase Assay, as described in Supporting Materials and Methods. Results are the mean of two independent samples. One-way ANOVA test, followed by Bonferroni post hoc test for multiple comparisons was performed and adjusted p values were calculated (LNCaP scramble: $^{***}p=0.0002$ MT *vs* DMSO. LNCaP β2siRNA: $^{**}p=0.0058$ MT *vs* DMSO. PC3 scramble: $^{**}p=0.0059$ MT *vs* DMSO. PC3 β2 siRNA: $^{*}p=0.029$ MT *vs* DMSO.

C. Western blotting shows the rate of AMPK β2 silencing and ACC phosphorylation in β2 knockdown cells in the presence or absence of MT 63-78.
