# Supplementary material for: A novel direct activator of AMPK inhibits prostate cancer growth by blocking lipogenesis
Source: EMBO Mol Med. 2014 Feb 4;6(4):519–38. doi: 10.1002/emmm.201302734 (PMC3992078; doi:10.1002/emmm.201302734)
Supplement: Supplementary file 13 [file emmm0006-0519-sd13.pdf]

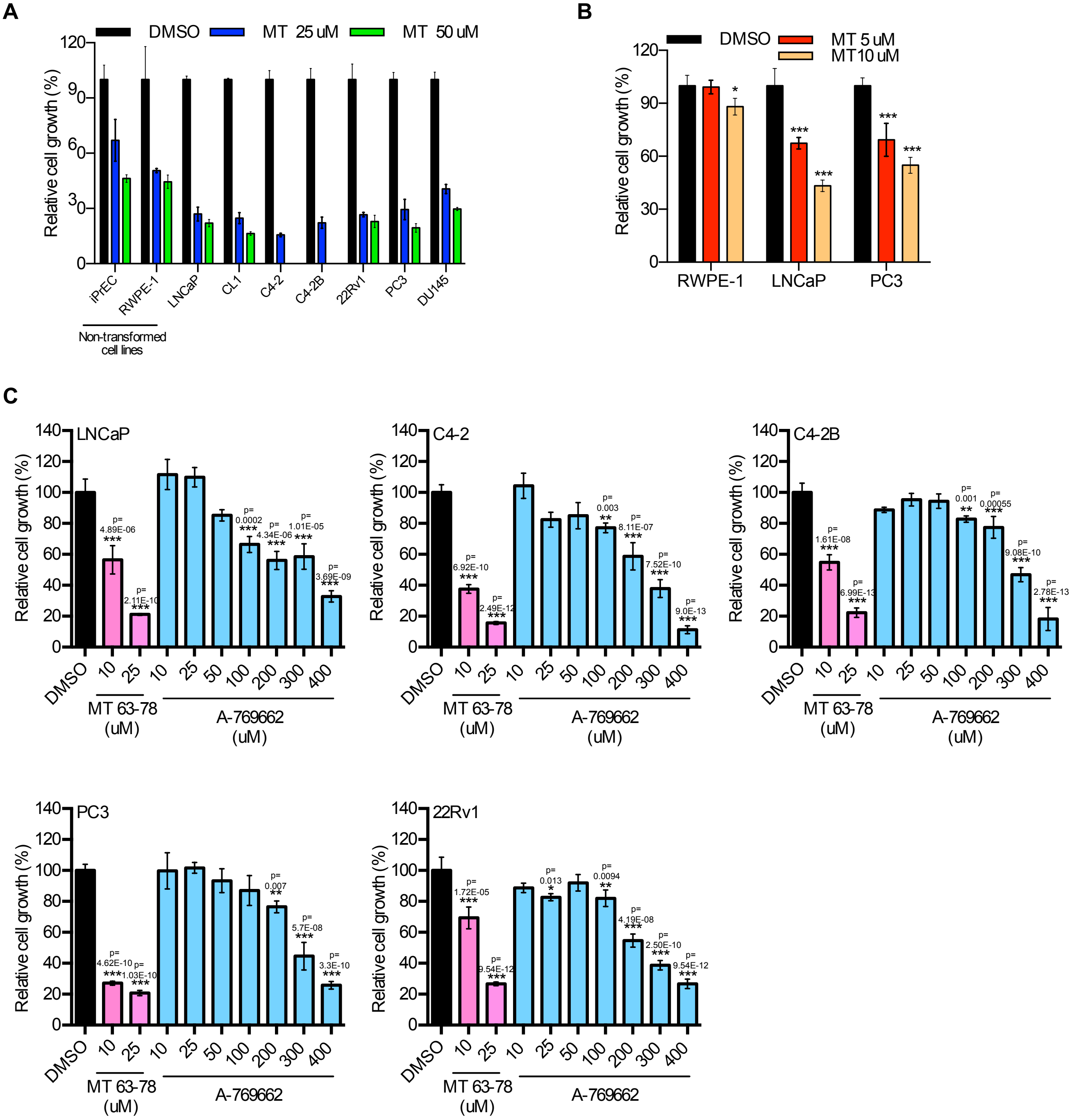


**Supporting Information Fig 5. Cell growth inhibition induced by MT 63-78.**

**A.** Relative growth of non-transformed iPrEC and RWPE-1 cells compared to PCa cells (LNCaP CL1, C4-2, C4-2B, 22Rv1, PC3, DU145), following 72-hr treatment with 25 and 50 uM MT 63-78. Results are expressed as percentage of cells compared to control (DMSO) ±SD of three independent experiments.

**B.** Relative growth of non-transformed RWPE-1 cells compared to PCa cells LNCaP and PC3 cells, following 48-hr treatment with 5 and 10 uM MT 63-78. The results are expressed as percentage of cells compared to control (DMSO) ±SD of three independent samples. One-way ANOVA test, followed by Dunnet's post hoc test for multiple comparisons was performed and adjusted p values were calculated (RWPE-1: *p= 0.0217 MT 25uM vs DMSO; LNCaP: ***p< 0.0001 MT 10uM or MT 25uM *vs* DMSO; PC3: ***p< 0.0001 MT 10uM or MT 25uM *vs* DMSO).

**C.** Relative growth of PCa cells (LNCaP, C4-2, C4-2B, PC3, and 22Rv1) following 72-hr treatment with MT 63-78 or A-769662. Results are expressed as percentage of cells compared to control (DMSO) ±SD of three independent samples. One-way ANOVA test, followed by Dunnett's post hoc test for multiple comparisons was performed. Significant p values are reported on the bar graphs.
