# Supplementary material for: A novel direct activator of AMPK inhibits prostate cancer growth by blocking lipogenesis
Source: EMBO Mol Med. 2014 Feb 4;6(4):519–38. doi: 10.1002/emmm.201302734 (PMC3992078; doi:10.1002/emmm.201302734)
Supplement: Supplementary file 14 [file emmm0006-0519-sd14.pdf]

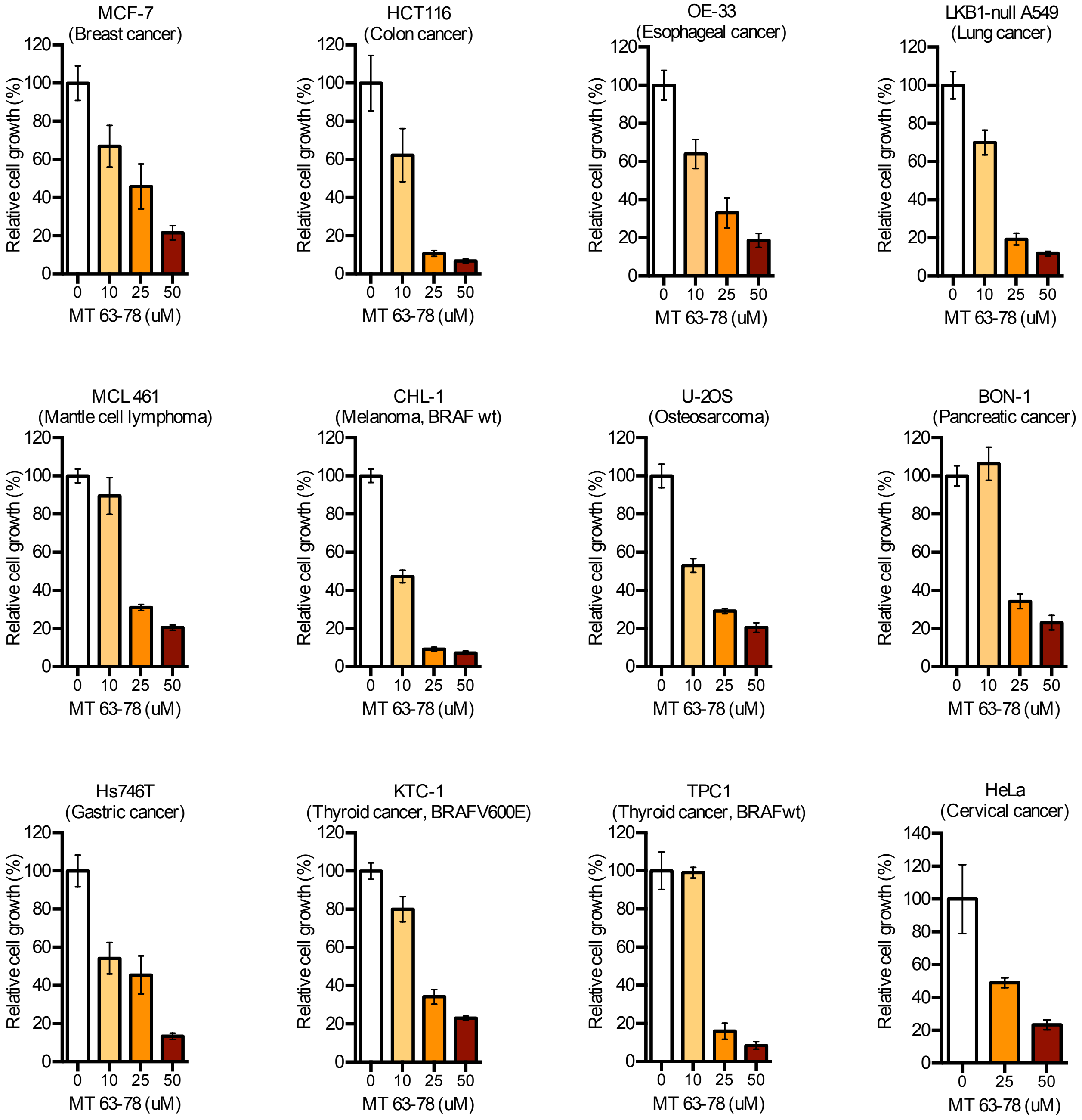


**Supporting Information Fig 6. The growth inhibitory effect of MT 63-78 is not limited to prostate cancer.**

Relative cell growth, following 72-hr treatment with 10, 25, and 50 uM MT 63-78. Results are expressed as percentage of cells compared to control (DMSO) ±SD of three independent samples.
