# Supplementary material for: A novel direct activator of AMPK inhibits prostate cancer growth by blocking lipogenesis
Source: EMBO Mol Med. 2014 Feb 4;6(4):519–38. doi: 10.1002/emmm.201302734 (PMC3992078; doi:10.1002/emmm.201302734)
Supplement: Supplementary file 15 [file emmm0006-0519-sd15.pdf]

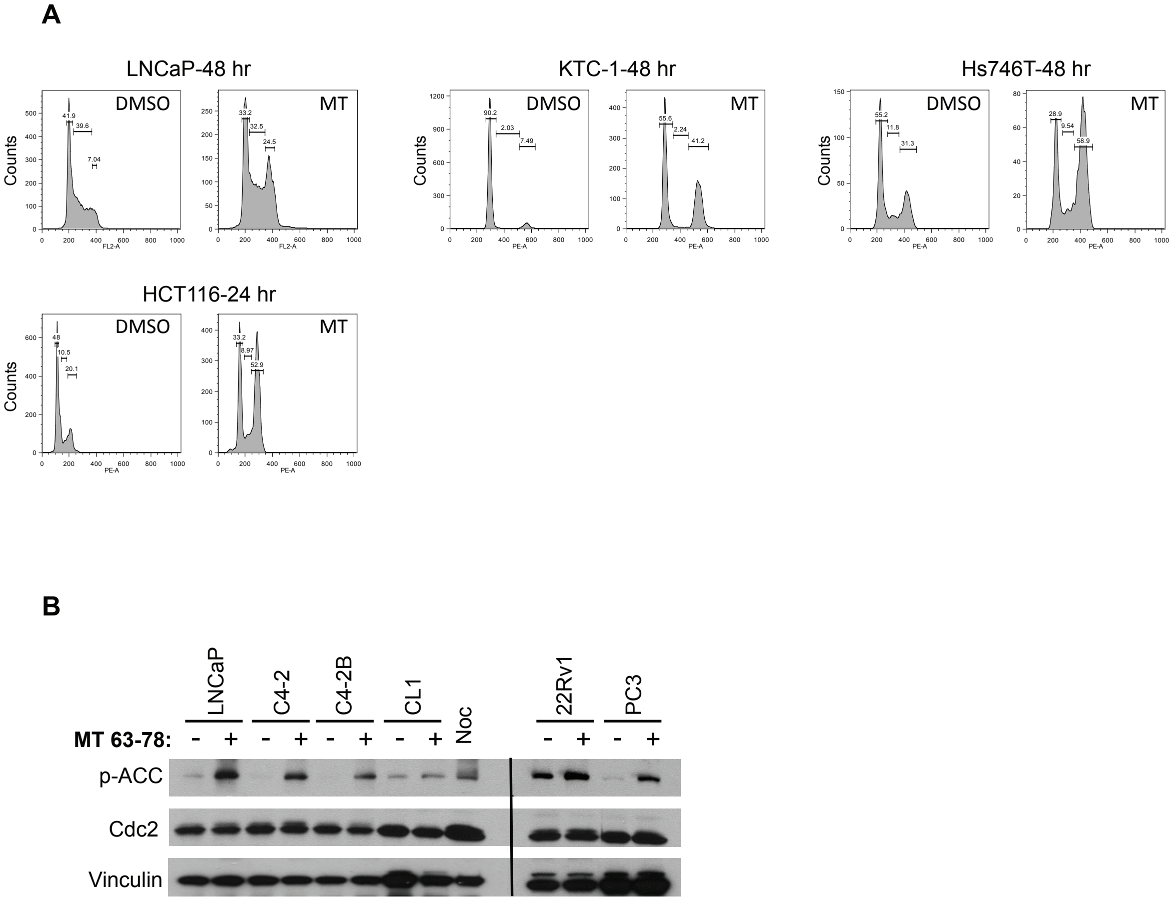

Supporting Information Fig 7. MT 63-78-induced mitotic arrest is not limited to prostate cancer.

A. Flow citometry analysis of LNCaP, KTC-1 (thyroid cancer), Hs746T (gastric carcinoma), HCT116 (colon cancer) treated with 25 uM MT 63-78 (MT) for the indicated times. Percentage of cells in G1, S, G2-M phases is indicated.

B. Cdc-2 expression levels in LNCaP and CRPC cells, following 24-hr treatment with MT 63-78 (25uM). Lysate from LNCaP cells treated with 100 ng/mL Nocodazole (Noc) for 14 hrs was used as positive control.
