# Supplementary material for: A novel direct activator of AMPK inhibits prostate cancer growth by blocking lipogenesis
Source: EMBO Mol Med. 2014 Feb 4;6(4):519–38. doi: 10.1002/emmm.201302734 (PMC3992078; doi:10.1002/emmm.201302734)
Supplement: Supplementary file 16 [file emmm0006-0519-sd16.pdf]

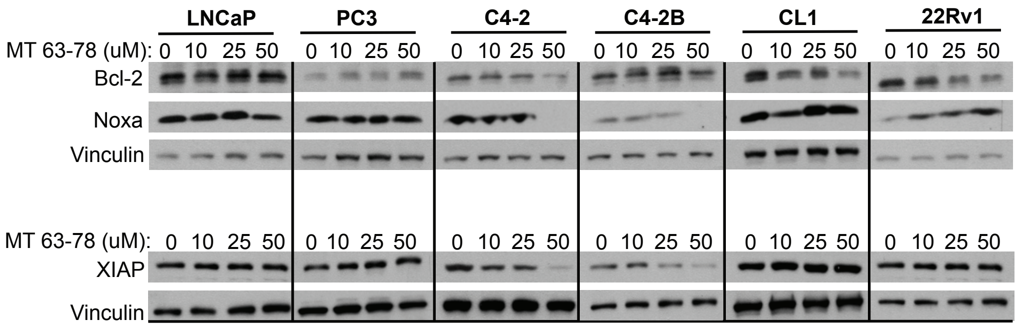

Supporting Information Fig 8. Expression of pro-survival and pro-apoptotic proteins in PCa cells, following treatment with MT 63-78.
Western blotting analysis of Bcl-2, Noxa, and XIAP expression levels in PCa cells, following 24-hr treatment with MT 63-78.
