# Supplementary material for: A novel direct activator of AMPK inhibits prostate cancer growth by blocking lipogenesis
Source: EMBO Mol Med. 2014 Feb 4;6(4):519–38. doi: 10.1002/emmm.201302734 (PMC3992078; doi:10.1002/emmm.201302734)
Supplement: Supplementary file 17 [file emmm0006-0519-sd17.pdf]

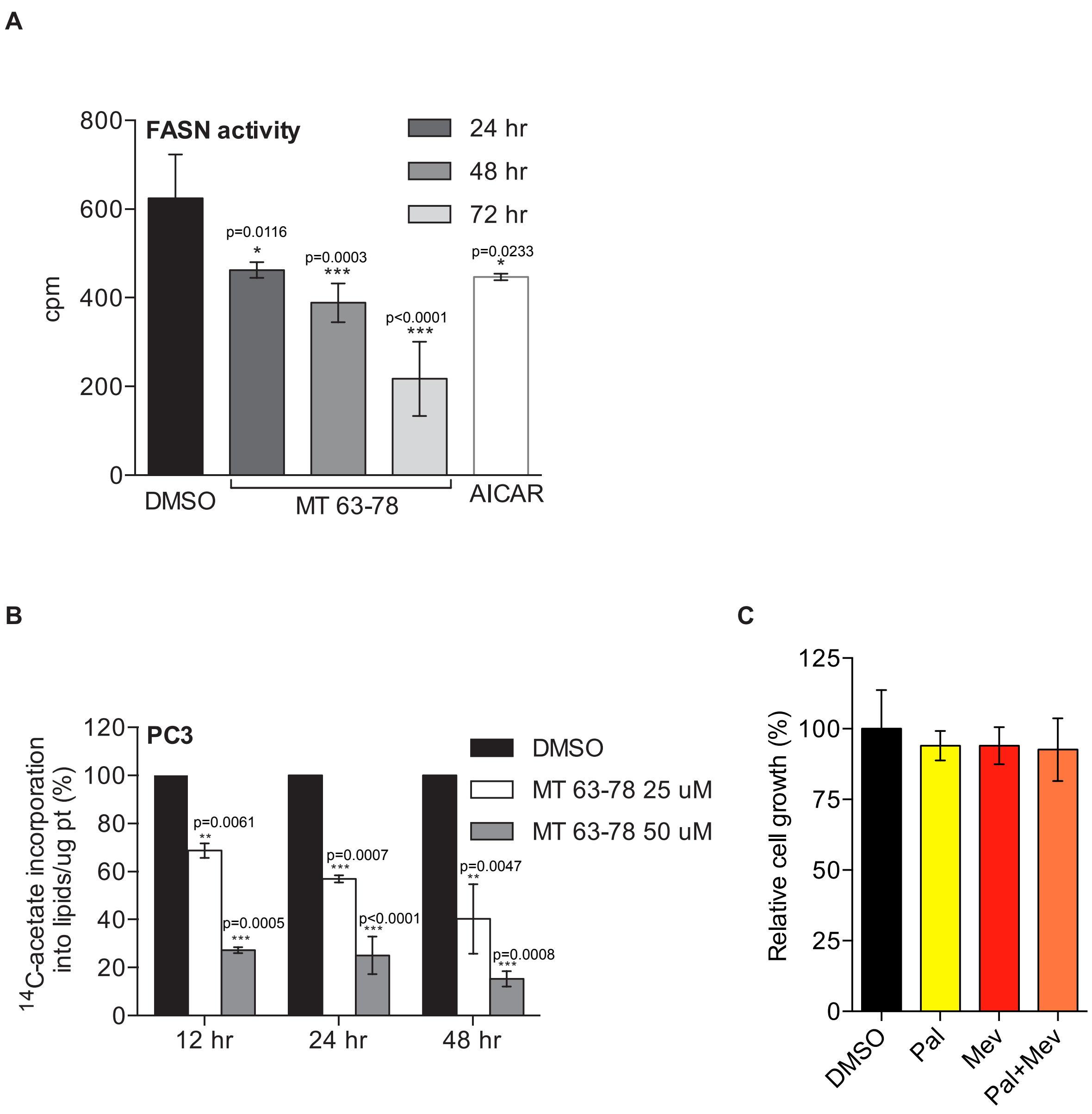

Supporting Information Fig 9. MT 63-78 inhibits FASN activity and *de novo* lipogenesis.

A. FASN activity in LNCaP cells, following treatment with MT 63-78 (25 uM) and AICAR (1mM, 48 hr) for the indicated time points. FASN activity was measured as described in the Supporting Materials and Methods. Results are expressed as means ±SD of three independent samples. One-way ANOVA test, followed by Dunnett's post hoc test for multiple comparisons was performed. Significant p values are reported on the bar graph.

B. Incorporation of ^{14}C-acetate into lipids. Results are expressed as means ±SD of three independent samples. One-way ANOVA test, followed by Dunnett's post hoc test for multiple comparisons was performed. Significant p values are reported on the bar graph.

C. Relative growth of LNCaP cells, following incubation with Palmitate (75uM) and/or Mevalonate (100uM) for 72 hr. One-way ANOVA test, followed by Dunnett's post hoc test for multiple comparisons was performed. No significant differences were observed.
