# Supplementary material for: A novel direct activator of AMPK inhibits prostate cancer growth by blocking lipogenesis
Source: EMBO Mol Med. 2014 Feb 4;6(4):519–38. doi: 10.1002/emmm.201302734 (PMC3992078; doi:10.1002/emmm.201302734)
Supplement: Supplementary file 18 [file emmm0006-0519-sd18.pdf]

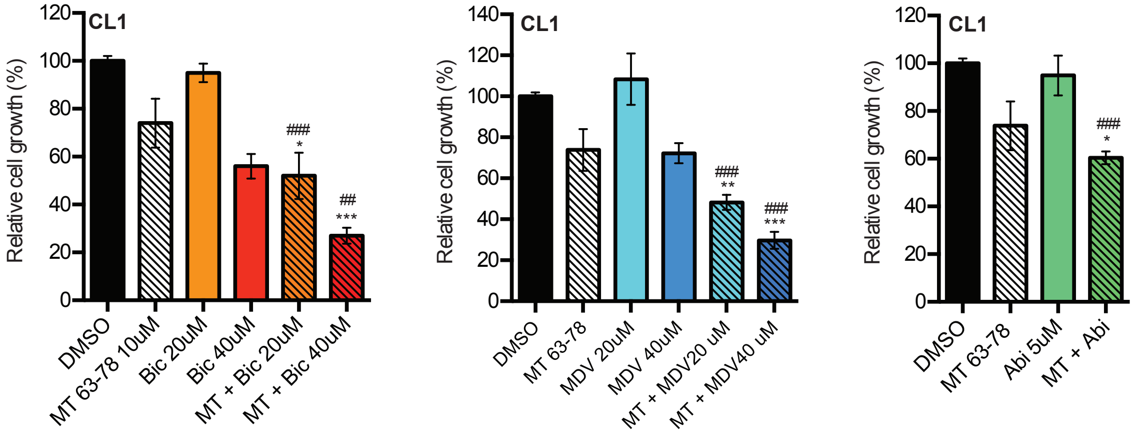

Supporting Information Fig 10. MT 63-78 enhances the growth inhibitory effect of AR signaling inhibitors in CRPC cells.

Relative growth of CL1 cells, following 3-day treatment with MT 63-78 (MT) alone or in combination with bicalutamide (Bic), MDV3100 (MDV), abiraterone (Abi) at the indicated concentrations. Results are expressed as means ±SD of three independent samples. One-way ANOVA test, followed by Tukey's post hoc test for multiple comparisons was performed. Adjusted p values were calculated (<u>Bicalutamide:</u> *p=0.013 MT *vs* MT+ Bic 20uM; ***p=8.09E-06 MT *vs* MT+ Bic 40uM; $^{\#\#\#}$p=4.29E-05 Bic 20uM *vs* MT+ Bic 20uM; $^{\#\#}$p=0.0023 Bic 40uM *vs* MT+ Bic 40uM. <u>MDV3100:</u> **p=0.0071 MT *vs* MT+ MDV3100 20uM; ***p=8.09E-06 MT *vs* MT+ MDV3100 40uM; $^{\#\#\#}$p=3.02E-06 MDV3100 20uM *vs* MT+ MDV3100 20uM; $^{\#\#}$p=0.0001 MDV3100 40uM *vs* MT+ MDV3100 40uM. <u>Abiraterone:</u> *p=0.00317 MT *vs* MT+ Abi; $^{\#\#\#}$p=0.0001 Abi *vs* MT+ Abi).
