# Supplementary material for: A novel direct activator of AMPK inhibits prostate cancer growth by blocking lipogenesis
Source: EMBO Mol Med. 2014 Feb 4;6(4):519–38. doi: 10.1002/emmm.201302734 (PMC3992078; doi:10.1002/emmm.201302734)
Supplement: Supplementary file 19 [file emmm0006-0519-sd19.pdf]

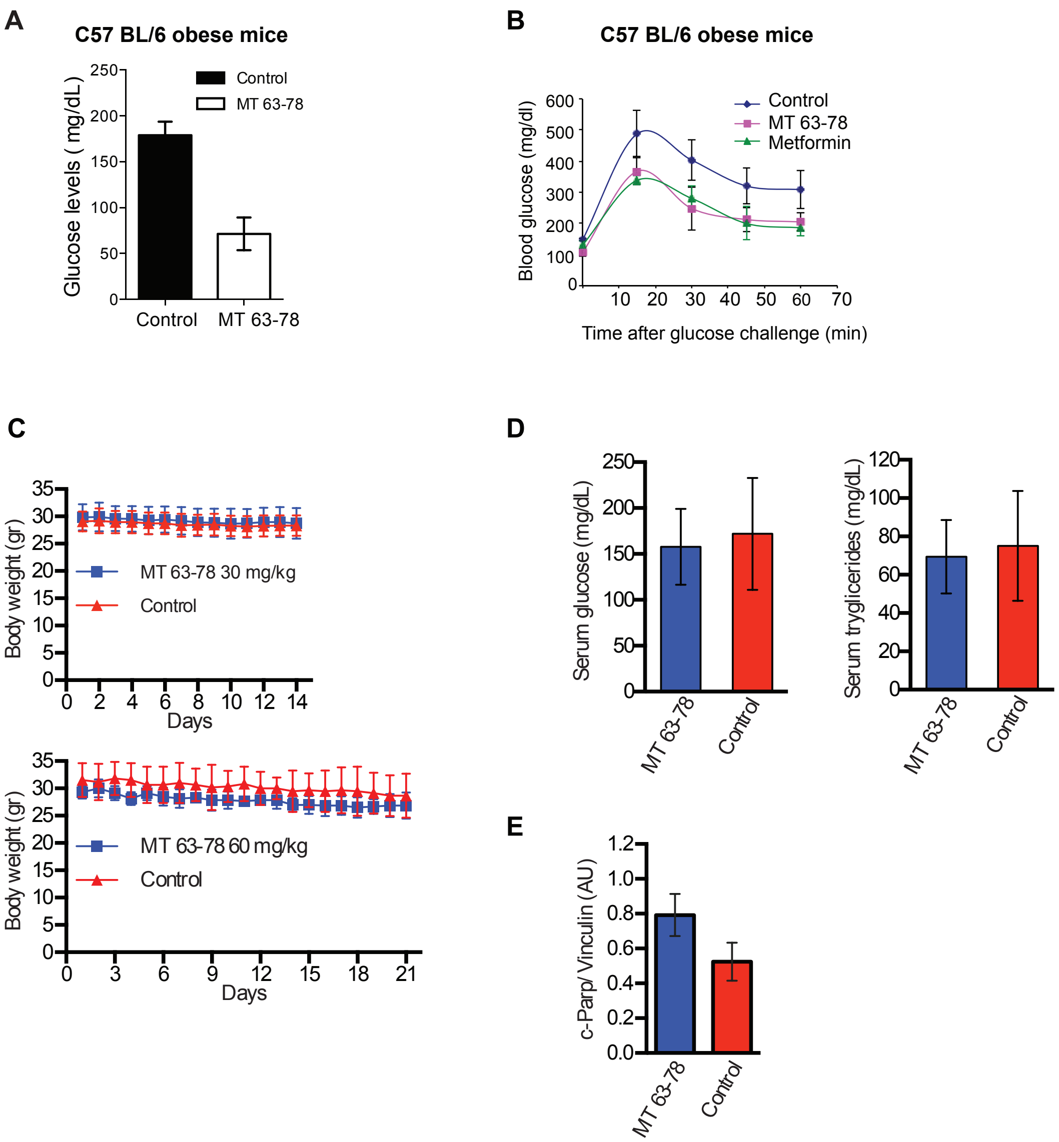

Supporting Information Fig 11. ***In vivo* effects of MT 63-78.**

A. Glucose levels in C57 BL/6 mice fed on a high fat diet (n=5), following 16-hr treatment with MT 63-78 (30 mg/kg) and overnight fasting. Results are expressed as mean ±SD.

B. Glucose tolerance test in C57 BL/6 mice fed on a high fat diet (n=5) after 5-days treatment with MT 63-78 (30 mg/kg b.i.d.), metformin (200 mg/kg b.i.d.), and vehicle. Results are expressed as mean ±SD.

C. Body weight ±SD of treated and control mice throughout the course of the study.

D. Serum glucose and triglyceride levels in 17 treated mice (MT 63-78, 30 mg/kg i.p.) and 15 controls at the end of treatment period (14 days). Values are expressed as means ±SD. Unpaired t test was performed. No significant differences were observed.

E. Densitometric analysis of cleaved-Parp (c-Parp) in tumor homogenates from treated mice (MT 63-78, 60 mg/kg i.p.) and controls at the end of treatment period (21 days). Results are normalized to Vinculin and expressed as mean ±SEM. Unpaired t test was performed. No significant difference was observed. AU= arbitrary units.
