# Supplementary material for: A novel direct activator of AMPK inhibits prostate cancer growth by blocking lipogenesis
Source: EMBO Mol Med. 2014 Feb 4;6(4):519–38. doi: 10.1002/emmm.201302734 (PMC3992078; doi:10.1002/emmm.201302734)
Supplement: Supplementary file 20 [file emmm0006-0519-sd20.pdf]

Figure 9-Statistical analysis.

A. Relative growth of LNCaP and CRPC cells C4-2 and 22Rv1, following 3-day treatment with MT 63-78 (MT), AR antagonist bicalutamide (Bic), and combined treatment (MT+Bic). Results are expressed as mean ±SD of three independent samples. One-way ANOVA test, followed by Tukey's post hoc test for multiple comparisons was performed and adjusted p values were calculated

LNCaP:

***p= 3.18E-06 MT+ Bic 10uM *vs* MT

***p= 1.61E-07 MT+ Bic 20 uM *vs* MT

***p= 1.02E-08 MT+ Bic 40 uM *vs* MT

$^{\#\#\#}$p= 1.24E-07 MT+ Bic 10uM *vs* Bic 10uM

$^{\#\#\#}$p= 3.39E-07 MT+ Bic 20 uM *vs* Bic 20uM

$^{\#\#\#}$p= 1.20E-05 MT+ Bic 40 uM *vs* Bic 40uM

C4-2:

**p= 0.0041 MT+ Bic 20 uM *vs* MT

***p= 2.04E-06 MT+ Bic 40 uM *vs* MT

$^{\#\#\#}$p= 1.83E-06 MT+ Bic 20 uM *vs* Bic 20 uM

$^{\#\#\#}$p= 4.32E-06 MT+ Bic 40 uM *vs* Bic 40 uM

22Rv1:

**p= 0.00516 MT+ Bic 20 uM *vs* MT

***p= 6.95E-06 MT+ Bic 40 uM *vs* MT

$^{\#\#}$p= 0.002 MT+ Bic 20 uM *vs* Bic 20 uM

$^{\#\#}$p= 0.002 MT+ Bic 40 uM *vs* Bic 40 uM

B. Relative growth of LNCaP and CRPC cells C4-2 and 22Rv1, following 3-day treatment with MT 63-78 (MT), AR antagonist MDV3100 (MDV), and combined treatment (MT+MDV). Results are expressed as mean ±SD of three independent samples. One-way ANOVA test, followed by Tukey's post hoc test for multiple comparisons was performed and adjusted p values were calculated.

LNCaP:

***p= 0.0001744 MT+ MDV 20uM *vs* MT

***p= 0.000153 MT+ MDV 40 uM *vs* MT

$^{\#\#\#}$p= 4.53E-07 MT+ MDV 20 uM *vs* MDV 20uM

$^{\#\#\#}$p= 0.0002 MT+ MT+ MDV 40 uM *vs* MDV 40uM

C4-2:

***p= 8.93E-06 MT+ MDV 20uM *vs* MT

***p= 4.78E-07 MT+ MDV 40 uM *vs* MT

$^{###}$p= 1.23E-09 MT+ MDV 20 uM *vs* MDV 20uM

$^{###}$p= 8.54E-06 MT+ MT+ MDV 40 uM *vs* MDV 40uM

22Rv1:

***p= 0.000286 MT+ MDV 20uM *vs* MT

***p= 5.51E-07 MT+ MDV 40 uM *vs* MT

$^{###}$p= 6.88E-06 MT+ MDV 20 uM *vs* MDV 20uM

$^{###}$p= 2.65E-06 MT+ MT+ MDV 40 uM *vs* MDV 40uM

C. Relative growth of LNCaP and CRPC cells C4-2 and 22Rv1, following 3-day treatment with MT 63-78 (MT), CYP17A1 inhibitor abiraterone (Abi) and combined treatment (MT+Abi). Results are expressed as mean ±SD of three independent samples. One-way ANOVA test, followed by Tukey's post hoc test for multiple comparisons was performed and adjusted p values were calculated.

LNCaP:

***p= 1.92E-07 MT+ Abi *vs* MT

$^{###}$p= 1.17E-05 MT+ Abi *vs* Abi

C4-2:

**p= 0.0012 MT+ Abi *vs* MT

$^{###}$p= 6.5E-11 MT+ Abi *vs* Abi

22Rv1:

***p= 0.00013 MT+ Abi *vs* MT

$^{###}$p= 7.33E-06 MT+ Abi *vs* Abi
