# Supplementary material for: A novel direct activator of AMPK inhibits prostate cancer growth by blocking lipogenesis
Source: EMBO Mol Med. 2014 Feb 4;6(4):519–38. doi: 10.1002/emmm.201302734 (PMC3992078; doi:10.1002/emmm.201302734)
Supplement: Supplementary file 21 [file emmm0006-0519-sd21.pdf]

Supporting Materials and Methods

Chemicals

MT 63-78 (Mercury Pharmaceuticals, Inc., Woburn, MA) was dissolved in DMSO and in 5% hydroxypropyl beta-cyclodextrine for *in vitro* and *in vivo* studies, respectively. 0.25% Polyvinylpyrrolidone (PVP, Sigma-Aldrich) dissolved in water was added to 5% hydroxypropyl beta-cyclodextrine to increase drug solubilization. A-769662 (LC Laboratories) was dissolved in DMSO. AICAR (Toronto Research Chemicals Inc.), simvastatin, 5-(tetradecyloxy)-2-furoic acid (TOFA), STO-609, (±)-mevalonolactone, C75, rapamycin (Biomol International), metformin, ALLN, acetyl-CoA, malonyl-CoA (Sigma-Aldrich) were dissolved according to the manufacturer's guidelines. Lysosomal inhibitors Cloroquine and Bafilomycin A1 (Sigma-Aldrich) were dissolved in water and DMSO, respectively Bicalutamide (Sigma-Aldrich), MDV3100 and Abiraterone (Selleck Chemicals LLC) were dissolved in DMSO. Palmitic acid (Cayman Chemical) was dissolved in ethanol and complexed to FA-free BSA (Sigma-Aldrich). Recombinant AMPK complexes, CaMKKβ, and PP2Cα are commercial available (Sigma-Aldrich and R&D systems*)*.

Cell lines and culture conditions

Cells (LNCaP, DU145, PC3, HeLa, 22Rv1, HCT-116, U-2OS, HepG2) were purchased from ATCC and maintained at 37°C and 5% CO2. Cell lines were cultured in standard conditions, as described by ATCC. For LNCaP-derived CL1 cells, 10% charcoal stripped serum was used. Immortalized human prostate epithelial cells iPrEC [a gift from Dr William Hahn, Dana Farber Cancer Institute (DFCI), Boston, MA] were grown in PrEBM medium supplemented with PrEGM Single Quots (Lonza). RWPE-1 cells were grown in MEM medium supplemented with 10% FBS, 1 mM MEM non-essential amino acids, 1 mM Na-pyruvate, 10 ug/ml gentamicin, and 1 mM glutamine. C4-2 and C4-2B cells were kindly provided from Drs. Steven Balk and Pier Paolo Pandolfi [Beth Israel Deaconess Medical Center (BIDMC), Boston, MA) and they were grown in RPMI, 10% FBS, 1% penicillin/streptomycin. A549 and MCL461 (from the laboratory of Matthew Meyerson, DFCI), Hs46T and CHL-1 (from the laboratory of Dr. Levi Garraway, DFCI), MCF-7 (from the laboratory of Dr. Kornelia Polyak, DFCI), OE-33 (from the laboratory of Dr. Adam Bass, DFCI), BON-1 [from the laboratory of Dr. Umar Mahmood, Massachusetts General Hospital (MGH), Boston, MA], KTC-1 and TPC-1 (from Dr. Carmelo Nucera, BIDMC) cells were cultured in standard conditions, as previously described. AMPK $\alpha1^{-/-}$ and $\alpha2^{-/-}$ and LKB1$^{-/-}$ MEFs were obtained from Dr. Benoit Viollet (INSERM, Paris, Fr) and Nabeel El-Bardeesy (MGH), respectively.

Western blotting

Cells were lysed in 1% NP-40 buffer (20 mM Tris-HCl, 150 mM NaCl, 1 mM EDTA, 1 mM EGTA, 1% NP-40) with the addition of phosphatases and complete protease inhibitor cocktail (Roche). To detect mature form of SREBP-1, cells were incubated with 50 ug/mL ALLN 6 hr before lysis. ALLN (25 ug/mL) was also added in the lysis buffer. For tumor xenograft samples, tissues were homogenized in 1% NP-40 buffer using Tissue-Tearor homogenizer (BioSpec Products). Protein quantification was performed using a Bradford protein assay (Bio-Rad). Equal amounts of protein (20-30 ug) were resolved on precast 10%, 4-12%, 4-20% Tris-glycine SDS-polyacrylamide gels (Invitrogen). Bands were quantified by densitometry using the Quantity One software, version 4.6.1 (Bio-Rad). Results were normalized to β-actin or Vinculin and were expressed in arbitrary units. Primary antibodies were all from Cell Signaling Technology, except for anti-CaMKK (Abcam), anti-FASN, anti-SREBP-1 (BD Transduction laboratories), anti-Beclin (Santa Cruz Technology), anti-LC3-II/LC3-I (MBL), anti-Noxa (Calbiochem) anti-β-actin, anti-Vinculin (Sigma). Antibodies were used at concentrations suggested by the manufacturer.

Western blotting in conditioned media was performed after floating cells were collected by centrifugation and lysed.

Hoechst 33342 staining

Cells (1-3x10^5) were plated in 1-well Chamber Slides (Nunc™ Lab-Tek™) and treated for 24 hrs with MT 63-78 (25uM) or DMSO. Nuclei were stained with Hoechst 33342 (Invitrogen), following standard procedures. Briefly, cells were first washed three times with PBS and fixed in 4% paraformaldehyde solution for 20 min. Cells were then washed three times with PBS, quenched with 50 mM NH_4Cl, permeabilized in 0.1% Triton X-100/PBS for 5 min, and washed again twice with PBS. Cells were incubated with Hoechst 33342 (1uM) for 5min and washed again three times with PBS. Coverslips were mounted on slides with Vectashield Mounting medium (Vector Laboratories). Images were captured with a 40x objective and analyzed using Olympus BX50 microscope and RETIGA EX*i* Fast 1394 CCD camera (QImaging).

Soft agar assays

1x10^3 PC3 cells were resuspended with 0.4% agar (Becton Dickinson) in DMEM/Ham F12 medium and seeded in triplicate in six-well plates coated with 0.6% agar. After 3 weeks, colonies larger than 0.5 mm were counted both by eye using a dissecting microscope and by the use of the publicly available Image J software (version 1.41).

Cell cycle and apoptosis studies

The percentage of cells in G1, S, and G2-M phase of the cell cycle was analyzed with a BD LSR II flow cytometer (Becton Dickinson), after PI staining. Expression/ activation of mitotic markers and proteins involved in mitosis was analyzed by western blotting using antibodies against cyclin B1, cdc2, Aurora Kinases A and B and their phosphorylated forms, PLK1, phospho-Histone H3. Apoptosis was analyzed both by detection of cleaved Caspase 3 (c-Caspase 3) and cleaved Parp (c-Parp) in cell lysates and by measuring membrane redistribution of phosphatidylserine using Apo Alert Annexin V-FITC/PI apoptosis kit (Clontech), as described by the manufacturer. Flow citometry data were analyzed using the software package FloJo (versions 9.6.4 and 7.6.5). Expression/ activation of BH3 family members (Puma, Noxa, Mcl-1, Bcl-2) and anti-apoptotic XIAP was analyzed by western blotting.

Pharmacokinetics of MT 63-78

MT 63-78 (2 mg/ Kg or 10 mg/ kg) was administered i.p. or orally using the antiacid Mylanta as vehicle in three C57 BL/6 male mice. MT 63-78 concentration in the plasma was measured by high-performance liquid chromatography (HPLC)/mass spectrometry (MS). Briefly, an aliquot of plasma was mixed with three volumes of methanol containing internal standard (propranolol, diclofenac, etc.). The samples were incubated 10 min on ice and centrifuged. The supernatant was filtered using the Captiva system (Varian). The filtrate was then analyzed by LC/MS/MS using an Agilent 6410 mass spectrometer coupled with an Agilent 1200 HPLC and a CTC PAL chilled autosampler. After separation on a C18 reverse phase HPLC column (Agilent) with an acetonitrile-water gradient system, peaks were analyzed by MS, using ESI ionization in MRM mode. Oral bioavailability of MT 63-78 was calculated as the ratio AUC (oral dose)/AUC (i.p. dose). AUC is the area under the curve measured in the plot of MT 63-78 concentration *vs* time. AUC (i.p. dose) was set as 100%.

Measurement of AMPK activity

To measure AMPK activity *in vitro*, the complex α1β1γ1 was cloned and expressed in a baculovirus/Sf9 system. Twenty ng of human recombinant GST-ACC peptide (1-150) were incubated with 1 ng of recombinant α1β1γ1 in the reaction mix (80 mM Hepes-Na, pH 8, 160 mM NaCl, 2.5 mM $MgCl_2$, 1mM DTT, 0.08% Tween-20, 100 uM ATP) at 30°C for 30 min. Reaction was ended by addition of 5 ul of Laemmli's buffer and subjected to gel electrophoresis. Phosphorylation of the ACC peptide was used as readout of AMPK activation, by using anti-Phospho-ACC (Ser79) antibody. *In vitro* AMPK activity, using recombinant α1β1γ1, α1β2γ1, α2β1γ1, α2β2γ1 heterotrimers, was also assessed by ACC phosphorylation using alpha (amplified luminescent proximity homogeneous assay) screen technology

(Perkin Elmer), as described by manufacturer. Briefly, naturally biotinylated ACC is conjugated with a streptavidin donor bead and incubated with AMPK recombinant heterotrimer in the reaction mix. Phosphorylation of ACC is detected with an antibody raised against the phosphorylated Serine 79 and conjugated to an acceptor bead. The excitation of the donor bead (680 nm) provokes the release of singlet oxygen molecules that triggers a cascade of energy transfer in the acceptor bead, resulting in the emission of luminescence signal at 520-620 nm. Luminescence was measured at 540 nm with spectraMax Plus spectrophotometer.

To measure AMPK activity in cell lysates, we used both the semi-quantitative immunoassay CycLex AMPK Kinase Assay Kit (MBL International Corporation) as well as the measurement of ACC phosphorylation with alpha screen technology described above. For CycLex AMPK Kinase Assay, 700x10^3 LNCaP and 650x10^3 PC3 cells were seeded in 60-mm dishes for 24 hr, after which different concentrations of MT 63-78 were added for 30 min. Cells were lysed and AMPK activity was measured following the manufacturer's instructions. Absorbance was read at 450 nm with spectraMax Plus spectrophotometer. Results were normalized to protein content, after background subtraction.

Measurement of ATP, ADP, AMP levels

Intracellular ATP and ADP levels in PCa cells were measured using bioluminescence EnzyLight^{TM} ADP/ATP Ratio Assay Kit (ELDT-100), EnzyLight^{TM} ATP Assay Kit (EATP-100), and EnzyLight^{TM} ADP Assay Kit (EADP-100) (BioAssay Systems), as described by the manufacturer. Intracellular ATP, ADP, and AMP levels in HepG2 were measured by HPLC, as previously described (Manfredi et al, 2002).

AMPK dephosphorylation assay

Recombinant AMPK α1β1γ1 (100 ng) was incubated in the presence of recombinant CaMKKβ (100 ng) with 200 uM of ATP, 2.5 mM $MgCl_2$, 1mM DTT for 20 min at 37 °C. An aliquot of this reaction (5 ul) was incubated in 50 mM Hepes, pH 7.4, 2.5 mM $MgCl_2$ in the presence or absence of recombinant PP2Cα (26 ng) and in the presence or absence of 5 μM MT 63-78 for 20 min at 37 °C. Reactions were terminated by the addition of SDS-gel loading buffer. Samples were then subjected to western blot analysis. AMPK phosphorylation was detected using an antibody against the residue Thr172 on the α subunit [Phospho-AMPK〈 (Thr172) (40H9) Rabbit mAb, Cell Signaling Technology].

siRNA and plasmids for transient transfection experiments

LNCaP and PC3 cells were transfected with 80 pmols of siRNA for AMPK α1 and α2 (sc-45312, Santa Cruz Technology), 10 nM of siRNA targeting the β1 AMPK isoform (M-007675-00-0005, siGENOME, smart pool, Dharmacon), 25 nM of siRNA targeting the β2 AMPK isoform (M-007672-00-0005,

siGENOME, smart pool, Dharmacon), and a scrambled control siRNA (Control siRNA-A, sc-37007, Santa Cruz Technology; siGENOME Non-Targeting siRNA Pool #1, D-001206-13-05, Dharmacon) using Lipofectamine RNAi Max (Invitrogen), as described by the manufacturer. For mTORC1 rescue experiments, 1 ug of empty vector (pKH3-EV) or constitutively active, rapamycin-insensitive S6 kinase 1 vector (pKH3-HA- S6K1-F5A-T389E-R3A), kindly provided by Dr. Blenis (Harvard Medical School, Boston, MA), were transfected in LNCaP and PC3 cells using Fugene (Roche), following the manufacturer's instructions.

FASN activity assay

FASN activity was measured by incorporation 2-^{14}C-malonyl-CoA (Perkin Elmer) into lipids. Briefly, cells were washed, pelleted, and resuspended in hypotonic buffer (1mM DTT, 1 mM EDTA, 20 mM Tris-HCl, pH 7.5) for 15 min. Lysate (about 20 ug of protein) was mixed with 125 ul of NADPH solution (100 mM potassium phosphate, pH 7, 100mM KCl, 0.5 mM NADPH) and incubated at 37° C for 2 min. A substrate mixture (25nml acetyl-CoA, 25 nmol malonyl-CoA, 0.05 uCi of 2-^{14}C-malonyl-CoA) was then added and incubated at 37° C for 10 min. Reaction was stopped with 1N HCl/methanol (6:4, v/v). Lipids were extracted with petroleum ether. Radioactivity was measured by scintillation counting.

2-^{14}C-acetate incorporation into lipids and Thin Layer Chromatography (TLC)

Cells were incubated with 2-^{14}C-labeled acetate (57 mCi/mmol; 2 μCi/dish; Amersham International) for 4 hrs. After incubation, cells were collected by centrifugation, resuspended in 200 ul PBS, mixed with 750 ul methanol-chloroform (2:1, v/v) in glass tube, and stirred for 30 min. Two hundred-fifty ul of chloroform and 250 ul of water were added to complete the lipid extraction, as described by Bligh and Dyer (1959). 2-^{14}C-acetate incorporation into cellular lipids was quantitated by scintillation counting and normalized to protein content. Analysis of 2-^{14}C-acetate incorporation into neutral and polar lipids species were carried out as previously described (Bagnato and Igal, 2003), with some modifications. Briefly, neutral lipid species and total polar lipids were separated and identified on silica gel 60 TLC plates (EMD Chemicals) using hexane/diethyl ether/acetic acid (80:20:2, vol/vol/vol) as solvent system. Individual lipid spots detected by autoradiography were scraped into glass vials and radioactivity quantified as described above.

2-^{14}C -acetate oxidation experiments

LNCaP cells were seeded in 12-well plates for 24hr. Drugs were added to the medium 4 hr prior incubation with 2-^{14}C-acetate (2 uCi) for 2 hr at 37°C. Hundred ul of 70% perchloric acid was added to each well, firmly covered with a piece of Wathman filter paper soaked with NaOH (3M). Plates were

rocked for 2 hr at RT. The production of $^{14}C\text{-}CO_2$ was measured by scintillation counting and normalized to protein content.

Xenograft model and positron emission tomography (PET) imaging

LNCaP cells ($1x10^6$ in 100 ul PBS) were mixed with 100 ul of matrigel (BD Biosciences) and injected in the left side of thirty-two 8-week-old male nude mice (Charles River, MA). Treatment with MT 63-78 (30 mg/kg) started when the average tumor volumes reached around 50-100 mm^3 and was performed i.p. (once a day) for 14 days. Experiments were performed twice for a total number of 17 mice under treatment and 15 controls (treated with 5% hydroxypropyl beta-cyclodextrine as vehicle). A second study was performed using higher dose of MT 63-78 (60 mg/kg) and prolonged treatment (21 days). Six mice under treatment and six controls (treated with 5% hydroxypropyl beta-cyclodextrine plus 0.25% PVP to improve drug solubility as vehicle) were used. One treated mouse was removed from the study since biochemical analysis showed that MT 63-78 did not penetrate in the tumor for unknown reasons. Tumor size, and weight levels were measured every other day and every day, respectively. Tumor volume was calculated using the formula (π/6)*A*B2 (A= larger diameter of the tumor, B= smaller diameter of the tumor). At the end of treatment, mice were sacrificed by CO_2 asphyxiation. Terminal blood was taken by cardiac venipuncture and serum was collected by centrifugation using microtainer amber tubes with serum separator (Becton Dickinson). Tumors were measured after excision, snap-frozen and stored at -80°C for molecular analysis. For imaging studies, three male, nu/nu mice with palpable tumor (on the right side) were used to study ^{11}C-acetate uptake before and after 2 treatments with MT 63-78 (30 mg/kg) or vehicle (5% hydroxypropyl beta-cyclodextrine). Two mice were i.p. injected with the compound 24 hr and 1 hr before 2nd scan. One mouse received MT 63-78 injection intra-tumor. One mouse treated with AICAR (400mg/kg) and one with the FASN inhibitor C75 (30 mg/kg) were also included in the study as controls. Mice were fasted for 4hr before image acquisition and were kept warm using a heating lamp and heating pad. Isofluorane (5% and 2%) was used as an inhaled anesthetic to induce and maintain anesthesia, respectively. Imaging was performed using small-animal PET scanner (Concorde Microsytems). Images were acquired for 7 minutes on 2 bed positions (energy window 250-700), 15 minutes following intravenous tail injection of 14.8–18.5 MBq (400-500 μCi) of ^{11}C-acetate. Late images were acquired for 15 min, 1 hr after radiotracer injection. Images were reconstructed using 2-dimensional ordered-subset expectation maximization algorithm (2D OSEM). PET images were evaluated by analysis of the standardized uptake value (SUV) of the tumor using ASIPro VM software (Siemens Medical Solutions, Inc.) At the end of the imaging, mice were euthanized, and the tumors were excised and snap frozen for western blot analysis.

***Genetically engineered mouse models* (GEMMs)**

AMPK $\alpha 2^{-/-}$ mice (C57 BL/6), kindly provided from Benoit Viollet, were intercrossed with FASN-Tg mice (FVB), previously generated in our laboratory (Migita et al, 2009). Breeding was performed for several generations to generate FASN-Tg/ AMPK $\alpha 2^{+/+}$, FASN-Tg/ AMPK $\alpha 2^{+/-}$, and FASN-Tg/ AMPK $\alpha 2^{-/-}$ with mixed genetic background. Genotyping for the presence of FASN transgene was performed by PCR using three pairs of primers: forward 5′-TGCACCTTGTCAGTGAGGTC-3′ and reverse 5′-CGGAGTGAATCTGGGTTGAT-3′ located in the 3′ region of the rat probasin promoter and in the 5′ region of the *FASN* transgene, generating a 376-bp amplicon; forward 5′-CCAGGGATTTCAGTCGATGT-3′ and reverse 5′-AATCTCACGCAGGCAGTTCT-3′ located in the luciferase gene, generating a 185-bp amplicon; and forward 5′-CTAACGTTACTGGCCGAAGC-3′ and reverse 5′-AGGAACTGCTTCCTTCACGA-3′ located in the IRES sequence, generating a 202-bp amplicon. Genotyping to confirm the genetic ablation of AMPK $\alpha 2$ was performed by using Primer lox F1 (forward): 5'-GCTTAGCACGTTACCCTGGATGG-3' and Primer lox R1 (reverse): 5'-GCATTGAACCACAGTCCTTCCTC-3', generating a 200-pb amplicon for the wild type allele and a 600-pb amplicon for the floxed allele.

Glucose levels and glucose tolerance test in mouse models of obesity and insulin resistance.

C57 BL/6J mice were fed on high fat diet (fat 35 g%). These mice develop obesity, mild to moderate hyperglycemia, and hyperinsulinemia, thus they are commonly used as model of type 2 diabetes and obesity. After 16 hr of treatment with 30 mg/kg MT 63-78 and overnight fasting, glucose levels were measured in a drop of blood taken by vein tail sampling, utilizing a glucometer (Abbott). Glucose tolerance test was performed after 5 days of i.p. treatment with MT 63-78 (30 mg/kg, b.i.d) or metformin (200 mg/kg, bid) using standard procedure. Briefly, mice were fasted overnight (16 hr), after which glucose levels were measured as baseline (t=0). Glucose solution (2 g/kg) was i.p. injected and glucose levels were measured at 15, 30, 45, 60 min after injection.

Histopathology

Prostates were removed from mice sacrificed at the age of 13-16 months. Prostates were micro-dissected to isolate the anterior, ventral, and dorsolateral lobes. Dissected tissue samples were fixed overnight in 10% formalin, processed in a Tissue-Tek VIP processing instrument (Sakura), and embedded in paraffin. Sections of formalin-fixed paraffin-embedded (FFPE) tissues (5 μm thick) were stained with hematoxyl and eosin and blindly analyzed by an expert pathologist (Sabina Signoretti) for the presence of hyperplasia, dysplasia, and prostatic intraepithelial neoplasia (PIN).
