# Supplementary material for: A novel direct activator of AMPK inhibits prostate cancer growth by blocking lipogenesis
Source: EMBO Mol Med. 2014 Feb 4;6(4):519–38. doi: 10.1002/emmm.201302734 (PMC3992078; doi:10.1002/emmm.201302734)
Supplement: Supplementary file 22 [file emmm0006-0519-sd22.pdf]

Supporting Information

Zadra G et al, "A novel direct activator of AMPK inhibits prostate cancer growth by blocking lipogenesis".

Table of Content

Supporting Materials and Methods pp. 2-8

Supporting References p.9

Supporting statistical analysis pp.10-11

Supporting Figures

Supporting Figure 1 p.12

Supporting Figure 2 p.13

Supporting Figure 3 p.14

Supporting Figure 4 p.15

Supporting Figure 5 p.16

Supporting Figure 6 p.17

Supporting Figure 7 p.18

Supporting Figure 8 p.19

Supporting Figure 9 p.20

Supporting Figure 10 p.21

Supporting Figure 11 p.22

Supporting References

1. Bagnato C, Igal RA (2003) Overexpression of diacylglycerol acyltransferase-1 reduces phospholipid synthesis, proliferation, and invasiveness in simian virus 40-transformed human lung fibroblasts. *J Biol Chem* **278:** 52203-52211

2. Bligh EG, Dyer WJ (1959) A rapid method of total lipid extraction and purification. *Can J Biochem Physiol* **37:** 911-917

3. Manfredi G, Yang L, Gajewski CD, Mattiazzi M (2002) Measurements of ATP in mammalian cells. *Methods* **26:** 317-326

4. Migita T, Ruiz S, Fornari A, Fiorentino M, Priolo C, Zadra G, Inazuka F, Grisanzio C, Palescandolo E, Shin E, *et al* (2009) Fatty acid synthase: a metabolic enzyme and candidate oncogene in prostate cancer. *J Natl Cancer Inst* **101:** 519-532
